# Supplementary material for: Deciphering the transcriptomic regulation of heat stress responses in Nothofagus pumilio
Source: PLoS One. 2021 Mar 30;16(3):e0246615. doi: 10.1371/journal.pone.0246615 (PMC8009359; doi:10.1371/journal.pone.0246615)
Supplement: S1 File — (PDF) [file pone.0246615.s001.pdf]

List of Figures

S1 **Pearson's correlation test between biological replicates** 2
S2 **Length distribution of *Nothofagus pumilio* assembled transcripts** 3
S3 **Differentially expressed contigs** 4
S4 **Semantically reduced overrepresented Gene Ontology molecular functions in genes repressed (A) and promoted (B) in response to high temperature** . . . 5
S5 **Semantically reduced overrepresented Gene Ontology cellular components in genes repressed (A) and promoted (B) in response to high temperature** . . . 6
S6 **Semantically reduced overrepresented Gene Ontology biological processes (A), molecular functions (B), and cellular components (C) in genes promoted by high temperature in *N. pumilio*, *A. thaliana* and *P. tomentosa*** 7
S7 **Maximum Likelihood phylogenetic trees for genes of interest.** 8

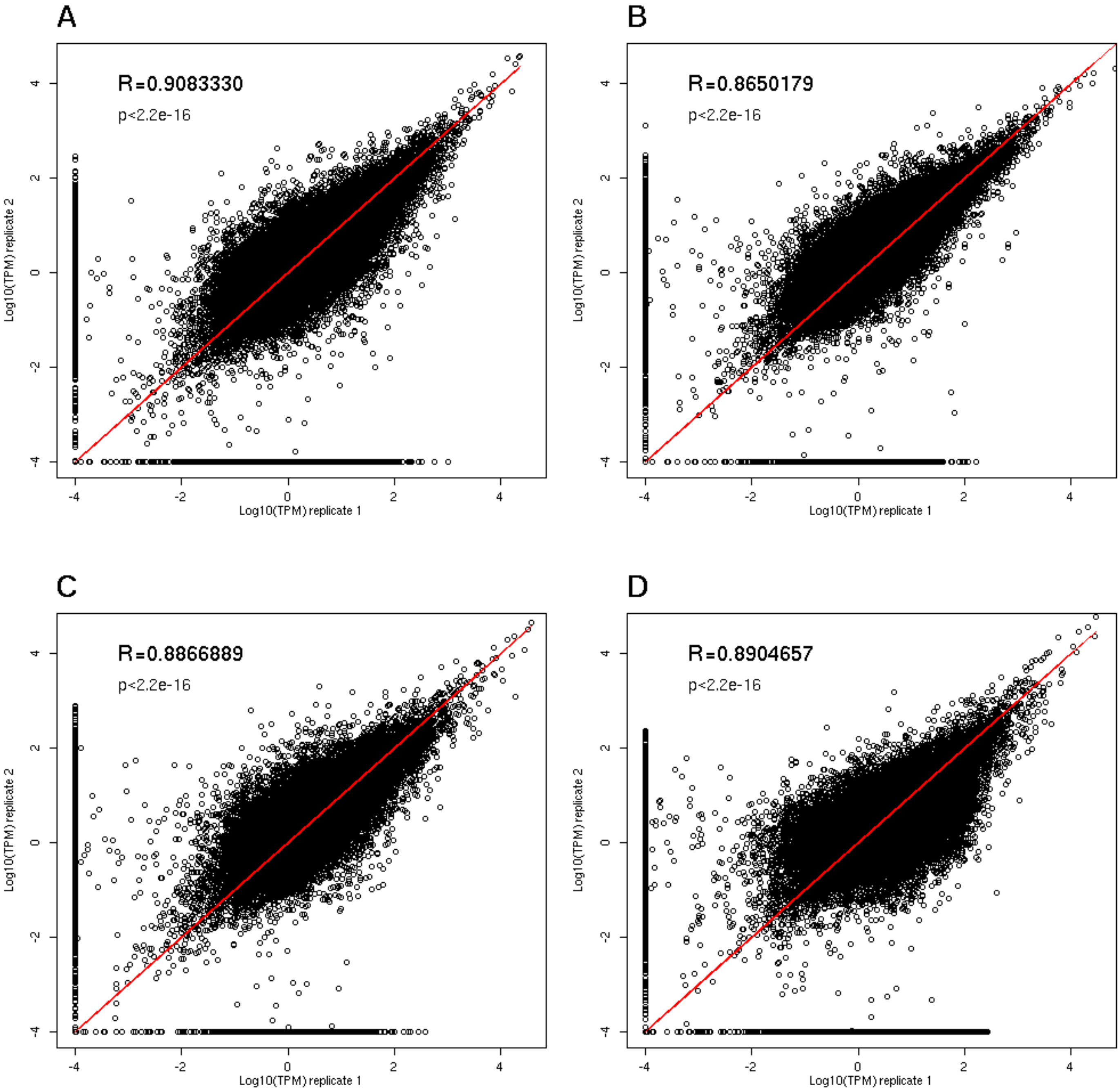

Figure S1: **Pearson's correlation test between biological replicates.** **A**: 20°C, 48 hours after onset of temperature treatment (h.a.t.). **B**: 20°C, 60 h.a.t. **C**: 34°C, 48 h.a.t. **D**: 34°C, 60 h.a.t. Low TPM (transcripts per million) values are represented together at the lower end of both axes for better visualization

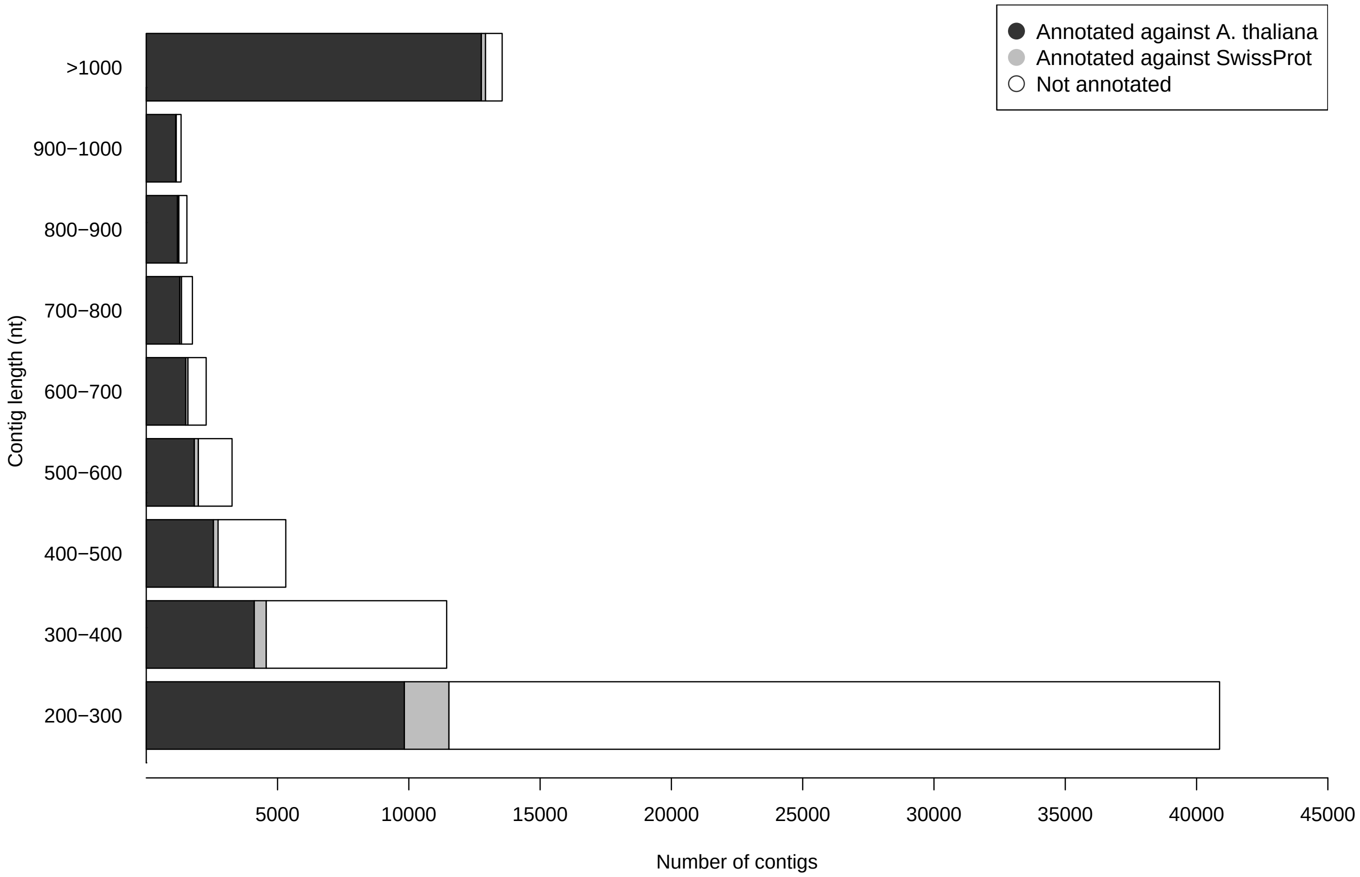

Figure S2: **Length distribution of *Nothofagus pumilio* assembled transcripts.** Each bar discriminates between annotated and unannotated contigs of the corresponding length interval

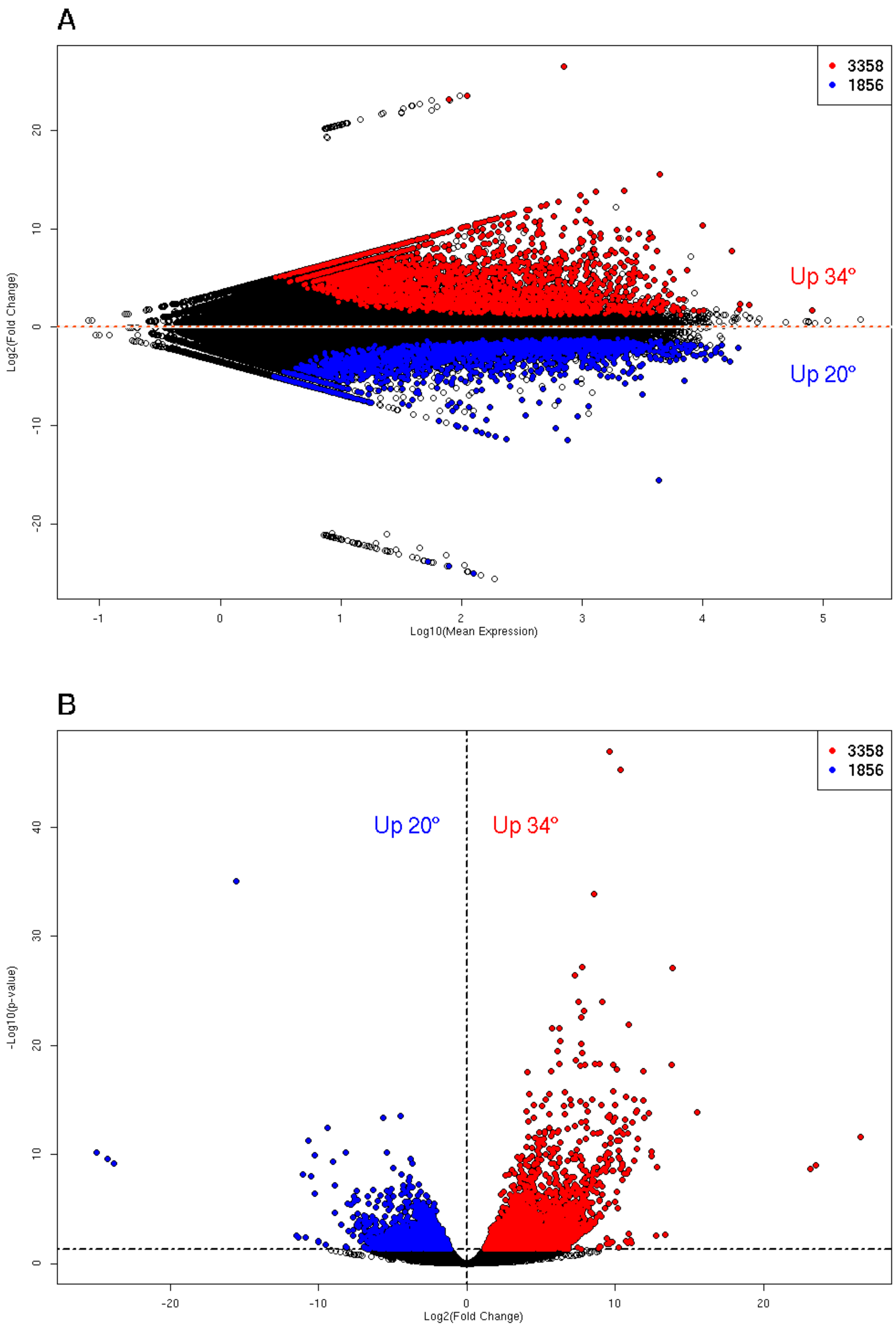

Figure S3: **Differentially expressed contigs. A**: MA-plot. **B**: Volcano plot. **Red** and **Blue**: differentially expressed contigs. **Black**: contigs not differentially expressed. Total contigs: 81761

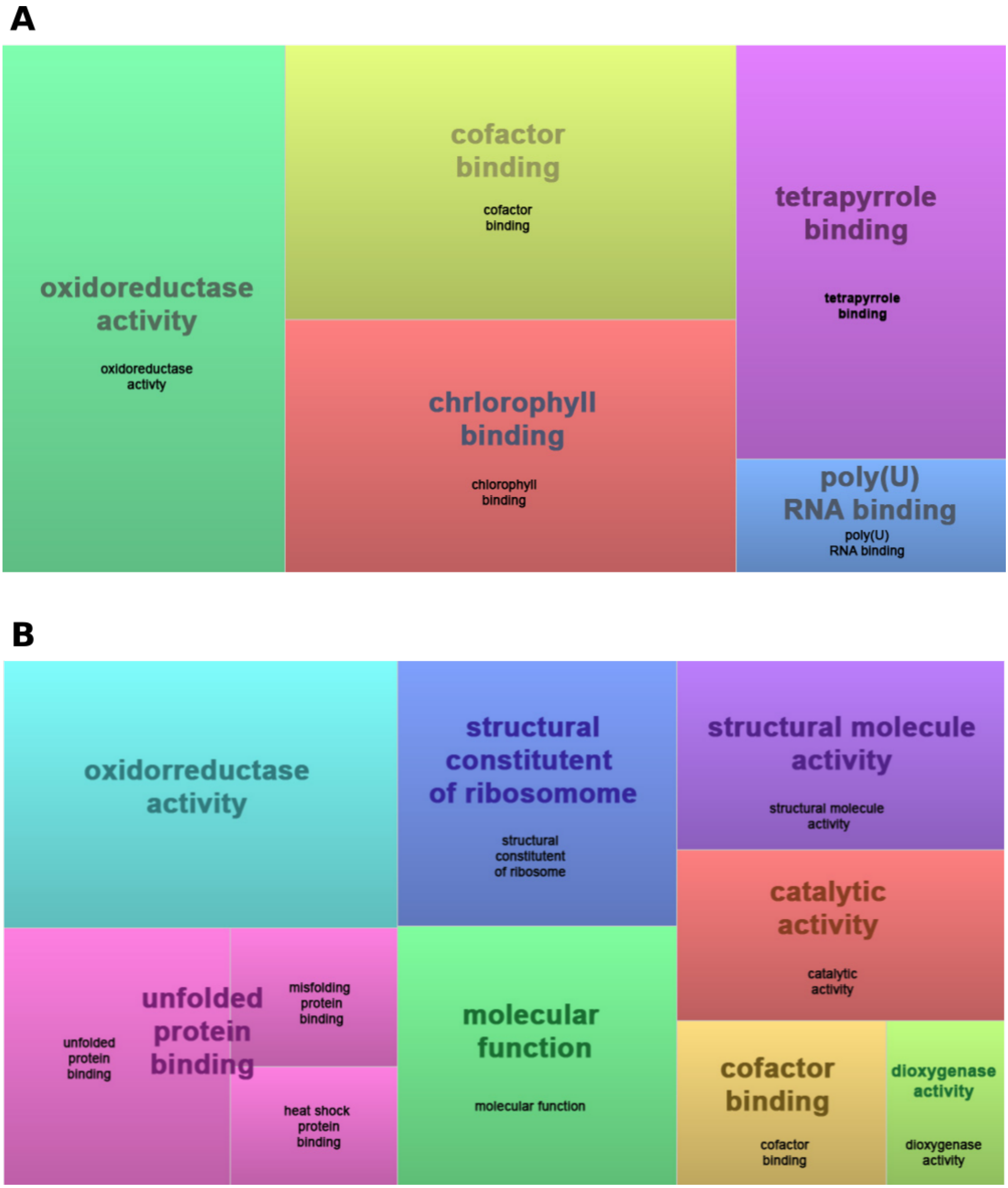

Figure S4: **Semantically reduced overrepresented Gene Ontology molecular functions in genes repressed (A) and promoted (B) in response to high temperature**

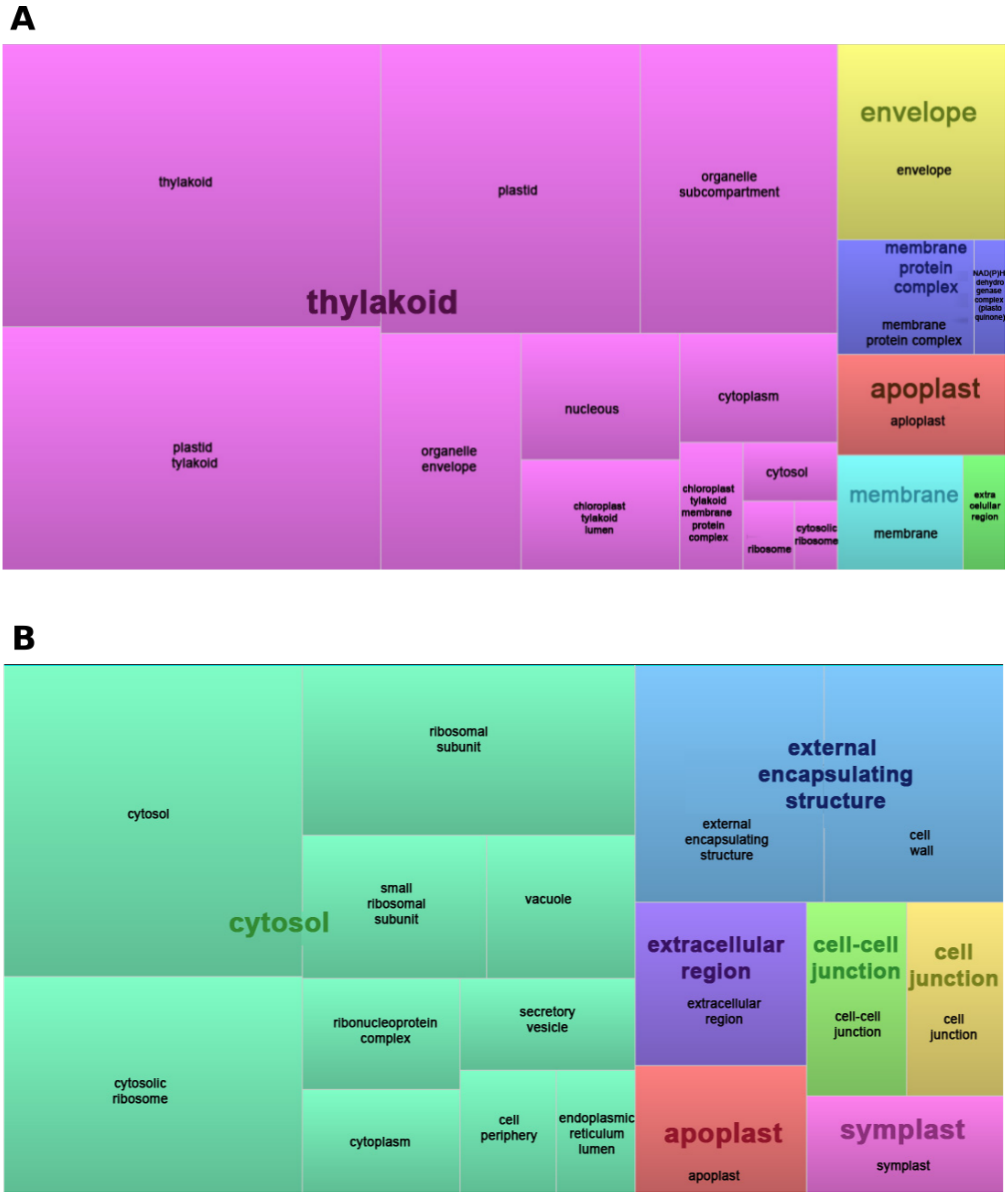

Figure S5: **Semantically reduced overrepresented Gene Ontology cellular components in genes repressed (A) and promoted (B) in response to high temperature**

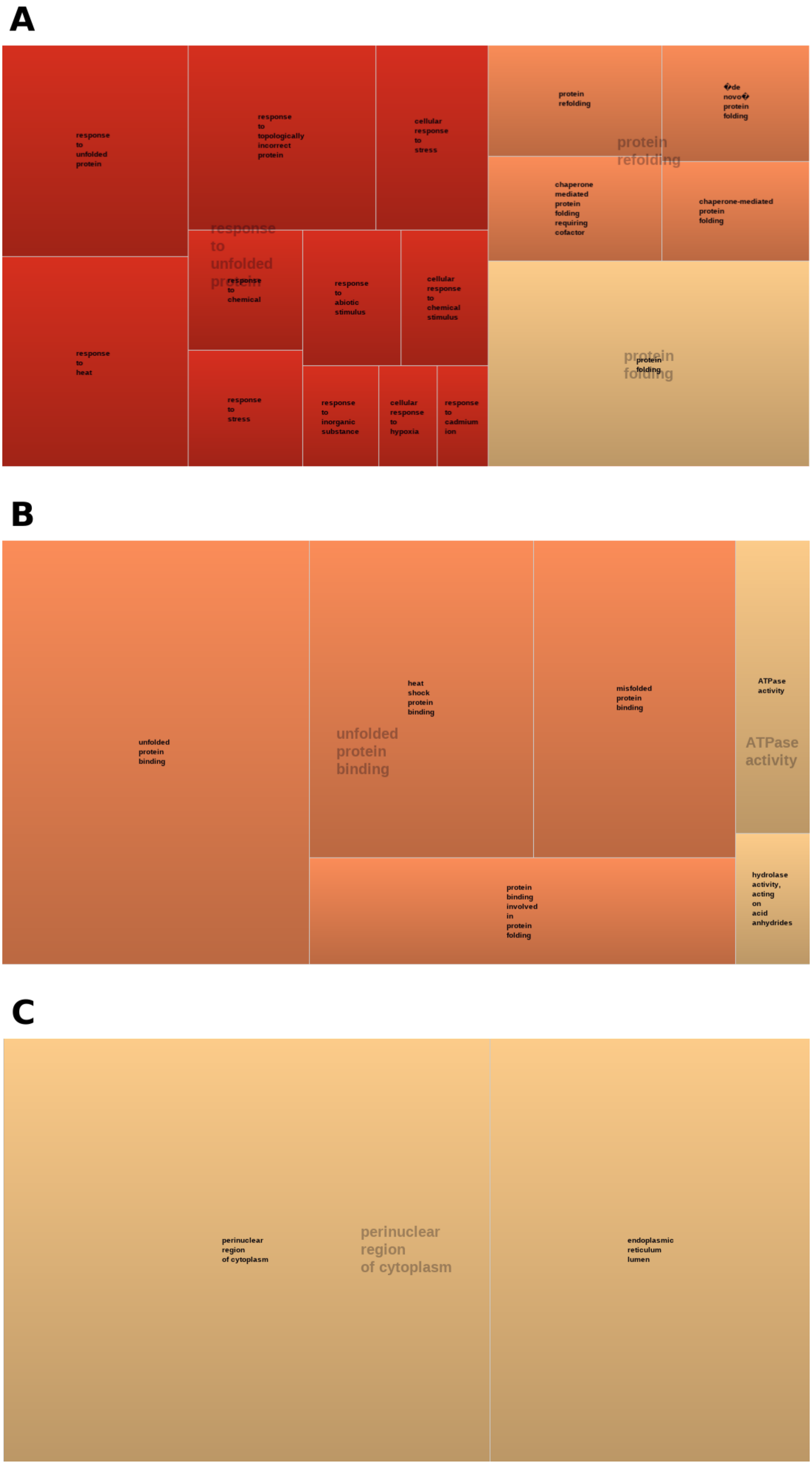

Figure S6: **Semantically reduced overrepresented Gene Ontology biological processes (A), molecular functions (B), and cellular components (C) in genes promoted by high temperature in *N. pumilio*, *A. thaliana* and *P. tomentosa***

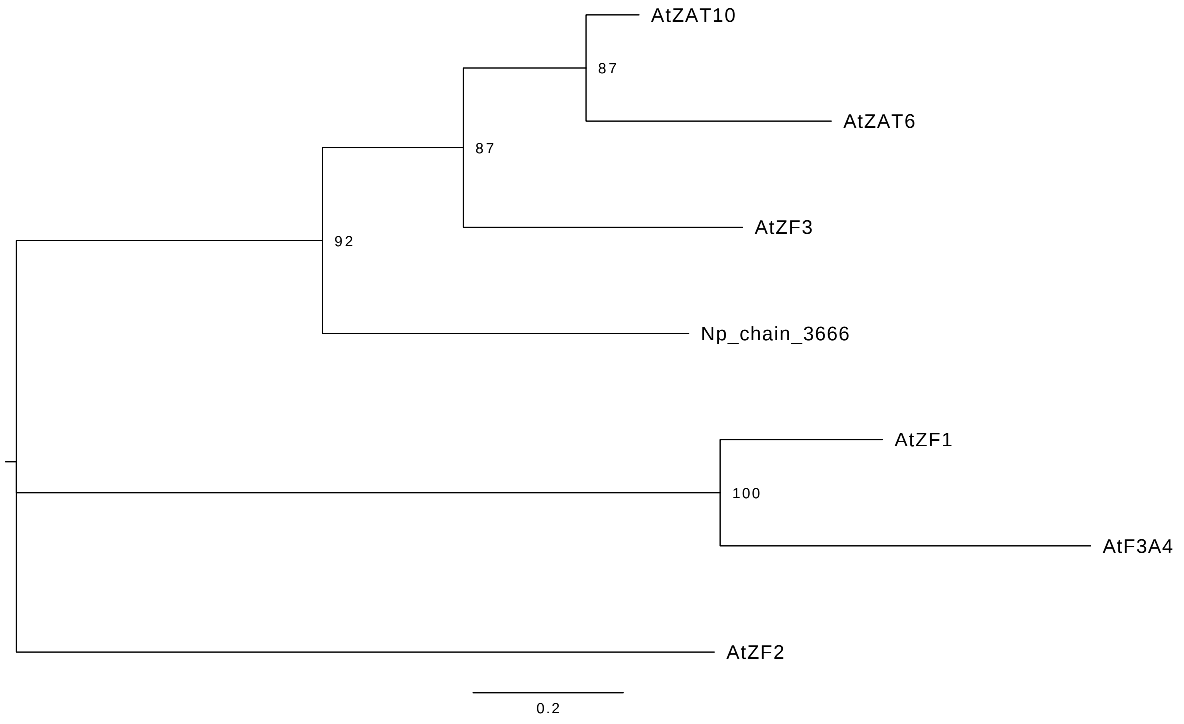

(a) **ZAT10**

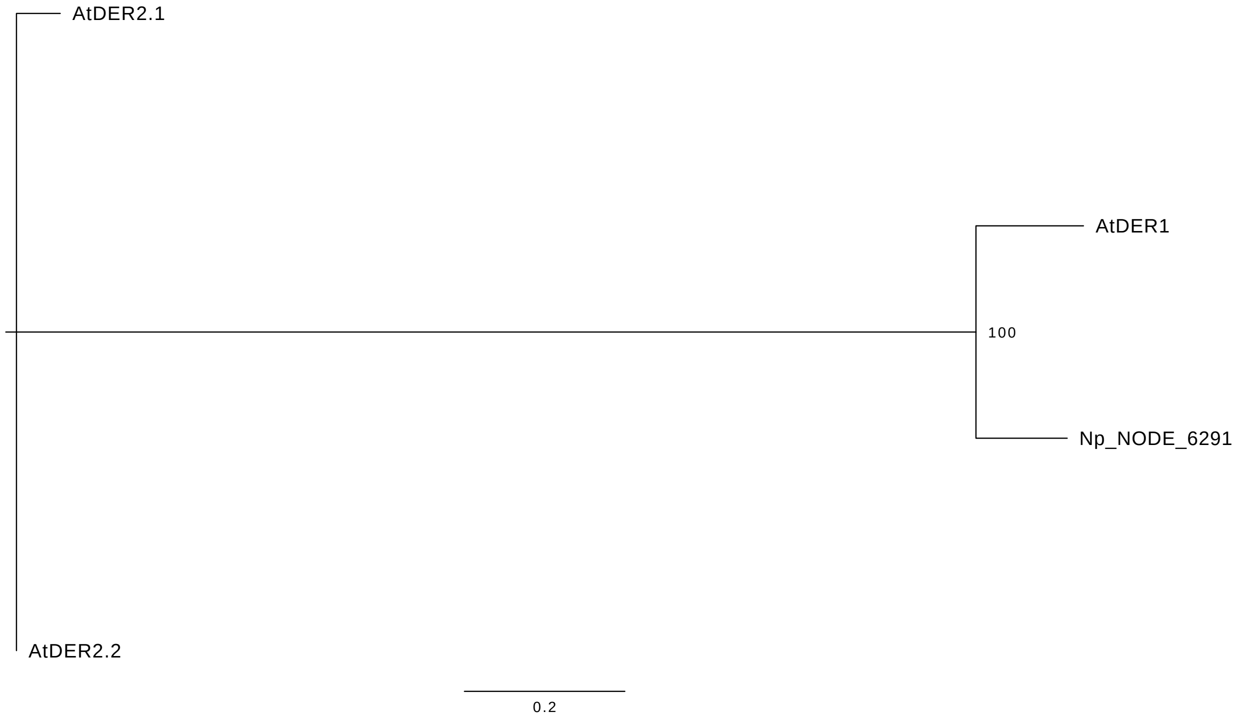

(b) **DER1**

Figure S7: **Maximum Likelihood phylogenetic trees for genes of interest.** Bootstrap support is shown in each bipartition.

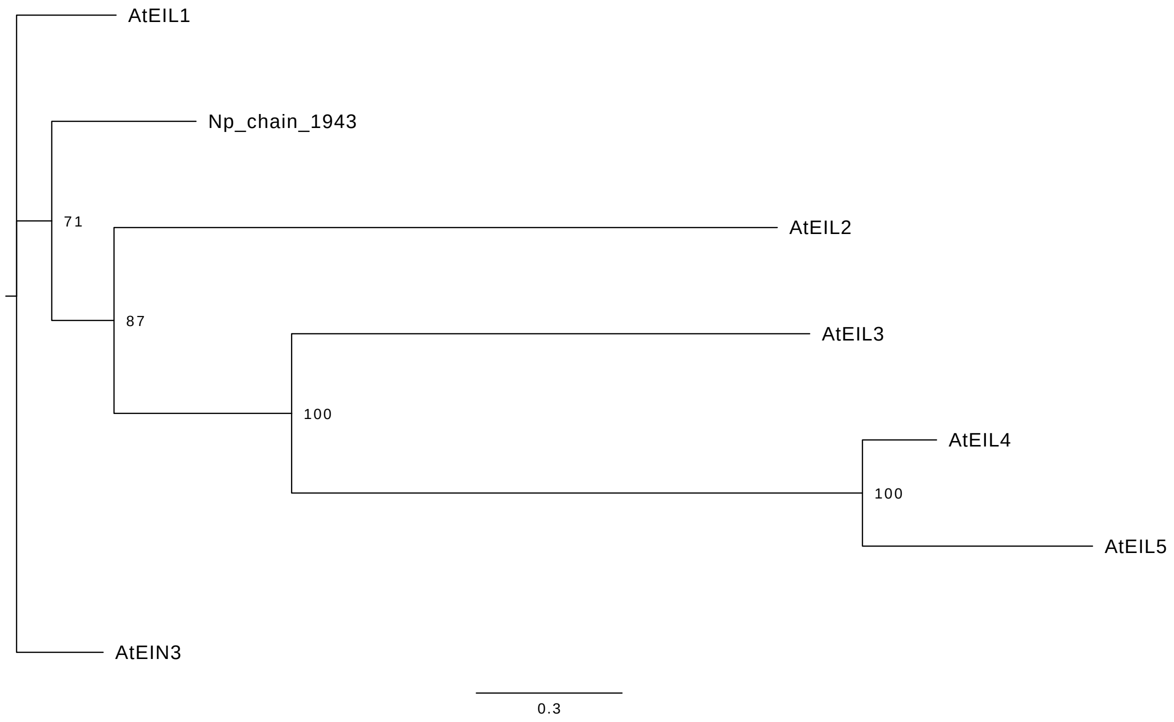

(c) **EIN3**

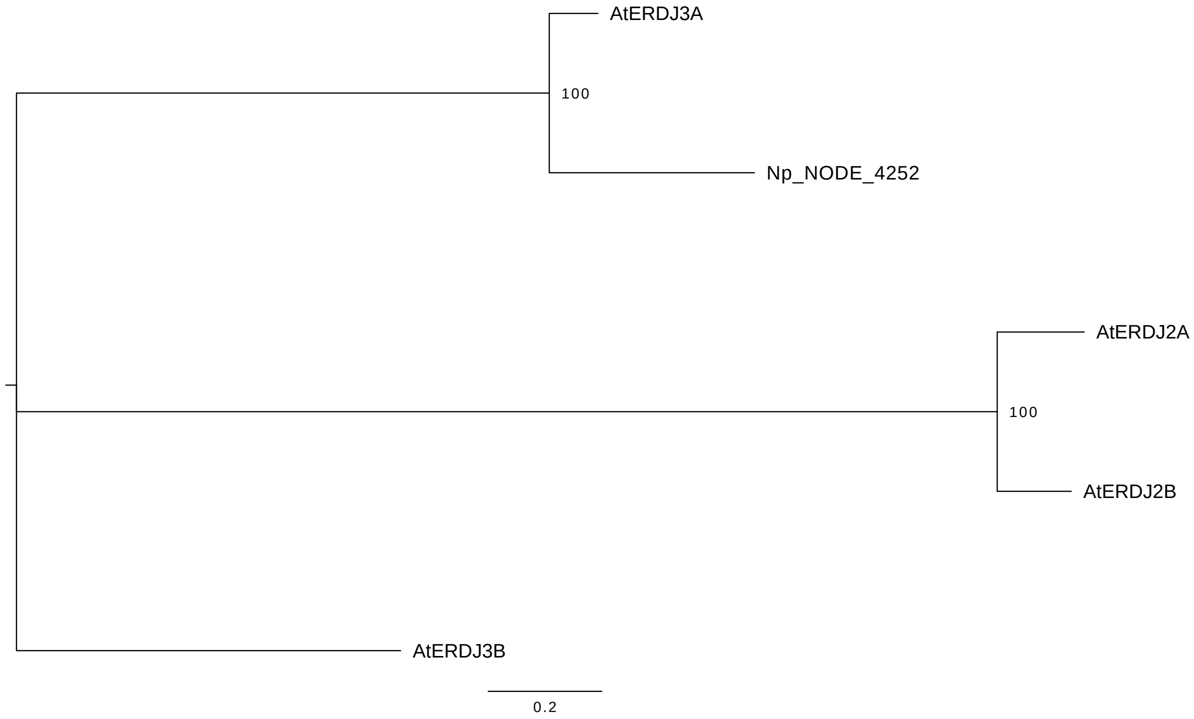

(d) **ERDJ3A**

Figure S7: **Maximum Likelihood phylogenetic trees for genes of interest** (cont.). Bootstrap support is shown in each bipartition.

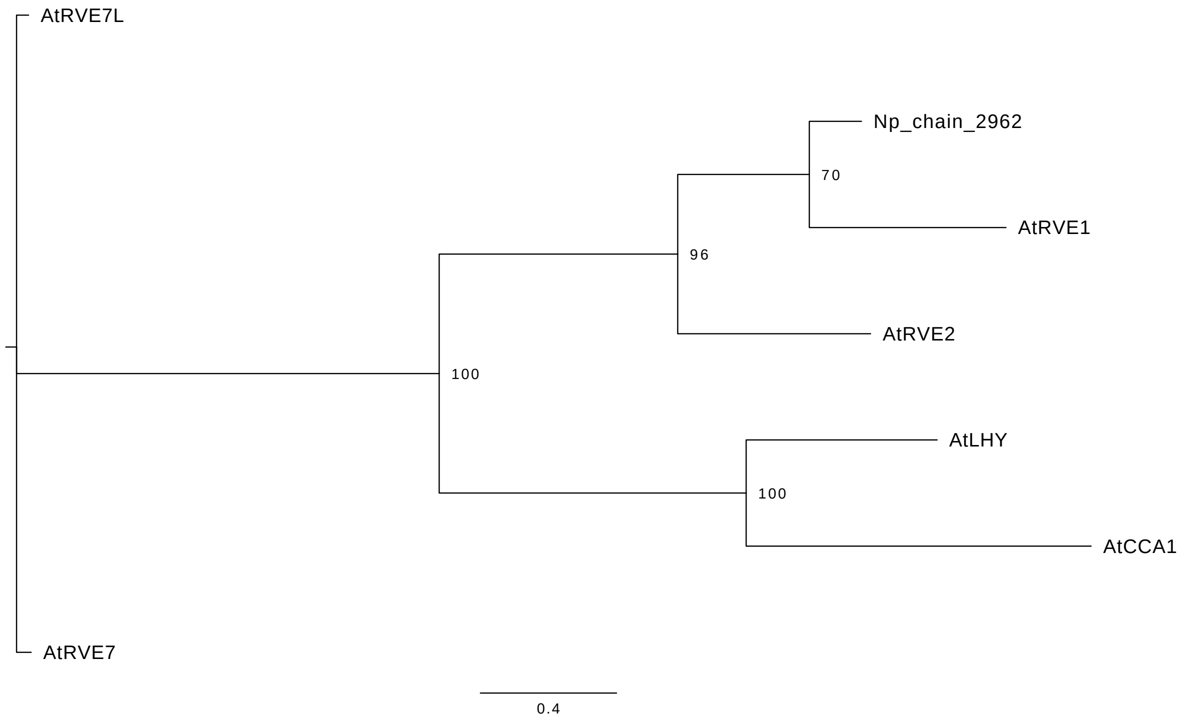

(e) **RVE1**

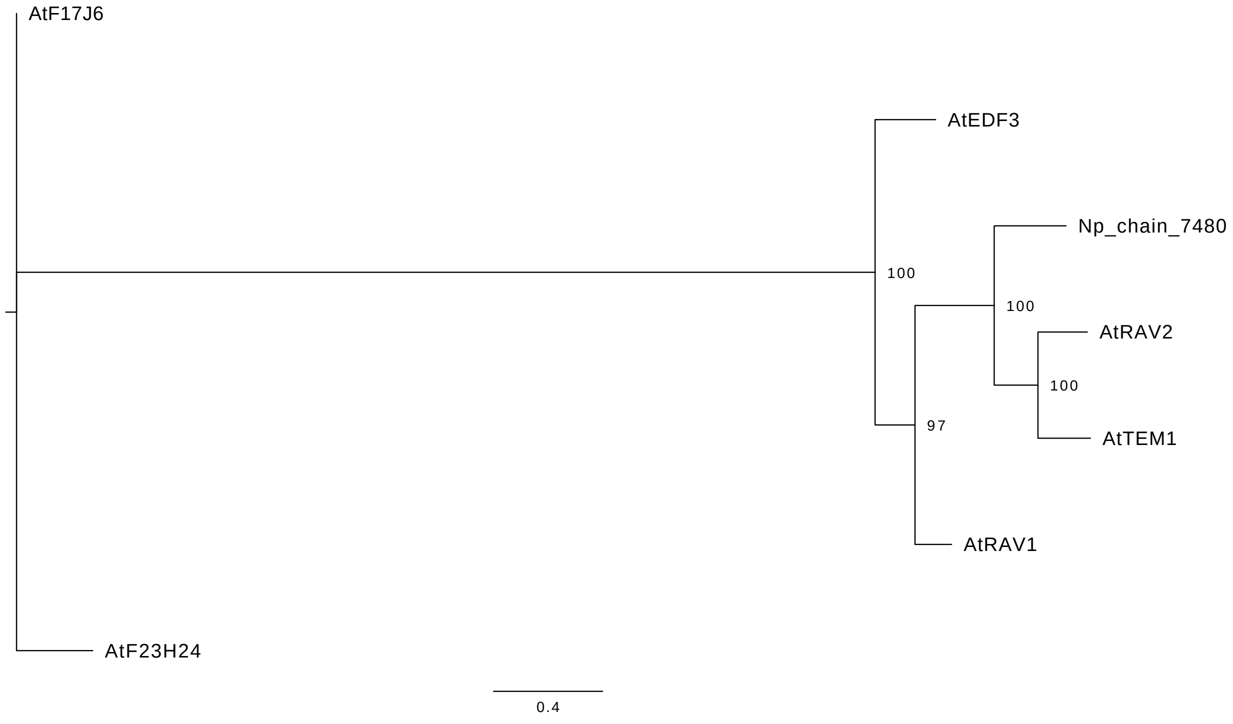

(f) **RAV1**

Figure S7: **Maximum Likelihood phylogenetic trees for genes of interest** (cont.). Bootstrap support is shown in each bipartition.

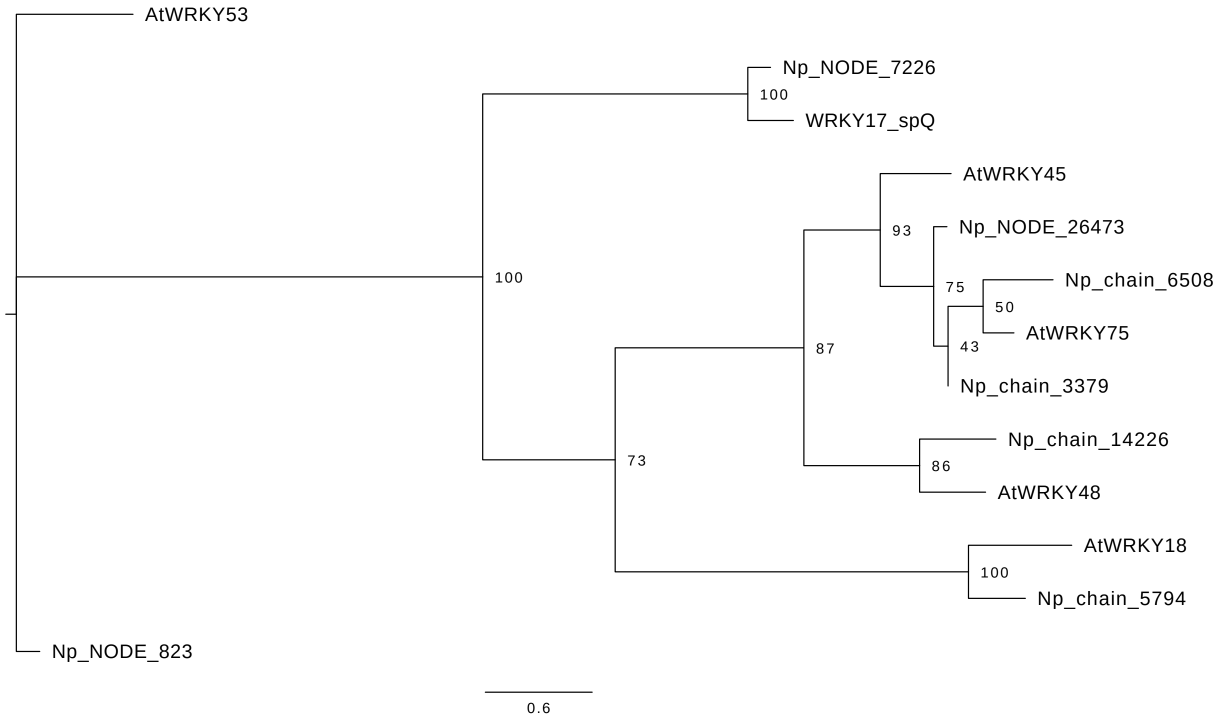

(g) **WRKYs**

Figure S7: **Maximum Likelihood phylogenetic trees for genes of interest** (cont.). Bootstrap support is shown in each bipartition.
