# Supplementary material for: Deciphering the transcriptomic regulation of heat stress responses in Nothofagus pumilio
Source: PLoS One. 2021 Mar 30;16(3):e0246615. doi: 10.1371/journal.pone.0246615 (PMC8009359; doi:10.1371/journal.pone.0246615)
Supplement: S2 File — (PDF) [file pone.0246615.s002.pdf]

List of Tables

S1 Primer sequences for qRT-PCR validation . . . 2
S2 Comparison between assembled NGS and Sanger *Nothofagus pumilio* sequences . . 2
S3 *Nothofagus pumilio* transcriptome deposition information . . . 3
S4 Overrepresented pathways in genes repressed at 34°C . . . 3
S5 Overrepresented pathways in genes promoted at 34°C . . . 4
S6 Overrepresented GO terms in genes repressed at 34°C . . . 5
S7 Overrepresented GO terms in genes promoted at 34°C . . . 6
S8 Transcription factor families annotated in *N. pumilio* transcriptome . . . 8
S9 Transcription factors whose targets are enriched in genes promoted at 34°C . . . 10
S10 Genes promoted by high temperature in *N. pumilio*, *A. thaliana* and *P. tomentosa* 13
S11 Overrepresented GO terms in genes promoted by high temperature in *N. pumilio*, *A. thaliana* and *P. tomentosa* . . . 15
S12 Transcription factors whose targets are enriched in genes promoted by high temperature in *N. pumilio*, *A. thaliana* and *P. tomentosa* . . . 16
S13 Annotated contigs repressed at 34°C in *Nothofagus pumilio* . . . 17
S14 Annotated contigs promoted at 34°C in *Nothofagus pumilio* . . . 71

Table S1: **Primer sequences for qRT-PCR validation**

Gene name	Gene symbol	Strand	Sequence
Sedoheptulose-1,7-bisphosphatase	*SBP*	F	5’ AGGACAGCTTCTTGTGGAGC 3’
		R	5’ GTTGGCAAGCATATCCACGG 3’
Photosystem II reaction center W protein	*PSBW*	F	5’ TGGCTCTGGTGGATGAGAGA 3’
		R	5’ CACCCAACAGGATCCAACCA 3’
Cytochrome P450 90A1	*CYP90A1*	F	5’ TCGGGCTGTACATTTGGACC 3’
		R	5’ CCCGAGTTGTTCTGCCATCT 3’
NAD(P)H-quinone oxido-reductase subunit N	*ndhN*	F	5’ CAGTTTGCCCTGCCCATCTA 3’
		R	5’ GCAGTGCAGAGAGCCTGTAA 3’
DEAD-box ATP-dependent RNA helicase 3	*RH3*	F	5’ TTGAGTTTGTCAGTCCACCGAA 3’
		R	5’ AGAGTAGCAACCACGTGCTCA 3’
E4 SUMO-protein ligase PIAL2	*PIAL2*	F	5’ TTCTCCTGAATGGAAGGGCA 3’
		R	5’ TGTTGGTATCTGTGGTCCTGT 3’
bHLH DNA-binding superfamily protein	*MUL3.10*	F	5’ CATCGGCGGCATCGAAGAA 3’
		R	5’ GGGGACCACTGCTCTAAGTG 3’
23.6 kDa heat shock protein	*HSP23.6*	F	5’ AACAAGGAGCTTGAGCCAGG 3’
		R	5’ CTCGAGACGCTGCGACAAAT 3’
15.7 kDa heat shock protein	*HSP15.7*	F	5’ GCTTGAGTCCCCTAATGCCC 3’
		R	5’ AGGCTCCTCTTTCCCACCTT 3’
bHLH120 transcription factor	*bHLH120*	F	5’ ACAACGAAGGCAAGAAATAGCG 3’
		R	5’ AATTCGCAGCCTCGTTCATGT 3’
MYB108 transcription factor	*MYB108*	F	5’ CCAGGAAGAACCGACAACGA 3’
		R	5’ GCTCAACCATCCTTGGCATC 3’
Heat stress transcription factor A-1d	*HSFA1D*	F	5’ TGTGTCATGGAATGCCGAGG 3’
		R	5’GGCAACAAGAGGTCGGAGAA 3’
Ethylene-responsive transcription factor ERF113	*ERF113*	F	5’ GAGACCATGGGGGAAATGGG 3’
		R	5’GCCTCAGCTGTGTCGAAAGT 3’
Derlin-2.2	*DER2.2*	F	5’ TGCTTGGGTGGATCTACTGG 3’
		R	5’ AATCTCACATTTGCAGGCCG 3’
Probable protein phosphatase 2C 22	*P2C22*	F	5’ GAGCCAAAATGCTGTGGATT 3’
		R	5’ AGCTTTGCAACTCCCTCAAA 3’

Table S2: **Comparison between assembled NGS and Sanger *Nothofagus pumilio* sequences**

Gene	Aligned contig(s)	Alignment length (nt)	% Similarity	GenBank ID	Reference
CDSP32	chain_4856<br>chain_11445	240	99.17	MF446370	Ann. For. Sci. **2020**, *77*, 4
Cum	chain_7193	462	98.48	MF446382	
LEA2	chain_8713	715	99.30	MF446379	
SOS2	chain_70783	451	98.67	MF446388	
rbcL	NODE_2948	1345	99.03	NFGCPRBCLS	Aust. Syst. Bot **1993**, *6*, 441

Table S3: ***Nothofagus pumilio* transcriptome deposition information**

NCBI BioProject	PRJNA414196			
NCBI Study	SRP133997			
NCBI BioSample	SAMN07782031	SAMN07782033	SAMN07782035	SAMN07782037
	SAMN07782032	SAMN07782034	SAMN07782036	SAMN07782038
SRA run	SRR6808716	SRR6808714	SRR6808721	SRR6808719
	SRR6808715	SRR6808717	SRR6808720	SRR6808718
TSA Accession	GHFL00000000			

Table S4: **Overrepresented pathways in genes repressed at 34°C**

KEGG Pathway	Corrected p-value
Photosynthesis	4.70×10^{-24}
Metabolic pathways	2.54×10^{-19}
Carbon fixation in photosynthetic organisms	1.46×10^{-14}
Carbon metabolism	9.52×10^{-13}
Photosynthesis - antenna proteins	1.57×10^{-12}
Glyoxylate and dicarboxylate metabolism	1.22×10^{-11}
Biosynthesis of secondary metabolites	5.19×10^{-9}
Glycine, serine and threonine metabolism	1.24×10^{-5}
Pentose phosphate pathway	1.65×10^{-5}
Alanine, aspartate and glutamate metabolism	1.45×10^{-4}
Carotenoid biosynthesis	1.79×10^{-4}
Nitrogen metabolism	2.42×10^{-4}
Biosynthesis of amino acids	8.09×10^{-4}
Glycolysis / Gluconeogenesis	9.74×10^{-4}
Fructose and mannose metabolism	8.14×10^{-3}
Arginine biosynthesis	1.19×10^{-2}
Ascorbate and aldarate metabolism	1.28×10^{-2}
Ribosome	1.38×10^{-2}
Linoleic acid metabolism	1.48×10^{-2}
Glutathione metabolism	1.98×10^{-2}
ABC transporters	2.24×10^{-2}
Porphyrin and chlorophyll metabolism	3.20×10^{-2}
Taurine and hypotaurine metabolism	4.23×10^{-2}

Table S5: **Overrepresented pathways in genes promoted at 34°C**

KEGG Pathway	Corrected p-value
Metabolic pathways	3.00×10^{-27}
Biosynthesis of secondary metabolites	4.74×10^{-20}
Carbon metabolism	3.73×10^{-11}
Ribosome	1.70×10^{-10}
Protein processing in endoplasmic reticulum	1.15×10^{-9}
Biosynthesis of amino acids	3.88×10^{-8}
Glycolysis / Gluconeogenesis	2.06×10^{-6}
Carbon fixation in photosynthetic organisms	1.64×10^{-5}
Glyoxylate and dicarboxylate metabolism	2.67×10^{-5}
Flavonoid biosynthesis	4.67×10^{-5}
Valine, leucine and isoleucine degradation	1.08×10^{-4}
Citrate cycle (TCA cycle)	2.03×10^{-4}
Glutathione metabolism	2.10×10^{-4}
Cysteine and methionine metabolism	2.31×10^{-4}
Phenylpropanoid biosynthesis	2.38×10^{-4}
Alanine, aspartate and glutamate metabolism	5.91×10^{-4}
Beta-alanine metabolism	6.54×10^{-4}
Galactose metabolism	1.26×10^{-3}
Starch and sucrose metabolism	1.96×10^{-3}
Fructose and mannose metabolism	1.96×10^{-3}
Proteasome	3.42×10^{-3}
Phenylalanine metabolism	4.84×10^{-3}
Arginine and proline metabolism	5.69×10^{-3}
2-Oxocarboxylic acid metabolism	5.91×10^{-3}
Arachidonic acid metabolism	7.35×10^{-3}
MAPK signaling pathway - plant	7.35×10^{-3}
Nitrogen metabolism	8.93×10^{-3}
Propanoate metabolism	1.24×10^{-2}
Amino sugar and nucleotide sugar metabolism	1.48×10^{-2}
Tropane, piperidine and pyridine alkaloid biosynthesis	2.35×10^{-2}
Phagosome	2.46×10^{-2}
Spliceosome	2.46×10^{-2}
Monoterpenoid biosynthesis	2.46×10^{-2}
Oxidative phosphorylation	3.03×10^{-2}
RNA transport	3.24×10^{-2}
Pentose phosphate pathway	3.24×10^{-2}
Selenocompound metabolism	3.24×10^{-2}
Sesquiterpenoid and triterpenoid biosynthesis	3.24×10^{-2}
RNA degradation	3.45×10^{-2}
Pyruvate metabolism	3.61×10^{-2}

Table S6: **Overrepresented GO terms in genes repressed at 34°C**

Term	p-value
**Biological process**	
photosynthesis, light harvesting in photosystem I	7.40×10^{-7}
photosynthesis, light harvesting	3.87×10^{-7}
generation of precursor metabolites and energy	1.17×10^{-15}
photosynthesis, light reaction	4.61×10^{-20}
photosynthesis	3.62×10^{-25}
photosynthetic electron transport in photosystem I	6.24×10^{-3}
photosynthetic electron transport chain	1.57×10^{-6}
electron transport chain	1.72×10^{-3}
oxidation-reduction process	6.16×10^{-4}
protein-chromophore linkage	1.07×10^{-4}
carbon fixation	4.45×10^{-2}
gluconeogenesis	1.53×10^{-2}
glucose metabolic process	1.47×10^{-3}
hexose metabolic process	3.27×10^{-2}
monosaccharide metabolic process	1.92×10^{-2}
hexose biosynthetic process	3.98×10^{-2}
monosaccharide biosynthetic process	4.06×10^{-2}
regulation of photosynthesis, light reaction	2.13×10^{-2}
regulation of photosynthesis	4.06×10^{-2}
regulation of generation of precursor metabolites and energy	3.51×10^{-3}
photosystem II assembly	2.13×10^{-2}
response to high light intensity	2.59×10^{-3}
response to light intensity	9.82×10^{-3}
response to light stimulus	3.33×10^{-7}
response to radiation	1.14×10^{-6}
response to abiotic stimulus	2.96×10^{-4}
response to stimulus	6.51×10^{-4}
response to cytokinin	1.90×10^{-2}
**Molecular function**	
chlorophyll binding	7.68×10^{-6}
tetrapyrrole binding	1.18×10^{-5}
cofactor binding	6.87×10^{-3}
oxidoreductase activity	1.11×10^{-3}
**Cellular component**	
chloroplast photosystem II	1.37×10^{-6}
chloroplast thylakoid membrane protein complex	2.45×10^{-11}
thylakoid part	3.85×10^{-44}
thylakoid	3.89×10^{-53}
membrane protein complex	1.43×10^{-6}
membrane part	6.30×10^{-3}
membrane	4.82×10^{-7}
chloroplast thylakoid membrane	2.42×10^{-43}
chloroplast thylakoid	1.39×10^{-45}

continued on the next page

Repressed GO terms (*continued*)

Term	p-value
chloroplast part	2.36×10^{-53}
chloroplast	3.86×10^{-34}
plastid	4.13×10^{-34}
cytoplasmic part	5.92×10^{-14}
cytoplasm	5.06×10^{-10}
plastid part	1.14×10^{-52}
intracellular organelle part	8.27×10^{-11}
organelle part	8.58×10^{-11}
plastid thylakoid	1.75×10^{-45}
organelle subcompartment	4.84×10^{-30}
plastid thylakoid membrane	3.12×10^{-43}
thylakoid membrane	7.98×10^{-42}
photosynthetic membrane	1.02×10^{-41}
photosystem II	2.42×10^{-12}
photosystem	7.55×10^{-23}
photosystem I reaction center	4.46×10^{-3}
photosystem I	4.34×10^{-13}
NAD(P)H dehydrogenase complex (plastoquinone)	2.02×10^{-2}
photosystem II oxygen evolving complex	6.88×10^{-5}
light-harvesting complex	8.49×10^{-6}
plastoglobule	4.20×10^{-12}
chloroplast stroma	6.31×10^{-31}
plastid stroma	1.54×10^{-30}
stromule	1.99×10^{-4}
plastid envelope	4.95×10^{-27}
organelle envelope	1.50×10^{-15}
envelope	1.50×10^{-15}
chloroplast thylakoid lumen	4.10×10^{-9}
plastid thylakoid lumen	4.10×10^{-9}
thylakoid lumen	1.39×10^{-11}
apoplast	1.25×10^{-7}
chloroplast membrane	6.28×10^{-3}
chloroplast envelope	1.84×10^{-26}
plastid membrane	1.24×10^{-2}
ribosome	2.84×10^{-2}
nuclear part	2.33×10^{-2}
nucleus	1.07×10^{-18}

Table S7: **Overrepresented GO terms in genes promoted at 34°C**

Term	p-value
**Biological process**	
cellular response to unfolded protein	1.15×10^{-2}
response to unfolded protein	1.15×10^{-2}
response to topologically incorrect protein	1.42×10^{-2}

continued on the next page

Promoted GO terms (*continued*)

Term	p-value
response to stress	7.33×10^{-9}
response to stimulus	7.30×10^{-10}
response to organic substance	8.58×10^{-5}
response to chemical	1.67×10^{-12}
cellular response to topologically incorrect protein	2.28×10^{-2}
protein refolding	3.96×10^{-2}
protein folding	2.24×10^{-3}
response to cadmium ion	1.02×10^{-7}
response to metal ion	1.82×10^{-7}
response to inorganic substance	9.17×10^{-9}
translation	9.96×10^{-7}
cellular protein metabolic process	4.16×10^{-2}
metabolic process	2.24×10^{-2}
organonitrogen compound metabolic process	2.99×10^{-6}
organic substance biosynthetic process	1.85×10^{-3}
biosynthetic process	7.34×10^{-3}
peptide biosynthetic process	1.32×10^{-6}
peptide metabolic process	1.78×10^{-7}
cellular amide metabolic process	2.23×10^{-7}
organonitrogen compound biosynthetic process	4.65×10^{-6}
amide biosynthetic process	1.82×10^{-6}
cellular nitrogen compound biosynthetic process	8.52×10^{-3}
response to oxidative stress	2.74×10^{-3}
response to drug	1.32×10^{-3}
response to salt stress	9.03×10^{-3}
response to osmotic stress	8.59×10^{-3}
response to abiotic stimulus	5.63×10^{-5}
response to other organism	7.92×10^{-5}
response to external biotic stimulus	7.92×10^{-5}
response to external stimulus	2.22×10^{-4}
response to biotic stimulus	8.12×10^{-5}
multi-organism process	1.40×10^{-4}
oxidation-reduction process	4.35×10^{-4}
defense response	1.91×10^{-3}
response to oxygen-containing compound	8.80×10^{-4}
catabolic process	1.98×10^{-2}
DNA metabolic process	1.37×10^{-2}
nucleic acid metabolic process	1.98×10^{-3}
**Molecular function**	
misfolded protein binding	2.51×10^{-2}
unfolded protein binding	2.38×10^{-3}
structural constituent of ribosome	7.14×10^{-9}
structural molecule activity	3.34×10^{-8}
oxidoreductase activity	2.16×10^{-5}
catalytic activity	6.41×10^{-3}

continued on the next page

Promoted GO terms (*continued*)

Term	p-value
**Cellular component**	
endoplasmic reticulum lumen	1.24×10^{-2}
cytoplasmic part	1.26×10^{-2}
cytoplasm	2.09×10^{-2}
cytosolic small ribosomal subunit	2.47×10^{-6}
small ribosomal subunit	5.89×10^{-5}
ribosomal subunit	8.93×10^{-10}
ribonucleoprotein complex	1.71×10^{-2}
ribosome	1.56×10^{-7}
cytosolic ribosome	1.95×10^{-11}
cytosolic part	1.76×10^{-12}
cytosol	4.18×10^{-12}
cytosolic large ribosomal subunit	2.92×10^{-5}
large ribosomal subunit	1.89×10^{-3}
apoplast	1.78×10^{-7}
extracellular region	1.19×10^{-5}
cell wall	4.60×10^{-9}
external encapsulating structure	2.49×10^{-9}
cell periphery	1.05×10^{-2}
plasmodesma	1.75×10^{-4}
symplast	1.75×10^{-4}
cell-cell junction	1.75×10^{-4}
cell junction	1.75×10^{-4}
vacuole	7.43×10^{-5}
integral component of membrane	3.80×10^{-2}

Table S8: **Transcription factor families annotated in *N. pumilio* transcriptome**. An asterisk indicates an enrichment ratio larger than 2, i.e. more than twice TFs observed than expected for the corresponding temperature

Family	Members promoted at 34°C	Members repressed at 20°C	Total annotated members
MYB	8	2	63
bHLH	5	2	56
ERF	11*	1	43
NAC	3	0	38
bZIP	2	1	36
C2H2	3	1	35
WRKY	6*	0	35
MYB_like	2	3*	30
C3H	2	1	29
HD-ZIP	3	0	25
Trihelix	3	0	22
GRAS	0	0	21

continued on the next page

Transcription factor families (*continued*)

Family	Members promoted at 34°C	Members repressed at 20°C	Total annotated members
G2-like	1	1	18
FAR1	1	1	17
HSF	2	0	15
ARF	1	2*	15
GATA	0	0	14
Dof	0	0	13
CO-like	0	1*	11
TCP	0	1*	11
LBD	2*	0	9
ZF-HD	0	0	9
AP2	1	0	8
TALE	0	0	8
SBP	0	0	8
MIKC_MADS	0	0	8
HB-other	1	0	7
ARR-B	0	0	7
B3	0	0	7
Nin-like	0	0	7
DBB	0	1*	6
E2F/DP	0	0	6
CAMTA	0	1*	5
NF-YB	0	0	5
NF-YC	0	0	4
BBR-BPC	0	0	4
BES1	0	0	4
NF-YA	0	0	4
GRF	0	0	4
WOX	1*	0	3
LSD	0	0	3
GeBP	0	0	3
SRS	0	0	3
M-type_MADS	0	0	3
CPP	0	0	3
EIL	1*	0	2
RAV	0	1*	2
S1Fa-like	0	0	2
NF-X1	0	0	2
HB-PHD	0	0	2
STAT	0	0	2
HRT-like	0	0	2
YABBY	0	0	2
Whirly	0	0	1

continued on the next page

Transcription factor families (*continued*)

Family	Members promoted at 34°C	Members repressed at 20°C	Total annotated members
VOZ	0	0	1

Table S9: **Transcription factors whose targets are enriched in genes promoted at 34°C**

Name	UniProt ID	Enrichment p-value
Protein NLP4	Q9LE38	2.811×10^{-27}
Protein ASYMMETRIC LEAVES 2	O04479	3.631×10^{-23}
Trihelix transcription factor GTL2	Q8H181	1.493×10^{-21}
GLABROUS1 enhancer-binding protein	Q9ASZ1	5.785×10^{-20}
Ethylene-responsive transcription factor ERF122	Q38Q40	5.121×10^{-19}
Calmodulin-binding transcription activator 3	Q8GSA7	3.570×10^{-14}
Transcription factor E2FB	Q9FV71	1.127×10^{-13}
Ethylene-responsive transcription factor RAP2-4	Q8H1E4	2.748×10^{-12}
Protein LATERAL ORGAN BOUNDARIES	Q9FML4	2.188×10^{-11}
Ethylene-responsive transcription factor ERF018	Q9S7L5	3.732×10^{-11}
NAC (No Apical Meristem) domain transcriptional regulator superfamily protein	Q9LSI4	7.873×10^{-11}
Probable WRKY transcription factor 63	Q9C6H5	5.390×10^{-10}
B3 domain-containing transcription factor FUS3	Q9LW31	1.043×10^{-9}
Ethylene-responsive transcription factor ERF019	O80542	5.402×10^{-9}
Dehydration-responsive element-binding protein 2C	Q8LFR2	5.974×10^{-9}
NAC domain-containing protein 62	Q9SCK6	8.615×10^{-9}
GATA transcription factor 16	Q9FJ10	9.355×10^{-8}
Ethylene-responsive transcription factor ERF013	Q9CAP4	1.181×10^{-7}
Transcription factor MYB83	Q9C6U1	9.480×10^{-7}
Protein NLP7	Q84TH9	1.031×10^{-6}
Ethylene-responsive transcription factor ABR1	Q9FGF8	1.495×10^{-6}
Transcription factor bHLH78	Q9FJL4	1.582×10^{-6}
Transcription factor ABA-INDUCIBLE bHLH-TYPE	Q9ZPY8	2.550×10^{-6}
Ethylene-responsive transcription factor RAP2-12	Q9SSA8	3.534×10^{-6}
Ethylene-responsive transcription factor ERF003	Q94AW5	4.933×10^{-6}
Ethylene-responsive transcription factor 9	Q9FE67	5.891×10^{-6}
Transcription factor MYB63	Q6R0A6	6.784×10^{-6}
Ethylene-responsive transcription factor ERF115	Q9LY29	9.144×10^{-6}
Transcription factor MYB56	Q6R053	1.472×10^{-5}
Ethylene-responsive transcription factor ERF015	Q6NLD5	1.482×10^{-5}
Ethylene-responsive transcription factor 3	O80339	1.739×10^{-5}
At2g23280	O22179	3.283×10^{-5}
Ethylene-responsive transcription factor RAP2-6	Q7G1L2	3.322×10^{-5}
Ethylene-responsive transcription factor ERF112	P93007	4.976×10^{-5}
Ethylene-responsive transcription factor 5	O80341	6.789×10^{-5}
Ethylene-responsive transcription factor 1B	Q8LDC8	8.541×10^{-5}
Transcription factor RAX3	Q9M2Y9	9.981×10^{-5}

continued on the next page

Transcription factors (*continued*)

Name	UniProt ID	Enrichment p-value
Zinc finger protein ZAT2	Q9SIJ0	1.409×10^{-4}
Transcription factor MYB58	Q9SA47	1.501×10^{-4}
Putative NAC domain-containing protein 94	Q9FIW5	1.576×10^{-4}
Transcription factor TCP9	O64647	1.617×10^{-4}
Transcription factor MYB15	Q9LTC4	1.755×10^{-4}
Transcription factor MYB51	O49782	1.808×10^{-4}
LOB domain-containing protein 13	Q9AT61	2.140×10^{-4}
Cell division cycle 5-like protein	P92948	2.337×10^{-4}
B3 domain-containing protein REM1	Q84J39	2.337×10^{-4}
Trihelix transcription factor ASIL2	Q9LJG8	2.398×10^{-4}
Ethylene-responsive transcription factor SHINE 3	Q3E958	2.440×10^{-4}
GATA transcription factor 28	Q8H1G0	2.779×10^{-4}
Transcription factor MYB52	Q6R0C4	3.475×10^{-4}
E2F transcription factor-like E2FD	Q9LFQ9	3.630×10^{-4}
GATA transcription factor 1	Q8LAU9	3.931×10^{-4}
Ethylene-responsive transcription factor 11	Q9C5I3	3.994×10^{-4}
Sequence-specific DNA binding transcription factor	Q8GXR6	4.366×10^{-4}
Ethylene-responsive transcription factor LEP	Q9M644	5.215×10^{-4}
Ethylene-responsive transcription factor ABI4	A0MES8	5.994×10^{-4}
Protein BASIC PENTACYSTEINE1	Q9SKD0	6.806×10^{-4}
Auxin response factor 8	Q9FGV1	7.466×10^{-4}
At1g74650	Q9CA52	1.026×10^{-3}
Homeobox-leucine zipper protein HAT22	P46604	1.133×10^{-3}
Transcription factor MYB44	Q9FDW1	1.570×10^{-3}
Protein SENSITIVE TO PROTON RHIZOTOXICITY 1	Q9C8N5	1.748×10^{-3}
Homeobox-leucine zipper protein ATHB-12	Q9M276	1.782×10^{-3}
Ethylene-responsive transcription factor ERF025	Q9FJ90	1.861×10^{-3}
Transcription factor MYB105	Q9SEZ4	1.966×10^{-3}
GATA transcription factor 15	Q8LG10	2.167×10^{-3}
Transcription factor MYB111	Q9FJ07	2.308×10^{-3}
Zinc finger protein ZAT7	Q42453	2.436×10^{-3}
Ethylene-responsive transcription factor ERF021	Q9C9I2	2.616×10^{-3}
At5g05790	Q9FFJ9	2.805×10^{-3}
Homeobox-leucine zipper protein HAT1	P46600	2.987×10^{-3}
Auxin response factor 2	Q94JM3	3.150×10^{-3}
Transcription factor bHLH74	Q6NKN9	3.248×10^{-3}
Transcription factor MYB13	Q9LNC9	3.268×10^{-3}
Transcription factor JUNGBRUNNEN 1	Q9SK55	3.323×10^{-3}
Ethylene-responsive transcription factor ERF027	Q38Q39	4.264×10^{-3}
Ethylene-responsive transcription factor ERF057	Q9FJQ2	4.321×10^{-3}
Transcription factor MYB12	O22264	4.643×10^{-3}
Dehydration-responsive element-binding protein 2F	Q9SVX5	4.651×10^{-3}
B3 domain-containing protein At5g18090	Q9FK61	5.081×10^{-3}
Protein SHI RELATED SEQUENCE 1	Q9SD40	5.413×10^{-3}

continued on the next page

Transcription factors (*continued*)

Name	UniProt ID	Enrichment p-value
Homeobox-leucine zipper protein ATHB-9	O04292	5.983×10^{-3}
Dehydration-responsive element-binding protein 1A	Q9M0L0	6.178×10^{-3}
Transcription factor VIP1	Q9MA75	7.517×10^{-3}
Ethylene-responsive transcription factor ERF012	Q9SFE4	7.667×10^{-3}
Zinc finger protein ZAT10	Q96289	7.745×10^{-3}
Transcription factor HBI1	Q9ZPW3	8.025×10^{-3}
B3 domain-containing transcription factor ABI3	Q01593	8.876×10^{-3}
Transcription factor MYB96	Q24JK1	9.078×10^{-3}
LOB domain-containing protein 16	Q9SLB7	9.483×10^{-3}
At1g49010	Q9M9A3	1.006×10^{-2}
GATA transcription factor 24	Q8GXL7	1.174×10^{-2}
Ethylene-responsive transcription factor ERF054	Q9M0J3	1.209×10^{-2}
Homeobox-leucine zipper protein ATHB-15	Q9ZU11	1.259×10^{-2}
Zinc finger protein WIP5	Q8W031	1.268×10^{-2}
Sequence-specific DNA binding transcription factor	F4K0Q7	1.320×10^{-2}
Dehydration-responsive element-binding protein 1B	P93835	1.325×10^{-2}
Ethylene-responsive transcription factor RAP2-11	Q6J9S1	1.487×10^{-2}
Transcription factor MYB10	Q9LTV4	1.502×10^{-2}
Transcription factor MYB17	Q9M2D9	1.509×10^{-2}
Ethylene-responsive transcription factor 7	Q9LDE4	1.550×10^{-2}
Ethylene-responsive transcription factor ERF096	Q9LSX0	1.590×10^{-2}
B3 domain-containing transcription factor NGA4	O82595	1.593×10^{-2}
NAC domain-containing protein 68	A8MQY1	1.622×10^{-2}
Ethylene-responsive transcription factor RAP2-10	Q9SW63	1.647×10^{-2}
LOB domain-containing protein 23	P59467	2.111×10^{-2}
Ethylene-responsive transcription factor ERF073	Q8H0T5	2.149×10^{-2}
Ethylene-responsive transcription factor ERF017	Q84QC2	2.172×10^{-2}
Transcription factor SRM1	Q9FNN6	2.220×10^{-2}
WRKY transcription factor 18	Q9C5T4	2.320×10^{-2}
Ethylene-responsive transcription factor RAP2-7	Q9SK03	2.365×10^{-2}
Dehydration-responsive element-binding protein 2B	O82133	2.389×10^{-2}
At1g15720	Q9LMR2	2.446×10^{-2}
Transcription factor HRS1	Q9FX67	2.542×10^{-2}
Transcription factor IIIA	Q84MZ4	2.578×10^{-2}
Transcription factor MYB46	Q9LXV2	2.610×10^{-2}
bZIP transcription factor 18	O22873	2.695×10^{-2}
Transcription factor MYB77	Q9SN12	2.728×10^{-2}
Transcription factor MYB61	Q8VZQ2	2.779×10^{-2}
Dehydration-responsive element-binding protein 1D	Q9FJ93	2.914×10^{-2}
AT3g12720/MBK21_8	Q9LTW2	3.014×10^{-2}
Transcription factor MYB41	Q9M0J5	3.051×10^{-2}
Transcription factor HHO6	Q9FX84	3.068×10^{-2}
MYB transcription factor	Q9LRU5	3.300×10^{-2}
Uridine monophosphate kinase	Q94JR1	3.628×10^{-2}

continued on the next page

Transcription factors (*continued*)

Name	UniProt ID	Enrichment p-value
Dehydration-responsive element-binding protein 2G	P61827	3.787×10^{-2}
C2H2-like zinc finger protein	Q9FFA9	4.166×10^{-2}
BES1-interacting Myc-like protein 3	A0A1P8BB12	4.189×10^{-2}
Transcription factor BIM3	Q9FMB6	4.189×10^{-2}
LOB domain-containing protein 2	Q9LNB9	4.220×10^{-2}
Transcription factor MYB3	Q9S9K9	4.269×10^{-2}
Probable WRKY transcription factor 38	Q8GWF1	4.343×10^{-2}
Nuclear transcription factor Y subunit B-1	Q9SLG0	4.512×10^{-2}
NAC domain-containing protein 73	O49459	4.598×10^{-2}
Ethylene-responsive transcription factor ERF094	Q9LND1	4.863×10^{-2}
ABSCISIC ACID-INSENSITIVE 5-like protein 4	Q9M7Q5	4.979×10^{-2}
GATA transcription factor 9	O82632	4.995×10^{-2}

Table S10: **Genes promoted by high temperature in *N. pumilio*, *A. thaliana* and *P. tomentosa***

Name	UniProt ID
15.7 kDa heat shock protein, peroxisomal	Q9FHQ3
17.6 kDa class I heat shock protein 1	Q9XIE3
18.1 kDa class I heat shock protein	P19037
23.6 kDa heat shock protein, mitochondrial	Q96331
3-hydroxy-3-methylglutaryl-coenzyme A reductase 1	P14891
60S acidic ribosomal protein P1-3	Q8LEQ0
Aha1 domain-containing protein	Q9LHL7
Alpha/beta-Hydrolases superfamily protein	F4HXL0
Amine oxidase	Q8L866
At1g27330	Q84K46
AT5g58110/k21l19_90	Q9FGT3
Calreticulin-2	Q38858
Chaperone protein ClpB1	P42730
Chaperone protein dnaJ 3	Q94AW8
COBRA-like protein 8	Q9LIB6
DBH-like monooxygenase	O65233
DEAD-box ATP-dependent RNA helicase 10	Q8GY84
DEAD-box ATP-dependent RNA helicase 21	P93008
DEAD-box ATP-dependent RNA helicase 40	Q9SQV1
Derlin-1	Q8VZU9
DnaJ protein ERDJ3A	Q9SR96
DnaJ protein P58IPK homolog	Q9LYW9
Endoplasmic reticulum oxidoreductin-1	Q9C7S7
Endoplasmin homolog	Q9STX5
Eukaryotic aspartyl protease family protein	Q9M8R6
F-box protein PP2-B11	Q949S5
Fe^{2+} transport protein 3, chloroplastic	Q8LE59
Glucose-6-phosphate/phosphate translocator 2, chloroplastic	Q94B38

continued on the next page

Common promoted genes (*continued*)

Name	UniProt ID
Heat shock 70 kDa protein 10, mitochondrial	Q9LDZ0
Heat shock 70 kDa protein 5	Q9S9N1
Heat shock 70 kDa protein 6, chloroplastic	Q9STW6
Heat shock 70 kDa protein 8	Q9SKY8
Heat shock 70 kDa protein 9, mitochondrial	Q8GUM2
Heat shock protein 90-1	P27323
Heat shock protein 90-2	P55737
Heat shock protein 90-6, mitochondrial	F4JFN3
Heat stress transcription factor B-2b	Q9T0D3
Inosine-5'-monophosphate dehydrogenase 1	P47996
Inosine-5'-monophosphate dehydrogenase 2	Q9SA34
Kinesin-like protein KIN-UB	Q9LPC6
Late embryogenis abundant protein 41	Q39084
Leucine-rich repeat receptor-like protein kinase PEPR1	Q9SSL9
Mediator of RNA polymerase II transcription subunit 37a	Q9LKR3
Microfibril-associated protein-like	Q9FKN6
Mitochondrial import inner membrane translocase subunit TIM23-3	Q9S837
Multiprotein-bridging factor 1c	Q9LV58
O-fucosyltransferase 28	Q9M393
Pectinesterase inhibitor 10	Q9SI74
PHD finger-like domain-containing protein 5B	Q0WMV8
Probable aquaporin PIP2-5	Q9SV31
Probable carboxylesterase 4, mitochondrial	Q9FX93
Probable galactinol–sucrose galactosyltransferase 2	Q94A08
Probable galactinol–sucrose galactosyltransferase 6	Q8RX87
Probable inactive poly [ADP-ribose] polymerase SRO2	Q9ZUD9
Probable mediator of RNA polymerase II transcription subunit 37c	P22954
Probable mediator of RNA polymerase II transcription subunit 37c	Q9LHA8
Probable nucleoredoxin 1	O80763
Probable plastid-lipid-associated protein 1, chloroplastic	O81439
Probable WRKY transcription factor 48	Q9FGZ4
Probable xyloglucan endotransglucosylase/hydrolase protein 23	Q38910
Pyridoxal 5'-phosphate synthase-like subunit PDX1.2	Q9ZNR6
T-complex protein 1 subunit eta	Q9SF16
Translationally controlled tumor protein 2	Q9M9V9
Trihelix transcription factor ASIL2	Q9LJG8
Trihelix transcription factor GT-3b	O80450
Ubiquitin carboxyl-terminal hydrolase 9	Q93Y01
Zinc finger CCCH domain-containing protein 22	Q9SK49
Zinc finger protein ZAT10	Q96289

Table S11: **Overrepresented GO terms in genes promoted by high temperature in *N. pumilio*, *A. thaliana* and *P. tomentosa***

Term	p-value
**Biological process**	
response to unfolded protein	1.76×10^{-12}
cellular response to unfolded protein	5.11×10^{-11}
protein refolding	1.78×10^{-6}
cellular response to topologically incorrect protein	9.02×10^{-10}
response to topologically incorrect protein	6.49×10^{-11}
chaperone cofactor-dependent protein refolding	5.12×10^{-6}
de novo posttranslational protein folding	5.12×10^{-6}
de novo protein folding	7.50×10^{-6}
endoplasmic reticulum unfolded protein response	2.67×10^{-2}
protein folding	4.87×10^{-20}
chaperone-mediated protein folding	5.09×10^{-5}
response to heat	2.75×10^{-12}
cellular response to hypoxia	1.83×10^{-2}
cellular response to oxygen levels	2.04×10^{-2}
cellular response to decreased oxygen levels	2.04×10^{-2}
response to temperature stimulus	3.35×10^{-11}
response to hypoxia	4.72×10^{-2}
response to cadmium ion	3.11×10^{-2}
cellular response to stress	8.10×10^{-7}
cellular response to chemical stimulus	3.95×10^{-4}
response to inorganic substance	5.58×10^{-3}
response to abiotic stimulus	1.36×10^{-4}
response to organic substance	9.85×10^{-3}
response to chemical	1.03×10^{-4}
response to stress	1.22×10^{-4}
**Molecular function**	
misfolded protein binding	4.53×10^{-10}
protein folding chaperone	2.28×10^{-7}
heat shock protein binding	4.03×10^{-11}
unfolded protein binding	6.12×10^{-20}
ATPase activity	6.51×10^{-4}
nucleoside-triphosphatase activity	1.74×10^{-2}
pyrophosphatase activity	3.31×10^{-2}
hydrolase activity, acting on acid anhydrides	3.78×10^{-2}
hydrolase activity, acting on acid anhydrides, in phosphorus-containing anhydrides	3.57×10^{-2}
**Cellular component**	
endoplasmic reticulum lumen	1.35×10^{-2}
perinuclear region of cytoplasm	1.49×10^{-3}

Table S12: **Transcription factors whose targets are enriched in genes promoted by high temperature in *N. pumilio*, *A. thaliana* and *P. tomentosa***

Name	UniProt ID	Enrichment p-value
GATA transcription factor 15	Q8LG10	8.225×10^{-5}
Protein NLP4	Q9LE38	1.145×10^{-4}
GLABROUS1 enhancer-binding protein	Q9ASZ1	2.013×10^{-4}
GATA transcription factor 16	Q9FJ10	2.518×10^{-4}
Ethylene-responsive transcription factor ERF021	Q9C9I2	2.866×10^{-4}
Sequence-specific DNA binding transcription factor	Q8GXR6	4.514×10^{-4}
Probable WRKY transcription factor 60	Q9SK33	4.570×10^{-4}
GATA transcription factor 1	Q8LAU9	6.780×10^{-4}
Probable WRKY transcription factor 62	Q9LZV6	9.302×10^{-4}
Ethylene-responsive transcription factor 5	O80341	1.492×10^{-3}
Transcription factor MYB10	Q9LTV4	2.106×10^{-3}
Ethylene-responsive transcription factor LEP	Q9M644	2.160×10^{-3}
Ethylene-responsive transcription factor 9	Q9FE67	2.404×10^{-3}
Ethylene-responsive transcription factor SHINE 3	Q3E958	2.586×10^{-3}
Transcription factor MYB13	Q9LNC9	2.650×10^{-3}
Zinc finger protein ZAT10	Q96289	2.714×10^{-3}
GATA transcription factor 24	Q8GXL7	2.787×10^{-3}
Trihelix transcription factor ASIL2	Q9LJG8	3.483×10^{-3}
NAC domain-containing protein 96	Q9LS24	3.659×10^{-3}
LOB domain-containing protein 18	O22131	5.245×10^{-3}
Zinc finger protein AZF1	Q9SSW1	5.884×10^{-3}
Sequence-specific DNA binding transcription factor	F4K0Q7	6.256×10^{-3}
GATA transcription factor 28	Q8H1G0	6.376×10^{-3}
Transcription factor MYB83	Q9C6U1	7.421×10^{-3}
Transcription factor MYB15	Q9LTC4	9.146×10^{-3}
Ethylene-responsive transcription factor ERF057	Q9FJQ2	9.392×10^{-3}
Ethylene-responsive transcription factor ERF038	Q9ZQP3	1.018×10^{-2}
Ethylene-responsive transcription factor ERF003	Q94AW5	1.266×10^{-2}
Protein LATERAL ORGAN BOUNDARIES	Q9FML4	1.470×10^{-2}
Ethylene-responsive transcription factor ERF027	Q38Q39	1.595×10^{-2}
Ethylene-responsive transcription factor ERF013	Q9CAP4	1.623×10^{-2}
Ethylene-responsive transcription factor ERF015	Q6NLD5	1.835×10^{-2}
Ethylene-responsive transcription factor ERF010	Q9FH94	1.977×10^{-2}
Ethylene-responsive transcription factor ERF039	Q9SUK8	2.086×10^{-2}
Ethylene-responsive transcription factor 7	Q9LDE4	2.089×10^{-2}
Ethylene-responsive transcription factor ERF011	Q9SNE1	2.510×10^{-2}
Ethylene-responsive transcription factor ERF012	Q9SFE4	3.015×10^{-2}
Ethylene-responsive transcription factor RAP2-11	Q6J9S1	3.723×10^{-2}
Ethylene-responsive transcription factor RAP2-12	Q9SSA8	4.070×10^{-2}
Dehydration-responsive element-binding protein 2G	P61827	4.099×10^{-2}
Ethylene-responsive transcription factor ERF014	Q9LPE8	4.176×10^{-2}
Ethylene-responsive transcription factor ERF112	P93007	4.217×10^{-2}

continued on the next page

Transcription factors (*continued*)

Name	UniProt ID	Enrichment p-value
Ethylene-responsive transcription factor RAP2-6	Q7G1L2	4.359×10^{-2}
Ethylene-responsive transcription factor ABR1	Q9FGF8	4.359×10^{-2}
Dehydration-responsive element-binding protein 2D	Q9LQZ2	4.456×10^{-2}
Ethylene-responsive transcription factor ERF105	Q8VY90	4.511×10^{-2}
Ethylene-responsive transcription factor ERF087	Q9FZ90	4.563×10^{-2}
Ethylene-responsive transcription factor ERF054	Q9M0J3	4.623×10^{-2}
Ethylene-responsive transcription factor 3	O80339	4.727×10^{-2}
Ethylene-responsive transcription factor 15	Q8VYM0	4.754×10^{-2}
Ethylene-responsive transcription factor ERF115	Q9LY29	4.776×10^{-2}
Ethylene-responsive transcription factor ERF086	Q6J9Q2	4.802×10^{-2}

Table S13: **Annotated contigs repressed at 34°C in *Nothofagus pumilio*. Target coverage:** Percentage of *A. thaliana* protein covered by the alignment with *N. pumilio* query. **Alignment identity:** Percentage of matches relative to the length of the *N. pumilio* – *A. thaliana* alignment

Name	UniProt ID	Contig	$\log_2$ (fold-Change)	Target coverage (%)	Alignment identity (%)
E3 ubiquitin ligase BIG BROTHER-related	Q9LT17	chain_2178	-25.0072	76.08	79.7
Probable carotenoid cleavage dioxygenase 4, chloroplastic	O49675	chain_54149	-15.593	90.2	79.58
Thiamine thiazole synthase, chloroplastic	Q38814	chain_50095	-11.4771	94.23	93.84
Phosphoribulokinase, chloroplastic	P25697	chain_46733	-11.3968	88.03	92.74
Cytochrome P450 90A1	Q42569	NODE_16792	-10.8883	86.89	85.71
Ribulose bisphosphate carboxylase/oxygenase activase, chloroplastic	P10896	chain_10305	-10.5486	89.77	94.27
Photosystem II reaction center W protein, chloroplastic	Q39194	chain_55506	-10.2589	65.38	73.13
Protein ECERIFERUM 1	F4HVY0	chain_12442	-10.0486	88.0	70.48
Ribulose bisphosphate carboxylase/oxygenase activase, chloroplastic	P10896	chain_21296	-9.5219	70.97	86.05
Germin-like protein subfamily 3 member 3	P94072	chain_5407	-9.4402	95.95	71.52
Photosystem I reaction center subunit II-2, chloroplastic	Q9SA56	chain_31418	-9.0376	69.62	88.89
Glutamine synthetase, chloroplastic/mitochondrial	Q43127	chain_21209	-8.9393	74.07	89.47
Ribulose bisphosphate carboxylase/oxygenase activase, chloroplastic	P10896	chain_26023	-8.9259	60.53	90.91
GDSL esterase/lipase At4g28780	Q9SVU5	chain_36713	-8.5101	71.64	77.89

continued on the next page

Contigs repressed at 34°C (*continued*)

Name	UniProt ID	Contig	$\log_2$ (fold-Change)	Target coverage (%)	Alignment identity (%)
Fructose-bisphosphate aldolase 3, chloroplastic	Q9ZU52	chain_61649	-8.165	91.87	73.75
Leucine-rich repeat (LRR) family protein	Q9LT85	chain_26841	-8.13	77.14	64.15
Fructose-bisphosphate aldolase 2, chloroplastic	Q944G9	chain_45617	-8.0614	71.11	88.89
ABC transporter G family member 40	Q9M9E1	chain_5556	-7.9881	27.27	86.96
Ribulose bisphosphate carboxylase small chain 3B, chloroplastic	P10798	chain_2026	-7.9029	72.07	83.54
Protein NRT1/ PTR FAMILY 2.9	Q9M9V7	chain_16667	-7.7984	78.3	60.81
Lipoxygenase 2, chloroplastic	P38418	NODE_5928	-7.703	89.02	75.76
Chlorophyll a-b binding protein 2.4, chloroplastic	Q9XF87	NODE_26321	-7.6825	88.24	76.07
Histone H3-like 3	Q9LR02	chain_33964	-7.6681	92.59	97.96
Photosystem I reaction center subunit XI, chloroplastic	Q9SUI4	chain_17136	-7.6289	88.52	75.47
Cytochrome P450 86A1	P48422	NODE_21040	-7.6235	39.24	64.81
DNA repair protein RAD5A	Q9FNI6	chain_6120	-7.4692	73.51	80.91
At1g50450/F11F12_20	Q94BZ0	chain_2238	-7.4537	100	79.54
Proline-rich protein 4	Q9T0I5	NODE_16001	-7.4205	57.35	61.21
Aspartyl protease AED1	Q9LEW3	chain_69304	-7.3819	79.71	68.75
Ribulose bisphosphate carboxylase small chain 3B, chloroplastic	P10798	chain_44223	-7.3638	55.26	85.37
Histone H4	P59259	NODE_24431	-7.2636	84.55	91.26
Cytochrome P450 86A1	P48422	chain_17832	-7.1793	24.22	66.67
Elongation factor 1-alpha 2	Q8W4H7	NODE_28618	-7.1145	77.48	95.29
Glycine dehydrogenase (decarboxylating) 1, mitochondrial	Q94B78	chain_31496	-6.9742	48.08	83.33
Ribosomal protein L23AB	A8MS83	chain_67722	-6.9679	39.62	90.0
31 kDa ribonucleoprotein, chloroplastic	Q04836	chain_54487	-6.9424	46.51	94.74
Leucine-rich repeat transmembrane protein kinase	F4I3K4	chain_35934	-6.9368	91.23	68.63
Ribulose bisphosphate carboxylase/oxygenase activase, chloroplastic	P10896	chain_2700	-6.874	84.76	93.48
Polyubiquitin 9	Q9FHQ6	NODE_28339	-6.8609	100	89.6

continued on the next page

Contigs repressed at 34°C (*continued*)

Name	UniProt ID	Contig	$\log_2$ (fold-Change)	Target coverage (%)	Alignment identity (%)
Probable xyloglucan endotransglucosylase/hydrolase protein 16	Q8LG58	chain_50711	-6.8586	92.75	76.19
60S ribosomal protein L35-1	Q9SF53	chain_41157	-6.8295	90.1	80.0
Chlorophyll a-b binding protein 2.4, chloroplastic	Q9XF87	NODE_47960	-6.8138	85.88	80.28
Superoxide dismutase [Mn] 2, mitochondrial	Q9LYK8	NODE_38702	-6.8036	62.5	66.67
Elongation factor 1-alpha 2	Q8W4H7	chain_16525	-6.7842	58.74	74.42
Golgi apparatus membrane protein-like protein ECHIDNA	Q8LEK2	chain_9654	-6.7771	82.08	84.97
Peptidyl-prolyl cis-trans isomerase CYP18-4	Q42406	NODE_30744	-6.7762	86.52	68.42
Photosystem I reaction center subunit VI-1, chloroplastic	Q9SUI7	chain_3708	-6.7495	53.93	97.87
Ubiquitin-specific protease 13	F4J7I2	chain_64126	-6.6902	96.93	93.02
40S ribosomal protein S8-1	Q93VG5	NODE_25533	-6.6881	35.97	81.63
Xyloglucan galactosyltransferase XLT2	F4K6F1	NODE_18365	-6.6717	63.55	67.19
Glyceraldehyde-3-phosphate dehydrogenase GAPB, chloroplastic	P25857	chain_60098	-6.6652	50.0	95.65
Exostosin family protein	Q9XID1	chain_12005	-6.6642	90.64	80.72
WAT1-related protein At5g64700	Q9FGG3	NODE_11432	-6.6467	38.61	56.67
Photosystem II 10 kDa polypeptide, chloroplastic	P27202	NODE_24931	-6.6313	36.91	66.67
Elongation factor 1-alpha 2	Q8W4H7	chain_16861	-6.5779	80.34	85.07
GDSL esterase/lipase At4g28780	Q9SVU5	chain_32173	-6.5635	70.0	77.03
60S ribosomal protein L18a-3	Q9LUD4	NODE_32049	-6.5586	54.78	70.97
Leucine-rich repeat transmembrane protein kinase	F4I3K4	chain_24355	-6.5466	64.29	66.42
Probable histone H2AXb	Q9S9K7	NODE_58559	-6.5284	60.53	73.33
Aldehyde dehydrogenase family 2 member C4	Q56YU0	NODE_33625	-6.5036	27.27	68.97
(S)-2-hydroxy-acid oxidase GLO2	Q9LRS0	NODE_67681	-6.4831	100	78.13
Metal tolerance protein C4	Q8H1G3	chain_29733	-6.4759	25.0	94.74
Cytochrome P450 94B3	Q9SMP5	NODE_21434	-6.4542	29.75	67.44
Chlorophyll a-b binding protein 2.1, chloroplastic	Q9SHR7	NODE_30426	-6.4387	100	70.69

continued on the next page

Contigs repressed at 34°C (*continued*)

Name	UniProt ID	Contig	$\log_2$ (fold-Change)	Target coverage (%)	Alignment identity (%)
Probable pectate lyase 8	Q9M8Z8	NODE_30596	-6.4016	96.0	82.11
Chlorophyll a-b binding protein 2.1, chloroplastic	Q9SHR7	chain_53865	-6.3646	67.78	67.24
Protein NDH-DEPENDENT CYCLIC ELECTRON FLOW 5	Q9C503	NODE_7941	-6.3637	53.38	61.43
Ribulose bisphosphate carboxylase/oxygenase activase, chloroplastic	P10896	chain_25281	-6.3086	75.44	83.33
Glyceraldehyde-3-phosphate dehydrogenase GAPB, chloroplastic	P25857	chain_61058	-6.2895	36.54	100.0
Probable receptor-like protein kinase At4g10390	Q9SV83	chain_15102	-6.2787	56.84	70.0
S-locus lectin protein kinase family protein	A0A1P8B6Y2	chain_16900	-6.2775	33.06	61.54
Chlorophyll a-b binding protein, chloroplastic	Q39142	chain_66712	-6.259	53.49	81.82
Fructose-bisphosphate aldolase 1, chloroplastic	Q9SJU4	chain_21660	-6.2543	91.3	66.13
Histone superfamily protein	A8MRL0	NODE_26029	-6.2323	70.97	88.37
Organic cation/carnitine transporter 2	O64515	NODE_12485	-6.1636	85.83	68.64
Probable disease resistance protein At1g59620	Q9LQ54	chain_31074	-6.1479	69.64	60.53
Probable carboxylesterase 15	Q9FG13	NODE_35709	-6.1473	54.74	62.75
At4g34790	Q9SW55	chain_23606	-6.1467	91.67	67.44
Fructose-bisphosphate aldolase 6, cytosolic	Q9SJQ9	chain_58342	-6.1435	82.65	85.0
AT4g18560/F28J12_220	Q8L7S5	NODE_31692	-6.0872	62.26	93.75
Tubulin alpha-3 chain	Q56WH1	NODE_45724	-6.0709	62.86	95.24
Ribosomal L29 family protein	F4KDR2	chain_21367	-6.0669	65.85	71.7
Chlorophyll a-b binding protein 3, chloroplastic	Q9S7M0	chain_64196	-6.0406	50.68	90.28
60S ribosomal protein L31-2	Q9STR1	chain_16766	-6.0399	85.29	67.44
Leucine-rich repeat receptor-like serine/threonine-protein kinase At1g17230	Q9SHI2	chain_37884	-6.0161	40.35	81.82
Peptidyl-prolyl cis-trans isomerase CYP19-2	Q9SKQ0	chain_29098	-6.0126	70.86	82.88
Receptor protein kinase CLAVATA1	Q9SYQ8	chain_17004	-5.9936	97.3	63.49
Probable LRR receptor-like serine/threonine-protein kinase At1g34110	C0LGF5	NODE_32542	-5.976	84.07	50.22

continued on the next page

Contigs repressed at 34°C (*continued*)

Name	UniProt ID	Contig	$\log_2$ (fold-Change)	Target coverage (%)	Alignment identity (%)
Serine–glyoxylate aminotransferase	Q56YA5	chain_59147	-5.9726	50.0	89.47
Glycine dehydrogenase (decarboxylating) 1, mitochondrial	Q94B78	NODE_70321	-5.9437	53.49	90.91
Aspartyl protease family protein At5g10770	Q8S9J6	chain_16849	-5.9344	84.91	70.45
Palmitoyl-monogalactosyldiacylglycerol delta-7 desaturase, chloroplastic	Q949X0	NODE_52962	-5.8838	73.58	76.32
Polyadenylate-binding protein RBP47C'	Q9SX80	chain_45323	-5.8565	65.63	90.0
Fructose-1,6-bisphosphatase, cytosolic	Q9MA79	chain_27566	-5.8511	72.0	94.12
40S ribosomal protein S8-2	Q9FIF3	chain_37700	-5.846	42.42	74.07
Ubiquitin-40S ribosomal protein S27a-1	P59271	chain_34236	-5.8279	32.14	92.31
Photosystem I reaction center subunit IV A, chloroplastic	Q9S831	chain_38971	-5.8228	66.67	80.95
Probable aquaporin NIP5-1	Q9SV84	chain_25973	-5.8137	93.06	89.39
Sterol 14-demethylase	Q9SAA9	chain_67902	-5.7854	43.64	86.96
Peroxisomal membrane 22 kDa (Mpv17/PMP22) family protein	Q9LV46	chain_66347	-5.785	58.0	78.57
Histone H4	P59259	NODE_44678	-5.7606	62.5	92.59
Rieske (2Fe-2S) domain-containing protein	Q9C9I7	chain_26705	-5.7392	77.78	83.01
Polyubiquitin 10	Q8H159	NODE_45243	-5.7296	49.06	91.03
Fructose-bisphosphate aldolase 2, chloroplastic	Q944G9	chain_35076	-5.7262	18.24	83.33
40S ribosomal protein S15-2	Q9FY66	chain_24026	-5.7222	90.63	67.86
U11/U12 small nuclear ribonucleoprotein 31 kDa protein	Q9CAE4	NODE_20619	-5.6831	52.86	79.45
Photosystem I chlorophyll a/b-binding protein 6, chloroplastic	Q8LCQ4	chain_59855	-5.6808	74.07	94.74
Transcription repressor OFP13	Q9FMC8	NODE_18166	-5.6768	31.22	65.08
Ribulose bisphosphate carboxylase small chain 3B, chloroplastic	P10798	chain_28578	-5.6745	63.58	78.13
Ribulose bisphosphate carboxylase small chain 3B, chloroplastic	P10798	chain_27840	-5.6463	38.37	78.13
Ankyrin repeat family protein	A0A1P8B631	NODE_15231	-5.5997	41.96	60.87
Dentin sialophosphoprotein-like protein	Q9SD87	chain_14119	-5.5922	19.55	92.0
Phosphoglycolate phosphatase 1B, chloroplastic	P0DKC4	chain_64188	-5.5685	72.41	85.0
Curculin-like (Mannose-binding) lectin family protein	Q8GY81	NODE_2980	-5.5658	59.77	60.45

continued on the next page

Contigs repressed at 34°C (*continued*)

Name	UniProt ID	Contig	$\log_2$ (fold-Change)	Target coverage (%)	Alignment identity (%)
Serine–glyoxylate aminotransferase	Q56YA5	chain_70394	-5.5639	84.38	92.45
Senescence-associated protein 13	Q9ZW18	chain_66080	-5.5618	32.47	75.0
Chaperone protein dnaJ 3	Q94AW8	chain_66652	-5.5599	55.56	79.31
Ribulose bisphosphate carboxylase small chain 3B, chloroplastic	P10798	chain_26675	-5.544	30.23	84.0
Sedoheptulose-1,7-bisphosphatase, chloroplastic	P46283	chain_7447	-5.5407	91.46	91.95
Kinesin-like protein KIN-5D	Q9LZU5	chain_61891	-5.5305	45.21	71.88
Nudix hydrolase 8	Q8L7W2	NODE_6496	-5.5176	58.09	71.65
Cytochrome b6-f complex iron-sulfur subunit, chloroplastic	Q9ZR03	chain_8546	-5.5111	48.53	93.55
AT4g29480/F17A13_300	Q9M0D5	chain_63536	-5.5022	50.0	78.26
ABC transporter B family member 21	Q9M1Q9	NODE_63100	-5.4862	95.95	82.44
At1g62730	Q9SI77	chain_63128	-5.4441	62.22	81.82
Peptidyl-prolyl cis-trans isomerase FKBP16-4, chloroplastic	Q9SR70	chain_55260	-5.4265	80.0	87.23
Fructose-bisphosphate aldolase 6, cytosolic	Q9SJQ9	chain_58341	-5.4244	93.98	85.71
Receptor-like protein kinase FERONIA	Q9SCZ4	chain_17041	-5.4191	51.9	65.0
Glycine dehydrogenase (decarboxylating) 1, mitochondrial	Q94B78	chain_2549	-5.3921	98.11	86.67
Fimbrin-3	Q9FJ70	NODE_25157	-5.3875	88.14	72.55
Auxin response factor 1	Q8L7G0	chain_38372	-5.3728	74.87	74.48
At1g26210	Q67YG7	chain_30383	-5.3688	24.72	80.95
Glucan endo-1,3-beta-glucosidase 8	Q6NKW9	NODE_42128	-5.3656	85.42	65.0
Arabinogalactan protein 20	Q9M373	NODE_23412	-5.359	56.79	86.67
Son of sevenless protein	Q9C794	chain_29101	-5.3518	40.0	90.32
Peptidyl-prolyl cis-trans isomerase CYP40	Q9C566	NODE_63149	-5.3418	63.51	67.39
Calcium-transporting ATPase 10, plasma membrane-type	Q9SZR1	NODE_48000	-5.3365	100.0	79.52
Chlorophyllase-1	O22527	NODE_21993	-5.3328	96.92	67.27
Ammonium transporter 2	Q9M6N7	chain_63262	-5.3216	74.0	69.44
14-3-3-like protein GF14 psi	P42644	chain_50315	-5.3201	30.23	76.0

continued on the next page

Contigs repressed at 34°C (*continued*)

Name	UniProt ID	Contig	$\log_2$ (fold-Change)	Target coverage (%)	Alignment identity (%)
60S ribosomal protein L8-2	Q4PSL7	chain_24214	-5.311	69.07	74.24
WAT1-related protein At4g30420	Q9M0B8	NODE_5315	-5.3035	15.48	52.94
Eukaryotic translation initiation factor 5A-1	Q9XI91	chain_56567	-5.2983	65.0	76.0
Proteinase inhibitor I4, serpin (DUF716)	Q9C893	NODE_25356	-5.2894	89.39	75.56
60S ribosomal protein L10-3	Q93W22	chain_24611	-5.282	24.04	75.0
Abscisic acid receptor PYL5	Q9FLB1	NODE_11997	-5.2718	56.1	68.14
ABC transporter B family member 18	Q9LSJ5	chain_9641	-5.261	98.32	70.18
Protein GFS12	F4JY12	chain_37918	-5.2468	69.86	70.83
Photosystem I reaction center subunit IV B, chloroplastic	Q9S714	chain_24689	-5.2438	52.34	85.45
Protein kinase superfamily protein	F4I5F8	chain_63913	-5.239	88.89	87.23
Calmodulin 2	F4IJ46	NODE_42117	-5.2316	78.79	86.27
Protein disulfide isomerase-like 1-1	Q9XI01	chain_980	-5.2118	82.68	70.75
Receptor-like protein 34	Q9SRL2	chain_22873	-5.1872	72.09	66.67
60S ribosomal protein L30-3	Q9LSA3	chain_21424	-5.1813	73.91	81.67
UDP-glycosyltransferase 72E2	Q9LVR1	NODE_69448	-5.1702	71.67	59.52
Glucan synthase-like 10	A0A178V7F9	chain_433	-5.1598	88.03	66.94
At1g02460	Q9FWX5	chain_17812	-5.1583	81.67	83.33
Elongation factor 1-alpha 2	Q8W4H7	NODE_38379	-5.1514	81.54	82.69
Ribulose bisphosphate carboxylase/oxygenase activase, chloroplastic	P10896	chain_13035	-5.1403	54.74	96.08
Photosystem I subunit O	Q949Q5	chain_59331	-5.1399	64.71	85.71
Leucine-rich repeat protein kinase family protein	F4IB69	chain_45428	-5.1357	100.0	55.17
At2g21530	Q8GWP4	chain_37985	-5.1331	33.77	76.0
Tubulin beta-3 chain	Q9ASR0	chain_37810	-5.1316	61.02	81.69
tRNA wybutosine-synthesizing protein 2/3/4	Q8W4K1	chain_3442	-5.1218	43.71	63.89
40S ribosomal protein S30	P49689	NODE_46351	-5.1197	29.07	75.0

continued on the next page

Contigs repressed at 34°C (*continued*)

Name	UniProt ID	Contig	$\log_2$ (fold-Change)	Target coverage (%)	Alignment identity (%)
Cytochrome P450, family 76, subfamily C, polypeptide 6	Q9LQ25	chain_15452	-5.1056	65.98	50.79
Putative disease resistance protein At1g59780	Q9XIF0	NODE_60110	-5.0932	55.26	60.98
ADP-ribosylation factor A1E	Q9M1P5	chain_66454	-5.0862	100.0	95.83
Plant cysteine oxidase 3	Q1G3U6	chain_35132	-5.08	91.11	74.43
30S ribosomal protein S10, chloroplastic	Q9LK61	chain_43855	-5.0775	33.33	100.0
Receptor-like protein 13	Q9C6A6	chain_55882	-5.0531	87.5	60.98
Tropinone reductase homolog At5g06060	Q9LHT0	chain_58418	-5.0476	42.86	68.57
MDIS1-interacting receptor like kinase 2	Q8VZG8	NODE_21475	-5.0298	97.62	62.5
Glutamine synthetase cytosolic isozyme 1-3	Q9LVI8	chain_32259	-5.0278	71.88	86.36
Phosphoribulokinase, chloroplastic	P25697	chain_24823	-5.0098	85.71	91.57
Glycine dehydrogenase (decarboxylating) 1, mitochondrial	Q94B78	chain_58529	-5.0088	32.35	80.95
G-type lectin S-receptor-like serine/threonine-protein kinase SD2-2	Q39203	chain_9604	-5.0032	11.46	60.71
AT3g14620/MIE1_12	Q9LUD2	NODE_31590	-4.9858	93.02	69.62
Transmembrane protein, putative (DUF247)	Q9SN06	chain_7246	-4.9839	11.42	57.69
F22L4.11 protein	Q9LMM4	chain_59585	-4.9761	78.49	76.47
Chlorophyll a-b binding protein 2.1, chloroplastic	Q9SHR7	chain_46968	-4.9706	41.18	92.59
Germin-like protein subfamily 3 member 3	P94072	NODE_5407	-4.9636	100.0	70.56
Fructose-bisphosphate aldolase 5, cytosolic	O65581	chain_44990	-4.9483	60.71	75.76
Non-specific serine/threonine protein kinase	A0A1P8AWP3	chain_2853	-4.9478	82.03	80.71
Polyubiquitin 8	Q39256	chain_46385	-4.9232	62.75	80.87
ABC transporter G family member 11	Q8RXN0	chain_23760	-4.9219	87.91	64.56
Spermidine hydroxycinnamoyl transferase	O64470	chain_12069	-4.9168	26.08	53.78
Cathepsin B-like protease 3	Q94K85	chain_1238	-4.9066	74.14	76.19
Oxygen-evolving enhancer protein 2-1, chloroplastic	Q42029	chain_45271	-4.9054	50.67	72.97
Protein RADIALIS-like 2	Q9SIJ5	chain_15410	-4.8995	60.0	72.41
ATPase 5, plasma membrane-type	Q9SJB3	chain_58782	-4.8863	38.78	94.44

continued on the next page

Contigs repressed at 34°C (*continued*)

Name	UniProt ID	Contig	$\log_2$ (fold-Change)	Target coverage (%)	Alignment identity (%)
Non-intrinsic ABC protein 9	F4KCB8	chain_5358	-4.8815	92.63	87.71
Probable carotenoid cleavage dioxygenase 4, chloroplastic	O49675	chain_48404	-4.8746	27.14	94.44
Peptidyl-prolyl cis-trans isomerase FKBP17-2, chloroplastic	Q9LDY5	chain_5048	-4.8696	60.07	76.16
Glycine dehydrogenase (decarboxylating) 1, mitochondrial	Q94B78	NODE_33692	-4.8658	76.71	92.73
Fructose-bisphosphate aldolase 6, cytosolic	Q9SJQ9	NODE_17389	-4.8447	92.86	77.38
Leucine-rich repeat transmembrane protein kinase	A0A1P8ASI5	chain_14671	-4.8401	86.92	67.82
Ribulose bisphosphate carboxylase small chain 3B, chloroplastic	P10798	chain_31770	-4.8329	98.33	86.21
Disease resistance family protein / LRR family protein	O64757	chain_27125	-4.8157	55.0	60.0
Oxygen-evolving enhancer protein 1-2, chloroplastic	Q9S841	chain_33731	-4.8116	32.89	75.0
Plant invertase/pectin methylesterase inhibitor superfamily protein	Q9LVA3	NODE_13433	-4.8019	76.61	63.06
Photosystem I chlorophyll a/b-binding protein 2, chloroplastic	Q9SYW8	chain_70944	-4.7982	87.4	90.91
Cationic amino acid transporter 1	Q84MA5	NODE_15630	-4.7951	98.72	67.79
Protein CURVATURE THYLAKOID 1A, chloroplastic	O04616	chain_14250	-4.7898	72.67	88.79
Transcriptional corepressor LEUNIG	Q9FUY2	NODE_26586	-4.7836	88.04	57.5
Plant intracellular Ras-group-related LRR protein 6	O64566	NODE_35119	-4.7825	87.01	67.69
Cysteine-rich receptor-like protein kinase 24	O65483	chain_9839	-4.7808	61.76	56.45
Probable leucine-rich repeat receptor-like protein kinase At1g35710	Q9LP24	NODE_10832	-4.7701	74.74	50.13
Serine/arginine-rich splicing factor RS41	P92966	chain_4948	-4.7611	58.82	78.95
Glycine dehydrogenase (decarboxylating) 1, mitochondrial	Q94B78	NODE_35745	-4.7524	74.19	81.82
Actin-8	Q96293	chain_23316	-4.7439	86.05	77.27
Ribulose bisphosphate carboxylase small chain 3B, chloroplastic	P10798	chain_52023	-4.7396	55.34	87.74
Leucine-rich repeat transmembrane protein kinase	F4I3K4	chain_31639	-4.7375	54.84	72.73
B-box zinc finger protein 25	Q9SID1	chain_8983	-4.7138	68.13	81.48
Translation elongation factor EF1B/ribosomal protein S6 family protein	Q9LSA1	chain_8545	-4.7128	51.25	76.54
Protein EMSY-LIKE 4	Q08A72	chain_1715	-4.712	71.08	71.2

continued on the next page

Contigs repressed at 34°C (*continued*)

Name	UniProt ID	Contig	$\log_2$ (fold-Change)	Target coverage (%)	Alignment identity (%)
Ribulose bisphosphate carboxylase small chain 1A, chloroplastic	P10795	chain_60106	-4.6772	73.51	78.9
Ribulose bisphosphate carboxylase/oxygenase activase, chloroplastic	P10896	NODE_35848	-4.6634	75.56	81.82
Protein MET1, chloroplastic	Q94BS2	chain_53138	-4.6517	60.76	76.6
Cationic amino acid transporter 1	Q84MA5	NODE_31039	-4.639	82.47	78.48
Probable GABA transporter 2	Q8L4X4	NODE_27121	-4.6384	72.13	79.07
AT3G26890 protein	Q949N7	NODE_3171	-4.5953	49.09	74.57
High affinity nitrate transporter 2.4	Q9FJH8	NODE_43526	-4.5863	50.59	66.67
Bifunctional inhibitor/lipid-transfer protein/seed storage 2S albumin superfamily protein	Q9SUX3	NODE_26965	-4.5731	87.85	66.67
At2g24350	Q0WPW8	chain_19470	-4.5654	31.4	73.08
30S ribosomal protein S5, chloroplastic	P93014	chain_2904	-4.562	92.89	83.08
Molybdate transporter 1	Q9SL95	NODE_54029	-4.5537	45.59	73.33
Ferredoxin–NADP reductase, leaf isozyme 1, chloroplastic	Q9FKW6	chain_48889	-4.5492	56.1	95.45
Actin family protein	A0A1P8AXV8	NODE_30934	-4.5487	84.0	78.05
Ferredoxin–NADP reductase, leaf isozyme 1, chloroplastic	Q9FKW6	chain_14446	-4.5299	65.57	87.18
G-type lectin S-receptor-like serine/threonine-protein kinase At4g27290	O81832	chain_14794	-4.5279	65.97	66.13
Probable leucine-rich repeat receptor-like serine/threonine-protein kinase At3g14840	C0LGN2	chain_23934	-4.5149	52.56	60.0
FANTASTIC four-like protein (DUF3049)	F4HX10	NODE_13943	-4.502	22.82	75.93
Receptor-like protein 18	Q9ZUK7	chain_44104	-4.4945	71.58	50.75
U-box domain-containing protein 52	Q9FKG6	chain_5660	-4.4744	82.89	62.0
NADPH-dependent alkenal/one oxidoreductase, chloroplastic	Q9ZUC1	chain_1124	-4.4737	37.7	62.22
Glutamate–glyoxylate aminotransferase 2	Q9S7E9	NODE_57956	-4.4726	96.0	78.26
NAD(P)H-quinone oxidoreductase subunit N, chloroplastic	Q9LVM2	NODE_26597	-4.4695	60.0	93.1
Bidirectional sugar transporter SWEET3	Q6NQN5	chain_14095	-4.4435	85.71	69.77
DHHA1 domain protein	F4HRG2	chain_4189	-4.425	94.85	74.6

continued on the next page

Contigs repressed at 34°C (*continued*)

Name	UniProt ID	Contig	$\log_2$ (fold-Change)	Target coverage (%)	Alignment identity (%)
Heavy metal-associated isoprenylated plant protein 35	Q9C7J6	NODE_49427	-4.4202	87.67	58.73
G-type lectin S-receptor-like serine/threonine-protein kinase At4g27290	O81832	chain_17139	-4.3953	65.0	60.53
Oxygen-evolving enhancer protein 1-1, chloroplastic	P23321	NODE_27296	-4.3938	76.92	84.62
AT5g40850/MHK7_8	Q42606	chain_53299	-4.3933	90.4	84.27
Uncharacterized protein At3g55240	Q9M3C7	NODE_19012	-4.3809	66.67	76.19
GDP-mannose 3,5-epimerase	Q93VR3	chain_4960	-4.3799	52.11	85.71
Ribulose bisphosphate carboxylase/oxygenase activase, chloroplastic	P10896	chain_67223	-4.3722	48.08	91.67
Ribulose bisphosphate carboxylase/oxygenase activase, chloroplastic	P10896	chain_44592	-4.3706	62.82	93.75
Chlorophyll a-b binding protein 4, chloroplastic	P27521	chain_64850	-4.3586	41.89	93.33
Acetamidase/Formamidase family protein	Q9SZE9	chain_17295	-4.3556	45.28	95.65
Ribulose bisphosphate carboxylase small chain	F4KA76	chain_28496	-4.3492	85.71	77.36
Acclimation of photosynthesis to environment	Q2HIR7	chain_2132	-4.3475	79.6	83.67
Chlorophyll a-b binding protein 6, chloroplastic	Q01667	chain_16034	-4.3458	65.88	81.82
60S ribosomal protein L36-2	Q9M352	chain_25584	-4.3444	87.18	93.94
HIPL1 protein	Q9SSG3	NODE_21432	-4.3417	94.2	90.63
Serine hydroxymethyltransferase 1, mitochondrial	Q9SZJ5	chain_63332	-4.3314	96.15	89.19
Serine–glyoxylate aminotransferase	Q56YA5	NODE_46245	-4.3295	48.84	92.68
CCT motif family protein	Q8L602	chain_12424	-4.3288	76.22	72.48
Ribulose bisphosphate carboxylase/oxygenase activase, chloroplastic	P10896	chain_1156	-4.3254	81.58	76.67
Ribulose bisphosphate carboxylase small chain 3B, chloroplastic	P10798	chain_37887	-4.3242	62.86	85.71
ABC transporter B family member 5	Q9SYI3	NODE_57532	-4.3076	49.28	66.67
Putative disease resistance RPP13-like protein 1	Q9LRR4	chain_5815	-4.3049	12.2	62.03
V-type proton ATPase subunit B3	Q8W4E2	chain_3668	-4.2929	94.43	98.11
Cellulose synthase-like protein E1	Q8VZK9	NODE_29896	-4.285	73.98	66.67

continued on the next page

Contigs repressed at 34°C (*continued*)

Name	UniProt ID	Contig	$\log_2$ (fold-Change)	Target coverage (%)	Alignment identity (%)
Wall-associated receptor kinase-like 17	Q9M092	NODE_25137	-4.2785	36.84	85.0
Protein TIC 20-v, chloroplastic	Q9FM67	chain_46849	-4.2716	68.38	81.13
Receptor-like serine/threonine-protein kinase SD1-7	Q39086	chain_6303	-4.2534	76.7	66.94
Cytochrome P450 78A6	Q9ZNR0	chain_6692	-4.2494	94.63	74.94
Actin-like ATPase superfamily protein	Q8LGE0	NODE_58311	-4.2424	61.82	66.67
Phytochrome-associated serine/threonine-protein phosphatase 1	Q9SX52	chain_6024	-4.2366	72.08	96.45
Protein ROOT INITIATION DEFECTIVE 3	Q9M3B4	chain_37197	-4.213	50.0	68.75
Chlorophyll a-b binding protein CP26, chloroplastic	Q9XF89	chain_20939	-4.2113	82.14	95.56
Auxin-responsive protein IAA27	Q9ZSY8	chain_1850	-4.2071	89.32	77.5
Ribulose bisphosphate carboxylase small chain	F4KA76	chain_7175	-4.1945	54.08	82.69
Ribulose bisphosphate carboxylase small chain 3B, chloroplastic	P10798	NODE_64850	-4.1846	70.97	80.95
GDSL esterase/lipase At1g33811	Q8L5Z1	NODE_40742	-4.1843	65.96	80.33
Chlorophyll a-b binding protein CP29.2, chloroplastic	Q9XF88	chain_33724	-4.1789	66.07	61.11
Putative oxygen-evolving enhancer protein 2-2	O49344	chain_60406	-4.1708	71.74	75.0
Putative leucine-rich repeat receptor-like serine/threonine-protein kinase At2g04300	Q9SI06	chain_10664	-4.1662	85.34	69.32
S-adenosyl-L-methionine-dependent methyltransferases superfamily protein	Q8GUP2	chain_24374	-4.1643	100.0	65.93
14-3-3-like protein GF14 iota	Q9C5W6	chain_65918	-4.1599	89.04	64.06
Cysteine-rich/transmembrane domain A-like protein	Q9SKY1	chain_14257	-4.1576	80.0	80.39
Probable GABA transporter 2	Q8L4X4	chain_54853	-4.1527	98.69	75.78
Glycerate dehydrogenase HPR, peroxisomal	Q9C9W5	chain_63834	-4.1402	50.72	93.94
G-type lectin S-receptor-like serine/threonine-protein kinase At2g19130	O64477	chain_7397	-4.136	51.72	68.38
Ribulose bisphosphate carboxylase small chain 3B, chloroplastic	P10798	chain_32244	-4.1334	59.57	88.89
Leucine-rich repeat protein kinase family protein	F4J927	chain_21456	-4.1215	81.71	67.74
Photosystem I reaction center subunit VI-1, chloroplastic	Q9SUI7	chain_26362	-4.0938	39.22	100.0
Cryptochrome-1	Q43125	chain_24641	-4.0917	30.0	85.0

continued on the next page

Contigs repressed at 34°C (*continued*)

Name	UniProt ID	Contig	$\log_2$ (fold-Change)	Target coverage (%)	Alignment identity (%)
Tropinone reductase homolog At2g29310	Q9ZW14	chain_3337	-4.0853	93.98	63.64
Folylpolyglutamate synthase	F4J2K2	NODE_10267	-4.0842	37.01	84.78
Cytochrome P450 714A1	Q93Z79	NODE_7665	-4.0809	86.49	76.17
Ribulose bisphosphate carboxylase/oxygenase activase, chloroplastic	P10896	NODE_50462	-4.0761	92.0	81.82
Leucine-rich repeat transmembrane protein kinase	F4I3K0	NODE_19558	-4.0754	32.12	60.47
Sucrose-phosphate synthase 1	Q94BT0	chain_6204	-4.0699	99.06	78.82
ABC transporter G family member 24	Q9MAG3	chain_35544	-4.0676	57.14	90.32
Chlorophyll a-b binding protein 2.1, chloroplastic	Q9SHR7	chain_45715	-4.0611	100	86.0
Probable sucrose-phosphate synthase 4	F4JLK2	chain_5983	-4.043	93.47	80.42
Aluminum induced protein with YGL and LRDR motifs	Q94BR2	NODE_39311	-4.0382	37.68	80.0
Chlorophyll a-b binding protein, chloroplastic	A0A1I9LMB4	chain_949	-4.0341	63.41	77.08
Chaperone protein dnaJ 3	Q94AW8	NODE_48441	-4.0291	55.0	90.48
Ribulose bisphosphate carboxylase/oxygenase activase, chloroplastic	P10896	chain_7328	-4.0265	94.41	93.04
Oxygen-evolving enhancer protein 1-2, chloroplastic	Q9S841	chain_17773	-4.0261	65.66	85.94
NADH–cytochrome b5 reductase 1	Q9ZNT1	NODE_15616	-4.0225	73.48	84.85
Bifunctional nuclease 1	Q9FWS6	chain_50260	-4.0194	57.5	95.45
Protochlorophyllide reductase B, chloroplastic	P21218	chain_29053	-4.0182	67.44	89.29
Beta-galactosidase 1	Q9SCW1	chain_58504	-4.0136	96.73	84.5
Serine carboxypeptidase-like 42	Q9FH05	chain_12903	-4.0129	92.9	78.11
Ribulose bisphosphate carboxylase small chain 3B, chloroplastic	P10798	NODE_72122	-4.0101	100.0	95.0
Cellulose synthase-like protein E1	Q8VZK9	chain_69817	-3.9975	96.77	69.75
Ribulose bisphosphate carboxylase small chain 3B, chloroplastic	P10798	chain_28934	-3.9943	41.38	86.96
Aluminum induced protein with YGL and LRDR motifs	A0A1P8B9Q2	chain_46447	-3.993	70.16	88.37
SOUL heme-binding family protein	Q8VZ90	chain_17645	-3.9856	98.64	70.14
CRN (Crooked neck) protein	Q9FNM3	chain_64195	-3.9812	30.49	91.67

continued on the next page

Contigs repressed at 34°C (*continued*)

Name	UniProt ID	Contig	$\log_2$ (fold-Change)	Target coverage (%)	Alignment identity (%)
Probable LRR receptor-like serine/threonine-protein kinase At1g51810	Q9FZB8	NODE_20165	-3.9783	42.31	80.95
Chlorophyll a-b binding protein 6, chloroplastic	Q01667	chain_54613	-3.9782	28.89	88.0
Ribulose bisphosphate carboxylase small chain 3B, chloroplastic	P10798	NODE_48601	-3.9774	48.84	85.0
Expressed protein	Q9SKI3	chain_22482	-3.975	26.51	60.47
Uncharacterized protein (Fragment)	A0A1P8B771	chain_4588	-3.9694	81.43	60.71
Wiskott-aldrich syndrome family protein, putative (DUF1118)	Q9SD79	chain_15674	-3.9689	62.77	79.49
Uncharacterized protein (Fragment)	A0A1P8B5H9	chain_59402	-3.9633	77.19	75.61
Ribulose bisphosphate carboxylase small chain 3B, chloroplastic	P10798	chain_3242	-3.9503	22.83	90.0
Photosystem II reaction center W protein, chloroplastic	Q39194	chain_64265	-3.9498	86.08	82.09
Oxygen-evolving enhancer protein 1-2, chloroplastic	Q9S841	chain_37120	-3.947	30.68	88.46
Indole-3-glycerol phosphate synthase, chloroplastic	P49572	chain_4315	-3.9461	71.36	75.49
Glycine dehydrogenase (decarboxylating) 2, mitochondrial	O80988	chain_29006	-3.9449	80.23	85.29
Rhodanese-like domain-containing protein 8, chloroplastic	F4I933	chain_20599	-3.9374	91.11	72.5
Photosystem I reaction center subunit N, chloroplastic	P49107	chain_69949	-3.9371	78.26	92.45
D-ribulose-5-phosphate-3-epimerase	Q9SAU2	chain_60109	-3.9345	27.4	89.47
Protein NDL1	Q9FJT7	NODE_47623	-3.9324	48.0	78.26
NAD(P)H-quinone oxidoreductase subunit T, chloroplastic	Q9SMS0	NODE_6774	-3.9182	57.41	74.68
Ribulose bisphosphate carboxylase/oxygenase activase, chloroplastic	P10896	chain_66439	-3.9173	32.22	78.57
Serine–glyoxylate aminotransferase	Q56YA5	chain_893	-3.916	94.83	85.98
Phosphoglycerate kinase 2, chloroplastic	P50318	chain_65828	-3.9157	72.06	85.42
ARM repeat superfamily protein	A0A1P8AX98	chain_5839	-3.9095	84.62	57.82
Serine carboxypeptidase-like 49	P32826	NODE_76300	-3.9089	57.14	89.47
Receptor-like protein 47	Q9SVN2	chain_66259	-3.9057	48.15	63.16
Protochlorophyllide reductase A, chloroplastic	Q42536	chain_3423	-3.9005	100.0	92.35
Mitochondrial outer membrane protein porin 1	Q9SRH5	NODE_46655	-3.8987	58.73	61.11

continued on the next page

Contigs repressed at 34°C (*continued*)

Name	UniProt ID	Contig	log_2 (fold-Change)	Target coverage (%)	Alignment identity (%)
Wall-associated receptor kinase-like 4	Q9S9M2	chain_11961	-3.8966	25.78	68.75
Ribulose bisphosphate carboxylase small chain 3B, chloroplastic	P10798	chain_33792	-3.8964	88.33	84.62
UDP-rhamnose/UDP-galactose transporter 4	Q8RWW7	chain_1187	-3.8934	93.47	84.08
Ribulose bisphosphate carboxylase small chain 1A, chloroplastic	P10795	chain_43697	-3.8912	73.64	77.42
Ribulose bisphosphate carboxylase small chain 3B, chloroplastic	P10798	chain_29805	-3.889	95.83	89.74
Auxin-responsive protein SAUR77	Q9LQI6	chain_54399	-3.8844	85.71	81.16
Magnesium-protoporphyrin IX monomethyl ester [oxidative] cyclase, chloroplastic	Q9M591	chain_1296	-3.8729	65.95	90.79
Germin-like protein subfamily 3 member 3	P94072	chain_53717	-3.8626	95.95	71.52
Photosystem I chlorophyll a/b-binding protein 2, chloroplastic	Q9SYW8	chain_14692	-3.859	87.4	90.91
Glutathione S-transferase L1	Q6NLB0	NODE_35979	-3.8572	98.51	75.38
Cytochrome P450 71A26	Q9STK7	chain_10846	-3.857	84.75	58.28
At1g50380/F14I3_27	Q9SX53	chain_19980	-3.8559	52.38	85.71
Probable LRR receptor-like serine/threonine-protein kinase At1g07550	C0LGD8	chain_14358	-3.8514	50.54	58.93
Ribulose bisphosphate carboxylase small chain 1A, chloroplastic	P10795	chain_50278	-3.8502	71.52	77.68
DVL19	Q6IM82	NODE_61964	-3.8473	63.04	71.43
Cytochrome P450 78A5	Q9LMX7	chain_15367	-3.8261	99.28	78.37
Probable histone H2A.4	Q9LZ46	chain_22156	-3.8237	79.43	92.75
Photosystem I reaction center subunit PSI-N, chloroplast, putative / PSI-N, putative (PSAN)	F4KC80	chain_22743	-3.8184	43.96	64.1
Serine hydroxymethyltransferase 1, mitochondrial	Q9SZJ5	chain_45420	-3.8063	94.2	92.19
Photosystem II 22 kDa protein, chloroplastic	Q9XF91	chain_25340	-3.8045	66.07	88.89
Ribulose bisphosphate carboxylase/oxygenase activase, chloroplastic	P10896	chain_59325	-3.8008	40.63	78.95
24-methylenesterol C-methyltransferase 3	Q94JS4	chain_49799	-3.7973	90.24	78.18
Ribulose bisphosphate carboxylase small chain 3B, chloroplastic	P10798	chain_31744	-3.7886	88.57	90.0
Cellulose synthase-like protein E1	Q8VZK9	chain_69631	-3.7768	35.92	75.0

continued on the next page

Contigs repressed at 34°C (*continued*)

Name	UniProt ID	Contig	$\log_2$ (fold-Change)	Target coverage (%)	Alignment identity (%)
Putative elongation factor TypA-like SVR3, chloroplastic	F4K410	chain_35853	-3.7666	53.33	91.3
Protein CONSERVED IN THE GREEN LINEAGE AND DIATOMS 27, chloroplastic	Q9FN15	chain_51039	-3.7595	66.2	84.78
Malate dehydrogenase 2, peroxisomal	Q9ZP05	NODE_15697	-3.7514	97.9	89.21
Ribulose bisphosphate carboxylase/oxygenase activase, chloroplastic	P10896	chain_39007	-3.7507	54.74	96.08
Glucomannan 4-beta-mannosyltransferase 9	Q9LZR3	chain_10294	-3.7404	93.86	86.38
Ypt/Rab-GAP domain of gyp1p superfamily protein	F4JX92	chain_33913	-3.7394	55.96	66.12
Serine–glyoxylate aminotransferase	Q56YA5	NODE_75085	-3.7225	75.76	79.17
Fructose-bisphosphate aldolase 6, cytosolic	Q9SJQ9	chain_58138	-3.7193	44.9	80.95
Ribulose bisphosphate carboxylase small chain	F4KA76	chain_57923	-3.7168	100	86.36
Uncharacterized protein	A0A1P8AUF7	chain_56551	-3.7108	69.05	71.43
Sterol 14-demethylase	Q9SAA9	chain_8215	-3.703	64.47	79.17
Alpha,alpha-trehalose-phosphate synthase [UDP-forming] 1	Q9SYM4	chain_6957	-3.7028	89.38	85.99
Ferredoxin-dependent glutamate synthase 1, chloroplastic/mitochondrial	Q9ZNZ7	chain_62344	-3.6988	35.9	77.78
DDT domain-containing protein PTM	F4JYC8	chain_24440	-3.6983	70.63	59.77
Serine–glyoxylate aminotransferase	Q56YA5	chain_862	-3.6903	93.99	86.79
Triose phosphate/phosphate translocator TPT, chloroplastic	Q9ZSR7	chain_20490	-3.6865	35.62	84.0
Pentatricopeptide repeat-containing protein At3g22150, chloroplastic	Q9LIE7	chain_66564	-3.6823	50.0	83.33
Oligopeptide transporter 5	Q9SUA4	NODE_16110	-3.6654	90.7	73.51
Dipeptidyl peptidase IV-like protein	Q9FNF6	chain_24897	-3.6575	100	86.96
Oxygen-evolving enhancer protein 1-2, chloroplastic	Q9S841	chain_61514	-3.6502	48.6	76.47
WAT1-related protein At3g18200	Q9LV20	NODE_7957	-3.6382	52.13	54.17
Transmembrane protein, putative (DUF247)	Q9SN06	NODE_17201	-3.6368	38.26	60.47
Aminopeptidase P1	F4JQH3	chain_59960	-3.6333	51.16	85.71
Chlorophyll a-b binding protein 3, chloroplastic	Q8VZ87	chain_22490	-3.6332	67.65	90.91
AP-4 complex subunit mu	Q9SB50	chain_22138	-3.6325	80.09	83.33

continued on the next page

Contigs repressed at 34°C (*continued*)

Name	UniProt ID	Contig	$\log_2$ (fold-Change)	Target coverage (%)	Alignment identity (%)
Oxygen-evolving enhancer protein 1-2, chloroplastic	Q9S841	chain_55558	-3.6294	49.09	92.31
Ribulose bisphosphate carboxylase small chain 1A, chloroplastic	P10795	chain_28806	-3.6228	30.14	85.71
Polyubiquitin 9	Q9FHQ6	chain_42360	-3.599	53.45	89.57
Glutamine synthetase cytosolic isozyme 1-2	Q8LCE1	NODE_45505	-3.5977	35.29	91.3
Glutamate receptor 1.3	Q9FH75	chain_56619	-3.5968	50.0	71.43
Subtilisin-like protease SBT1.7	O65351	NODE_550	-3.5928	89.77	72.17
Alpha 1,4-glycosyltransferase family protein	F4IS01	NODE_27404	-3.5826	42.96	74.47
Elongation factor Ts, mitochondrial	Q9SZD6	chain_18493	-3.5822	68.75	90.48
Photosystem I subunit O	Q949Q5	chain_39167	-3.5817	54.55	82.61
GDSL esterase/lipase 1	Q9FLN0	chain_66576	-3.5774	63.97	73.33
At2g44370	O64871	NODE_18611	-3.5773	19.89	69.44
Probable sucrose-phosphate synthase 3	Q8RY24	NODE_6604	-3.5756	90.16	70.97
At2g21530	Q8GWP4	chain_1728	-3.5696	81.48	82.35
Tetrapyrrole-binding protein, chloroplastic	Q9LX31	chain_65241	-3.5604	37.68	84.0
60S ribosomal protein L13a-3	Q9SVR0	chain_25034	-3.5574	43.1	79.59
Ferredoxin–NADP reductase, leaf isozyme 1, chloroplastic	Q9FKW6	chain_28585	-3.5547	67.65	90.91
Fructose-bisphosphate aldolase 6, cytosolic	Q9SJQ9	NODE_39316	-3.5544	90.0	83.02
Ribulose bisphosphate carboxylase/oxygenase activase, chloroplastic	P10896	chain_66426	-3.5325	45.61	88.0
Magnesium-protoporphyrin IX monomethyl ester [oxidative] cyclase, chloroplastic	Q9M591	chain_14683	-3.5305	81.16	92.22
Ribulose bisphosphate carboxylase/oxygenase activase, chloroplastic	P10896	chain_12060	-3.5285	84.98	94.42
Expressed protein	Q9ZU82	NODE_8527	-3.5249	45.63	84.95
Auxin response factor 6	Q9ZTX8	NODE_16102	-3.5212	46.58	93.94
Chlorophyll a-b binding protein 2.4, chloroplastic	Q9XF87	chain_36853	-3.5075	95.77	86.57
Allantoate deiminase	O49434	chain_6593	-3.4992	80.41	77.01

continued on the next page

Contigs repressed at 34°C (*continued*)

Name	UniProt ID	Contig	$\log_2$ (fold-Change)	Target coverage (%)	Alignment identity (%)
At2g39210/T16B24.15	O80960	NODE_24545	-3.499	58.82	71.43
Photosystem I subunit O	Q949Q5	chain_40	-3.4882	22.41	84.0
ABC transporter C family member 9	Q9M1C7	NODE_70542	-3.4878	100.0	71.43
Ferredoxin–NADP reductase, leaf isozyme 1, chloroplastic	Q9FKW6	chain_69695	-3.4875	54.21	91.23
PGR5-like protein 1A, chloroplastic	Q8H112	chain_28263	-3.483	95.37	83.33
Ribulose bisphosphate carboxylase/oxygenase activase, chloroplastic	P10896	chain_60524	-3.4811	53.19	83.33
Ribulose bisphosphate carboxylase/oxygenase activase, chloroplastic	P10896	NODE_42259	-3.4806	73.63	93.94
Glyceraldehyde-3-phosphate dehydrogenase GAPA2, chloroplastic	Q9LPW0	chain_30002	-3.4791	56.0	88.89
Post-illumination chlorophyll fluorescence increase	F4J034	chain_43038	-3.4693	39.29	80.95
Ribulose bisphosphate carboxylase/oxygenase activase, chloroplastic	P10896	chain_63251	-3.4671	74.58	90.48
AT4g32260/F10M6_100	Q42139	chain_65664	-3.4518	86.36	82.14
Polyol transporter 5	Q8VZ80	chain_9119	-3.451	92.86	71.71
Alpha/beta-Hydrolases superfamily protein	Q8VZ57	NODE_39158	-3.4312	77.42	74.47
Ribulose bisphosphate carboxylase small chain	F4KA76	NODE_46242	-3.4273	63.95	81.48
DEAD-box ATP-dependent RNA helicase 3, chloroplastic	Q8L7S8	NODE_53916	-3.4239	68.18	70.45
Ribulose bisphosphate carboxylase small chain 3B, chloroplastic	P10798	chain_47356	-3.4205	27.84	84.62
Ribulose bisphosphate carboxylase small chain 3B, chloroplastic	P10798	chain_63029	-3.4138	83.33	87.88
F18B13.21 protein	Q9SSC6	NODE_2373	-3.411	80.65	66.67
Glutamate dehydrogenase 1	Q43314	chain_2301	-3.4092	100	89.72
Photosystem II reaction center W protein, chloroplastic	Q39194	chain_48796	-3.4088	68.0	78.79
Oligopeptide transporter 1	Q9FG72	chain_41981	-3.4028	71.69	67.83
KDEL-tailed cysteine endopeptidase CEP3	Q9STL5	chain_56	-3.4021	70.8	68.35
Putative cysteine-rich receptor-like protein kinase 35	Q9LDQ3	NODE_19847	-3.4015	87.5	55.07
Glyceraldehyde-3-phosphate dehydrogenase GAPA1, chloroplastic	P25856	chain_68459	-3.3831	87.67	80.95

continued on the next page

Contigs repressed at 34°C (*continued*)

Name	UniProt ID	Contig	$\log_2$ (fold-Change)	Target coverage (%)	Alignment identity (%)
Tryptophan synthase beta chain 2, chloroplastic	P25269	NODE_75582	-3.3699	52.27	95.45
Plant UBX domain-containing protein 3	Q9SUG6	chain_1706	-3.3689	88.12	81.98
Ribulose bisphosphate carboxylase/oxygenase activase, chloroplastic	P10896	chain_48539	-3.3527	40.43	94.44
Glyceraldehyde-3-phosphate dehydrogenase GAPB, chloroplastic	P25857	chain_576	-3.3466	44.17	90.14
ABC transporter B family member 9	Q9M0M2	chain_52761	-3.3457	27.41	80.56
MAP kinase	Q9LDN6	chain_66409	-3.3442	81.63	91.18
Ribulose bisphosphate carboxylase small chain 1A, chloroplastic	P10795	chain_58704	-3.3417	62.96	77.27
ABC transporter C family member 7	Q9LK62	NODE_7774	-3.3394	80.69	73.3
Chlorophyll a-b binding protein, chloroplastic	A0A1I9LMB4	chain_4798	-3.3377	55.56	86.21
Ribulose bisphosphate carboxylase/oxygenase activase, chloroplastic	P10896	chain_56521	-3.3375	83.05	93.81
Photosystem I reaction center subunit VI-1, chloroplastic	Q9SUI7	chain_54776	-3.3347	54.55	97.5
LAMMER-type protein kinase AFC2	A0A1P8B6P6	NODE_26412	-3.3318	70.0	85.29
Ubiquitin-like superfamily protein	Q8GXI1	NODE_25795	-3.3294	62.5	62.92
At5g65690	B5X574	chain_27271	-3.3217	97.87	92.7
Fructose-bisphosphate aldolase 6, cytosolic	Q9SJQ9	chain_4328	-3.3179	90.85	82.03
Photosystem I reaction center subunit N, chloroplastic	P49107	chain_21952	-3.3163	73.97	92.45
ATP synthase subunit delta, chloroplastic	Q9SSS9	chain_12714	-3.3155	30.73	75.93
Photosystem I reaction center subunit II-1, chloroplastic	Q9S7H1	chain_69932	-3.3132	86.61	93.75
Ribulose bisphosphate carboxylase/oxygenase activase, chloroplastic	P10896	chain_23285	-3.303	66.04	79.41
Photosystem I reaction center subunit VI-1, chloroplastic	Q9SUI7	chain_56199	-3.3016	45.59	76.67
Fructose-bisphosphate aldolase 2, chloroplastic	Q944G9	NODE_28922	-3.3004	100	54.0
Ferric reduction oxidase 3, mitochondrial	F4I4K7	chain_40113	-3.2975	44.83	54.9
VQ motif-containing protein 22	Q9LIE6	chain_12643	-3.2951	16.12	88.37
Cytochrome P450 71A23	Q9STL0	chain_42326	-3.2888	92.68	62.16

continued on the next page

Contigs repressed at 34°C (*continued*)

Name	UniProt ID	Contig	$\log_2$ (fold-Change)	Target coverage (%)	Alignment identity (%)
Protein PAM71-homolog, chloroplastic	Q9T0H9	chain_33337	-3.2837	88.0	85.71
Alpha,alpha-trehalose-phosphate synthase [UDP-forming] 1	Q9SYM4	chain_1973	-3.2756	79.64	86.26
5'-adenylylsulfate reductase 1, chloroplastic	P92979	NODE_6484	-3.2754	92.71	85.52
Wiskott-aldrich syndrome family protein, putative (DUF1118)	Q9SD79	chain_25596	-3.2683	87.27	78.26
Glutamate decarboxylase 3	Q9ZPS4	chain_61047	-3.263	52.27	90.91
Glyceraldehyde-3-phosphate dehydrogenase GAPA2, chloroplastic	Q9LPW0	chain_244	-3.2598	88.69	82.2
L-ascorbate peroxidase 1, cytosolic	Q05431	chain_47626	-3.2589	40.76	69.79
Fructose-bisphosphate aldolase 1, chloroplastic	Q9SJU4	NODE_66080	-3.2585	45.83	93.75
10 kDa chaperonin 1, chloroplastic	Q9M1C2	chain_48932	-3.258	35.85	94.44
Aspartate-glutamate racemase family	Q9XI28	NODE_8286	-3.2574	58.16	62.32
Chloroplast stem-loop binding protein of 41 kDa b, chloroplastic	Q9SA52	chain_57944	-3.2554	88.46	100.0
Sugar transport protein 4	Q39228	chain_10807	-3.2545	85.71	69.1
Protein phosphatase 2C 57	P49599	chain_50092	-3.2459	95.89	69.13
Beta carbonic anhydrase 2, chloroplastic	P42737	chain_18021	-3.2459	72.31	76.09
Eukaryotic translation initiation factor 5A-1	Q9XI91	chain_5084	-3.2443	70.21	87.5
Galactinol synthase 6	Q8H1S1	chain_36943	-3.2422	76.92	64.1
ATP-citrate synthase alpha chain protein 1	Q9SGY2	chain_34239	-3.233	87.83	83.04
Ribulose bisphosphate carboxylase small chain 3B, chloroplastic	P10798	NODE_64488	-3.2317	80.0	88.57
ATP-dependent Clp protease proteolytic subunit-related protein 3, chloroplastic	Q8L770	chain_27725	-3.228	94.19	84.83
Transmembrane protein, putative (DUF247)	Q9SN06	chain_70549	-3.2265	82.93	66.67
Probable calcium-binding protein CML25	Q9FYK2	chain_65418	-3.2121	38.65	64.41
Glyceraldehyde-3-phosphate dehydrogenase GAPB, chloroplastic	P25857	chain_43398	-3.2008	43.33	88.0
Receptor-like protein 39	F4J7T6	NODE_18450	-3.1951	61.98	56.48
Germin-like protein subfamily 3 member 1	P94040	chain_5475	-3.188	89.51	70.08
FtsH extracellular protease family	A0A1P8AXC1	chain_52065	-3.1805	57.89	95.24

continued on the next page

Contigs repressed at 34°C (*continued*)

Name	UniProt ID	Contig	$\log_2$ (fold-Change)	Target coverage (%)	Alignment identity (%)
Ribulose bisphosphate carboxylase/oxygenase activase, chloroplastic	P10896	NODE_52445	-3.1795	100	95.38
At2g15020	Q9ZUK9	chain_16229	-3.1776	99.57	68.37
Uncharacterized protein At3g61260	Q9M2D8	NODE_71987	-3.1766	79.07	72.73
AT5g40850/MHK7_8	Q42606	chain_156	-3.1744	90.4	84.27
Glutathione S-transferase F10	P42761	chain_5057	-3.172	93.51	67.8
RHOMBOID-like protein 9, chloroplastic	Q9FFX0	chain_13582	-3.1702	26.33	77.67
Splicing factor, CC1-like protein	Q93Y25	NODE_51106	-3.17	44.44	86.96
Aldolase-type TIM barrel family protein	A8MS37	chain_53746	-3.1695	52.27	90.91
Oxygen-evolving enhancer protein 1-2, chloroplastic	Q9S841	chain_65680	-3.1693	39.88	85.94
Probable aquaporin PIP2-4	Q9FF53	chain_27759	-3.1659	84.3	80.45
AP-1 complex subunit sigma-1	Q8LEZ8	chain_65122	-3.1625	52.38	90.48
Plastocyanin major isoform, chloroplastic	P42699	chain_40403	-3.1613	53.09	76.19
Fructose-bisphosphate aldolase 1, chloroplastic	Q9SJU4	chain_25487	-3.1609	28.99	89.47
Putative tRNA (cytidine(32)/guanosine(34)-2'-O)-methyltransferase	Q8GUN8	chain_42198	-3.1552	91.32	93.49
Ribulose bisphosphate carboxylase/oxygenase activase, chloroplastic	P10896	chain_65124	-3.1542	65.22	75.0
Probably inactive leucine-rich repeat receptor-like protein kinase At3g28040	Q9LRT1	chain_16061	-3.153	90.63	52.63
Chlorophyll a-b binding protein 2.1, chloroplastic	Q9SHR7	chain_53216	-3.1524	44.86	78.72
Chitinase-like protein 2	Q9LSP9	chain_60772	-3.1441	24.55	80.77
Lysine-tRNA ligase	Q8RWI4	chain_3710	-3.1429	82.68	90.03
Photosystem I reaction center subunit psaK, chloroplastic	Q9SUI5	chain_36817	-3.1419	42.86	88.46
Cytochrome b6-f complex iron-sulfur subunit, chloroplastic	Q9ZR03	chain_48079	-3.1417	61.11	90.48
Beta carbonic anhydrase 1, chloroplastic	P27140	chain_54997	-3.1348	97.96	80.85
Glyceraldehyde-3-phosphate dehydrogenase GAPB, chloroplastic	P25857	chain_689	-3.1302	44.17	90.14
S-adenosyl-L-methionine-dependent methyltransferases superfamily protein	Q8GUP2	chain_42163	-3.1262	100.0	65.93

continued on the next page

Contigs repressed at 34°C (*continued*)

Name	UniProt ID	Contig	$\log_2$ (fold-Change)	Target coverage (%)	Alignment identity (%)
G-type lectin S-receptor-like serine/threonine-protein kinase At4g27290	O81832	NODE_6679	-3.1251	47.8	66.67
Pentatricopeptide repeat-containing protein At1g62670, mitochondrial	Q9SXD1	chain_44435	-3.1228	83.45	57.73
At2g35260/T4C15.7	O82167	chain_50865	-3.1201	52.82	80.61
Phospholipase D zeta 1	Q9LRZ5	chain_44468	-3.1195	16.92	80.95
At1g62510	Q9SXE6	chain_794	-3.1188	76.15	65.0
Ribulose bisphosphate carboxylase/oxygenase activase, chloroplastic	P10896	chain_61301	-3.1174	37.66	92.86
Protein NRT1/ PTR FAMILY 6.1	Q9LYR6	NODE_6633	-3.1126	89.34	82.39
Photosystem II reaction center W protein, chloroplastic	Q39194	chain_815	-3.1108	70.24	84.48
Ribulose bisphosphate carboxylase/oxygenase activase, chloroplastic	P10896	chain_55603	-3.1084	29.09	93.55
Ribulose bisphosphate carboxylase small chain 3B, chloroplastic	P10798	chain_47690	-3.1051	42.97	86.27
At4g13270	Q52K84	chain_16395	-3.1033	92.75	53.54
Ribulose bisphosphate carboxylase small chain 1A, chloroplastic	P10795	chain_42126	-3.0991	69.63	77.17
Photosystem I chlorophyll a/b-binding protein 2, chloroplastic	Q9SYW8	chain_58510	-3.0961	50.0	90.0
60S ribosomal protein L36-2	Q9M352	chain_57087	-3.0897	87.18	93.94
Chlorophyll a-b binding protein CP26, chloroplastic	Q9XF89	NODE_34992	-3.0881	56.1	86.36
Putative polyol transporter 1	Q9XIH7	NODE_24481	-3.0874	88.3	77.03
F9L1.32 protein	Q9XI32	chain_49483	-3.0845	35.85	94.44
Tyrosine aminotransferase	Q9LVY1	NODE_30224	-3.0735	19.01	86.36
Chaperone protein dnaJ 3	Q94AW8	chain_69731	-3.073	35.9	85.19
Ribulose bisphosphate carboxylase small chain 1A, chloroplastic	P10795	NODE_68244	-3.0715	51.85	85.19
Photosystem II core complex proteins psbY, chloroplastic	O49347	chain_46001	-3.0678	22.16	73.91
Ribulose bisphosphate carboxylase/oxygenase activase, chloroplastic	P10896	chain_33869	-3.0655	46.28	76.36
Protein LURP-one-related 5	Q9SSC7	chain_9227	-3.0614	79.61	84.03
Auxin efflux carrier component 5	Q9FFD0	NODE_5758	-3.0594	92.38	70.83

continued on the next page

Contigs repressed at 34°C (*continued*)

Name	UniProt ID	Contig	$\log_2$ (fold-Change)	Target coverage (%)	Alignment identity (%)
At5g65690	B5X574	NODE_9056	-3.0542	96.36	87.62
Alanine-tRNA ligase	Q94AZ5	chain_47276	-3.0526	90.12	77.78
Ribulose bisphosphate carboxylase small chain 3B, chloroplastic	P10798	chain_49062	-3.0504	34.72	83.33
Transketolase-2, chloroplastic	F4IW47	chain_9886	-3.0475	91.18	85.41
Ribulose bisphosphate carboxylase/oxygenase activase, chloroplastic	P10896	chain_26982	-3.038	60.75	75.0
AT4g20760/F21C20_110	Q941A7	chain_12153	-3.0326	91.3	80.14
Ribulose bisphosphate carboxylase/oxygenase activase, chloroplastic	P10896	chain_44137	-3.0255	67.69	86.05
Catalase-1	Q96528	chain_7088	-3.0169	91.3	88.26
At1g50380/F14I3_27	Q9SX53	chain_61286	-3.0163	52.38	85.71
Exportin-2	Q9ZPY7	chain_4875	-3.0153	98.13	80.27
At2g35260/T4C15.7	O82167	NODE_14973	-3.0142	68.75	65.63
Ribulose bisphosphate carboxylase/oxygenase activase, chloroplastic	P10896	NODE_42722	-3.0091	80.0	92.31
D-tagatose-1,6-bisphosphate aldolase subunit	Q8L771	chain_51116	-3.0085	71.06	75.64
Caveolin-1 protein	F4I2E7	chain_12009	-3.0036	77.1	68.21
Photosystem II 10 kDa polypeptide, chloroplastic	P27202	chain_49591	-3.0021	68.6	72.73
DNA repair RAD52-like protein 2, chloroplastic	Q9FIJ4	chain_23565	-2.9985	80.77	84.68
Ribulose bisphosphate carboxylase/oxygenase activase, chloroplastic	P10896	chain_65985	-2.9946	65.38	78.79
Serine hydroxymethyltransferase 1, mitochondrial	Q9SZJ5	chain_58322	-2.9916	94.2	92.19
Fructose-bisphosphate aldolase 1, chloroplastic	Q9SJU4	NODE_54841	-2.9894	88.46	90.91
Calcium sensing receptor, chloroplastic	Q9FN48	chain_25439	-2.9836	96.57	71.86
At4g26810	Q9SZ21	chain_573	-2.9829	97.62	90.12
Ribulose bisphosphate carboxylase/oxygenase activase, chloroplastic	P10896	chain_3458	-2.9811	71.64	91.49
Photosystem I reaction center subunit XI, chloroplastic	Q9SUI4	chain_66171	-2.9809	43.75	90.0

continued on the next page

Contigs repressed at 34°C (*continued*)

Name	UniProt ID	Contig	$\log_2$ (fold-Change)	Target coverage (%)	Alignment identity (%)
AT5g40850/MHK7_8	Q42606	chain_70760	-2.9802	90.4	84.27
PPPDE thiol peptidase family protein	O49393	chain_8799	-2.9799	50.48	53.26
Glycine dehydrogenase (decarboxylating) 1, mitochondrial	Q94B78	chain_1030	-2.977	94.57	87.5
Glycine dehydrogenase (decarboxylating) 1, mitochondrial	Q94B78	NODE_71900	-2.9765	50.72	91.18
Glycine dehydrogenase (decarboxylating) 1, mitochondrial	Q94B78	chain_46390	-2.9702	77.33	92.98
S-adenosyl-L-methionine-dependent methyltransferases superfamily protein	Q8GUP2	chain_65886	-2.9697	100.0	65.93
Thioredoxin M4, chloroplastic	Q9SEU6	chain_50867	-2.9665	59.18	68.42
Phytochrome C	P14714	NODE_541	-2.9647	100	61.8
Leucine-rich repeat protein kinase family protein	F4IB62	NODE_37220	-2.9639	44.3	64.71
Glycosyltransferase (Fragment)	W8Q376	NODE_16238	-2.9636	96.92	68.36
Glycerate dehydrogenase HPR, peroxisomal	Q9C9W5	chain_56582	-2.963	50.0	88.0
Phospholipase D zeta 1	Q9LRZ5	chain_48191	-2.9612	22.68	80.95
Ribulose bisphosphate carboxylase small chain 3B, chloroplastic	P10798	chain_2290	-2.9552	61.9	77.92
Thioredoxin F2, chloroplastic	Q9XFH9	chain_24408	-2.9538	89.53	75.86
DHHA1 domain protein	F4HRG2	chain_26513	-2.9499	94.85	74.6
Fructose-bisphosphate aldolase 6, cytosolic	Q9SJQ9	chain_25699	-2.9346	65.63	90.0
Probable NAD(P)H dehydrogenase (quinone) FQR1-like 1	Q6NQE2	chain_8413	-2.9343	93.88	74.73
Ribulose bisphosphate carboxylase small chain 3B, chloroplastic	P10798	NODE_74957	-2.929	50.0	82.61
Leucine-rich repeat protein kinase family protein	F4KGL1	chain_33021	-2.9243	76.33	75.63
NAP1-related protein 1	Q9CA59	chain_616	-2.9209	87.21	75.68
Transcription factor MYB3	Q9S9K9	chain_69493	-2.9207	51.0	77.78
Peptidyl-prolyl cis-trans isomerase FKBP62	Q38931	chain_8207	-2.9177	81.65	63.64
Plant VAMP (Vesicle-associated membrane protein) family protein	A0A1P8B930	chain_1785	-2.9151	86.94	73.3
Probable protein phosphatase 2C 34	Q9M9W9	chain_8069	-2.9131	96.45	72.84
Thioredoxin M4, chloroplastic	Q9SEU6	NODE_64216	-2.9077	36.36	89.47
60S ribosomal protein L36-2	Q9M352	chain_2085	-2.9039	87.18	93.94

continued on the next page

Contigs repressed at 34°C (*continued*)

Name	UniProt ID	Contig	log_2 (fold-Change)	Target coverage (%)	Alignment identity (%)
Receptor protein kinase CLAVATA1	Q9SYQ8	NODE_20419	-2.9033	75.82	86.13
General transcription and DNA repair factor IIH helicase subunit XPB2	Q9FUG4	chain_67165	-2.8992	63.46	93.75
Glyceraldehyde-3-phosphate dehydrogenase GAPA2, chloroplastic	Q9LPW0	chain_27719	-2.8977	100.0	90.77
At1g51400/F5D21_10	Q9SYE2	NODE_73690	-2.8936	96.67	85.71
Senescence-induced receptor-like serine/threonine-protein kinase	O64483	NODE_24309	-2.8872	37.75	57.14
At3g49890	Q9M2W9	chain_15804	-2.8854	40.3	62.37
Ferric reduction oxidase 5	Q9FLW2	NODE_30159	-2.8809	86.07	63.46
Histidine-containing phosphotransfer protein 5	Q8L9T7	chain_1080	-2.8784	87.5	63.57
Ribulose bisphosphate carboxylase/oxygenase activase, chloroplastic	P10896	chain_55353	-2.8779	47.92	90.91
Malate dehydrogenase 2, peroxisomal	Q9ZP05	chain_5172	-2.868	96.85	91.43
Aminomethyltransferase, mitochondrial	O65396	chain_36053	-2.8619	97.96	84.1
Ethylene-responsive transcription factor ERF107	Q9FKG2	chain_1501	-2.8588	24.28	81.82
Ferredoxin–NADP reductase, chloroplastic	F4JZ46	chain_30006	-2.8556	29.46	94.59
Ribulose bisphosphate carboxylase small chain	F4KA76	chain_70918	-2.8549	23.08	79.31
Phytochrome-interacting ankyrin-repeat protein 2	Q9FNP4	chain_8316	-2.8458	82.76	77.89
Phospholipase D zeta 1	Q9LRZ5	chain_47857	-2.8415	22.68	80.95
Sedoheptulose-1,7-bisphosphatase, chloroplastic	P46283	chain_6625	-2.8375	91.46	91.95
Threonine–tRNA ligase, chloroplastic/mitochondrial 2	F4IFC5	chain_6649	-2.824	82.14	87.43
Protein kinase superfamily protein	Q9C5K4	chain_65732	-2.8239	83.33	62.77
At1g70820	Q9SSL0	NODE_1020	-2.82	87.48	83.46
At1g63855	Q8GWK3	chain_2050	-2.8178	72.48	77.57
Chlorophyll a-b binding protein 6, chloroplastic	Q01667	chain_70664	-2.8155	25.84	86.36
Phosphate transporter PHO1 homolog 1	Q93ZF5	NODE_753	-2.8152	86.59	78.0
6-phosphogluconate dehydrogenase, decarboxylating 3	Q9FWA3	chain_47295	-2.8101	36.54	88.89
Ribulose bisphosphate carboxylase/oxygenase activase, chloroplastic	P10896	NODE_54348	-2.81	81.25	100.0

continued on the next page

Contigs repressed at 34°C (*continued*)

Name	UniProt ID	Contig	$\log_2$ (fold-Change)	Target coverage (%)	Alignment identity (%)
Folylpolyglutamate synthase	F4J2K2	NODE_13055	-2.8094	86.6	79.14
Leucine carboxyl methyltransferase	F4K327	chain_2134	-2.8	87.1	73.17
Ferric reduction oxidase 8, mitochondrial	Q8VY13	chain_34174	-2.7979	56.63	64.52
Glutamate–glyoxylate aminotransferase 2	Q9S7E9	chain_5682	-2.7919	92.92	86.11
Lysine-tRNA ligase	Q8RWI4	chain_180	-2.7917	96.62	88.94
Adenine nucleotide alpha hydrolases-like superfamily protein	Q93W91	chain_25421	-2.7852	73.56	66.96
Haloacid dehalogenase-like hydrolase domain-containing protein At4g39970	Q680K2	chain_2246	-2.7814	67.98	83.08
NADPH-protochlorophyllide oxidoreductase	F4I2F8	NODE_37196	-2.7719	63.64	93.75
Somatic embryogenesis receptor kinase 2	Q9XIC7	NODE_17780	-2.7647	96.65	77.66
Chaperone protein dnaJ 3	Q94AW8	chain_2631	-2.7604	35.9	85.19
CRM-domain containing factor CFM3A, chloroplastic/mitochondrial	F4J2U9	chain_13109	-2.7594	85.56	71.83
Aminomethyltransferase, mitochondrial	O65396	chain_2568	-2.7577	68.92	90.0
Ribulose bisphosphate carboxylase/oxygenase activase, chloroplastic	P10896	chain_21068	-2.7573	81.52	80.6
Chlorophyll a-b binding protein CP26, chloroplastic	Q9XF89	chain_45057	-2.7543	46.0	95.45
Glyceraldehyde-3-phosphate dehydrogenase GAPA1, chloroplastic	P25856	chain_32320	-2.754	53.23	93.75
Photosystem II 22 kDa protein, chloroplastic	Q9XF91	chain_268	-2.7535	76.58	83.14
BTB/POZ domain-containing protein At3g44820	Q9FYC8	chain_9280	-2.752	85.87	78.21
Protochlorophyllide reductase B, chloroplastic	P21218	chain_26457	-2.7518	79.63	95.24
Ferredoxin–NADP reductase, leaf isozyme 1, chloroplastic	Q9FKW6	NODE_60988	-2.7517	73.33	95.24
DHHA1 domain protein	F4HRG2	chain_2862	-2.7491	94.85	74.6
Cysteine/Histidine-rich C1 domain family protein	Q9FM59	NODE_15781	-2.7473	20.47	64.71
Chlorophyll a-b binding protein 6, chloroplastic	Q01667	chain_26216	-2.7466	50.0	87.72
Thiamine thiazole synthase, chloroplastic	Q38814	chain_46166	-2.7435	89.91	95.88
Glyceraldehyde 3-phosphate dehydrogenase A subunit 2	F4HNZ6	chain_66812	-2.741	71.11	77.42
ATP-dependent Clp protease ATP-binding subunit	Q9FIS4	chain_55284	-2.7394	27.5	68.75

continued on the next page

Contigs repressed at 34°C (*continued*)

Name	UniProt ID	Contig	$\log_2$ (fold-Change)	Target coverage (%)	Alignment identity (%)
Rhodanese-like domain-containing protein 9, chloroplastic	O48529	NODE_58459	-2.7386	100.0	90.63
Alpha/beta-Hydrolases superfamily protein	Q9LR26	chain_5311	-2.7363	87.8	78.84
Ribulose bisphosphate carboxylase/oxygenase activase, chloroplastic	P10896	chain_39031	-2.7351	77.78	79.41
Histone H2B.4	Q9ZUS0	chain_10168	-2.7327	68.03	79.27
Ribulose bisphosphate carboxylase small chain 3B, chloroplastic	P10798	chain_69750	-2.7312	59.7	84.62
MLO-like protein 2	Q9SXB6	chain_4607	-2.7254	85.07	74.61
S-adenosyl-L-methionine-dependent methyltransferases superfamily protein	Q8GUP2	chain_24975	-2.7251	100.0	65.93
Pathogenesis-related family protein	Q8L9B0	chain_6351	-2.7215	91.72	64.18
3-epi-6-deoxocathasterone 23-monooxygenase	Q9M066	chain_2068	-2.7201	78.38	83.09
NADPH-protochlorophyllide oxidoreductase	F4I2F8	chain_47774	-2.7196	46.51	89.47
Peptidyl-prolyl cis-trans isomerase FKBP17-2, chloroplastic	Q9LDY5	chain_615	-2.7107	99.05	75.73
Protein NRT1/ PTR FAMILY 8.2	Q9LFB8	NODE_33288	-2.7088	84.15	66.18
Triose phosphate/phosphate translocator TPT, chloroplastic	Q9ZSR7	chain_61636	-2.7074	44.62	96.43
Uncharacterized protein	A0A1P8AUF7	chain_4059	-2.7063	58.59	73.21
40S ribosomal protein S11-3	P42733	chain_112	-2.7041	97.56	90.57
Cytokinin riboside 5'-monophosphate phosphoribohydrolase LOG5	Q8LBB7	NODE_11235	-2.702	81.15	64.47
Photosystem I subunit O	Q949Q5	chain_54650	-2.6993	73.56	87.3
Chlorophyll a-b binding protein 3, chloroplastic	Q8VZ87	chain_21721	-2.6933	46.94	90.91
Calmodulin-like protein 2	Q9SU00	chain_4249	-2.6929	31.03	64.79
Sedoheptulose-1,7-bisphosphatase, chloroplastic	P46283	NODE_73091	-2.6851	70.59	95.74
Aminomethyltransferase, mitochondrial	O65396	chain_27132	-2.6812	98.65	88.89
Serine–glyoxylate aminotransferase	Q56YA5	chain_65280	-2.6796	93.81	86.76
U-box domain-containing protein 44	Q9LM76	chain_51449	-2.6792	99.0	69.15
ATP synthase subunit delta', mitochondrial	Q96252	chain_64570	-2.6749	76.44	77.19
Ribulose bisphosphate carboxylase small chain 1A, chloroplastic	P10795	chain_18102	-2.6742	79.85	79.81

continued on the next page

Contigs repressed at 34°C (*continued*)

Name	UniProt ID	Contig	$\log_2$ (fold-Change)	Target coverage (%)	Alignment identity (%)
Protochlorophyllide reductase A, chloroplastic	Q42536	chain_8868	-2.6717	100.0	92.35
Photosystem I reaction center subunit II-1, chloroplastic	Q9S7H1	chain_56829	-2.6713	82.58	92.59
Ribulose bisphosphate carboxylase small chain 1A, chloroplastic	P10795	chain_20368	-2.6712	63.04	78.43
Aldehyde dehydrogenase family 7 member B4	Q9SYG7	chain_49354	-2.6694	28.95	95.24
Lycopene beta/epsilon cyclase protein	Q0WVA1	chain_8330	-2.6674	99.49	79.49
Protein RADIALIS-like 1	F4JVB8	chain_63321	-2.6658	54.17	76.0
Putative tRNA (cytidine(32)/guanosine(34)-2'-O)-methyltransferase	Q8GUN8	chain_13020	-2.6644	75.57	93.49
Aminomethyltransferase, mitochondrial	O65396	chain_55942	-2.6561	72.73	95.65
Chlorophyll a-b binding protein 6, chloroplastic	Q01667	chain_66819	-2.6515	33.33	85.71
Chlorophyll a-b binding protein 3, chloroplastic	Q9S7M0	chain_46822	-2.6497	35.85	94.44
Protein NRT1/ PTR FAMILY 2.13	Q8RX77	NODE_1559	-2.649	86.97	70.75
Chlorophyll a-b binding protein, chloroplastic	Q9LMQ2	chain_24109	-2.6489	58.97	90.91
Photosystem I reaction center subunit N, chloroplastic	P49107	chain_33371	-2.6423	78.26	92.45
Tetratricopeptide repeat (TPR)-like superfamily protein	F4IJS7	chain_4384	-2.6418	90.26	79.07
At2g15020	Q9ZUK9	chain_11801	-2.6416	59.2	69.5
Ribulose bisphosphate carboxylase small chain 2B, chloroplastic	P10797	chain_68643	-2.6362	36.0	80.77
Glutamate receptor 3.3	Q9C8E7	NODE_7103	-2.6346	99.69	63.97
Fatty acid desaturase 4, chloroplastic	Q9SZ42	chain_5685	-2.6338	87.36	75.21
At2g44370	O64871	chain_11637	-2.6289	21.51	69.44
Photosystem I reaction center subunit II-2, chloroplastic	Q9SA56	chain_55971	-2.6289	47.37	94.12
Probable lipid desaturase ADS3.2, chloroplastic	Q9LVZ3	NODE_9573	-2.6274	83.24	70.59
Probable alpha,alpha-trehalose-phosphate synthase [UDP-forming] 11	Q9ZV48	chain_6991	-2.6265	90.31	71.24
ABC transporter G family member 34	Q7PC87	chain_12130	-2.6258	98.44	68.02
30S ribosomal protein	Q94K97	NODE_15206	-2.6237	88.74	79.28
Phospholipase D zeta 1	Q9LRZ5	chain_35569	-2.6199	22.68	80.95

continued on the next page

Contigs repressed at 34°C (*continued*)

Name	UniProt ID	Contig	$\log_2$ (fold-Change)	Target coverage (%)	Alignment identity (%)
Ribulose bisphosphate carboxylase/oxygenase activase, chloroplastic	P10896	chain_29655	-2.6175	51.16	85.71
Photosystem I subunit O	Q949Q5	chain_278	-2.6155	70.45	88.52
ABC transporter G family member 34	Q7PC87	chain_49797	-2.6139	49.1	78.75
Photosystem I reaction center subunit XI, chloroplastic	Q9SUI4	chain_43974	-2.6116	79.61	85.19
Ribulose bisphosphate carboxylase small chain 3B, chloroplastic	P10798	chain_48836	-2.6111	65.63	85.37
AT3G08760 protein	Q9C9Y3	NODE_10376	-2.6042	58.76	76.85
Alanine–glyoxylate aminotransferase 2 homolog 3, mitochondrial	Q9SR86	NODE_14836	-2.6042	95.5	80.95
Photosystem I reaction center subunit III, chloroplastic	Q9SHE8	chain_9912	-2.6028	89.88	89.33
Putative mandelonitrile lyase	Q8GXX6	NODE_6960	-2.598	79.89	63.21
Ribulose bisphosphate carboxylase small chain 1A, chloroplastic	P10795	chain_58702	-2.5942	80.81	75.64
Probable pre-mRNA-splicing factor ATP-dependent RNA helicase DEAH9	F4JRJ6	chain_61803	-2.5915	92.88	78.67
AT1g18060/T10F20.23	Q9LM40	NODE_10891	-2.5894	98.57	84.33
Catalase 3	A0A1P8AWT7	chain_569	-2.5838	81.82	79.03
Ribulose bisphosphate carboxylase small chain 1A, chloroplastic	P10795	chain_44341	-2.5799	70.66	77.68
Pathogenesis-related family protein	F4IBV1	chain_14629	-2.5791	37.74	54.17
AT4g32260/F10M6_100	Q42139	chain_27861	-2.5729	86.36	82.14
Late embryogenesis abundant (LEA) hydroxyproline-rich glycoprotein family	Q9M287	chain_12587	-2.5727	46.25	80.56
Plastocyanin	A0A1P8APR2	chain_61089	-2.571	52.27	86.36
O-fucosyltransferase 11	Q8GUM0	NODE_3920	-2.5689	66.2	70.8
Cellulose synthase-like protein E1	Q8VZK9	NODE_13702	-2.5675	81.5	75.18
Ribulose bisphosphate carboxylase small chain 1A, chloroplastic	P10795	chain_50537	-2.5671	66.93	78.57
Glutamate receptor 3.2	Q93YT1	NODE_11324	-2.565	74.43	64.81
Phytol kinase 1, chloroplastic	Q9LZ76	chain_57612	-2.5631	39.29	78.13
Protein NOI4	O22633	chain_58200	-2.5621	28.77	95.0
UDP-glycosyltransferase 85A2	Q9ZWJ3	chain_31676	-2.5598	94.2	65.35

continued on the next page

Contigs repressed at 34°C (*continued*)

Name	UniProt ID	Contig	$\log_2$ (fold-Change)	Target coverage (%)	Alignment identity (%)
Photosystem I reaction center subunit VI-2, chloroplastic	Q9SUI6	chain_68757	-2.5565	71.58	98.51
Photosystem I reaction center subunit psaK, chloroplastic	Q9SUI5	NODE_71578	-2.5533	62.16	86.36
Probable xyloglucan endotransglucosylase/hydrolase protein 6	Q8LF99	chain_43963	-2.5518	34.09	79.31
Putative tRNA (cytidine(32)/guanosine(34)-2'-O)-methyltransferase	Q8GUN8	chain_10103	-2.5477	76.01	93.49
Fructose-bisphosphate aldolase 6, cytosolic	Q9SJQ9	NODE_6733	-2.5466	94.87	84.93
ABC transporter D family member 2, chloroplastic	Q6NLC1	chain_4939	-2.5455	95.55	79.6
Transmembrane protein, putative (DUF247)	Q9SN06	chain_27690	-2.5452	28.65	57.69
Phosphoribulokinase, chloroplastic	P25697	chain_1542	-2.5449	85.92	93.39
PsbP domain-containing protein 3, chloroplastic	Q9S720	NODE_9967	-2.5442	93.55	72.83
Ribulose bisphosphate carboxylase/oxygenase activase, chloroplastic	P10896	NODE_48193	-2.5424	89.36	78.05
Ribulose bisphosphate carboxylase/oxygenase activase, chloroplastic	P10896	NODE_51277	-2.5404	84.0	90.0
High mobility group B protein 9	Q9SGS2	chain_5608	-2.5393	93.23	79.77
Ferredoxin–NADP reductase, leaf isozyme 2, chloroplastic	Q8W493	chain_34548	-2.5366	59.52	79.17
Type I inositol polyphosphate 5-phosphatase 8	Q0WT19	NODE_9586	-2.5353	88.46	67.97
At4g28230	Q9M0I4	NODE_6133	-2.5287	76.77	72.14
Photosystem II core complex proteins psbY, chloroplastic	O49347	chain_12524	-2.5284	63.44	76.16
Catalase-3	Q42547	chain_19271	-2.5263	74.75	67.69
Fe-S cluster assembly factor HCF101, chloroplastic	Q6STH5	chain_23129	-2.5218	95.21	87.08
Photosynthetic NDH subunit of subcomplex B 2, chloroplastic	Q94AQ8	chain_2171	-2.5207	73.5	66.26
Fructose-bisphosphate aldolase 3, chloroplastic	Q9ZU52	chain_3431	-2.5201	52.25	80.0
Ribulose bisphosphate carboxylase/oxygenase activase, chloroplastic	P10896	NODE_42258	-2.5165	60.44	90.74
Protein NRT1/ PTR FAMILY 5.1	Q8VZR7	chain_10620	-2.5159	98.04	78.85
Oxygen-evolving enhancer protein 1-2, chloroplastic	Q9S841	chain_54602	-2.5147	61.7	92.86
Ascorbate transporter, chloroplastic	Q8GX78	NODE_27187	-2.5123	100	87.32

continued on the next page

Contigs repressed at 34°C (*continued*)

Name	UniProt ID	Contig	$\log_2$ (fold-Change)	Target coverage (%)	Alignment identity (%)
At5g65690	B5X574	chain_5428	-2.5077	95.57	91.57
Phosphoribulokinase, chloroplastic	P25697	NODE_52935	-2.5068	65.0	96.08
Transducin family protein / WD-40 repeat family protein	Q9LIM7	chain_24135	-2.5057	78.9	63.64
Cytochrome P450 82C3	O49396	chain_55461	-2.4976	88.79	58.46
Rho termination factor	F4JRN0	NODE_1330	-2.4955	21.38	80.7
Haloacid dehalogenase-like hydrolase (HAD) superfamily protein	A0A1P8BF18	chain_7700	-2.4926	85.32	69.27
Monothiol glutaredoxin-S10	Q9LIF1	NODE_11666	-2.4908	93.52	58.0
Oxygen-evolving enhancer protein 1-2, chloroplastic	Q9S841	chain_8081	-2.4844	94.34	83.33
TRM32-like protein (DUF3741)	Q9LZ87	chain_7709	-2.4823	6.47	58.14
Dbj—BAA84809.1	Q9FHX6	chain_3072	-2.4822	92.81	84.29
Ribulose bisphosphate carboxylase small chain 3B, chloroplastic	P10798	chain_49913	-2.4817	75.58	83.33
NAD(P)H-quinone oxidoreductase subunit M, chloroplastic	Q2V2S7	chain_1202	-2.4816	86.5	80.15
Thiamine thiazole synthase, chloroplastic	Q38814	chain_69414	-2.4814	99.48	88.89
At1g72510	Q9C9E9	chain_916	-2.475	65.77	66.67
Serine hydroxymethyltransferase 1, mitochondrial	Q9SZJ5	chain_2600	-2.4749	93.0	91.3
Probable protein phosphatase 2C 46	Q9SD12	chain_44539	-2.4746	95.74	82.09
Chlorophyll a-b binding protein 6, chloroplastic	Q01667	chain_68501	-2.4707	37.78	93.94
Protein kinase superfamily protein	Q9FYC5	chain_8358	-2.4707	61.74	78.95
At2g04039/At2g04039	Q93Z84	chain_52889	-2.47	46.56	76.32
F18B13.24 protein	Q9SSC3	NODE_10005	-2.4636	65.69	68.32
Polyubiquitin 8	Q39256	chain_61762	-2.4607	38.24	78.21
Myelin-associated oligodendrocyte basic protein	Q8RWI0	chain_2046	-2.4605	79.75	80.0
Glyceraldehyde-3-phosphate dehydrogenase GAPB, chloroplastic	P25857	chain_50144	-2.4593	48.18	80.77
Cellulose synthase A catalytic subunit 3 [UDP-forming]	Q941L0	chain_2520	-2.4565	38.3	100.0
Chlorophyll a-b binding protein 3, chloroplastic	Q9S7M0	chain_64182	-2.4565	60.87	96.3
Photosystem I reaction center subunit N, chloroplastic	P49107	chain_45533	-2.452	84.06	92.98

continued on the next page

Contigs repressed at 34°C (*continued*)

Name	UniProt ID	Contig	$\log_2$ (fold-Change)	Target coverage (%)	Alignment identity (%)
30S ribosomal protein S10, chloroplastic	Q9LK61	NODE_63739	-2.4512	79.59	89.47
BTB/POZ domain-containing protein At4g08455	Q6DBN1	chain_17146	-2.4509	62.82	79.17
ADP-ribosylation factor A1E	Q9M1P5	chain_26753	-2.4463	36.73	94.12
Alcohol dehydrogenase class-P	P06525	chain_9345	-2.4444	94.32	81.21
At1g52870/F14G24_14	Q9C933	chain_3536	-2.443	62.86	86.31
Photosystem I reaction center subunit XI, chloroplastic	Q9SUI4	chain_25657	-2.4419	79.61	85.19
RNA polymerase sigma factor sigD, chloroplastic	Q9ZSL6	chain_5318	-2.4408	75.62	65.57
Protochlorophyllide reductase A, chloroplastic	Q42536	chain_8181	-2.4406	100.0	92.35
NADPH-dependent alkenal/one oxidoreductase, chloroplastic	Q9ZUC1	chain_45561	-2.4405	88.37	87.83
Zeaxanthin epoxidase, chloroplastic	Q9FGC7	chain_46133	-2.4367	89.76	73.85
Cytochrome b6-f complex iron-sulfur subunit, chloroplastic	Q9ZR03	chain_335	-2.4303	98.04	92.86
Elongation factor G, chloroplastic	Q9SI75	chain_14773	-2.43	100	91.15
At1g07200	Q8RWE4	chain_5911	-2.4298	19.55	58.82
At2g26500	O48717	chain_55759	-2.426	89.19	87.5
Ribulose bisphosphate carboxylase/oxygenase activase, chloroplastic	P10896	chain_9915	-2.426	56.52	84.0
DHHA1 domain protein	F4HRG2	chain_7445	-2.4257	94.85	74.6
Transcription factor PERIANTHIA	Q9SX27	chain_45529	-2.4244	92.16	87.1
Ribulose bisphosphate carboxylase/oxygenase activase, chloroplastic	P10896	NODE_53893	-2.4227	60.0	72.34
Putative elongation factor TypA-like SVR3, chloroplastic	F4K410	chain_4205	-2.4085	84.44	89.18
Receptor-like serine/threonine-protein kinase SD1-6	Q9S972	chain_10025	-2.4075	69.48	59.79
Ferric reduction oxidase 5	Q9FLW2	NODE_9254	-2.4022	98.99	74.86
Glutamate–glyoxylate aminotransferase 2	Q9S7E9	chain_70232	-2.3991	66.2	84.95
Ribulose bisphosphate carboxylase small chain 3B, chloroplastic	P10798	chain_39726	-2.3965	29.35	84.62
(S)-2-hydroxy-acid oxidase GLO2	Q9LRS0	NODE_37020	-2.3959	100	87.78
Photosystem I reaction center subunit III, chloroplastic	Q9SHE8	chain_62146	-2.3947	55.32	88.0

continued on the next page

Contigs repressed at 34°C (*continued*)

Name	UniProt ID	Contig	$\log_2$ (fold-Change)	Target coverage (%)	Alignment identity (%)
ATP synthase subunit O, mitochondrial	Q96251	chain_68676	-2.393	98.08	72.0
Oxygen-evolving enhancer protein 2-1, chloroplastic	Q42029	chain_47856	-2.3918	59.18	75.0
Serine–glyoxylate aminotransferase	Q56YA5	chain_41700	-2.3915	95.6	88.74
Autophagy-related protein 8e	Q8S926	chain_54060	-2.3899	26.58	85.0
F6D8.24 protein	Q9SSQ6	chain_57845	-2.388	75.56	63.64
Oxygen-evolving enhancer protein 1-2, chloroplastic	Q9S841	chain_50696	-2.3866	45.63	63.04
Ribulose bisphosphate carboxylase small chain 3B, chloroplastic	P10798	chain_41564	-2.3817	66.35	79.41
ATP synthase subunit delta, chloroplastic	Q9SSS9	chain_41578	-2.3804	38.64	87.88
Sugar transport protein 1	P23586	chain_10464	-2.3767	82.26	67.28
Cytochrome P450 97B3, chloroplastic	O23365	NODE_23273	-2.3614	41.94	81.25
Fructose-bisphosphate aldolase 3, chloroplastic	Q9ZU52	chain_67533	-2.3608	60.56	88.1
Palmitoyl-monogalactosyldiacylglycerol delta-7 desaturase, chloroplastic	Q949X0	chain_7019	-2.3602	69.29	75.46
Photosystem I reaction center subunit IV B, chloroplastic	Q9S714	chain_39501	-2.3582	92.06	85.96
Photosystem I reaction center subunit II-2, chloroplastic	Q9SA56	NODE_50416	-2.3569	100.0	87.65
Thioredoxin family protein	F4JKW0	NODE_3114	-2.3561	70.21	77.69
ABC transporter B family member 9	Q9M0M2	chain_12420	-2.3554	78.22	72.01
Lycopene epsilon cyclase, chloroplastic	Q38932	chain_4389	-2.3459	88.6	82.74
TraB family protein	Q9FJ89	NODE_6088	-2.3458	89.82	74.5
Chlorophyll a-b binding protein, chloroplastic	A0A1I9LMB4	chain_27319	-2.3454	49.15	89.29
G-type lectin S-receptor-like serine/threonine-protein kinase SD1-1	O81833	chain_34894	-2.3454	25.0	86.96
Protein NSP-INTERACTING KINASE 2	Q8RY65	NODE_4873	-2.3447	96.71	83.82
G-type lectin S-receptor-like serine/threonine-protein kinase At1g11330	Q9SXB8	chain_13061	-2.3406	85.41	67.05
Pentatricopeptide repeat-containing protein At5g25630	Q8GZ63	NODE_592	-2.3396	73.04	65.44
Lysine-tRNA ligase	Q8RWI4	chain_5286	-2.3396	96.62	88.94
Photosystem I reaction center subunit II-2, chloroplastic	Q9SA56	chain_56947	-2.3371	66.67	96.0

continued on the next page

Contigs repressed at 34°C (*continued*)

Name	UniProt ID	Contig	$\log_2$ (fold-Change)	Target coverage (%)	Alignment identity (%)
Ribulose bisphosphate carboxylase small chain 1A, chloroplastic	P10795	chain_73	-2.3362	72.6	79.81
Glycine dehydrogenase (decarboxylating) 1, mitochondrial	Q94B78	chain_26671	-2.3346	60.0	84.21
Glutamate receptor 3.3	Q9C8E7	NODE_5367	-2.3345	97.46	79.82
ACT domain-containing protein ACR10	Q9SJM1	chain_7926	-2.3342	91.69	73.54
Ribulose bisphosphate carboxylase small chain 1A, chloroplastic	P10795	chain_70173	-2.3319	32.79	89.47
F19K23.17 protein	O04589	chain_9476	-2.3312	84.09	76.09
At4g24810	Q67Y06	chain_14472	-2.3301	84.86	76.89
Thiamine thiazole synthase, chloroplastic	Q38814	chain_26574	-2.3297	92.77	82.89
Ribulose bisphosphate carboxylase small chain 3B, chloroplastic	P10798	chain_36955	-2.3287	95.83	89.74
Serine hydroxymethyltransferase 1, mitochondrial	Q9SZJ5	chain_55916	-2.3282	94.2	92.19
Plastocyanin major isoform, chloroplastic	P42699	chain_47751	-2.3281	53.54	67.31
AP2/ERF and B3 domain-containing transcription factor RAV1	Q9ZWM9	chain_7480	-2.3271	68.78	87.73
Fructose-1,6-bisphosphatase 1, chloroplastic	P25851	chain_12840	-2.3237	91.33	90.45
Protein LURP-one-related 5	Q9SSC7	chain_69003	-2.3223	79.61	84.03
G-type lectin S-receptor-like serine/threonine-protein kinase SD2-2	Q39203	chain_1306	-2.3211	11.46	60.71
Photosystem II 22 kDa protein, chloroplastic	Q9XF91	chain_51992	-2.3118	46.15	84.62
Omega-hydroxypalmitate O-feruloyl transferase	Q94CD1	chain_53104	-2.3076	95.0	73.9
29 kDa ribonucleoprotein, chloroplastic	Q43349	chain_1301	-2.3016	41.79	79.45
Receptor-like protein kinase THESEUS 1	Q9LK35	chain_24726	-2.3001	93.92	91.8
Glutamine synthetase cytosolic isozyme 1-3	Q9LVI8	chain_25442	-2.2996	51.16	90.48
Elongation factor Tu, chloroplastic	P17745	chain_38189	-2.2993	41.07	81.82
Sodium-dependent phosphate transport protein 1, chloroplastic	O82390	chain_4451	-2.2977	82.92	88.32
Beta carbonic anhydrase 1, chloroplastic	P27140	chain_62735	-2.2971	39.33	79.41
ATP-citrate synthase alpha chain protein 1	Q9SGY2	chain_59356	-2.2971	35.94	95.45
Alanine–tRNA ligase, chloroplastic/mitochondrial	Q9FFC7	NODE_7525	-2.2958	99.6	80.0
Probable choline kinase 1	Q9M9H6	chain_1734	-2.2958	90.14	71.66

continued on the next page

Contigs repressed at 34°C (*continued*)

Name	UniProt ID	Contig	$\log_2$ (fold-Change)	Target coverage (%)	Alignment identity (%)
NAD(P)H-quinone oxidoreductase subunit M, chloroplastic	Q2V2S7	chain_53245	-2.2949	61.73	82.22
Glycine dehydrogenase (decarboxylating) 2, mitochondrial	O80988	chain_4001	-2.294	65.07	80.46
Serine/threonine-protein kinase STN8, chloroplastic	Q9LZV4	NODE_2064	-2.2935	64.9	76.92
Chlorophyll a-b binding protein, chloroplastic	F4K8I1	chain_25631	-2.2899	52.27	90.91
Cytokinin dehydrogenase 7	Q9FUJ1	NODE_6079	-2.2887	95.4	72.93
F18B13.21 protein	Q9SSC6	NODE_5101	-2.2857	66.3	61.67
Ribulose bisphosphate carboxylase/oxygenase activase, chloroplastic	P10896	chain_59594	-2.2837	69.23	100.0
Ferredoxin-thioredoxin reductase catalytic chain, chloroplastic	Q9SJ89	chain_7848	-2.2825	80.67	84.17
Ribulose bisphosphate carboxylase/oxygenase activase, chloroplastic	P10896	NODE_61594	-2.2754	52.7	78.95
F19K23.17 protein	O04589	chain_5443	-2.2726	97.46	77.49
[Pyruvate dehydrogenase (acetyl-transferring)] kinase, mitochondrial	Q9SBJ1	chain_4501	-2.2709	99.5	88.08
Protease Do-like 1, chloroplastic	O22609	NODE_5091	-2.2703	99.18	88.33
Ribulose bisphosphate carboxylase small chain 3B, chloroplastic	P10798	NODE_72500	-2.2681	56.25	84.62
Sodium/calcium exchanger NCL	Q8L636	chain_8668	-2.2654	89.26	70.9
Serine hydroxymethyltransferase 1, mitochondrial	Q9SZJ5	chain_10083	-2.2639	87.74	81.52
Photosynthetic NDH subunit of subcomplex B 1, chloroplastic	Q9S9N6	chain_1935	-2.2622	89.08	79.56
Probable plastid-lipid-associated protein 12, chloroplastic	Q8LAP6	chain_3165	-2.2613	92.34	80.1
Kinesin-like protein KIN-14P	Q0WN69	chain_57137	-2.2609	88.0	85.71
Sedoheptulose-1,7-bisphosphatase, chloroplastic	P46283	chain_26069	-2.2596	98.86	92.44
Plastocyanin	A0A1P8APR2	chain_61955	-2.2561	52.63	79.49
Fructose-bisphosphate aldolase 6, cytosolic	Q9SJQ9	chain_43781	-2.2557	63.86	82.03
Ribulose bisphosphate carboxylase small chain	F4KA76	chain_62487	-2.2513	76.0	72.97
Photosystem I subunit O	Q949Q5	chain_65958	-2.2507	60.27	83.72
Elongation factor G, chloroplastic	Q9SI75	chain_586	-2.2433	100	91.15

continued on the next page

Contigs repressed at 34°C (*continued*)

Name	UniProt ID	Contig	$\log_2$ (fold-Change)	Target coverage (%)	Alignment identity (%)
Glutamine synthetase cytosolic isozyme 1-1	Q56WN1	chain_49687	-2.2413	73.33	85.71
Thioredoxin M4, chloroplastic	Q9SEU6	chain_48773	-2.2394	84.54	66.67
Thiamine thiazole synthase, chloroplastic	Q38814	chain_45012	-2.2383	90.43	94.17
Probable plastid-lipid-associated protein 11, chloroplastic	O81304	chain_5182	-2.2335	93.65	75.88
AT5g48790/K24G6_12	Q94F50	NODE_7168	-2.2328	96.77	86.55
Photosynthetic NDH subunit of lumenal location 1, chloroplastic	O80634	chain_5076	-2.2319	56.62	77.24
ATP synthase gamma chain 2, chloroplastic	Q01909	chain_47187	-2.2315	51.92	88.46
Photosystem I reaction center subunit V, chloroplastic	Q9S7N7	chain_40008	-2.231	27.59	93.55
Vacuolar cation/proton exchanger 1	Q39253	chain_70770	-2.2294	88.06	77.92
Photosystem I chlorophyll a/b-binding protein 2, chloroplastic	Q9SYW8	chain_51352	-2.2288	52.44	90.48
Sedoheptulose-1,7-bisphosphatase, chloroplastic	P46283	chain_2036	-2.2273	89.29	92.93
Ribulose bisphosphate carboxylase small chain 3B, chloroplastic	P10798	chain_47262	-2.2265	39.24	85.19
Phosphoribulokinase, chloroplastic	P25697	chain_55992	-2.2236	32.84	95.24
Pyruvate, phosphate dikinase 1, chloroplastic	O23404	chain_63952	-2.2227	99.55	84.37
Cytochrome b6-f complex iron-sulfur subunit, chloroplastic	Q9ZR03	NODE_48930	-2.2213	87.95	90.14
Leucine-rich repeat protein kinase family protein	Q9SN80	chain_5933	-2.2213	78.57	71.88
Chlorophyll a-b binding protein 4, chloroplastic	P27521	chain_1402	-2.2207	78.57	62.79
ABC transporter C family member 3	Q9LK64	chain_55003	-2.2195	99.51	80.44
Plastidal glycolate/glycerate translocator 1, chloroplastic	Q9FVQ4	chain_2436	-2.2183	88.32	95.0
Protein DETOXIFICATION 27	Q9FKQ1	NODE_4138	-2.215	100.0	75.42
Ribulose bisphosphate carboxylase small chain 1A, chloroplastic	P10795	NODE_66352	-2.2127	35.48	90.48
Chlorophyll a-b binding protein, chloroplastic	A0A1I9LMB4	chain_51544	-2.202	41.67	86.21
AT4g28030/T13J8_140	Q94AC8	chain_1008	-2.2018	90.76	75.35
ATP sulfurylase 4, chloroplastic	Q9S7D8	NODE_1843	-2.2007	80.71	79.89
Photosystem I reaction center subunit N, chloroplastic	P49107	chain_63425	-2.2005	78.26	92.45
Ribulose bisphosphate carboxylase small chain 3B, chloroplastic	P10798	chain_66277	-2.2002	60.0	84.62

continued on the next page

Contigs repressed at 34°C (*continued*)

Name	UniProt ID	Contig	$\log_2$ (fold-Change)	Target coverage (%)	Alignment identity (%)
Fructose-bisphosphate aldolase 3, chloroplastic	Q9ZU52	chain_29732	-2.1996	61.54	78.26
Protein DETOXIFICATION 31	Q9LPV4	chain_41102	-2.198	95.16	75.13
30S ribosomal protein S20, chloroplastic	Q9ASV6	chain_68875	-2.1958	45.37	77.08
ATP synthase gamma chain 1, chloroplastic	Q01908	chain_64094	-2.1927	47.73	85.0
ABC transporter F family member 5	Q9LV93	chain_3543	-2.1915	86.18	83.81
Probable pectinesterase/pectinesterase inhibitor 44	Q9SMY7	chain_5242	-2.191	89.8	76.56
Chlorophyll a-b binding protein, chloroplastic	Q39142	chain_2992	-2.1897	53.01	93.02
Myelin-associated oligodendrocyte basic protein	Q8RWI0	chain_5586	-2.1868	85.01	80.68
Putative 60S ribosomal protein L13-2	Q9SMT4	chain_4293	-2.1856	94.36	77.6
AT4g12830/T20K18_180	Q93ZN4	NODE_4352	-2.1808	95.39	78.38
Xanthoxin dehydrogenase	Q9C826	chain_5287	-2.1804	91.0	77.31
Chlorophyll a-b binding protein 2.4, chloroplastic	Q9XF87	chain_49278	-2.1764	45.95	90.91
Methylcrotonoyl-CoA carboxylase beta chain, mitochondrial	Q9LDD8	chain_43990	-2.1753	36.23	75.0
Fructose-1,6-bisphosphatase, cytosolic	Q9MA79	chain_8045	-2.1711	86.02	78.48
Sedoheptulose-1,7-bisphosphatase, chloroplastic	P46283	chain_21746	-2.1693	81.82	91.43
Chloroplast stem-loop binding protein of 41 kDa b, chloroplastic	Q9SA52	chain_1623	-2.1688	99.48	85.71
Oxygen-evolving enhancer protein 3-2, chloroplastic	Q41932	chain_35100	-2.1687	66.02	77.61
Ribulose bisphosphate carboxylase/oxygenase activase, chloroplastic	P10896	chain_68771	-2.1671	34.78	80.65
Ribulose bisphosphate carboxylase small chain 1A, chloroplastic	P10795	chain_49909	-2.1667	70.33	82.46
Mediator of RNA polymerase II transcription subunit 37a	Q9LKR3	chain_12760	-2.1587	42.11	79.49
S-adenosylmethionine decarboxylase proenzyme 3	Q9LSU6	chain_44996	-2.1584	51.22	90.0
Ribosome-recycling factor, chloroplastic	Q9M1X0	NODE_18759	-2.1582	39.66	90.91
Membrane metalloprotease ARASP, chloroplastic	O80885	NODE_41322	-2.1524	84.62	65.63
Protein CURVATURE THYLAKOID 1B, chloroplastic	Q8LCA1	chain_14359	-2.1519	68.88	72.44
Plastocyanin minor isoform, chloroplastic	P11490	chain_62220	-2.1507	35.21	75.0

continued on the next page

Contigs repressed at 34°C (*continued*)

Name	UniProt ID	Contig	$\log_2$ (fold-Change)	Target coverage (%)	Alignment identity (%)
Carboxyl-terminal-processing peptidase 1, chloroplastic	F4KHG6	chain_11219	-2.1485	86.51	77.78
Proline dehydrogenase 1, mitochondrial	P92983	NODE_2315	-2.1469	87.17	65.66
At2g25605	Q6NM03	chain_4879	-2.1386	93.62	88.51
Ribulose bisphosphate carboxylase small chain 3B, chloroplastic	P10798	chain_59756	-2.1369	65.63	85.37
AT3g29240/MXO21_9	Q9LS71	NODE_7112	-2.1336	80.0	72.38
Phosphoglycerate kinase 1, chloroplastic	Q9LD57	chain_550	-2.1332	100	86.81
Calcium sensing receptor, chloroplastic	Q9FN48	chain_3134	-2.1326	84.76	80.68
AT1G67910 protein	Q9C9V7	chain_52640	-2.1323	29.03	76.92
Protein SUPPRESSOR OF QUENCHING 1, chloroplastic	Q8VZ10	chain_5497	-2.1264	87.55	79.9
Alpha/beta-Hydrolases superfamily protein	Q9FFW9	chain_3499	-2.1262	73.83	76.21
Photosystem I reaction center subunit II-2, chloroplastic	Q9SA56	chain_70109	-2.1258	86.36	92.04
Trifunctional UDP-glucose 4,6-dehydratase/UDP-4-keto-6-deoxy-D-glucose 3,5-epimerase/UDP-4-keto-L-rhamnose-reductase RHM1	Q9SYM5	NODE_67705	-2.1248	90.32	88.89
Photosystem I chlorophyll a/b-binding protein 2, chloroplastic	Q9SYW8	chain_54781	-2.1231	100.0	94.0
Aluminum-activated malate transporter 9	Q9LS46	chain_9415	-2.1222	32.18	62.77
Fructose-1,6-bisphosphatase, cytosolic	Q9MA79	NODE_30012	-2.1217	50.54	89.13
Pentatricopeptide repeat-containing protein At3g04760, chloroplastic	Q9SR00	chain_12056	-2.1208	90.25	73.75
Thiamine thiazole synthase, chloroplastic	Q38814	chain_59576	-2.1182	36.84	100.0
Light-harvesting complex-like protein OHP1, chloroplastic	O81208	chain_27042	-2.1168	71.05	84.62
Cytochrome P450	Q9LUD3	chain_4475	-2.1108	93.66	66.0
DUF21 domain-containing protein At2g14520	Q9ZQR4	chain_1184	-2.1091	94.94	72.31
Photosystem I chlorophyll a/b-binding protein 5, chloroplastic	Q9C639	NODE_6426	-2.1071	77.29	82.86
Ubiquitin carboxyl-terminal hydrolase 8	Q9C585	chain_1364	-2.1068	71.08	78.43
Thiamine thiazole synthase, chloroplastic	Q38814	chain_24433	-2.1038	89.91	95.88
DEAD-box ATP-dependent RNA helicase 22	Q944S1	chain_6472	-2.1029	84.87	71.09

continued on the next page

Contigs repressed at 34°C (*continued*)

Name	UniProt ID	Contig	$\log_2$ (fold-Change)	Target coverage (%)	Alignment identity (%)
Protein CURVATURE THYLAKOID 1B, chloroplastic	Q8LCA1	NODE_38153	-2.1016	51.02	81.63
At5g17570	A2RVM4	NODE_15867	-2.0993	81.51	70.65
At5g49760	Q8GZ99	chain_13867	-2.0988	93.18	72.36
Zinc finger CCCH domain-containing protein 67	Q5RJC5	chain_2039	-2.0982	60.35	72.34
At5g23440	Q9FHL4	chain_1802	-2.0981	51.34	66.32
Potassium transporter 6	Q8W4I4	chain_50918	-2.0981	95.75	83.51
NAD(P)-linked oxidoreductase superfamily protein	Q949S6	chain_35080	-2.0979	71.51	82.68
Probable aldo-keto reductase 3	O22707	NODE_49106	-2.0968	44.0	80.95
Chlorophyll a-b binding protein CP26, chloroplastic	Q9XF89	chain_38157	-2.0947	56.82	93.88
Photosystem I chlorophyll a/b-binding protein 2, chloroplastic	Q9SYW8	NODE_58186	-2.0936	52.38	85.71
3-oxo-Delta(4,5)-steroid 5-beta-reductase	Q9STX2	chain_4927	-2.0936	24.55	61.29
ABC transporter C family member 9	Q9M1C7	NODE_3656	-2.0925	95.0	79.3
Delta(12)-fatty-acid desaturase	P46313	NODE_6883	-2.0861	75.06	80.14
Glutathione reductase, chloroplastic	P42770	chain_3027	-2.0835	92.07	84.67
Leucine-rich repeat transmembrane protein kinase protein	Q9SZV2	chain_3463	-2.083	62.5	67.68
At1g36070	Q0V7U5	chain_2054	-2.0826	75.7	74.59
Oxygen-evolving enhancer protein 3-1, chloroplastic	Q9XFT3	chain_232	-2.0817	85.26	71.25
LOW PSII ACCUMULATION-like protein	F4JM22	NODE_4889	-2.0813	85.38	75.25
Leucine-rich repeat protein kinase family protein	F4KGL1	chain_6462	-2.0785	76.33	75.63
Ribulose bisphosphate carboxylase small chain 3B, chloroplastic	P10798	chain_44561	-2.0764	41.51	95.24
Photosystem I chlorophyll a/b-binding protein 2, chloroplastic	Q9SYW8	chain_56489	-2.0763	54.68	91.03
Oxygen-evolving enhancer protein 1-2, chloroplastic	Q9S841	chain_101	-2.0725	55.88	75.68
Protein NBR1 homolog	Q9SB64	chain_58191	-2.0717	44.26	80.77
Photosynthetic NDH subunit of lumenal location 4, chloroplastic	Q9SCY3	chain_6539	-2.0704	68.67	77.99
Probable protein phosphatase 2C 76	Q94AT1	chain_8509	-2.07	47.83	65.63
Protein LOW PSII ACCUMULATION 3, chloroplastic	Q8H0W0	chain_6561	-2.0697	98.25	82.51

continued on the next page

Contigs repressed at 34°C (*continued*)

Name	UniProt ID	Contig	$\log_2$ (fold-Change)	Target coverage (%)	Alignment identity (%)
Beta carbonic anhydrase 2, chloroplastic	P42737	chain_54339	-2.0692	72.31	76.09
CF9 protein	Q9SLU2	NODE_5738	-2.0686	96.84	62.9
Photosystem I reaction center subunit XI, chloroplastic	Q9SUI4	chain_15528	-2.0673	60.36	87.88
YlmG homolog protein 2, chloroplastic	Q9C595	NODE_14497	-2.0664	53.09	82.69
Rhodanese-like domain-containing protein 15, chloroplastic	Q38853	chain_2972	-2.0658	84.13	76.92
Photosystem I reaction center subunit VI-2, chloroplastic	Q9SUI6	chain_22590	-2.0631	48.91	79.07
Photosystem I chlorophyll a/b-binding protein 2, chloroplastic	Q9SYW8	chain_69533	-2.0628	40.63	90.2
Guanosine nucleotide diphosphate dissociation inhibitor At5g09550	Q9LXC0	NODE_2936	-2.0617	98.47	84.44
Photosystem I chlorophyll a/b-binding protein 3-1, chloroplastic	Q9SY97	chain_48594	-2.0615	72.5	100.0
Photosynthetic NDH subunit of lumenal location 3, chloroplastic	Q9SGH4	chain_3633	-2.0596	64.07	77.37
Ferredoxin C 2, chloroplastic	Q9C7Y4	chain_5013	-2.0588	60.0	91.2
Triacylglycerol lipase 2	Q67ZU1	chain_68915	-2.0579	84.29	61.02
Purine-uracil permease NCS1	Q9LZD0	NODE_3610	-2.0564	91.57	74.37
Cytochrome P450 71A26	Q9STK7	chain_55173	-2.0557	96.43	58.55
Glyceraldehyde-3-phosphate dehydrogenase GAPA2, chloroplastic	Q9LPW0	chain_55473	-2.0542	54.72	100.0
Cytokinin riboside 5'-monophosphate phosphoribohydrolase LOG5	Q8LBB7	NODE_6655	-2.0539	80.08	85.1
Two-component response regulator-like APRR3	Q9LVG4	NODE_1012	-2.0499	26.28	69.62
Oxygen-evolving enhancer protein 1-2, chloroplastic	Q9S841	chain_5922	-2.0459	23.46	88.89
AT3G13950 protein	B9DG91	chain_9741	-2.0453	13.33	62.86
Magnesium transporter MRS2-11, chloroplastic	Q058N4	chain_28727	-2.0445	67.49	84.66
Transketolase-2, chloroplastic	F4IW47	chain_10741	-2.0403	91.18	85.41
Rac-like GTP-binding protein ARAC8	Q9SU67	chain_7756	-2.0403	88.57	88.31
Phosphoglycerate mutase-like protein AT74	Q9MAA2	NODE_4776	-2.0375	90.95	62.65
Peptidyl-prolyl cis-trans isomerase FKBP16-4, chloroplastic	Q9SR70	chain_29595	-2.0354	80.0	87.23
UDP-glucuronic acid decarboxylase 1	F4JAG3	chain_5391	-2.0346	33.33	73.08
Translation initiation factor IF-1, chloroplastic	O82499	NODE_14319	-2.0328	48.76	77.59

continued on the next page

Contigs repressed at 34°C (*continued*)

Name	UniProt ID	Contig	$\log_2$ (fold-Change)	Target coverage (%)	Alignment identity (%)
UDP-rhamnose/UDP-galactose transporter 4	Q8RWW7	chain_9952	-2.0326	93.47	84.08
Photosystem I reaction center subunit II-2, chloroplastic	Q9SA56	chain_32732	-2.0311	100.0	95.56
Probable pectinesterase/pectinesterase inhibitor 32	Q9LXK7	chain_48604	-2.0281	99.66	59.85
Post-illumination chlorophyll fluorescence increase	Q9LVZ5	chain_705	-2.0271	98.25	80.0
Violaxanthin de-epoxidase, chloroplastic	Q39249	NODE_2118	-2.027	86.45	82.17
Chlorophyll a-b binding protein CP26, chloroplastic	Q9XF89	NODE_39135	-2.0261	50.0	93.94
Serine carboxypeptidase-like 34	Q0WPR4	chain_53857	-2.0252	97.76	70.71
Cytochrome P450, family 89, subfamily A, polypeptide 3	F4K231	chain_51919	-2.0243	64.62	70.75
Carboxyl-terminal-processing peptidase 2, chloroplastic	O23614	NODE_294	-2.021	85.03	83.38
Probable plastid-lipid-associated protein 14, chloroplastic	Q9LV04	chain_6676	-2.0172	90.91	76.14
Uncharacterized protein At5g42070	Q8RWR9	chain_48681	-2.017	32.67	81.25
Leucine-rich repeat transmembrane protein kinase protein	Q9SZV2	NODE_5453	-2.0155	84.47	59.74
Probable protein phosphatase 2C 59	Q8RXV3	NODE_20790	-2.0148	34.55	100.0
Fructose-1,6-bisphosphatase 1, chloroplastic	P25851	chain_1554	-2.0128	72.12	85.49
Cysteine-rich RLK (RECEPTOR-like protein kinase) 16	A0A1P8B4Z8	chain_9427	-2.01	92.74	61.92
Adenine nucleotide transporter BT1, chloroplastic/mitochondrial	Q9SUV1	NODE_3917	-2.0094	70.64	78.09
Thiamine thiazole synthase, chloroplastic	Q38814	chain_9777	-2.0071	96.43	91.74
Thioredoxin-like protein AAED1, chloroplastic	Q9ZUU2	chain_14705	-2.0064	48.43	83.09
PP2A regulatory subunit TAP46	Q8LDQ4	chain_6488	-2.0004	100	69.52
Potassium transporter	Q9LYL4	chain_1173	-1.9996	70.66	82.05
Protein CHUP1, chloroplastic	Q9LI74	chain_4197	-1.9993	90.98	89.17
AT4g34150/F28A23_90	Q945K9	chain_3479	-1.9973	79.03	76.29
Uncharacterized protein At4g15545	Q93W28	chain_2548	-1.9966	56.31	75.44
Thioredoxin-like 2-1, chloroplastic	Q8LEK4	NODE_5519	-1.996	60.44	77.27
Glutamine synthetase	F4ID91	NODE_22817	-1.9947	47.06	95.65
Zinc knuckle (CCHC-type) family protein	F4K466	NODE_6090	-1.9928	83.54	74.81

continued on the next page

Contigs repressed at 34°C (*continued*)

Name	UniProt ID	Contig	$\log_2$ (fold-Change)	Target coverage (%)	Alignment identity (%)
Glucose-6-phosphate 1-dehydrogenase 4, chloroplastic	Q93ZW0	NODE_2568	-1.9925	88.84	82.35
Serine/threonine-protein kinase STN7, chloroplastic	Q9S713	chain_48465	-1.9856	75.14	86.52
Topless-related protein 2	Q9LRZ0	NODE_32111	-1.9849	90.91	89.83
AT3g13460/MRP15_10	Q9LJE5	chain_57809	-1.9837	57.6	70.81
Glucose-1-phosphate adenylyltransferase large subunit 1, chloroplastic	P55229	chain_66801	-1.9773	74.47	82.35
Oxygen-evolving enhancer protein 2-1, chloroplastic	Q42029	chain_38239	-1.9748	72.29	72.88
ATP synthase gamma chain 1, chloroplastic	Q01908	NODE_33370	-1.9736	100	83.78
Thioredoxin F2, chloroplastic	Q9XFH9	chain_58800	-1.9734	98.85	83.53
Germin-like protein subfamily 3 member 3	P94072	chain_21099	-1.9728	95.95	71.52
Photosystem I reaction center subunit IV A, chloroplastic	Q9S831	chain_52752	-1.9691	44.07	96.0
O-fucosyltransferase 29	Q8LPF8	chain_9086	-1.9675	82.12	81.78
Glucose-1-phosphate adenylyltransferase small subunit, chloroplastic	P55228	NODE_23637	-1.9657	40.43	94.44
Sugar transport protein 13	Q94AZ2	chain_3151	-1.9634	96.32	82.22
AT3G27110 protein	Q9LSC4	chain_5853	-1.9627	80.06	84.42
Chlorophyll a-b binding protein CP26, chloroplastic	Q9XF89	chain_63221	-1.9607	60.42	85.71
Oxysterol-binding protein-related protein 4B	Q9SW00	chain_37381	-1.9606	53.45	66.67
D-3-phosphoglycerate dehydrogenase 3, chloroplastic	Q9LT69	chain_11315	-1.9603	82.66	86.62
Thiamine thiazole synthase, chloroplastic	Q38814	chain_6698	-1.9523	89.91	95.88
Hypersensitive-induced response protein 1	Q9FM19	chain_1304	-1.9507	98.27	85.87
Pentatricopeptide repeat-containing protein At5g25630	Q8GZ63	chain_3287	-1.949	67.84	68.19
Rieske (2Fe-2S) domain-containing protein	Q9C9I7	chain_1269	-1.9479	79.59	84.42
Proteinaceous RNase P 1, chloroplastic/mitochondrial	Q66GI4	chain_11598	-1.9436	71.38	66.0
50S ribosomal protein L31, chloroplastic	Q9FWS4	chain_41850	-1.9415	42.86	84.21
Putative 60S ribosomal protein L13-2	Q9SMT4	chain_34910	-1.9401	94.36	77.6
K+ efflux antiporter 2	F4JHE9	chain_18132	-1.9376	92.31	88.31
DHHA1 domain protein	F4HRG2	chain_2327	-1.9369	94.85	74.6

continued on the next page

Contigs repressed at 34°C (*continued*)

Name	UniProt ID	Contig	$\log_2$ (fold-Change)	Target coverage (%)	Alignment identity (%)
Putative 60S ribosomal protein L13-2	Q9SMT4	chain_693	-1.9351	94.36	77.6
Ribulose bisphosphate carboxylase/oxygenase activase, chloroplastic	P10896	NODE_26230	-1.9348	76.32	82.14
50S ribosomal protein L12-1, chloroplastic	P36210	chain_47008	-1.9344	23.01	76.0
AT1G67910 protein	Q9C9V7	chain_675	-1.9342	31.03	76.92
Uncharacterized protein	A8MS05	chain_3198	-1.9327	41.61	69.09
AT4g32260/F10M6_100	Q42139	chain_28075	-1.9301	78.38	79.13
Protein KINESIN LIGHT CHAIN-RELATED 2	Q9LII8	chain_7669	-1.9291	86.59	80.0
AT4g35250/F23E12_190	O65502	NODE_4159	-1.929	90.08	88.94
ATP synthase subunit delta, chloroplastic	Q9SSS9	chain_64683	-1.9271	89.47	72.73
Phytochrome-interacting ankyrin-repeat protein 2	Q9FNP4	chain_1207	-1.9241	82.76	77.89
F-box/kelch-repeat protein At1g67480	Q9CAG8	NODE_3111	-1.9232	97.21	68.75
At1g09870/F21M12_26	Q941B2	chain_17625	-1.923	35.79	63.64
Membrane metalloprotease ARASP, chloroplastic	O80885	NODE_2583	-1.9225	100.0	91.49
Haloacid dehalogenase-like hydrolase domain-containing protein At4g39970	Q680K2	chain_17890	-1.9221	100	90.91
P-loop containing nucleoside triphosphate hydrolases superfamily protein	F4K1G7	chain_5251	-1.9207	99.51	84.84
Oxygen-evolving enhancer protein 2-1, chloroplastic	Q42029	chain_62355	-1.9204	75.9	72.58
Serine/threonine-protein kinase VPS15	Q9M0E5	chain_63832	-1.92	39.8	94.74
Serine/threonine-protein kinase SRK2H	Q9FFP9	chain_53241	-1.9182	54.55	75.86
Glycine cleavage system H protein 3, mitochondrial	Q9LQL0	chain_560	-1.9176	78.11	83.97
Photosystem I chlorophyll a/b-binding protein 5, chloroplastic	Q9C639	NODE_54362	-1.9145	88.24	72.41
Protein WEAK CHLOROPLAST MOVEMENT UNDER BLUE LIGHT 1	O48724	chain_9502	-1.9145	61.68	78.66
F-box protein PP2-A12	Q9LN77	chain_7517	-1.9138	91.61	75.89
Major facilitator superfamily protein	F4IMD7	NODE_5752	-1.9075	15.11	56.52
Alpha/beta-Hydrolases superfamily protein	Q9FN84	chain_1237	-1.9022	87.78	83.66
Calmodulin-binding transcription activator 3	Q8GSA7	chain_6736	-1.8952	35.31	70.18

continued on the next page

Contigs repressed at 34°C (*continued*)

Name	UniProt ID	Contig	$\log_2$ (fold-Change)	Target coverage (%)	Alignment identity (%)
50S ribosomal protein L9, chloroplastic	P25864	chain_5101	-1.8932	82.95	83.96
Ubiquitin-specific protease 13	F4J7I2	chain_22442	-1.8811	96.93	93.02
Peptidyl-prolyl cis-trans isomerase FKBP16-4, chloroplastic	Q9SR70	chain_2599	-1.881	56.82	87.84
GPI mannosyltransferase 1	Q500W7	chain_12901	-1.8809	74.75	73.64
Chlorophyll a-b binding protein 6, chloroplastic	Q01667	chain_57280	-1.8807	70.59	90.14
Methionine aminopeptidase 1B, chloroplastic	Q9FV52	chain_3	-1.8804	59.09	86.84
Glyceraldehyde-3-phosphate dehydrogenase GAPA2, chloroplastic	Q9LPW0	chain_66945	-1.8781	46.77	84.21
Thioredoxin superfamily protein	A0A1I9LR27	chain_39701	-1.8778	52.34	89.39
Peroxisomal membrane 22 kDa (Mpv17/PMP22) family protein	Q9LV46	chain_778	-1.8765	85.31	78.39
Fructose-bisphosphate aldolase 3, chloroplastic	Q9ZU52	chain_43182	-1.8757	100	77.89
Phosphatidylserine decarboxylase proenzyme 3	A4GNA8	NODE_74979	-1.8749	62.5	91.67
Protein CHAPERONE-LIKE PROTEIN OF POR1, chloroplastic	Q9FN50	chain_2980	-1.8742	87.5	80.63
Serine–glyoxylate aminotransferase	Q56YA5	chain_33991	-1.8726	96.22	87.28
Starch synthase 3, chloroplastic/amyloplastic	F4IAG2	chain_6409	-1.8723	98.44	80.44
Maltose excess protein 1, chloroplastic	Q9LF50	chain_9688	-1.8716	96.92	73.6
Alpha/beta-Hydrolases superfamily protein	Q94K11	NODE_878	-1.8666	78.55	82.56
Ascorbate oxidase-like protein	Q8LPL3	chain_4359	-1.8659	92.14	72.75
AT5g40850/MHK7_8	Q42606	chain_7504	-1.8648	90.4	84.27
Chlorophyll a-b binding protein 6, chloroplastic	Q01667	chain_54924	-1.8643	45.61	92.0
Photosystem I reaction center subunit XI, chloroplastic	Q9SUI4	chain_796	-1.8635	79.61	85.19
ATP-dependent zinc metalloprotease FTSH 8, chloroplastic	Q8W585	chain_8147	-1.8633	79.85	91.71
Photosystem I reaction center subunit VI-1, chloroplastic	Q9SUI7	chain_67400	-1.8627	67.57	86.49
ABC transporter G family member 31	Q7PC88	chain_3787	-1.8625	93.98	66.04
Rubredoxin family protein	Q9FFJ2	chain_46648	-1.8561	62.35	82.69
Protein PLASTID MOVEMENT IMPAIRED 1-RELATED 1	F4K5K6	chain_19287	-1.8533	87.23	76.54
Peptidyl-prolyl cis-trans isomerase CYP28, chloroplastic	O65220	chain_7261	-1.8529	85.92	80.84

continued on the next page

Contigs repressed at 34°C (*continued*)

Name	UniProt ID	Contig	$\log_2$ (fold-Change)	Target coverage (%)	Alignment identity (%)
COP9 signalosome complex subunit 3	Q8W575	chain_7309	-1.8529	96.88	69.64
Photosystem II reaction center W protein, chloroplastic	Q39194	chain_56596	-1.8479	72.34	82.09
O-fucosyltransferase 9	Q8H1E6	chain_9792	-1.845	87.11	81.55
Multidrug resistance-associated protein 6	A0A1I9LPV3	chain_4920	-1.8448	87.46	71.64
At2g44670/F16B22.16	O80506	chain_43783	-1.8425	12.5	72.97
Proline-rich receptor-like protein kinase PERK4	Q9ZNQ8	NODE_8672	-1.8412	72.32	54.31
Fructose-bisphosphate aldolase 5, cytosolic	O65581	chain_3939	-1.8404	85.29	84.88
At2g27290	Q9XIN6	chain_5674	-1.8391	98.31	94.74
Transcription initiation factor TFIID subunit 1	Q8LRK9	chain_62173	-1.8369	56.73	70.69
At2g43320/T1O24.6	O22847	chain_5541	-1.8367	83.96	81.6
Photosystem I reaction center subunit XI, chloroplastic	Q9SUI4	chain_69203	-1.8359	59.85	84.62
Oxygen-evolving enhancer protein 1-1, chloroplastic	P23321	chain_45696	-1.8319	34.25	83.33
At2g46220/T3F17.13	O82347	chain_1937	-1.8306	80.21	76.32
Probable glutamyl endopeptidase, chloroplastic	Q8VZF3	chain_21954	-1.8306	84.21	85.61
Lysine-specific histone demethylase 1 homolog 1	Q8VXV7	chain_12368	-1.8302	66.55	80.83
Protein REVEILLE 8	Q8RWU3	chain_1813	-1.8234	75.28	81.36
Cytochrome c biogenesis protein family	F4K0A8	chain_5718	-1.8222	67.36	86.49
Magnesium-chelatase subunit ChlH, chloroplastic	Q9FNB0	chain_9611	-1.8194	96.91	86.5
Uncharacterized protein At5g02240	Q94EG6	chain_6242	-1.8189	100	82.83
Acetamidase/Formamidase family protein	Q9SZE9	chain_2949	-1.8171	80.2	89.63
Receptor protein-tyrosine kinase CEPR1	Q9FGL5	NODE_4678	-1.8143	77.75	78.64
At5g49760	Q8GZ99	chain_15285	-1.8143	91.46	73.41
AT1G71480 protein	Q9C9I5	chain_5023	-1.8143	78.95	83.05
Photosystem II 22 kDa protein, chloroplastic	Q9XF91	chain_864	-1.8115	88.89	83.39
PGR5-like protein 1A, chloroplastic	Q8H112	chain_2974	-1.8107	92.65	85.48
Uncharacterized protein At5g50100, chloroplastic	Q8W485	chain_3255	-1.8103	63.6	75.0

continued on the next page

Contigs repressed at 34°C (*continued*)

Name	UniProt ID	Contig	$\log_2$ (fold-Change)	Target coverage (%)	Alignment identity (%)
Chlorophyll a-b binding protein 2.1, chloroplastic	Q9SHR7	chain_50125	-1.8082	61.82	91.04
Actin-related protein 8	Q9FKT0	chain_7564	-1.8074	95.65	78.71
Photosystem II reaction center W protein, chloroplastic	Q39194	chain_68853	-1.8049	72.34	82.09
Thioredoxin F2, chloroplastic	Q9XFH9	chain_362	-1.8047	98.86	83.53
F1N19.7	Q9SGW5	NODE_3826	-1.804	52.22	73.6
Protein LUTEIN DEFICIENT 5, chloroplastic	Q93VK5	NODE_7522	-1.8037	85.55	86.61
Probable aquaporin PIP2-5	Q9SV31	chain_44403	-1.8008	100	91.21
Ribulose bisphosphate carboxylase small chain 1A, chloroplastic	P10795	chain_34307	-1.7994	79.84	79.21
Protein LURP-one-related 5	Q9SSC7	NODE_26369	-1.797	77.22	81.67
Uncharacterized protein	A8MS05	chain_67613	-1.792	41.61	69.09
At1g64850/F13O11_15	Q9XIR0	chain_9645	-1.792	85.56	69.28
At1g77930	Q9SH08	chain_50124	-1.7902	89.44	85.31
Glutamine synthetase, chloroplastic/mitochondrial	Q43127	chain_4770	-1.7893	75.69	82.41
Cysteine proteinase inhibitor 6	Q8H0X6	chain_11208	-1.7858	98.8	68.38
At1g49010	Q9M9A3	chain_7856	-1.7851	54.74	80.39
Thiamine thiazole synthase, chloroplastic	Q38814	chain_122	-1.784	98.78	81.25
Chlorophyll a-b binding protein, chloroplastic	Q9LMQ2	NODE_41321	-1.7838	60.61	79.49
At1g30880	Q9FYH5	chain_2521	-1.7832	23.23	86.36
Myb-related protein 2	Q9SQQ9	NODE_10721	-1.7806	72.44	84.31
AT3G14830 protein	Q8L634	chain_2976	-1.7801	78.59	74.33
Glyceraldehyde-3-phosphate dehydrogenase GAPCP1, chloroplastic	Q9SAJ6	chain_69101	-1.7793	86.51	93.51
High chlorophyll fluorescence phenotype 173	Q8W4D6	chain_1587	-1.7755	79.71	84.94
AT1G49750 protein	Q9FXA1	chain_56267	-1.7749	61.11	65.63
Uncharacterized protein (Fragment)	A0A1P8B5H9	chain_313	-1.7746	78.57	75.61
Purine permease 1	Q9FZ96	chain_4611	-1.7734	79.38	66.14
Zinc finger protein CONSTANS	Q39057	chain_3410	-1.7725	98.71	72.01

continued on the next page

Contigs repressed at 34°C (*continued*)

Name	UniProt ID	Contig	$\log_2$ (fold-Change)	Target coverage (%)	Alignment identity (%)
At5g49760	Q8GZ99	chain_6123	-1.7714	56.16	72.41
AT3g24190/MUJ8_17	Q9LRN0	chain_5515	-1.7695	94.67	83.28
ARM repeat superfamily protein	Q93YW3	chain_10639	-1.7671	83.89	74.02
Thylakoid lumenal 29 kDa protein, chloroplastic	P82281	chain_32274	-1.766	85.0	75.91
Probable inactive ATP-dependent zinc metalloprotease FTSHI 3, chloroplastic	Q9M895	chain_12186	-1.765	98.56	85.71
Probable carboxylesterase 4, mitochondrial	Q9FX93	NODE_7918	-1.7624	68.98	55.12
Alkaline/neutral invertase E, chloroplastic	Q9FK88	chain_1366	-1.7611	88.54	79.76
Putative methylesterase 14, chloroplastic	Q9FVW3	chain_2439	-1.7591	89.47	76.1
SAUR-like auxin-responsive protein family	F4HZ54	chain_7839	-1.758	86.23	69.63
UDP-D-apiose/UDP-D-xylose synthase 1	Q9ZUY6	chain_6103	-1.7567	90.4	89.47
Probable ribose-5-phosphate isomerase 4, chloroplastic	Q9FI13	chain_52926	-1.7561	54.26	68.6
Chlorophyll a-b binding protein CP26, chloroplastic	Q9XF89	chain_253	-1.7525	55.67	86.79
Inositol-phosphate phosphatase	Q9M8S8	chain_62118	-1.7501	85.53	81.08
Ferredoxin-3, chloroplastic	Q9ZQG8	chain_54765	-1.7501	60.0	68.42
Phosphoglycolate phosphatase	F4K390	NODE_1510	-1.7491	60.14	73.66
Secondary thiamine-phosphate synthase enzyme	Q9LPU1	chain_1682	-1.7489	66.06	84.62
Telomere repeat-binding protein 3	Q9C7B1	chain_4598	-1.7476	57.31	76.92
Photosystem I reaction center subunit psaK, chloroplastic	Q9SUI5	chain_61774	-1.7467	45.76	88.46
Oxygen-evolving enhancer protein 2-1, chloroplastic	Q42029	chain_65581	-1.7459	54.55	75.86
At5g54760	Q9FFV1	chain_909	-1.7458	38.58	90.67
At2g41120	O80675	NODE_9752	-1.745	52.78	78.79
Expressed protein	Q9SKI3	chain_46634	-1.7446	42.13	70.27
Photosystem I reaction center subunit N, chloroplastic	P49107	NODE_63720	-1.7445	51.92	88.46
Inositol-tetrakisphosphate 1-kinase 3	Q9SUG3	chain_5712	-1.7428	85.53	85.19
Chlorophyllide a oxygenase, chloroplastic	Q9MBA1	chain_7154	-1.7424	92.24	82.97

continued on the next page

Contigs repressed at 34°C (*continued*)

Name	UniProt ID	Contig	$\log_2$ (fold-Change)	Target coverage (%)	Alignment identity (%)
Ribulose bisphosphate carboxylase/oxygenase activase, chloroplastic	P10896	chain_281	-1.74	36.54	88.89
Mannan endo-1,4-beta-mannosidase 7	Q9FJZ3	chain_308	-1.7392	88.43	57.82
Methylesterase 10	Q8S9K8	chain_7532	-1.7348	87.59	61.46
Rhodanese-like domain-containing protein 15, chloroplastic	Q38853	chain_21453	-1.7303	96.61	66.07
Protein GLUTAMINE DUMPER 5	Q3E965	NODE_18795	-1.7292	18.85	81.82
Probable protein phosphatase 2C 26	O64730	chain_5966	-1.7285	67.26	72.87
Histone H3-like 2	Q9FXI7	NODE_28755	-1.7227	46.94	90.91
Elongation factor Tu, chloroplastic	P17745	chain_61336	-1.7218	30.0	90.0
Photosystem I chlorophyll a/b-binding protein 3-1, chloroplastic	Q9SY97	NODE_36736	-1.7188	84.31	90.48
Homogentisate solanesyltransferase, chloroplastic	Q1ACB3	NODE_11099	-1.7187	100.0	86.7
Senescence-associated protein 13	Q9ZW18	chain_38468	-1.7186	48.0	77.14
Transmembrane protein, putative (DUF1118)	Q94F10	chain_2249	-1.7176	77.6	70.95
40S ribosomal protein S11-3	P42733	chain_14320	-1.7171	97.56	90.57
Transcription factor TCP3	Q9MAH8	chain_6121	-1.7163	24.93	87.95
Carotenoid 9,10(9',10')-cleavage dioxygenase 1	O65572	chain_48105	-1.7159	55.17	100.0
Peroxisomal membrane protein 11B	Q9STY0	chain_5551	-1.7157	97.45	77.94
Transcription factor PIF3	O80536	chain_3622	-1.7154	18.36	85.07
Transcription and mRNA export factor ENY2	Q6NQ54	chain_7311	-1.711	74.17	71.59
DNA-directed RNA polymerase subunit beta	A8MR80	chain_928	-1.71	25.82	79.63
Transcription factor PIF1	Q8GZM7	NODE_9144	-1.7042	37.93	86.84
PsbP domain-containing protein 4, chloroplastic	O49292	chain_62123	-1.7012	71.12	85.2
Actin-related protein 9	Q9LSW2	chain_4892	-1.7011	95.41	73.08
Chlorophyll a-b binding protein CP29.2, chloroplastic	Q9XF88	chain_48213	-1.7003	41.25	71.88
Protochlorophyllide-dependent translocon component 52, chloroplastic	Q8W496	chain_1271	-1.7003	97.24	74.94
Chlorophyll a-b binding protein, chloroplastic	Q39142	chain_45840	-1.6996	80.0	95.74

continued on the next page

Contigs repressed at 34°C (*continued*)

Name	UniProt ID	Contig	$\log_2$ (fold-Change)	Target coverage (%)	Alignment identity (%)
At1g22630/F12K8_2	Q9SKA5	chain_1221	-1.6978	80.77	79.52
Photosystem II 22 kDa protein, chloroplastic	Q9XF91	chain_44837	-1.6962	76.74	84.21
F-box protein At2g16365	Q84V03	chain_9837	-1.6952	9.02	81.82
Expressed protein	Q8VXY0	chain_2510	-1.695	60.1	83.33
Transmembrane protein	Q9LY44	chain_29399	-1.6943	32.99	87.1
Chlorophyll a-b binding protein, chloroplastic	Q9LMQ2	NODE_45370	-1.6913	46.97	73.33
NAD(P)H-quinone oxidoreductase subunit L, chloroplastic	Q9CAC5	chain_6621	-1.6866	44.9	68.97
Photosynthetic NDH subunit of lumenal location 2, chloroplastic	Q9XI73	chain_2031	-1.6843	60.47	80.39
Uncharacterized protein	A8MQF3	NODE_13220	-1.6819	98.1	73.53
Expressed protein	Q9SJH9	NODE_22340	-1.6806	68.0	72.73
Ferredoxin-1, chloroplastic	O04090	chain_49	-1.6803	95.33	76.56
Probable serine/threonine-protein kinase WNK7	Q8LST2	NODE_2454	-1.6801	70.86	75.92
DNA-directed DNA polymerase	Q9SYL7	chain_6832	-1.6759	25.38	69.39
Oxygen-evolving enhancer protein 1-1, chloroplastic	P23321	chain_59134	-1.6711	49.09	80.77
Photosystem II core complex proteins psbY, chloroplastic	O49347	chain_52154	-1.6702	56.0	75.62
Protochlorophyllide reductase A, chloroplastic	Q42536	chain_6384	-1.6672	100.0	92.35
Putative receptor-like protein kinase At3g47110	Q9SD62	chain_10892	-1.664	70.7	51.98
Putative L-ascorbate peroxidase 6	Q8GY91	chain_8683	-1.6639	70.38	74.55
ACT domain-containing protein ACR4	Q8LJW3	NODE_3325	-1.6534	87.19	69.97
AT3G26510 protein	Q6ID88	NODE_12483	-1.6533	65.93	74.42
DAR GTPase 3, chloroplastic	Q8H1F6	NODE_3404	-1.6529	79.1	75.84
Alpha/beta-Hydrolases superfamily protein	Q93Z34	chain_7317	-1.652	84.79	67.12
NAD(P)H-quinone oxidoreductase subunit U, chloroplastic	Q84VQ4	chain_6329	-1.6518	60.98	66.9
50S ribosomal protein L5, chloroplastic	O04603	chain_14438	-1.65	91.84	79.85
Pyruvate, phosphate dikinase 1, chloroplastic	O23404	chain_3615	-1.6465	96.41	83.61
Cytochrome P450 82C4	Q9SZ46	NODE_2457	-1.6445	81.06	62.31

continued on the next page

Contigs repressed at 34°C (*continued*)

Name	UniProt ID	Contig	$\log_2$ (fold-Change)	Target coverage (%)	Alignment identity (%)
At4g13220	Q6NLB6	chain_881	-1.6407	39.07	81.03
Chlorophyll a-b binding protein 6, chloroplastic	Q01667	chain_7910	-1.6404	57.53	92.68
Oxygen-evolving enhancer protein 3-1, chloroplastic	Q9XFT3	chain_14465	-1.6382	78.57	74.07
Lipoxygenase 3, chloroplastic	Q9LNR3	NODE_4896	-1.6355	98.33	70.73
ALBINO3-like protein 2, chloroplastic	Q8L718	chain_5629	-1.6353	87.02	64.4
RNA-binding protein CP31B, chloroplastic	Q9FGS0	chain_1776	-1.6349	61.62	75.23
Tubby-like F-box protein 3	Q8VY21	chain_11666	-1.6346	91.21	84.04
Cytochrome P450 97B3, chloroplastic	O23365	chain_4617	-1.6326	98.72	82.78
At4g31530	Q8GYZ0	chain_8361	-1.6279	80.25	85.26
Oxygen-evolving enhancer protein 1-1, chloroplastic	P23321	chain_34403	-1.6248	57.14	89.36
Alanine–tRNA ligase, chloroplastic/mitochondrial	Q9FFC7	chain_887	-1.6239	93.15	75.9
Protein LOW PSII ACCUMULATION 1, chloroplastic	Q9SRY4	chain_6064	-1.6235	92.83	79.0
ETO1-like protein 2	Q9LV01	chain_12995	-1.6231	88.67	73.64
Photosystem I chlorophyll a/b-binding protein 3-1, chloroplastic	Q9SY97	NODE_44817	-1.6227	63.64	90.24
Rubredoxin family protein	Q9FFJ2	NODE_3840	-1.6214	59.77	88.16
Bifunctional riboflavin kinase/FMN phosphatase	Q84MD8	chain_8912	-1.6188	36.96	62.5
Rhodanese-like domain-containing protein 7	Q1JPN0	chain_895	-1.6187	84.4	85.76
Plastid division protein CDP1, chloroplastic	Q8VY16	NODE_309	-1.6184	87.87	76.55
NOL1/NOP2/sun family protein / antitermination NusB domain-containing protein	Q8VYC4	chain_11358	-1.6172	99.53	79.05
Long chain base biosynthesis protein 2b	Q9M304	NODE_20114	-1.617	93.02	84.03
Isoflavone reductase homolog P3	P52577	chain_8370	-1.6168	94.62	69.39
At5g38640	Q9FFV8	chain_2435	-1.6132	89.36	87.56
Chloroplast sensor kinase, chloroplastic	F4HVG8	chain_7126	-1.6116	85.61	81.18
Photosystem II reaction center W protein, chloroplastic	Q39194	chain_13258	-1.6113	42.59	86.67
Glutathione hydrolase 3	Q9M0G0	NODE_67719	-1.6093	88.46	86.36

continued on the next page

Contigs repressed at 34°C (*continued*)

Name	UniProt ID	Contig	$\log_2$ (fold-Change)	Target coverage (%)	Alignment identity (%)
Thioredoxin-like protein HCF164, chloroplastic	O23166	chain_2634	-1.6083	70.23	79.23
Heparanase-like protein 2	Q8L608	chain_869	-1.6066	93.78	69.83
Lipoxygenase 4, chloroplastic	Q9FNX8	NODE_270	-1.6029	96.38	65.31
At5g49760	Q8GZ99	chain_7429	-1.6026	49.12	70.91
Leucine-rich repeat receptor-like serine/threonine-protein kinase BAM1	O49545	NODE_3947	-1.602	88.42	78.98
DUF1995 domain protein, putative (DUF1995)	Q94K68	NODE_7564	-1.6001	97.34	84.71
ATP-dependent Clp protease adapter protein CLPS1, chloroplastic	Q9SX29	chain_1875	-1.5927	66.08	86.61
Ribosome-recycling factor, chloroplastic	Q9M1X0	chain_847	-1.592	74.14	79.52
AFG1-like ATPase family protein	Q8L517	chain_44765	-1.5919	100.0	69.7
Chlorophyll a-b binding protein CP26, chloroplastic	Q9XF89	chain_54351	-1.5903	75.56	81.82
Protein FAR1-RELATED SEQUENCE 3	Q9ZVC9	chain_8642	-1.5902	96.81	76.39
50S ribosomal protein L12-3, chloroplastic	P36212	chain_56459	-1.5898	38.71	82.61
Transcription initiation factor TFIID subunit 12b	Q940A7	chain_5938	-1.5882	35.59	100.0
Oxygen-evolving enhancer protein 2-1, chloroplastic	Q42029	chain_62336	-1.5805	75.86	100.0
Cytochrome c biogenesis protein CCS1, chloroplastic	Q9XIA4	chain_5185	-1.5796	79.33	80.36
50S ribosomal protein L31, chloroplastic	Q9FWS4	chain_43905	-1.577	64.12	77.11
Protein RETICULATA-RELATED 5, chloroplastic	Q9SIY5	chain_8344	-1.5762	84.56	79.9
mRNA-decapping enzyme-like protein	Q9SJF3	chain_8087	-1.5735	61.01	83.18
Ribulose bisphosphate carboxylase small chain	F4KA76	NODE_31151	-1.5734	45.45	86.21
Glutamine synthetase cytosolic isozyme 1-3	Q9LVI8	NODE_40465	-1.5726	100	60.0
High mobility group B protein 3	P93047	chain_3538	-1.5683	61.43	70.59
Probable pectinesterase/pectinesterase inhibitor 6	O49298	chain_3752	-1.5677	61.82	71.18
ABC transporter G family member 32	O81016	chain_3352	-1.5638	58.93	70.0
Transketolase-2, chloroplastic	F4IW47	NODE_49847	-1.5634	42.59	81.82
Initiation factor 4F subunit (DUF1350)	Q9M236	NODE_5745	-1.5617	95.37	74.15
Leucine-rich repeat transmembrane protein kinase	F4I337	chain_25411	-1.5545	92.91	72.07

continued on the next page

Contigs repressed at 34°C (*continued*)

Name	UniProt ID	Contig	$\log_2$ (fold-Change)	Target coverage (%)	Alignment identity (%)
Glucose-6-phosphate 1-epimerase	Q9M308	chain_672	-1.552	87.71	77.18
At1g51400/F5D21_10	Q9SYE2	chain_14287	-1.5508	44.0	78.0
30S ribosomal protein S17, chloroplastic	P16180	chain_53937	-1.5483	59.78	82.29
Calcineurin B-like protein 3	Q8LEM7	chain_9443	-1.5483	98.27	90.71
Probable pyridoxal 5'-phosphate synthase subunit PDX2	Q8LAD0	chain_6591	-1.5466	81.67	69.07
Amidophosphoribosyltransferase 2, chloroplastic	Q9STG9	NODE_1023	-1.5464	89.61	85.12
NAD(P)H-quinone oxidoreductase subunit O, chloroplastic	Q9S829	NODE_10799	-1.5427	52.27	89.01
Magnesium-chelatase subunit ChlH, chloroplastic	Q9FNB0	chain_4805	-1.5383	90.26	89.47
Chlorophyll a-b binding protein 6, chloroplastic	Q01667	chain_1702	-1.537	55.75	92.8
DNA damage-binding protein 1a	Q9M0V3	chain_656	-1.5347	91.39	70.8
At2g28120/F24D13.9	Q9ZUV4	NODE_3138	-1.5254	92.98	66.67
Catalase-3	Q42547	chain_40546	-1.5219	53.73	71.43
Chromatin structure-remodeling complex protein BSH	P93045	chain_7635	-1.5172	89.49	78.17
Inositol requiring 1-1	F4KH41	NODE_108	-1.5167	89.42	70.83
Probable metal-nicotianamine transporter YSL5	Q9LUN2	chain_5996	-1.5104	96.38	68.94
Probable lactoylglutathione lyase, chloroplastic	Q8W593	chain_1260	-1.5034	78.26	97.14
PsbP domain-containing protein 1, chloroplastic	O23403	chain_3760	-1.4995	81.0	80.34
50S ribosomal protein L35, chloroplastic	Q8VZ55	chain_1161	-1.4992	47.4	87.5
Multiple organellar RNA editing factor 5, chloroplastic/mitochondrial	Q9C7Y2	chain_11124	-1.4881	44.06	69.35
Tetrapyrrole-binding protein, chloroplastic	Q9LX31	NODE_18628	-1.4859	88.48	68.28
At2g39670/F17A14.4	O48815	NODE_2677	-1.4857	95.8	81.59
WW domain-containing protein	Q67ZZ1	chain_1409	-1.4826	61.18	82.08
High mobility group B1	F4J5M5	chain_4184	-1.4823	65.31	66.67
Protein DETOXIFICATION 31	Q9LPV4	chain_2414	-1.481	95.16	75.13
NAD(P)-binding Rossmann-fold superfamily protein	Q8H1Q6	NODE_16367	-1.4755	100.0	75.37
AT4g21580/F18E5_200	O65423	chain_67202	-1.475	93.25	76.16

continued on the next page

Contigs repressed at 34°C (*continued*)

Name	UniProt ID	Contig	$\log_2$ (fold-Change)	Target coverage (%)	Alignment identity (%)
AT3g08780/F17O14_25	Q8RY09	chain_5678	-1.4735	94.12	61.83
TLDc domain protein	Q682Q6	chain_679	-1.4724	95.29	87.1
Chlorophyll a-b binding protein 2.4, chloroplastic	Q9XF87	NODE_70762	-1.4714	100	84.38
Glycine cleavage system H protein 1, mitochondrial	P25855	chain_3726	-1.4706	93.33	84.68
Putative calcium-transporting ATPase 7, plasma membrane-type	O64806	chain_12956	-1.4655	90.57	83.44
Protein REVEILLE 1	F4KGY6	chain_2962	-1.4643	51.55	79.59
Protein TAB2 homolog, chloroplastic	Q9SFB3	chain_1317	-1.4636	98.78	83.06
Probable carboxylesterase 3	Q9FX92	chain_3440	-1.4596	63.51	65.22
Protein TIC 22, chloroplastic	Q9SZB2	chain_3947	-1.4564	69.1	78.35
Alpha,alpha-trehalose-phosphate synthase [UDP-forming] 1	Q9SYM4	chain_9196	-1.455	79.64	86.26
ABC transporter G family member 32	O81016	chain_41186	-1.4537	79.59	69.82
Glutamyl-tRNA(Gln) amidotransferase subunit B, chloroplastic/mitochondrial	Q9FV81	NODE_1405	-1.4537	91.62	80.98
AT5g40850/MHK7_8	Q42606	chain_9728	-1.4305	90.4	84.27
AAA-ATPase At2g46620	F4IJ77	NODE_2251	-1.4286	81.25	76.78
Lycopene beta cyclase, chloroplastic	Q38933	chain_2507	-1.4115	88.34	87.13
50S ribosomal protein L34, chloroplastic	Q9LP37	NODE_12517	-1.4098	36.41	87.88
Fructose-1,6-bisphosphatase, cytosolic	Q9MA79	chain_1762	-1.4071	97.73	85.96
Photosystem I reaction center subunit III, chloroplastic	Q9SHE8	chain_5058	-1.3969	89.88	89.33
DHHA1 domain protein	F4HRG2	chain_53574	-1.3948	94.85	74.6
Uncharacterized TPR repeat-containing protein At1g05150	O23052	chain_10749	-1.3853	97.36	78.46
BTB/POZ domain-containing protein At3g19850	Q9LT24	NODE_17517	-1.3837	90.05	62.94
Putative GTP-binding protein ara-3	Q9FJF1	chain_5571	-1.3835	100.0	85.19
Guanosine deaminase	Q94BU8	NODE_11743	-1.3676	88.12	84.18
Protein plastid transcriptionally active 16, chloroplastic	Q9STF2	chain_29148	-1.3656	37.58	69.09
Fructose-1,6-bisphosphatase 1, chloroplastic	P25851	chain_14842	-1.3562	93.51	90.7
At2g16940	Q9ZVW9	chain_2977	-1.354	31.32	72.64

continued on the next page

Contigs repressed at 34°C (*continued*)

Name	UniProt ID	Contig	$\log_2$ (fold-Change)	Target coverage (%)	Alignment identity (%)
Protein indeterminate-domain 5, chloroplastic	Q9ZUL3	chain_5236	-1.3481	42.1	88.0
Protein SEH1	Q93VR9	NODE_4411	-1.341	96.17	73.09
NAD(P)-linked oxidoreductase superfamily protein	Q8VZ23	chain_10026	-1.3382	99.16	82.06
Alpha/beta-Hydrolases superfamily protein	Q8L7H5	chain_8265	-1.3334	79.7	60.56
At1g55530/T5A14_7	Q9ZVU8	NODE_2880	-1.3284	55.52	76.73
Acyltransferase-like protein At1g54570, chloroplastic	Q9ZVN2	chain_11873	-1.3196	83.7	71.7
Ubiquitin-specific protease 13	F4J7I2	chain_1843	-1.3187	96.93	93.02
Transducin/WD40 repeat-like superfamily protein	Q9M1E5	chain_4828	-1.312	94.82	69.15
Putative endoribonuclease E	Q8GVF1	chain_7082	-1.3101	80.04	86.18
Secondary thiamine-phosphate synthase enzyme	Q9LPU1	chain_33830	-1.298	57.82	85.95
Shaggy-related protein kinase alpha	P43288	NODE_2527	-1.2924	79.75	89.24
Pleckstrin homology (PH) domain-containing protein	F4JNE4	chain_6671	-1.2872	88.81	76.55
Soluble inorganic pyrophosphatase 4	Q9LFF9	NODE_60934	-1.2684	68.97	82.05
Glycine dehydrogenase (decarboxylating) 1, mitochondrial	Q94B78	chain_33602	-1.2543	98.11	86.67
Probable pyridoxal 5'-phosphate synthase subunit PDX2	Q8LAD0	NODE_10093	-1.254	74.68	89.47
Lysine-tRNA ligase	Q8RWI4	chain_9302	-1.2488	96.62	88.94
KH domain-containing protein At1g09660/At1g09670	Q8GWR3	NODE_3694	-1.2413	92.54	74.62
Serine protease SPPA, chloroplastic	Q9C9C0	chain_4459	-1.2376	86.33	79.07
Uncharacterized GPI-anchored protein At1g61900	Q8GUI4	chain_20750	-1.223	92.55	74.88
Leucine–tRNA ligase, chloroplastic/mitochondrial	Q9XEA0	chain_70979	-1.2222	95.22	80.1
UTP–glucose-1-phosphate uridylyltransferase 2	Q9M9P3	chain_4423	-1.2117	96.23	83.88
Zincin-like metalloproteases family protein	A0A1R7T3A4	chain_49240	-1.2085	98.14	83.58
Glutamine synthetase, chloroplastic/mitochondrial	Q43127	chain_13377	-1.2082	88.89	95.8
Thioredoxin-like 3-1, chloroplastic	Q9FG36	chain_7139	-1.2077	36.21	85.48
AT3g09210/F3L24_8	Q94AA3	NODE_6421	-1.1625	76.72	73.45
Myosin heavy chain-like protein	Q9SY40	NODE_5197	-1.1518	54.12	76.22

continued on the next page

Contigs repressed at 34°C (*continued*)

Name	UniProt ID	Contig	$\log_2$ (fold-Change)	Target coverage (%)	Alignment identity (%)
Probable esterase KAI2	Q9SZU7	NODE_5577	-1.0977	94.12	84.25

Table S14: **Annotated contigs promoted at 34°C in *Nothofagus pumilio*. Target coverage:** Percentage of *A. thaliana* protein covered by the alignment with *N. pumilio* query. **Alignment identity:** Percentage of matches relative to the length of the *N. pumilio* – *A. thaliana* alignment

Name	UniProt ID	Contig	$\log_2$ (fold-Change)	Target coverage (%)	Alignment identity (%)
Peptidyl-prolyl cis-trans isomerase CYP18-4	Q42406	chain_2034	23.5409	94.52	86.13
40S ribosomal protein S10-2	Q9FFS8	chain_4429	23.1602	23.3	91.3
Polypyrimidine tract-binding protein homolog 2	Q9FGL9	chain_13848	15.5539	64.8	95.0
Acidic endochitinase	P19172	NODE_22553	12.8037	62.03	68.75
ATP-dependent zinc metalloprotease FTSH 5, chloroplastic	Q9FH02	chain_3119	12.7599	69.46	96.43
Probable histone H2AXb	Q9S9K7	chain_1844	12.4364	95.24	95.68
Heat shock 70 kDa protein 10, mitochondrial	Q9LDZ0	chain_6607	12.282	77.51	88.72
Peptidyl-prolyl cis-trans isomerase CYP18-3	P34790	chain_24428	11.9153	24.11	76.92
3-oxoacyl-[acyl-carrier-protein] synthase III, chloroplastic	P49243	NODE_2540	11.8831	48.79	83.26
Expressed protein	Q9SL96	NODE_214	11.532	78.86	68.09
UDP-glycosyltransferase 74D1	Q9SKC5	chain_6519	11.4708	92.61	69.23
Uncharacterized protein At2g37660, chloroplastic	O80934	chain_70179	11.2598	100	87.32
Acidic endochitinase	P19172	NODE_37185	11.1772	76.92	66.67
5-methyltetrahydropteroyltriglutamate–homocysteine methyltransferase 3, chloroplastic	Q0WNZ5	chain_8894	11.0983	54.41	63.89
Alkaline ceramidase	Q94IB9	chain_147	10.9084	64.52	89.47
Polyadenylate-binding protein-interacting protein 12	Q9S7N9	chain_7279	10.8675	97.04	80.79
Evolutionarily conserved C-terminal region 4	A0A1P8AS03	chain_67583	10.6327	82.19	81.68
4-coumarate–CoA ligase-like 7	Q9M0X9	chain_13052	10.6213	87.94	59.46
L-type lectin-domain containing receptor kinase IV.2	Q9M345	chain_13571	10.5615	61.11	73.91

continued on the next page

Contigs promoted at 34°C (*continued*)

Name	UniProt ID	Contig	$\log_2$ (fold-Change)	Target coverage (%)	Alignment identity (%)
Cathepsin B-like protease 3	Q94K85	NODE_6799	10.4842	74.18	67.8
22.0 kDa heat shock protein	Q38806	NODE_13387	10.2988	86.21	75.3
Transmembrane protein	Q9LW52	NODE_16017	10.2763	34.64	54.55
Methyl-CpG-binding domain-containing protein 11	Q9LW00	chain_13748	10.2573	34.08	67.62
Homeobox-leucine zipper protein HAT7	Q00466	chain_10630	10.121	23.82	71.25
24-methylenesterol C-methyltransferase 3	Q94JS4	NODE_27931	10.1118	97.62	77.5
Glycine-rich protein family	A8MRN5	NODE_18888	10.0943	74.51	50.67
Protein EXORDIUM-like 2	Q9FE06	chain_898	9.8853	38.74	64.29
Probable glucan endo-1,3-beta-glucosidase BG1	Q9M2M0	chain_70082	9.8827	77.34	65.16
Protein SRG1	Q39224	NODE_5489	9.8431	54.95	61.73
Cupin family protein	Q9SIA7	chain_1929	9.6211	59.74	58.24
Protein NRT1/ PTR FAMILY 7.3	Q9LQL2	NODE_9734	9.6201	94.12	69.84
Wiskott-aldrich syndrome family protein, putative (DUF1118)	Q9SD79	chain_1168	9.5105	62.77	79.49
40S ribosomal protein S8-2	Q9FIF3	chain_804	9.4658	97.57	87.98
Acidic endochitinase	P19172	NODE_22554	9.442	76.92	66.67
Probable glucan endo-1,3-beta-glucosidase BG1	Q9M2M0	chain_39090	9.3646	55.32	80.0
Acidic endochitinase	P19172	chain_39977	9.3625	60.56	72.73
AT3g52230/F4F15_340	Q9SUY2	chain_45375	9.3046	19.39	81.08
Probable cysteine protease RDL2	Q9LT77	chain_12722	9.2863	50.54	58.47
Acetyl-CoA acetyltransferase, cytosolic 1	Q8S4Y1	chain_14406	9.2665	40.85	85.71
Vacuolar-processing enzyme alpha-isozyme	P49047	NODE_10428	9.1205	30.3	65.75
NAD(P)H-quinone oxidoreductase subunit T, chloroplastic	Q9SMS0	chain_32087	9.1164	57.41	74.68
Mediator of RNA polymerase II transcription subunit 25	Q7XYY2	NODE_18494	9.0438	13.92	73.08
Plant basic secretory protein (BSP) family protein	Q9ZUJ8	chain_30990	9.0152	70.72	66.04
Non-specific lipid-transfer protein 3	Q9LLR7	NODE_21736	8.9485	68.04	55.38
F12F1.21 protein	O65387	chain_12394	8.8661	51.68	68.42

continued on the next page

Contigs promoted at 34°C (*continued*)

Name	UniProt ID	Contig	$\log_2$ (fold-Change)	Target coverage (%)	Alignment identity (%)
Chitinase-like protein 2	Q9LSP9	chain_5580	8.765	27.72	74.07
Zinc finger CCCH domain-containing protein 22	Q9SK49	chain_10221	8.7546	99.71	69.37
Cytochrome b561 and DOMON domain-containing protein At3g59070	Q9LYS9	chain_12220	8.744	49.2	60.98
Protein MOTHER of FT and TFL1	Q9XFK7	chain_8281	8.6018	77.39	65.88
Probable glucan endo-1,3-beta-glucosidase BG1	Q9M2M0	NODE_10408	8.5487	85.51	62.96
Nudix hydrolase 15, mitochondrial	Q8GYB1	chain_4976	8.4961	80.06	79.83
Asparagine synthetase [glutamine-hydrolyzing] 1	P49078	NODE_31923	8.4667	79.45	87.72
Aspartic proteinase A1	O65390	NODE_21071	8.4146	62.64	58.93
DEAD-box ATP-dependent RNA helicase 20	Q9C718	chain_16721	8.3452	87.76	73.81
Glycine-rich protein 23	A0A1P8B273	NODE_14405	8.3217	74.1	49.86
Bidirectional sugar transporter SWEET15	Q9FY94	NODE_26914	8.1762	26.97	78.26
Mitochondrial import inner membrane translocase subunit PAM16 like 1	Q93W66	chain_12848	8.0774	70.87	72.29
Ethylene-responsive transcription factor ERF017	Q84QC2	NODE_14797	8.0571	54.19	67.47
Transmembrane protein	F4JLQ2	chain_34752	8.0418	47.3	70.59
Fructose-bisphosphate aldolase 8, cytosolic	Q9LF98	NODE_9399	8.0215	76.92	59.57
Transcription factor bHLH120	Q9FLI0	NODE_9162	8.0028	24.73	63.24
Glyceraldehyde-3-phosphate dehydrogenase GAPC2, cytosolic	Q9FX54	NODE_17555	8.0019	87.5	78.33
Probable pectin methyltransferase QUA2	Q9C9Q8	chain_5336	7.9646	77.85	77.24
Tubulin beta-5 chain	P29513	NODE_30140	7.8805	91.3	81.73
Probable glutathione peroxidase 2	O04922	NODE_36417	7.8682	27.45	70.37
Cystathionine gamma-synthase 1, chloroplastic	P55217	chain_57939	7.8603	27.06	81.82
Cell division control protein 48 homolog E	Q9LZF6	chain_13671	7.8137	89.83	82.69
Putative glycine-rich cell wall protein	O65514	chain_14575	7.7925	65.63	60.98
ATP-dependent zinc metalloprotease FTSH 1, chloroplastic	Q39102	chain_30277	7.7885	90.63	84.88
Putative glycine-rich cell wall protein	O65514	NODE_46301	7.7863	81.4	55.48
Triosephosphate isomerase, chloroplastic	Q9SKP6	NODE_16242	7.7861	87.95	80.65

continued on the next page

Contigs promoted at 34°C (*continued*)

Name	UniProt ID	Contig	$\log_2$ (fold-Change)	Target coverage (%)	Alignment identity (%)
Eukaryotic translation initiation factor 5A-3	Q9C505	NODE_58018	7.7824	28.57	80.95
Plasma membrane ATPase	A0A1P8B2V0	chain_65025	7.781	42.11	91.3
Proline-rich extensin-like protein EPR1	Q9ZQI0	chain_1293	7.7669	85.71	78.65
Pathogenesis-related thaumatin superfamily protein	Q9LNT0	NODE_30638	7.7642	100.0	74.29
S-adenosyl-L-methionine-dependent methyltransferases superfamily protein	Q9FJZ6	NODE_54393	7.745	46.84	61.11
Arogenate dehydrogenase 1, chloroplastic	Q944B6	chain_43850	7.74	30.88	85.0
17.6 kDa class I heat shock protein 2	Q9ZW31	chain_3364	7.7317	91.41	81.9
Cysteine proteinases superfamily protein	Q9LNC1	NODE_7482	7.7304	38.52	56.52
Ribulose bisphosphate carboxylase small chain 3B, chloroplastic	P10798	chain_65476	7.7169	10.53	100.0
Protein yippee-like At4g27745	Q9T096	chain_24847	7.7166	85.96	58.76
ABC transporter G family member 35	Q7PC86	chain_60446	7.7155	76.64	78.25
Naringenin,2-oxoglutarate 3-dioxygenase	Q9S818	chain_61519	7.715	62.79	92.31
Tubulin beta-3 chain	Q9ASR0	NODE_12616	7.6953	100	85.64
Acidic endochitinase	P19172	NODE_19415	7.685	95.81	66.09
Glycine-rich cell wall structural protein	P27483	NODE_18815	7.655	50.55	62.38
Dihydrolipoyllysine-residue acetyltransferase component 1 of pyruvate dehydrogenase complex, mitochondrial	Q0WQF7	NODE_36492	7.6429	58.42	69.39
Transcription initiation factor TFIID subunit 12b	Q940A7	NODE_6800	7.6325	26.0	65.52
tRNA pseudouridine synthase	F4I2L0	chain_44310	7.6214	81.82	79.55
Purine permease 1	Q9FZ96	chain_1172	7.5445	79.38	66.14
RNA-binding (RRM/RBD/RNP motifs) family protein	F4JNU1	NODE_35447	7.5404	25.53	78.26
Fe-S cluster assembly factor HCF101, chloroplastic	Q6STH5	chain_19747	7.5361	91.96	88.24
Succinate dehydrogenase [ubiquinone] flavoprotein subunit 2, mitochondrial	Q9ZPX5	NODE_21648	7.5302	99.42	79.41
Probable galactinol–sucrose galactosyltransferase 6	Q8RX87	NODE_76336	7.5188	78.46	75.51
Citrate synthase 5, mitochondrial	Q9M1D3	NODE_20027	7.5132	89.81	83.62
Allene oxide synthase, chloroplastic	Q96242	NODE_36372	7.4802	52.94	66.04

continued on the next page

Contigs promoted at 34°C (*continued*)

Name	UniProt ID	Contig	$\log_2$ (fold-Change)	Target coverage (%)	Alignment identity (%)
Fructose-bisphosphate aldolase 7, cytosolic	P22197	chain_722	7.4756	99.68	76.22
Microfibril-associated protein-like	Q9FKN6	chain_11460	7.357	40.63	76.32
Putative glycerol-3-phosphate transporter 5	Q9SL56	chain_1142	7.3457	40.28	92.86
Ethylene-responsive transcription factor ERF042	Q52QU1	chain_10417	7.3198	40.0	78.26
Peroxisomal (S)-2-hydroxy-acid oxidase GLO4	Q9LJH5	chain_56382	7.3132	50.0	65.12
At1g55450/T5A14_14	Q9ZVU4	NODE_10839	7.2989	90.82	61.31
Cytochrome P450 71A20	Q9T0K2	chain_15195	7.2864	94.02	64.52
Probable aquaporin PIP2-5	Q9SV31	NODE_61665	7.2863	89.29	87.5
F26K24.21 protein	Q9SF07	chain_12138	7.2799	81.71	63.64
Tubulin beta-3 chain	Q9ASR0	chain_13934	7.2665	93.5	80.7
Fructose-bisphosphate aldolase 5, cytosolic	O65581	NODE_26737	7.2538	73.6	70.33
Serine/threonine-protein kinase STY46	F4JTP5	chain_19641	7.2527	70.0	72.37
Proline-rich extensin-like family protein	Q84JV0	chain_25643	7.2433	27.5	51.85
DEAD-box ATP-dependent RNA helicase 56	Q9LFN6	chain_16517	7.2233	88.24	68.92
Protein kinase	Q9FGB1	NODE_9932	7.2127	27.46	84.21
Influenza virus NS1A-binding protein	F4K9G6	chain_11144	7.199	64.52	54.24
ABC transporter G family member 11	Q8RXN0	NODE_32439	7.1989	79.76	57.58
Vacuolar-processing enzyme gamma-isozyme	Q39119	chain_38258	7.1926	98.15	85.29
Translocator protein homolog	O82245	NODE_5859	7.1725	26.9	71.15
Calcium-transporting ATPase 3, endoplasmic reticulum-type	Q9SY55	chain_18944	7.1715	51.63	86.81
Ethylene-responsive transcription factor 15	Q8VYM0	NODE_9379	7.1667	9.66	70.37
Calcineurin-like metallo-phosphoesterase superfamily protein	F4JMQ8	chain_19073	7.1537	44.0	90.7
Aldehyde dehydrogenase family 2 member B4, mitochondrial	Q9SU63	NODE_37064	7.1414	99.01	67.03
Transmembrane protein	Q9LW52	NODE_31797	7.0892	74.0	55.56
Sedoheptulose-1,7-bisphosphatase, chloroplastic	P46283	chain_6732	7.0877	88.27	90.14
Polygalacturonase inhibitor 1	Q9M5J9	NODE_63064	7.0848	87.84	63.56

continued on the next page

Contigs promoted at 34°C (*continued*)

Name	UniProt ID	Contig	$\log_2$ (fold-Change)	Target coverage (%)	Alignment identity (%)
Phospholipase A1-Igamma2, chloroplastic	Q3EBR6	chain_12708	7.0633	43.48	58.97
Polyubiquitin 10	Q8H159	chain_12064	7.0603	29.67	98.63
Photosystem I reaction center subunit II-2, chloroplastic	Q9SA56	chain_17819	7.0384	70.1	92.54
Nudix hydrolase 15, mitochondrial	Q8GYB1	chain_4609	7.0375	80.06	79.83
NAD(P)-linked oxidoreductase superfamily protein	Q9SJV2	NODE_10901	7.0208	43.48	60.33
Vacuolar-processing enzyme alpha-isozyme	P49047	NODE_6550	7.0172	49.33	68.35
Ethylene-responsive transcription factor ERF094	Q9LND1	NODE_70264	7.0159	44.44	70.37
Nicotinate phosphoribosyltransferase 1	Q8RWM2	NODE_11647	7.0083	87.13	78.77
Bifunctional enolase 2/transcriptional activator	P25696	NODE_32829	7.008	94.44	73.26
Probable ubiquitin-like-specific protease 2B	Q8L7S0	NODE_193	7.0025	61.81	80.63
Cystathionine gamma-synthase 1, chloroplastic	P55217	chain_34455	6.9969	27.06	81.82
Putative NADP-dependent oxidoreductase	Q9M9M7	chain_16791	6.9713	97.4	67.57
Monooxygenase 3	Q9FLC2	NODE_12661	6.9638	34.81	56.45
At4g13860	Q6ID29	NODE_25526	6.9503	27.97	61.54
UDP-N-acetylglucosamine diphosphorylase 1	Q940S3	NODE_23375	6.9267	54.37	61.82
Fe(2+) transport protein 3, chloroplastic	Q8LE59	chain_33751	6.9022	14.29	81.82
Probable cysteine protease RD21C	Q9LT78	NODE_26260	6.8884	80.0	64.47
Heat shock protein 90-1	P27323	NODE_22825	6.8738	100.0	64.96
Beta-glucosidase 14	Q9SLA0	NODE_46904	6.8606	93.62	69.77
Allene oxide synthase, chloroplastic	Q96242	NODE_16481	6.8424	98.65	54.17
Citrate synthase 5, mitochondrial	Q9M1D3	chain_15941	6.8232	100	65.24
(E,E)-geranyllinalool synthase	Q93YV0	chain_15522	6.8201	72.09	64.0
Transcription factor PRE4	F4JCN9	NODE_36374	6.8065	98.36	68.42
Probable receptor-like protein kinase At2g23200	O22187	chain_4218	6.8034	14.35	75.0
Multiple organellar RNA editing factor 8, chloroplastic/mitochondrial	Q9LKA5	chain_733	6.7945	69.35	87.59
Acidic endochitinase	P19172	NODE_28759	6.7797	45.59	70.0

continued on the next page

Contigs promoted at 34°C (*continued*)

Name	UniProt ID	Contig	$\log_2$ (fold-Change)	Target coverage (%)	Alignment identity (%)
Transcription factor bHLH126	Q9STJ6	chain_15910	6.7702	65.67	65.12
Peroxidase 52	Q9FLC0	chain_975	6.77	97.08	64.71
Heat shock 70 kDa protein 6, chloroplastic	Q9STW6	chain_34903	6.7653	63.0	67.74
Heavy metal-associated isoprenylated plant protein 24	O81464	NODE_63798	6.7626	95.16	77.59
Quinone oxidoreductase-like protein	Q9LFK5	NODE_59789	6.752	76.32	69.81
GDSL esterase/lipase 1	Q9FLN0	chain_34462	6.7398	85.57	62.92
5-methyltetrahydropteroyltriglutamate–homocysteine methyltransferase 2	Q9SRV5	NODE_75703	6.7235	91.8	67.27
Disease resistance protein (TIR-NBS-LRR class) family	Q9SW60	chain_15138	6.6977	68.75	61.19
Cytochrome P450 71B10	Q9LVD2	chain_10147	6.6897	73.08	62.5
Polyketide cyclase/dehydrase and lipid transport superfamily protein	Q9XIR9	NODE_17563	6.679	100.0	74.29
Casein kinase-like protein	Q9LID3	chain_19724	6.6768	67.02	82.08
3-oxo-Delta(4,5)-steroid 5-beta-reductase	Q9STX2	chain_43416	6.6726	68.0	75.25
Delta-aminolevulinic acid dehydratase 1, chloroplastic	Q9SFH9	NODE_48266	6.6612	72.62	65.0
Pleckstrin homology (PH) domain-containing protein	F4JNE4	chain_10351	6.6564	84.62	69.89
Malate dehydrogenase 1, mitochondrial	Q9ZP06	NODE_33310	6.6538	100.0	66.36
Mediator of RNA polymerase II transcription subunit 37a	Q9LKR3	chain_15255	6.6444	85.9	79.82
Alpha/beta-Hydrolases superfamily protein	Q9C8G6	chain_38186	6.6405	78.95	72.73
Spliceosome-associated protein 130 B	P0DKL6	chain_69997	6.6296	80.95	72.73
Tropinone reductase homolog At2g29370	Q9ZW20	NODE_35292	6.6181	72.38	65.33
ATP synthase subunit alpha, chloroplastic	P56757	NODE_72637	6.6019	97.37	91.67
Adenosine kinase 1	Q9SF85	NODE_44109	6.6016	35.23	70.0
Nudix hydrolase 15, mitochondrial	Q8GYB1	chain_50505	6.589	80.06	79.83
Probable inactive purple acid phosphatase 1	Q9LMX4	NODE_36295	6.588	28.41	75.0
Mediator of RNA polymerase II transcription subunit 37a	Q9LKR3	chain_17526	6.5822	84.62	78.57
Ribulose bisphosphate carboxylase/oxygenase activase, chloroplastic	P10896	NODE_25034	6.5654	72.92	79.41

continued on the next page

Contigs promoted at 34°C (*continued*)

Name	UniProt ID	Contig	$\log_2$ (fold-Change)	Target coverage (%)	Alignment identity (%)
Dynamin-related protein 1B	Q84XF3	NODE_32627	6.5608	73.12	89.55
[Fructose-bisphosphate aldolase]-lysine N-methyltransferase, chloroplastic	Q9XI84	NODE_17705	6.5554	52.27	81.82
Glycine-rich protein 5	Q9LTP5	chain_17885	6.5408	46.0	64.44
Probable galactinol–sucrose galactosyltransferase 6	Q8RX87	chain_11641	6.5364	95.81	86.83
Inosine-5'-monophosphate dehydrogenase 1	P47996	NODE_35713	6.5202	85.29	73.68
S-adenosyl-L-methionine-dependent methyltransferases superfamily protein	Q0WL44	chain_1481	6.5144	91.85	65.53
Homeodomain-like superfamily protein	A0A1P8B7F3	chain_8762	6.5097	21.82	77.14
Phytanoyl-CoA dioxygenase	Q9ZVF6	NODE_40328	6.5047	50.0	60.87
Pre-mRNA-processing-splicing factor 8B	Q9T0I6	NODE_50076	6.4865	81.93	73.13
Probable phospholipid-transporting ATPase 11	Q9SAF5	chain_15677	6.4801	99.17	71.19
Peroxidase 5	Q9M9Q9	chain_42687	6.4782	90.0	63.64
Probable mitochondrial-processing peptidase subunit beta, mitochondrial	Q42290	NODE_42084	6.4682	63.04	50.88
40S ribosomal protein S17-2	Q9SJ36	NODE_26003	6.4558	68.82	69.84
Nudix hydrolase 15, mitochondrial	Q8GYB1	chain_64883	6.4429	80.06	79.83
At4g35785	Q494N5	chain_27079	6.4406	35.48	80.95
DEAD-box ATP-dependent RNA helicase 56	Q9LFN6	NODE_36894	6.4392	87.64	83.12
Calreticulin-2	Q38858	chain_40308	6.4366	44.23	86.36
Protein NTM1-like 9	F4JN35	NODE_29357	6.428	97.67	73.17
Eukaryotic translation initiation factor 2 subunit beta	Q41969	NODE_53462	6.4214	80.0	62.75
Ubiquitin domain-containing protein DSK2b	Q9SII8	chain_6239	6.4166	52.51	75.94
Protein disulfide isomerase-like 1-1	Q9XI01	NODE_7425	6.414	53.65	69.62
Expansin-A1	Q9C554	NODE_44621	6.4082	98.41	57.38
Transducin family protein / WD-40 repeat family protein	Q8RXQ4	chain_43434	6.3986	44.78	68.97
Glycine-rich protein	Q9M0B4	NODE_48627	6.389	24.69	89.47
Gb—AAD25673.1	Q9FMG5	NODE_56967	6.3666	74.03	66.07
Nudix hydrolase 15, mitochondrial	Q8GYB1	chain_15885	6.3597	80.06	79.83

continued on the next page

Contigs promoted at 34°C (*continued*)

Name	UniProt ID	Contig	$\log_2$ (fold-Change)	Target coverage (%)	Alignment identity (%)
Tubulin-folding cofactor B	Q67Z52	NODE_50925	6.3375	69.7	55.56
Putative NADP-dependent oxidoreductase	Q9M9M7	NODE_38218	6.3333	65.06	66.04
Protein disulfide isomerase-like 1-4	Q9FF55	NODE_41815	6.3247	75.0	74.51
60S ribosomal protein L17-1	Q93VI3	NODE_13814	6.3214	79.29	71.71
Tubulin beta-3 chain	Q9ASR0	NODE_25920	6.32	82.14	95.45
Pentatricopeptide repeat-containing protein At3g13150	Q9LK58	NODE_33026	6.3155	36.9	66.67
Mediator of RNA polymerase II transcription subunit 25	Q7XYY2	NODE_76095	6.3034	76.47	69.96
Cell division control protein 48 homolog D	Q9SCN8	NODE_31425	6.3004	94.12	93.62
Shaggy-related protein kinase epsilon	Q8VZD5	chain_16088	6.2975	86.11	83.33
Heat shock protein 90-2	P55737	NODE_792	6.2963	93.66	76.32
Glutamine synthetase cytosolic isozyme 1-3	Q9LVI8	NODE_5168	6.2841	93.75	66.91
Protein transport protein Sec61 subunit gamma-3	Q9SMP2	NODE_36922	6.2826	67.86	78.38
Ethylene-responsive transcription factor ERF115	Q9LY29	NODE_4458	6.2789	25.0	87.32
Protein phosphatase 2C 37	P49598	NODE_11200	6.2725	87.71	69.78
Chaperonin CPN60-like 1, mitochondrial	Q8L7B5	NODE_8624	6.2664	99.05	65.22
Protein DJ-1 homolog B	Q9MAH3	NODE_63644	6.2639	84.0	80.95
3-hydroxyisobutyryl-CoA hydrolase-like protein 3, mitochondrial	Q9T0K7	NODE_51288	6.2629	65.43	57.69
At2g07725	Q6NMS1	chain_16984	6.2601	96.25	90.67
PAR1 protein	Q9FHC1	chain_27656	6.259	49.41	68.29
Glycine-rich protein 23	O48848	NODE_44059	6.2582	98.88	57.65
Sm-like protein LSM5	Q9FKB0	NODE_55331	6.2483	84.62	81.4
Aldehyde dehydrogenase family 2 member B4, mitochondrial	Q9SU63	NODE_50330	6.2447	93.1	80.77
Leucine-rich repeat extensin-like protein 6	Q9LUI1	chain_3411	6.2303	21.31	52.94
UDP-glycosyltransferase 74C1	Q9SKC1	chain_13514	6.2303	97.18	69.12
Probable glucan endo-1,3-beta-glucosidase BG3	F4J270	chain_10053	6.2282	95.96	70.3
S-formylglutathione hydrolase	Q8LAS8	NODE_16583	6.2274	12.85	90.91

continued on the next page

Contigs promoted at 34°C (*continued*)

Name	UniProt ID	Contig	$\log_2$ (fold-Change)	Target coverage (%)	Alignment identity (%)
ADP-ribosylation factor B1C	Q9M9N1	NODE_45172	6.2256	81.54	53.85
60S ribosomal protein L26-1	P51414	NODE_70814	6.2212	81.43	62.5
Vacuolar-processing enzyme gamma-isozyme	Q39119	chain_69452	6.213	98.15	85.29
Peptidyl-prolyl cis-trans isomerase FKBP15-1	Q38935	NODE_31119	6.2082	42.05	66.67
At5g20700	Q8GYX2	NODE_66282	6.2061	84.62	85.71
At1g05870	Q9MA39	chain_12135	6.2024	73.86	83.48
Endochitinase EP3	Q9M2U5	NODE_61560	6.1991	44.9	95.24
Cell division control protein 48 homolog E	Q9LZF6	NODE_5079	6.199	93.17	72.6
AAA-ATPase At2g46620	F4IJ77	NODE_15829	6.1989	62.35	60.23
Coatomer subunit beta-1	Q9SV21	NODE_38416	6.1814	81.43	80.0
60S ribosomal protein L7-4	Q9LHP1	NODE_32607	6.1802	66.02	56.72
NADH dehydrogenase [ubiquinone] flavoprotein 1, mitochondrial	Q9FNN5	NODE_34574	6.165	73.33	80.0
Fructose-bisphosphate aldolase 6, cytosolic	Q9SJQ9	NODE_30606	6.1615	98.1	69.89
V-type proton ATPase subunit E3	P0CAN7	NODE_41410	6.1601	64.62	58.54
Laccase-7	Q9SR40	chain_21092	6.16	76.92	55.1
Cysteine protease XCP1	O65493	chain_11565	6.1551	36.84	60.98
Heat shock 70 kDa protein 10, mitochondrial	Q9LDZ0	NODE_35955	6.1548	100	83.52
Respiratory burst oxidase homolog protein D	Q9FIJ0	NODE_43616	6.1536	77.46	81.48
Signal peptidase I	Q9SSR2	NODE_34750	6.1481	91.49	64.29
Probable alpha,alpha-trehalose-phosphate synthase [UDP-forming] 9	Q9LRA7	chain_5891	6.1342	97.28	69.72
Ubiquitin receptor RAD23c	Q84L31	NODE_75058	6.131	82.61	94.44
Probable carboxylesterase 4, mitochondrial	Q9FX93	chain_15875	6.1282	16.11	63.64
Protein FIZZY-RELATED 1	Q8VZS9	NODE_26444	6.1255	75.34	74.07
Eukaryotic translation initiation factor 3 subunit I	Q38884	NODE_31854	6.1195	56.9	58.46
Probable 3-hydroxyisobutyrate dehydrogenase, mitochondrial	Q9SUC0	NODE_23017	6.119	89.66	64.94

continued on the next page

Contigs promoted at 34°C (*continued*)

Name	UniProt ID	Contig	$\log_2$ (fold-Change)	Target coverage (%)	Alignment identity (%)
At1g12570	Q66GI5	NODE_64585	6.1176	56.76	60.98
Transcription factor MYB74	Q9M0Y5	chain_16794	6.1135	89.09	89.69
FAD/NAD(P)-binding oxidoreductase family protein	Q9ZQN9	chain_52254	6.1099	49.23	67.74
60S ribosomal protein L10-3	Q93W22	NODE_13556	6.1063	94.77	77.08
ADP-ribosylation factor B1C	Q9M9N1	NODE_20496	6.1013	98.68	68.8
Vicilin-like seed storage protein At2g18540	F4IQK5	NODE_37176	6.094	95.24	50.65
Casein kinase II subunit alpha-4, chloroplastic	O64816	NODE_58	6.0905	44.94	88.57
Calcium-transporting ATPase 3, endoplasmic reticulum-type	Q9SY55	NODE_4924	6.0897	71.67	69.0
23.6 kDa heat shock protein, mitochondrial	Q96331	chain_4516	6.083	81.3	67.42
Probable phosphoglucomutase, cytoplasmic 2	Q9SGC1	NODE_30167	6.0828	85.25	79.27
Aquaporin TIP1-3	O82598	NODE_53497	6.0788	55.93	65.63
GDP-mannose 4,6 dehydratase 1	Q9SNY3	NODE_13145	6.0758	87.34	91.92
Ribosomal RNA small subunit methyltransferase J	Q93VT6	chain_9312	6.068	80.56	76.85
60S ribosomal protein L5-1	Q8LBI1	chain_9229	6.0655	62.62	67.91
Putative actin-5	Q8RYC2	NODE_4027	6.0543	98.94	80.97
Heat shock 70 kDa protein 5	Q9S9N1	chain_39057	6.0493	59.04	72.92
Cytosolic isocitrate dehydrogenase [NADP]	Q9SRZ6	chain_40311	6.0463	91.11	85.0
AT3g03330/T21P5_25	Q8L7U0	chain_15245	6.0462	31.19	66.67
LOB domain-containing protein 41	Q9M886	NODE_43579	6.0424	70.79	64.52
Ubiquitin-NEDD8-like protein RUB1	Q9SHE7	NODE_48004	6.042	84.52	76.32
Ferritin-1, chloroplastic	Q39101	chain_15080	6.0108	75.76	76.88
1,4-alpha-glucan-branching enzyme 2-2, chloroplastic/amyloplastic	Q9LZS3	NODE_53522	5.9997	64.1	87.5
ADP,ATP carrier protein 2, mitochondrial	P40941	chain_14201	5.9966	83.86	77.94
Ethylene-responsive transcription factor 13	Q8L9K1	chain_15602	5.9944	63.49	70.51
DEAD-box ATP-dependent RNA helicase 40	Q9SQV1	NODE_34985	5.9881	45.28	63.64
Protein DETOXIFICATION 5	Q9SIA1	NODE_68287	5.9852	86.96	61.54

continued on the next page

Contigs promoted at 34°C (*continued*)

Name	UniProt ID	Contig	$\log_2$ (fold-Change)	Target coverage (%)	Alignment identity (%)
At1g75800/T4O12_2	Q9LQT4	chain_33509	5.9823	69.44	67.35
ARM repeat superfamily protein	Q8RWE7	chain_33173	5.9796	50.0	80.95
Probable sulfate transporter 3.5	Q94LW6	NODE_1055	5.9729	83.11	72.42
Ubiquitin domain-containing protein DSK2a	Q9SII9	NODE_59802	5.9711	45.33	66.67
Polyubiquitin 9	Q9FHQ6	chain_4094	5.9691	82.26	86.97
Ornithine aminotransferase, mitochondrial	Q9FNK4	NODE_44697	5.9676	51.85	70.37
3-methyl-2-oxobutanoate hydroxymethyltransferase 2, mitochondrial	Q9M315	chain_1674	5.9623	64.59	75.97
Probable carboxylesterase 17	Q9LFR7	chain_17577	5.9609	32.37	58.18
Methylmalonate-semialdehyde dehydrogenase [acylating], mitochondrial	Q0WM29	NODE_41869	5.9584	75.41	75.56
F-box protein At4g02760	Q9SY03	chain_31497	5.9561	19.25	67.8
17.6 kDa class I heat shock protein 2	Q9ZW31	NODE_14847	5.9536	60.0	70.19
40S ribosomal protein S13-2	P59224	NODE_19206	5.9445	98.7	77.48
Hexokinase-1	Q42525	chain_22725	5.9438	90.63	78.67
Nudix hydrolase 15, mitochondrial	Q8GYB1	chain_68867	5.9211	80.06	79.83
Protein disulfide isomerase-like 1-1	Q9XI01	NODE_21347	5.9207	22.75	75.68
17.6 kDa class I heat shock protein 2	Q9ZW31	chain_31574	5.92	95.45	78.57
60S ribosomal protein L8-3	Q42064	NODE_10699	5.9174	90.67	69.63
Phosphate transporter PHO1 homolog 9	Q9LJW0	NODE_60479	5.9165	98.31	66.67
Succinate dehydrogenase [ubiquinone] iron-sulfur subunit 3, mitochondrial	Q9FJP9	NODE_48290	5.9151	84.75	73.47
Cytochrome P450, family 89, subfamily A, polypeptide 3	F4K231	chain_20250	5.914	73.68	70.75
Serine/threonine-protein phosphatase PP1 isozyme 5	P48485	NODE_32253	5.9104	83.33	89.66
Calcineurin-like metallo-phosphoesterase superfamily protein	F4JMQ8	NODE_16421	5.9072	97.25	65.71
Obg-like ATPase 1	Q9SA73	chain_20042	5.902	87.93	74.0
Ethylene-responsive transcription factor 15	Q8VYM0	chain_34761	5.9008	39.44	70.37
Probable phospholipid hydroperoxide glutathione peroxidase 6, mitochondrial	O48646	chain_36640	5.9002	75.61	84.78

continued on the next page

Contigs promoted at 34°C (*continued*)

Name	UniProt ID	Contig	$\log_2$ (fold-Change)	Target coverage (%)	Alignment identity (%)
PAR1 protein	Q9FHC1	chain_13031	5.8984	36.36	82.61
Glycine-rich protein 23	O48848	NODE_60071	5.8954	67.19	57.14
NAD(P)H-quinone oxidoreductase subunit T, chloroplastic	Q9SMS0	chain_2942	5.8917	57.41	74.68
Peptidyl-prolyl cis-trans isomerase CYP18-4	Q42406	NODE_12378	5.8905	82.91	72.39
Pre-mRNA-splicing factor 38	Q8LB54	NODE_32087	5.886	57.75	66.33
Cullin-4	Q8LGH4	NODE_44111	5.882	94.38	56.63
Pumilio homolog 6, chloroplastic	Q9C5E7	chain_15812	5.878	55.1	84.62
Probable acylpyruvase FAHD1, mitochondrial	Q93ZE5	NODE_36213	5.877	62.75	60.32
Cytosolic enolase 3	Q9ZW34	NODE_24264	5.872	97.5	61.32
ARM repeat superfamily protein	F4JAY0	chain_34526	5.8704	40.0	60.0
1-aminocyclopropane-1-carboxylate oxidase 4	Q06588	chain_20586	5.8658	95.42	76.36
Chlorophyll a-b binding protein CP29.2, chloroplastic	Q9XF88	chain_60304	5.8654	63.64	85.0
Branched-chain-amino-acid aminotransferase 1, mitochondrial	Q93Y32	chain_21793	5.8637	87.27	70.21
ATPase, V1 complex, subunit B protein	F4JTQ0	NODE_22675	5.8576	94.23	76.03
Heat shock 70 kDa protein 18	Q9C7X7	NODE_21689	5.8543	69.05	71.43
Glutamine synthetase cytosolic isozyme 1-4	Q9FMD9	chain_19565	5.841	29.21	84.0
N-alpha-acetyltransferase MAK3	O80438	chain_4963	5.8369	37.93	100.0
Monooxygenase 3	Q9FLC2	chain_10930	5.8311	36.42	58.06
Phosphoinositide phospholipase C 7	Q9LY51	NODE_30083	5.8295	18.85	81.82
Beta-adaptin-like protein B	Q9SUS3	NODE_76507	5.8292	79.1	88.46
At3g27200	Q8VY59	chain_38658	5.8238	13.77	88.89
HXXXD-type acyl-transferase family protein	Q9MAP9	chain_15282	5.8202	71.05	64.94
Serine/threonine-protein phosphatase PP1 isozyme 9	Q9M9W3	chain_16413	5.82	85.0	78.79
Aldehyde dehydrogenase family 2 member B7, mitochondrial	Q8S528	NODE_30820	5.8101	90.0	66.36
Vacuolar-processing enzyme alpha-isozyme	P49047	NODE_71978	5.81	45.71	77.42
ATP synthase subunit beta-3, mitochondrial	Q9C5A9	NODE_2446	5.809	94.44	76.0

continued on the next page

Contigs promoted at 34°C (*continued*)

Name	UniProt ID	Contig	$\log_2$ (fold-Change)	Target coverage (%)	Alignment identity (%)
Protein mago nashi homolog	O23676	chain_2072	5.8085	86.71	86.76
Putative glutathione peroxidase 7, chloroplastic	Q9SZ54	NODE_35563	5.8084	94.2	62.5
Endochitinase EP3	Q9M2U5	chain_662	5.8063	98.39	85.0
Calcium-transporting ATPase 12, plasma membrane-type	Q9LY77	chain_2894	5.8017	89.72	82.76
Glycerate dehydrogenase HPR, peroxisomal	Q9C9W5	chain_57611	5.7998	79.17	88.89
Cathepsin B-like protease 3	Q94K85	NODE_30248	5.7996	36.07	69.77
ADP-ribosylation factor 3	P40940	NODE_44597	5.796	88.64	63.16
Asparagine synthetase [glutamine-hydrolyzing] 3	Q9LFU1	chain_16316	5.7922	95.22	83.33
Core-2/I-branching beta-1,6-N-acetylglucosaminyltransferase family protein	Q8GYH0	NODE_38873	5.7878	89.58	67.06
T-complex protein 1 subunit eta	Q9SF16	NODE_27289	5.7855	85.71	82.61
Elongation factor 1-delta 2	Q9SI20	NODE_11044	5.7849	26.34	61.9
Endoplasmin homolog	Q9STX5	NODE_41648	5.7842	31.52	75.0
Ras-related protein RABA4b	Q9SMQ6	NODE_36977	5.784	100	60.61
Histone H3-like 4	Q9LXU8	NODE_59877	5.7798	95.77	71.67
Katanin p60 ATPase-containing subunit A1	Q9SEX2	NODE_39042	5.7761	70.59	62.86
Autophagy-related protein 18a	Q93VB2	chain_23063	5.7711	75.25	54.67
60S ribosomal protein L11-1	P42795	chain_9749	5.764	96.49	67.89
Tubulin alpha-4 chain	Q0WV25	chain_9164	5.7609	85.46	81.91
Malate dehydrogenase 1, cytoplasmic	P93819	NODE_19156	5.7594	79.55	65.22
Glutamine synthetase cytosolic isozyme 1-4	Q9FMD9	chain_14883	5.7585	95.97	78.81
Putative DUF21 domain-containing protein At1g03270	Q9ZVS8	NODE_34757	5.7552	28.04	75.86
Pathogenesis-related protein 1	P33154	chain_14443	5.7533	42.03	59.65
V-type proton ATPase subunit B3	Q8W4E2	NODE_41402	5.7505	82.14	62.22
Glutamate dehydrogenase 1	Q43314	NODE_49028	5.7488	54.22	56.82
UDP-glycosyltransferase 75B2	Q9ZVY5	chain_4825	5.7458	77.27	63.77

continued on the next page

Contigs promoted at 34°C (*continued*)

Name	UniProt ID	Contig	$\log_2$ (fold-Change)	Target coverage (%)	Alignment identity (%)
2-oxoglutarate (2OG) and Fe(II)-dependent oxygenase superfamily protein	Q9LE86	chain_20067	5.7431	33.85	90.48
Flavonol synthase/flavanone 3-hydroxylase	Q96330	chain_10161	5.7388	54.94	65.09
Auxin-responsive GH3 family protein	F4II77	chain_8614	5.7312	98.69	68.22
Photosystem I reaction center subunit II-2, chloroplastic	Q9SA56	chain_55448	5.7297	61.82	92.54
V-type proton ATPase catalytic subunit A	O23654	NODE_21043	5.7258	100.0	90.12
TRF-like 3	A0A1P8ASC6	chain_62223	5.7193	39.75	67.74
Calnexin homolog 2	Q38798	NODE_52718	5.718	36.11	84.0
Autophagy-related protein 8d	Q9SL04	NODE_67460	5.7161	42.86	94.12
Nucleotide-sugar transporter family protein	F4JNE0	chain_11397	5.707	96.28	67.69
Histone H3-like 4	Q9LXU8	NODE_33874	5.7015	64.86	68.09
Putative WD-repeat protein	Q9LV27	chain_3385	5.7015	96.57	89.07
Pathogenesis-related thaumatin superfamily protein	Q9LNT0	NODE_59019	5.699	96.49	62.96
Glycine-rich protein DOT1	Q9SIH2	chain_9666	5.6969	47.62	52.66
Proteasome subunit alpha type-6-B	O81147	NODE_28233	5.6901	50.0	80.0
Peptidyl-prolyl cis-trans isomerase FKBP15-2	Q38936	NODE_14329	5.6886	46.72	57.14
AT-hook motif nuclear-localized protein 17	Q9LTA2	chain_20024	5.6842	35.59	85.0
Eukaryotic translation initiation factor 2 gamma subunit	O64490	NODE_10483	5.6839	98.98	74.28
Ubiquitin-40S ribosomal protein S27a-1	P59271	NODE_15449	5.6815	51.45	65.91
ABC transporter G family member 32	O81016	NODE_26483	5.678	35.38	95.45
Aspartic proteinase PCS1	Q9LZL3	NODE_41073	5.6675	90.32	70.37
Chaperone protein dnaJ 3	Q94AW8	chain_13025	5.6611	93.3	77.13
DEAD-box ATP-dependent RNA helicase 10	Q8GY84	NODE_62510	5.654	100	74.32
ATP synthase subunit alpha, mitochondrial	P92549	NODE_2074	5.6503	80.83	82.4
26S proteasome non-ATPase regulatory subunit 14 homolog	Q9LT08	NODE_37481	5.6502	73.85	91.49
Pathogenesis-related protein 1	P33154	chain_22550	5.6485	50.88	59.65

continued on the next page

Contigs promoted at 34°C (*continued*)

Name	UniProt ID	Contig	$\log_2$ (fold-Change)	Target coverage (%)	Alignment identity (%)
ATP-dependent zinc metalloprotease FTSH 3, mitochondrial	Q84WU8	NODE_36556	5.6468	50.0	63.16
60S ribosomal protein L31-3	P51420	chain_10941	5.6457	63.71	70.51
Adenosylhomocysteinase 2	Q9LK36	NODE_18133	5.6424	100.0	76.3
At1g65720/F1E22_13	Q9SHY3	chain_6776	5.6228	14.09	85.0
Receptor-like protein 20	Q9SKK5	chain_54720	5.6133	45.79	56.25
Protein transport protein Sec61 subunit beta	Q9FKK1	NODE_20057	5.6125	51.96	55.77
Probable disease resistance protein At5g66900	Q9FKZ1	NODE_22789	5.6119	41.98	61.67
Beta-glucosidase 17	O64882	chain_3955	5.6106	89.34	57.69
Probable cysteine protease RDL5	Q9SUS9	NODE_10862	5.609	76.0	71.15
Basic endochitinase B	P19171	chain_4918	5.609	91.56	76.62
Pathogenesis-related protein 1	P33154	NODE_15144	5.5967	73.4	70.07
40S ribosomal protein S29	Q680P8	NODE_30662	5.5902	94.83	72.22
E3 ubiquitin-protein ligase UPL3	Q6WWW4	NODE_39939	5.5896	31.58	68.97
Proteasome subunit beta type-5-B	Q9LIP2	chain_21891	5.5892	85.26	70.0
Elongation factor 1-alpha 2	Q8W4H7	chain_4538	5.5877	98.49	78.98
At2g15220/F15A23.4	Q9SKL6	NODE_41766	5.5874	100	80.43
Cytochrome b-c1 complex subunit Rieske-2, mitochondrial	Q9LYR2	NODE_26673	5.5822	81.51	75.58
Beta-glucosidase 16	Q9M1D0	chain_6829	5.5794	92.8	60.17
Elongation factor 1-alpha 2	Q8W4H7	chain_30984	5.5686	100.0	94.12
Probable galactinol–sucrose galactosyltransferase 6	Q8RX87	NODE_34182	5.5587	77.5	60.66
60S ribosomal protein L4-1	Q9SF40	chain_9594	5.5561	53.61	71.77
RNA polymerase II C-terminal domain phosphatase-like 2	Q5YDB5	chain_11628	5.5534	38.41	82.46
60S ribosomal protein L3-2	P22738	NODE_5330	5.551	95.83	70.7
Acidic endochitinase	P19172	chain_42051	5.5503	48.81	75.0
GTP-binding nuclear protein Ran-1	P41916	NODE_9918	5.5464	84.15	75.73
40S ribosomal protein S16-1	Q9SK22	NODE_21600	5.5435	92.62	74.8

continued on the next page

Contigs promoted at 34°C (*continued*)

Name	UniProt ID	Contig	$\log_2$ (fold-Change)	Target coverage (%)	Alignment identity (%)
Leucine aminopeptidase 3, chloroplastic	Q8RX72	NODE_46029	5.5417	40.7	67.65
Cytochrome P450 82C3	O49396	chain_70043	5.541	83.17	65.38
60S ribosomal protein L38	O22860	NODE_29285	5.5391	91.78	81.82
Beta carbonic anhydrase 2, chloroplastic	P42737	chain_4101	5.5384	90.14	58.73
Serine/arginine-rich-splicing factor SR34	O22315	chain_43491	5.5286	63.44	60.34
26S proteasome regulatory subunit 10B homolog A	Q9SEI3	NODE_69783	5.5278	78.69	76.6
60S ribosomal protein L13a-3	Q9SVR0	NODE_15473	5.5264	65.88	69.57
AT5g10910/T30N20_180	Q9LEU9	chain_8018	5.5264	84.42	81.82
S-adenosylmethionine decarboxylase proenzyme 1	Q96286	chain_57037	5.5209	94.2	76.56
Protein SGT1 homolog A	Q9SUR9	NODE_42152	5.5186	60.0	71.88
E3 ubiquitin-protein ligase UPL2	Q8H0T4	NODE_58073	5.5185	82.5	65.63
Protein kinase superfamily protein	Q9C5K4	chain_58599	5.5179	61.11	100.0
AT5g12010/F14F18_180	Q9LYH2	chain_4344	5.5176	72.48	76.51
60S ribosomal protein L14-1	Q9SIM4	NODE_33725	5.5133	68.42	63.16
60S ribosomal protein L21-2	Q9FDZ9	NODE_19176	5.5084	68.24	56.52
At1g65020	Q5XF12	NODE_69773	5.5069	80.0	63.27
Tyrosine decarboxylase 1	Q8RY79	chain_4653	5.5009	53.83	79.68
2-oxoglutarate (2OG) and Fe(II)-dependent oxygenase superfamily protein	Q84K82	chain_2076	5.498	69.5	60.34
GLYCINE RICH PROTEIN 9	F4IH98	chain_3720	5.4978	41.8	56.0
Beta carbonic anhydrase 2, chloroplastic	P42737	NODE_41117	5.4933	89.36	65.85
26S proteasome regulatory subunit 4 homolog A	Q9SZD4	NODE_7717	5.4845	97.14	83.03
Lysine–tRNA ligase, cytoplasmic	Q9ZPI1	NODE_30181	5.4696	67.65	64.44
Alpha/beta-Hydrolases superfamily protein	A0A1P8B9I4	chain_10823	5.4653	14.88	65.38
Putative 60S ribosomal protein L13-2	Q9SMT4	chain_15859	5.4546	94.36	77.6
Aconitate hydratase 2, mitochondrial	Q94A28	NODE_13228	5.4518	32.17	55.56

continued on the next page

Contigs promoted at 34°C (*continued*)

Name	UniProt ID	Contig	$\log_2$ (fold-Change)	Target coverage (%)	Alignment identity (%)
26S proteasome regulatory subunit 8 homolog A	Q9C5U3	NODE_18710	5.4478	100	85.37
Ubiquitin carboxyl-terminal hydrolase 6	Q949Y0	chain_16107	5.4428	60.19	59.02
Pre-mRNA-splicing factor 38	Q8LB54	NODE_29688	5.4415	43.82	67.51
Patatin-like protein 2	O48723	NODE_25676	5.4385	75.0	78.22
MDIS1-interacting receptor like kinase 1	Q9M0G7	NODE_35118	5.4324	38.1	58.97
Glucosyltransferase-like protein (Fragment)	W8PV66	chain_9290	5.4299	29.71	67.5
G-type lectin S-receptor-like serine/threonine-protein kinase At5g24080	Q9FLV4	NODE_16250	5.4298	57.14	75.68
Cullin-associated NEDD8-dissociated protein 1	Q8L5Y6	chain_19628	5.4259	42.39	63.16
Chaperone protein ClpD, chloroplastic	P42762	NODE_72000	5.4222	98.55	83.58
Polyubiquitin 9	Q9FHQ6	chain_69659	5.4149	42.11	90.0
DEAD-box ATP-dependent RNA helicase 21	P93008	chain_17940	5.4104	50.0	73.08
Sucrose synthase 6	Q9FX32	NODE_20030	5.4103	89.73	61.59
Proteasome subunit alpha type	Q2V3Q0	NODE_22835	5.4032	88.97	74.77
Aconitate hydratase 3, mitochondrial	Q9SIB9	chain_43507	5.4011	40.38	85.0
WD-40 repeat-containing protein MSI2	O22468	chain_40074	5.3978	86.21	79.71
Probable mediator of RNA polymerase II transcription subunit 37c	P22954	NODE_8657	5.3949	93.97	64.94
Heat shock 70 kDa protein 9, mitochondrial	Q8GUM2	NODE_61019	5.3945	84.29	62.07
NAD(P)-binding Rossmann-fold superfamily protein	A0A1I9LNT2	NODE_65557	5.3914	50.68	61.11
Ankyrin repeat family protein	Q9LSB0	chain_5325	5.3895	26.47	67.97
Chaperonin CPN60-like 2, mitochondrial	Q93ZM7	NODE_44114	5.3878	90.48	72.97
Proteasome subunit beta type-7-B	Q7DLS1	NODE_22790	5.3803	33.74	59.26
UDP-glycosyltransferase 75B2	Q9ZVY5	chain_3400	5.3787	56.2	64.9
Eukaryotic translation initiation factor 3 subunit C	O49160	NODE_42430	5.3697	58.62	63.64
Succinate dehydrogenase [ubiquinone] flavoprotein subunit 2, mitochondrial	Q9ZPX5	chain_14668	5.3692	41.38	70.21
Tryptophan synthase	F4K727	NODE_27956	5.3553	94.12	76.6

continued on the next page

Contigs promoted at 34°C (*continued*)

Name	UniProt ID	Contig	$\log_2$ (fold-Change)	Target coverage (%)	Alignment identity (%)
Heat shock 70 kDa protein 5	Q9S9N1	chain_9449	5.3515	94.75	79.55
3-hydroxyisobutyryl-CoA hydrolase-like protein 2, mitochondrial	Q8RXN4	NODE_14941	5.3444	66.67	68.66
Heat shock 70 kDa protein 5	Q9S9N1	chain_1029	5.3341	83.6	83.78
Senescence-associated protein 13	Q9ZW18	NODE_38324	5.3255	56.67	81.82
E3 ubiquitin-protein ligase UPL4	Q9LYZ7	chain_14324	5.3244	85.62	78.26
Putative EG45-like domain containing protein 1	Q9M0C2	NODE_51209	5.3195	48.15	70.27
Ras-related protein RABA1i	Q9S810	NODE_27758	5.3035	98.95	81.72
Tubulin beta-3 chain	Q9ASR0	chain_6471	5.3024	94.25	86.39
S-adenosylmethionine synthase 3	Q9SJL8	NODE_27306	5.3008	68.38	65.82
Polyubiquitin 10	Q8H159	NODE_4716	5.2947	25.18	57.81
Hypersensitive-induced response protein 1	Q9FM19	chain_14758	5.2937	85.48	89.26
60S ribosomal protein L27-2	Q8LCL3	NODE_20332	5.2921	36.69	56.0
Probable disease resistance protein At5g66900	Q9FKZ1	chain_31329	5.2809	77.16	58.87
UDP-glycosyltransferase 74E1	P0C7P7	NODE_33296	5.2715	100	51.82
Nucleoside diphosphate kinase IV, chloroplastic/mitochondrial	Q8LAH8	chain_10548	5.2704	78.16	66.4
Cytochrome P450 82G1	Q9LSF8	NODE_63721	5.2685	79.69	66.0
Acidic endochitinase	P19172	chain_16046	5.2657	82.68	74.39
Peroxidase 9	Q96512	NODE_26596	5.255	98.08	62.65
Glycine-rich protein family	Q9SFY8	chain_15050	5.2544	27.0	52.54
ADP,ATP carrier protein 2, mitochondrial	P40941	chain_8607	5.2506	83.86	77.94
T-complex protein 1 subunit gamma	Q84WV1	NODE_45722	5.2499	46.81	80.95
Leucine-rich repeat extensin-like protein 3	Q9T0K5	chain_35960	5.2492	73.33	65.63
Nuclear poly(A) polymerase 4	Q8VYW1	chain_12272	5.2463	75.79	63.38
(E,E)-geranyllinalool synthase	Q93YV0	NODE_31421	5.245	18.8	80.95
NDR1/HIN1-like protein 3	Q9FNH6	NODE_13867	5.2376	75.36	65.91
Cytochrome P450 71B35	Q9LIP5	chain_12957	5.2166	92.31	63.33

continued on the next page

Contigs promoted at 34°C (*continued*)

Name	UniProt ID	Contig	$\log_2$ (fold-Change)	Target coverage (%)	Alignment identity (%)
Barwin-related endoglucanase	A0A1P8B4P8	NODE_4727	5.2129	67.27	66.2
ATP-dependent zinc metalloprotease FTSH 5, chloroplastic	Q9FH02	chain_12242	5.2045	69.46	96.43
At2g15220/F15A23.4	Q9SKL6	chain_46692	5.2028	97.37	85.71
Ubiquitin-conjugating enzyme E2 3	P42746	NODE_24553	5.2001	98.47	76.56
Dynamin-related protein 1B	Q84XF3	NODE_10724	5.1996	93.15	85.22
Patatin-like protein 2	O48723	chain_15369	5.1911	94.12	68.87
40S ribosomal protein S20-2	Q9STY6	NODE_20457	5.1844	79.84	74.51
Probable glucan endo-1,3-beta-glucosidase BG3	F4J270	chain_22841	5.1823	49.32	71.43
40S ribosomal protein S19-1	Q9SGA6	NODE_17493	5.1811	43.44	61.54
Putative Sar1 GTP binding protein	Q8VYP7	NODE_28467	5.1798	40.38	90.0
Probable cysteine protease RD19B	P43295	NODE_25407	5.1768	45.28	86.96
60S ribosomal protein L7-4	Q9LHP1	NODE_55848	5.1724	100.0	72.58
Multiprotein-bridging factor 1a	Q9SJI8	chain_17411	5.1695	92.0	57.58
Endochitinase EP3	Q9M2U5	NODE_32965	5.1657	70.41	64.71
Translationally controlled tumor protein 2	Q9M9V9	chain_41620	5.1553	94.44	79.38
Homeobox-leucine zipper protein ATHB-12	Q9M276	NODE_7979	5.1549	40.17	58.06
40S ribosomal protein Sa-1	Q08682	NODE_14172	5.1524	42.15	67.74
Probable glutathione peroxidase 5	Q9LYB4	NODE_31782	5.152	62.93	66.18
At5g10830	Q9LEV6	NODE_10419	5.1518	95.02	67.22
40S ribosomal protein S15a-4	Q9LX88	NODE_17554	5.1505	97.04	82.35
40S ribosomal protein S2-1	Q8L8Y0	NODE_10755	5.1484	86.91	73.95
At2g16940	Q9ZVW9	chain_47656	5.1461	75.94	86.09
Endochitinase At2g43580	O24598	chain_1070	5.1407	83.89	68.47
Receptor for activated C kinase 1B	Q9C4Z6	NODE_8224	5.1359	86.02	71.11
Putative oxygen-evolving enhancer protein 2-2	O49344	chain_61120	5.1353	75.86	80.95
Protein MOTHER of FT and TFL1	Q9XFK7	chain_21886	5.1312	15.05	70.37

continued on the next page

Contigs promoted at 34°C (*continued*)

Name	UniProt ID	Contig	$\log_2$ (fold-Change)	Target coverage (%)	Alignment identity (%)
Ribulose bisphosphate carboxylase small chain 3B, chloroplastic	P10798	chain_12104	5.1222	58.11	84.62
40S ribosomal protein S17-3	Q9SQZ1	NODE_60987	5.1161	81.4	73.53
Dynein light chain	Q84VW0	NODE_25672	5.1091	86.14	69.77
Probable galactinol–sucrose galactosyltransferase 6	Q8RX87	NODE_35837	5.1042	67.31	79.03
Protein phosphatase 2C 3	Q9LNW3	NODE_21416	5.1029	12.94	80.95
Purple acid phosphatase 22	Q8S340	NODE_31040	5.098	94.83	73.39
Trans-cinnamate 4-monooxygenase	P92994	chain_11285	5.096	60.34	85.29
ATP synthase subunit 9, mitochondrial	Q304C3	chain_10002	5.0885	62.28	68.57
Importin subunit beta-1	Q9FJD4	NODE_24143	5.0733	70.97	80.95
Heat shock protein 21, chloroplastic	P31170	NODE_8984	5.0729	70.54	82.86
Serine carboxypeptidase-like 50	Q9M9Q6	chain_17460	5.0672	76.28	67.71
26S proteasome regulatory subunit 6A homolog B	O04019	chain_19084	5.0669	90.1	86.67
Guanine nucleotide-binding protein subunit beta	P49177	chain_33902	5.0662	75.47	61.54
Peroxidase 5	Q9M9Q9	NODE_15103	5.0631	86.16	58.33
At2g15220/F15A23.4	Q9SKL6	chain_66764	5.0611	83.02	67.44
Prohibitin-4, mitochondrial	Q9LK25	NODE_23954	5.0602	71.76	58.7
RNA-binding (RRM/RBD/RNP motifs) family protein	A0A2H1ZEL7	NODE_40561	5.056	66.67	64.76
Monooxygenase 3	Q9FLC2	chain_41905	5.0544	34.81	56.45
Nudix hydrolase 15, mitochondrial	Q8GYB1	chain_54972	5.0508	80.06	79.83
Late embryogenesis abundant protein (LEA) family protein	F4IYB7	chain_4082	5.0483	90.68	77.24
60S ribosomal protein L13-1	P41127	NODE_14726	5.0478	56.1	58.82
40S ribosomal protein S6-2	P51430	NODE_11106	5.0321	70.36	66.67
Cysteine-rich/transmembrane domain A-like protein	Q9SKY1	chain_7100	5.03	80.0	80.39
F21O3.6 protein	Q9SRT1	NODE_25566	5.0288	64.83	64.52
Tropinone reductase homolog At2g29320	Q9ZW15	chain_7891	5.0211	93.96	73.68
Beta-glucosidase 17	O64882	chain_24808	5.0127	65.99	66.67

continued on the next page

Contigs promoted at 34°C (*continued*)

Name	UniProt ID	Contig	$\log_2$ (fold-Change)	Target coverage (%)	Alignment identity (%)
Nudix hydrolase 15, mitochondrial	Q8GYB1	chain_3123	5.0019	80.06	79.83
Leucine-rich repeat extensin-like protein 6	Q9LUI1	NODE_16866	5.0013	88.68	56.52
AP-2 complex subunit mu	O23140	NODE_57047	4.9956	79.22	61.67
WW domain-containing protein	Q67ZZ1	NODE_16106	4.989	32.39	100.0
3-methyl-2-oxobutanoate hydroxymethyltransferase 2, mitochondrial	Q9M315	chain_36635	4.9865	47.62	76.27
50S ribosomal protein L3-1, chloroplastic	Q9SKX4	chain_48537	4.9797	75.0	78.26
60S ribosomal protein L15-2	Q8VYF1	chain_9158	4.9658	85.56	69.78
At2g15220/F15A23.4	Q9SKL6	chain_3028	4.9644	67.68	70.63
(E,E)-geranyllinalool synthase	Q93YV0	NODE_54432	4.9638	68.35	71.7
O-Glycosyl hydrolases family 17 protein	F4JVS0	chain_3351	4.9634	78.15	71.18
At5g60530/muf9_180	Q9FKJ4	NODE_16108	4.9588	51.54	58.47
Probable L-gulonolactone oxidase 4	Q9FM82	NODE_61661	4.9587	100.0	72.6
Polyadenylate-binding protein 8	Q9FXA2	NODE_4034	4.9543	25.48	56.92
Wall-associated receptor kinase-like 4	Q9S9M2	chain_13641	4.9528	31.82	70.37
Iron-sulfur cluster assembly protein 3	O81433	NODE_43819	4.9492	85.53	68.75
3-methyl-2-oxobutanoate hydroxymethyltransferase 2, mitochondrial	Q9M315	chain_20987	4.9342	56.73	73.68
40S ribosomal protein S23-1	Q9SF35	NODE_18889	4.9341	94.74	78.01
LOB domain-containing protein 25	Q8L8Q3	NODE_9647	4.9286	44.86	62.07
Eukaryotic translation initiation factor 3 subunit D	Q9FKV6	NODE_31030	4.9231	53.57	63.64
Probable leucine-rich repeat receptor-like serine/threonine-protein kinase At3g14840	C0LGN2	chain_39812	4.9206	70.83	63.64
Serine carboxypeptidase-like 45	Q93Y09	chain_6445	4.9147	84.26	73.33
Cystathionine gamma-synthase 1, chloroplastic	P55217	chain_6448	4.9133	27.06	81.82
Nascent polypeptide-associated complex subunit alpha-like protein 4	Q9SZY1	NODE_11489	4.9088	75.34	65.89
60S ribosomal protein L18a-3	Q9LUD4	NODE_14699	4.9073	43.17	56.41

continued on the next page

Contigs promoted at 34°C (*continued*)

Name	UniProt ID	Contig	$\log_2$ (fold-Change)	Target coverage (%)	Alignment identity (%)
Beta carbonic anhydrase 2, chloroplastic	P42737	chain_15694	4.905	56.43	65.38
40S ribosomal protein S11-3	P42733	NODE_10321	4.9011	66.25	70.3
Microtubule-associated protein RP/EB family member 1B	Q9FJJ5	chain_15097	4.8989	23.73	74.07
GDSL esterase/lipase At3g26430	Q9LIN2	NODE_29283	4.8888	52.69	64.58
60S ribosomal protein L26-1	P51414	NODE_18289	4.8866	83.56	71.43
Magnesium-chelatase subunit ChlH, chloroplastic	Q9FNB0	NODE_55832	4.8816	38.98	81.82
60S ribosomal protein L19-1	Q9SRX2	NODE_17195	4.8786	86.29	69.33
Cystathionine gamma-synthase 1, chloroplastic	P55217	chain_40148	4.878	27.06	81.82
60S ribosomal protein L10a-1	Q8VZB9	NODE_6837	4.875	99.46	72.83
Mitochondrial phosphate carrier protein 2, mitochondrial	Q9M2Z8	NODE_13595	4.8724	74.77	67.11
ER protein carbohydrate-binding protein	F4J800	chain_54943	4.8669	13.02	80.95
MDIS1-interacting receptor like kinase 2	Q8VZG8	NODE_63940	4.8653	54.17	80.0
At3g57450	Q9SCM7	NODE_18812	4.8603	50.57	87.18
Peroxidase 52	Q9FLC0	chain_16614	4.8577	73.81	78.67
40S ribosomal protein S5-1	Q9ZUT9	NODE_13024	4.8568	84.07	81.28
Heat shock 70 kDa protein 6, chloroplastic	Q9STW6	chain_30395	4.8543	60.42	82.14
Calcium-dependent protein kinase 20	Q9ZV15	chain_37735	4.8515	91.67	64.81
Patatin-like protein 1	O23179	NODE_25338	4.8371	70.8	62.5
Cytochrome P450 82C4	Q9SZ46	chain_289	4.837	28.95	65.63
Alpha-N-acetylglucosaminidase	Q9FNA3	NODE_50898	4.8359	64.38	73.68
Pathogenesis-related thaumatin superfamily protein	Q1PFD2	chain_64409	4.834	52.24	61.02
Geranylgeranyl diphosphate reductase, chloroplastic	Q9CA67	chain_34812	4.8308	58.18	90.32
2-Cys peroxiredoxin BAS1-like, chloroplastic	Q9C5R8	NODE_16526	4.8272	88.89	65.47
Tryptophan synthase	F4K727	chain_5812	4.8264	42.17	70.59
Cystathionine gamma-synthase 1, chloroplastic	P55217	chain_50086	4.824	26.74	81.82
Probable glucan endo-1,3-beta-glucosidase BG1	Q9M2M0	NODE_15951	4.8099	88.04	65.79

continued on the next page

Contigs promoted at 34°C (*continued*)

Name	UniProt ID	Contig	$\log_2$ (fold-Change)	Target coverage (%)	Alignment identity (%)
18.1 kDa class I heat shock protein	P19037	chain_30509	4.8074	31.15	88.89
CAP (Cysteine-rich secretory proteins, Antigen 5, and Pathogenesis-related 1 protein) superfamily protein	F4JZQ4	chain_9438	4.8038	76.47	60.53
Plant basic secretory protein (BSP) family protein	Q9ZUJ8	chain_3936	4.7939	67.27	80.56
Endonuclease 2	Q9C9G4	NODE_19987	4.792	55.38	76.92
Elongation factor Tu, chloroplastic	P17745	NODE_26107	4.7854	48.78	94.74
CAP (Cysteine-rich secretory proteins, Antigen 5, and Pathogenesis-related 1 protein) superfamily protein	Q9LPM6	NODE_63018	4.7825	51.35	67.57
40S ribosomal protein S26-3	Q9LYK9	NODE_21327	4.7822	80.95	71.29
COBRA-like protein 2	Q8L8Q7	chain_70062	4.7786	85.07	82.14
ER protein carbohydrate-binding protein	F4J800	chain_15651	4.7757	9.51	75.0
Citrate synthase 4, mitochondrial	P20115	chain_3305	4.7755	98.92	86.37
Peroxidase 67	Q9LVL2	NODE_5579	4.7751	82.4	69.85
Ethylene-responsive transcription factor ERF107	Q9FKG2	chain_12474	4.7738	26.89	70.49
Basic endochitinase B	P19171	chain_12586	4.7711	90.0	76.26
Pyruvate dehydrogenase E1 component subunit beta-1, mitochondrial	Q38799	NODE_45182	4.7583	57.83	72.34
Fructose-bisphosphate aldolase 5, cytosolic	O65581	NODE_12008	4.758	12.71	90.91
40S ribosomal protein S15-5	Q9FIX6	NODE_20453	4.7512	99.24	69.77
Endoplasmin homolog	Q9STX5	NODE_29001	4.7505	75.86	80.95
GDSL esterase/lipase 1	Q9FLN0	chain_2051	4.7498	49.34	67.07
Serine hydroxymethyltransferase 6	Q9LM59	NODE_35616	4.7482	87.1	62.26
Tropinone reductase homolog At2g29360	Q9ZW19	NODE_45161	4.7437	96.36	66.67
At4g29227	Q944J2	NODE_19929	4.7428	98.61	73.05
At2g14110	Q6IDA8	NODE_29728	4.7395	59.82	69.7
Quinone oxidoreductase-like protein	Q9LFK5	NODE_39359	4.7244	87.37	81.71
Probable calcium-binding protein CML45	Q9MBG5	NODE_23559	4.7234	57.48	65.28
Cytochrome c-2	Q9T0G2	NODE_15299	4.7194	90.99	69.74

continued on the next page

Contigs promoted at 34°C (*continued*)

Name	UniProt ID	Contig	$\log_2$ (fold-Change)	Target coverage (%)	Alignment identity (%)
Cysteine-rich/transmembrane domain PCC1-like protein	Q9LHJ3	NODE_29607	4.7149	34.85	81.82
UDP-glucuronic acid decarboxylase 3	Q9FIE8	NODE_25715	4.7009	43.9	94.12
26S proteasome regulatory subunit 6B homolog	Q9SEI4	chain_17329	4.6904	85.19	90.91
Histone H2B.9	Q9LZ45	chain_12514	4.6815	72.13	75.86
Prohibitin-5, mitochondrial	Q9LY99	NODE_51058	4.6736	68.29	58.18
Ethylene-responsive transcription factor ERF113	Q9LYU3	NODE_17988	4.6711	37.63	87.5
2-oxoglutarate (2OG) and Fe(II)-dependent oxygenase superfamily protein	Q9SYM7	chain_13556	4.6689	56.41	64.12
Protein NRT1/ PTR FAMILY 2.9	Q9M9V7	chain_9037	4.665	96.97	69.09
Tryptophan N-monooxygenase 1	O81346	chain_10901	4.6583	85.37	66.67
40S ribosomal protein S18	P34788	NODE_9092	4.6578	98.7	75.5
Pathogenesis-related protein 1	P33154	chain_30784	4.6563	93.55	58.14
At1g75800/T4O12_2	Q9LQT4	chain_17910	4.6528	71.23	64.67
Leucine-rich repeat transmembrane protein kinase	F4HRH4	chain_51296	4.6482	89.52	59.14
Glutamine synthetase cytosolic isozyme 1-4	Q9FMD9	chain_24783	4.6458	99.44	87.08
Allyl alcohol dehydrogenase, putative	Q9C677	chain_24077	4.642	96.88	76.67
Aquaporin PIP2-2	P43287	chain_14577	4.6366	86.47	81.66
Probable glucan endo-1,3-beta-glucosidase BG3	F4J270	chain_37912	4.634	92.02	69.6
Sucrose synthase 6	Q9FX32	NODE_8200	4.6309	92.58	75.0
Probable calcium-binding protein CML50	Q9FYE4	NODE_20997	4.6301	11.39	94.12
Basic helix-loop-helix (BHLH) DNA-binding superfamily protein	F4KAJ4	chain_10522	4.6301	81.68	77.86
Receptor serine/threonine kinase	F4I5D4	chain_8706	4.6237	27.47	75.0
2-oxoglutarate (2OG) and Fe(II)-dependent oxygenase superfamily protein	Q8LF12	NODE_15011	4.62	52.94	62.79
NAD(P)-binding Rossmann-fold superfamily protein	O80924	chain_66477	4.6159	39.19	78.57
Glutathione S-transferase U22	Q8GYM1	NODE_37383	4.6129	91.0	55.68
Nudix hydrolase 15, mitochondrial	Q8GYB1	chain_1849	4.6107	80.06	79.83

continued on the next page

Contigs promoted at 34°C (*continued*)

Name	UniProt ID	Contig	$\log_2$ (fold-Change)	Target coverage (%)	Alignment identity (%)
Histone H4	P59259	NODE_16470	4.6103	97.67	96.39
F11M21.26 protein	Q9ZWA3	NODE_24874	4.6102	97.92	67.39
Glutathione S-transferase U24	Q9SHH6	NODE_7686	4.6098	100.0	66.98
28 kDa heat/acid-stable phosphoprotein-like protein	Q9FNM0	NODE_43573	4.6084	45.71	80.65
NEP1-interacting protein 1	Q8GT75	NODE_12247	4.6032	66.67	65.22
Glycine-rich protein family	Q9SL09	NODE_17625	4.5937	66.49	54.87
Polyadenylate-binding protein 8	Q9FXA2	chain_10994	4.5909	93.95	66.14
Histone-lysine N-methyltransferase ATXR3	O23372	NODE_17442	4.5774	78.79	80.0
Polyubiquitin 8	Q39256	chain_23407	4.5759	52.35	78.21
Endoplasmic reticulum oxidoreductin-1	Q9C7S7	NODE_42075	4.5753	35.48	80.95
AT1G15380 protein	Q9XI31	NODE_17911	4.5728	76.73	73.73
Ankyrin repeat family protein	Q9LSB0	NODE_20608	4.5695	13.89	79.17
2-Cys peroxiredoxin BAS1, chloroplastic	Q96291	NODE_8263	4.5618	64.03	64.38
Ubiquitin-conjugating enzyme E2 13	Q42541	NODE_24200	4.5615	55.64	58.9
Dehydrin Xero 1	P25863	chain_2340	4.5489	19.15	100.0
3-methyl-2-oxobutanoate hydroxymethyltransferase 2, mitochondrial	Q9M315	chain_63718	4.5486	85.11	87.18
E3 ubiquitin-protein ligase PUB22	Q9SVC6	NODE_5873	4.5446	93.92	70.54
tRNA/rRNA methyltransferase (SpoU) family protein	F4JP86	chain_35654	4.5433	44.07	96.0
Rac-like GTP-binding protein ARAC4	Q38919	chain_5538	4.5418	95.07	84.38
Obg-like ATPase 1	Q9SA73	chain_9097	4.5358	39.29	90.63
NAC domain-containing protein 90	Q9FMR3	NODE_10199	4.5328	62.28	68.39
Aldose 1-epimerase	Q9LVH6	chain_70920	4.5238	89.81	74.66
ATPase family AAA domain-containing protein FIGL1	F4JEX5	NODE_72554	4.5235	98.55	76.12
Beta carbonic anhydrase 4	Q94CE4	chain_8272	4.5233	72.46	70.0
Peroxidase 4	Q9LE15	NODE_15853	4.5143	79.31	63.64

continued on the next page

Contigs promoted at 34°C (*continued*)

Name	UniProt ID	Contig	$\log_2$ (fold-Change)	Target coverage (%)	Alignment identity (%)
5-methyltetrahydropteroyltriglutamate–homocysteine methyltransferase 3, chloroplastic	Q0WNZ5	chain_4919	4.513	18.18	62.3
Mitochondrial substrate carrier family protein	Q9LY28	NODE_25395	4.5109	97.74	81.4
Heat shock 70 kDa protein 5	Q9S9N1	chain_10271	4.507	100	70.09
Chaperone protein dnaJ 10	Q8GYX8	NODE_36844	4.5067	33.96	94.12
Chaperone protein ClpD, chloroplastic	P42762	NODE_5768	4.5059	97.24	78.02
O-Glycosyl hydrolases family 17 protein	F4KH28	chain_9816	4.5057	84.69	65.47
AT4g02340 protein	O81299	NODE_17804	4.5041	79.87	71.43
Protein DETOXIFICATION 31	Q9LPV4	chain_16436	4.5035	95.16	75.13
At5g20110	Q6NM36	NODE_77392	4.5001	71.43	87.18
Sucrose synthase 2	Q00917	NODE_6457	4.4984	98.66	67.94
1-aminocyclopropane-1-carboxylate oxidase homolog 3	Q8H1S4	chain_11145	4.4905	100.0	68.12
Diacylglycerol kinase 6	F4JKI3	NODE_51287	4.4903	90.24	78.08
At5g55180	Q0V7P5	chain_10978	4.4778	95.36	71.1
BTB/POZ domain-containing protein At2g30600	Q8LEV3	chain_33883	4.4707	46.3	75.0
Dehydrin Xero 1	P25863	NODE_17488	4.4671	31.22	82.46
Alpha-dioxygenase 1	Q9SGH6	NODE_957	4.466	99.62	76.0
Beta-fructofuranosidase, insoluble isoenzyme CWINV1	Q43866	chain_2673	4.461	94.85	69.73
60S ribosomal protein L18-3	Q940B0	NODE_16804	4.4365	98.82	66.01
Calcium-binding EF-hand family protein	Q9T0I9	chain_7583	4.4357	78.2	72.73
At1g32690	Q6NNH5	chain_20150	4.4334	30.89	65.75
At2g15220/F15A23.4	Q9SKL6	chain_35116	4.429	68.37	70.63
Flavanone 3-hydroxylase	F4J3A5	NODE_67086	4.4241	85.19	77.27
2-Cys peroxiredoxin BAS1-like, chloroplastic	Q9C5R8	chain_60541	4.4224	39.66	95.45
Transcription factor bHLH126	Q9STJ6	NODE_32818	4.4223	41.96	71.74
3-methyl-2-oxobutanoate hydroxymethyltransferase 2, mitochondrial	Q9M315	chain_55252	4.4107	60.98	83.33

continued on the next page

Contigs promoted at 34°C (*continued*)

Name	UniProt ID	Contig	$\log_2$ (fold-Change)	Target coverage (%)	Alignment identity (%)
Actin cross-linking protein (DUF569)	Q9XIF7	NODE_71921	4.409	96.88	67.21
NDR1/HIN1-like protein 10	Q9SJ52	NODE_20629	4.4079	79.22	66.67
Obg-like ATPase 1	Q9SA73	chain_45892	4.4076	97.31	84.92
Trihelix transcription factor GT-3b	O80450	NODE_8009	4.4076	76.97	70.9
Shikimate kinase 1, chloroplastic	Q9SJ05	chain_9835	4.4019	71.03	69.33
Protein DMP5	Q9M897	NODE_23655	4.4006	79.09	62.79
Emb—CAB67623.1	Q9FII9	NODE_27160	4.4006	81.25	76.56
Calreticulin-3	O04153	NODE_16534	4.3975	79.28	88.0
Probable alpha,alpha-trehalose-phosphate synthase [UDP-forming] 9	Q9LRA7	NODE_14036	4.3971	99.59	74.18
At2g15220/F15A23.4	Q9SKL6	chain_23376	4.3899	77.36	72.5
GLYCINE RICH PROTEIN 9	Q9SL23	NODE_13762	4.3809	54.95	56.91
Glutathione S-transferase U25	Q9SHH7	chain_39794	4.3786	95.61	69.59
Ankyrin repeat family protein	Q9LSB0	NODE_4751	4.3752	42.36	60.0
Probable glucan endo-1,3-beta-glucosidase At4g16260	Q8VZJ2	chain_42442	4.3739	85.53	76.29
Photosystem II core complex proteins psbY, chloroplastic	O49347	chain_15118	4.3687	39.25	80.77
Ethylene-responsive transcription factor ERF017	Q84QC2	NODE_22917	4.356	53.09	82.35
ATP-dependent zinc metalloprotease FTSH 5, chloroplastic	Q9FH02	chain_39650	4.3532	22.92	95.24
Cupredoxin superfamily protein	A0A1P8B4N7	chain_10490	4.3496	86.27	74.6
Serine carboxypeptidase-like 19	Q8VZU3	chain_1789	4.347	74.06	62.07
Probable galacturonosyltransferase-like 9	O04536	NODE_5066	4.3269	84.67	75.44
Endochitinase At2g43590	O24658	NODE_16372	4.3232	81.97	67.35
Core-2/I-branching beta-1,6-N-acetylglucosaminyltransferase family protein	Q1G300	NODE_22215	4.315	22.16	61.11
Phenylalanine ammonia-lyase 2	P45724	NODE_935	4.3071	84.48	72.42
Nardilysin-like	F4HNU6	NODE_70979	4.3016	61.7	71.43

continued on the next page

Contigs promoted at 34°C (*continued*)

Name	UniProt ID	Contig	$\log_2$ (fold-Change)	Target coverage (%)	Alignment identity (%)
Kinase superfamily with octicosapeptide/Phox/Bem1p domain-containing protein	Q9SAJ2	chain_19484	4.3	32.79	89.47
Basic endochitinase B	P19171	NODE_38008	4.2955	73.74	73.61
Transmembrane protein	Q9LW52	chain_12593	4.2949	33.65	61.99
Probable mediator of RNA polymerase II transcription subunit 37c	Q9LHA8	NODE_7345	4.2924	96.75	87.36
Transcription regulator	F4HVV7	NODE_17720	4.289	58.76	81.55
ARM repeat superfamily protein	O64651	chain_22602	4.2886	34.02	71.88
Chlorophyll a-b binding protein 3, chloroplastic	Q8VZ87	chain_572	4.2797	43.4	90.91
Elongation factor G-1, mitochondrial	Q9C641	chain_19147	4.2754	42.86	80.77
Fe-S cluster assembly factor HCF101, chloroplastic	Q6STH5	chain_26327	4.2682	91.96	88.24
Nuclear pore complex protein GP210	F4KHD8	chain_17128	4.2678	90.16	72.22
Methylthioribose kinase	Q9C6D2	chain_15981	4.2666	69.9	72.06
Naringenin,2-oxoglutarate 3-dioxygenase	Q9S818	NODE_27999	4.256	31.43	90.48
Proteasome subunit beta type-6	Q8LD27	chain_10588	4.2553	90.24	92.76
60S ribosomal protein L36a	O23290	NODE_54819	4.2536	90.24	69.44
Uncharacterized protein At5g17350	Q9LF49	chain_24839	4.253	15.18	63.64
Protein ENDOSPERM DEFECTIVE 1	O80588	chain_5590	4.2327	28.04	75.86
Cathepsin B-like protease 3	Q94K85	NODE_6928	4.231	74.18	67.23
Ubiquitin-conjugating enzyme 36	F4I615	NODE_23955	4.2256	72.22	72.55
Cytochrome P450, family 722, subfamily A, polypeptide 1	F4HP86	NODE_24816	4.2124	77.04	54.37
Protein TIFY 9	Q93ZM9	chain_3703	4.1978	72.09	63.85
Ferritin-1, chloroplastic	Q39101	chain_546	4.1922	75.76	76.88
Probable plastid-lipid-associated protein 1, chloroplastic	O81439	chain_7961	4.1885	53.19	79.17
Heat shock protein 90-1	P27323	chain_13194	4.1818	96.28	90.4
Cytochrome P450 82G1	Q9LSF8	NODE_74342	4.1818	94.12	60.32
Polypyrimidine tract-binding protein homolog 2	Q9FGL9	chain_1884	4.1792	89.14	82.12

continued on the next page

Contigs promoted at 34°C (*continued*)

Name	UniProt ID	Contig	$\log_2$ (fold-Change)	Target coverage (%)	Alignment identity (%)
Serine carboxypeptidase-like 14	Q9C7D3	NODE_2486	4.1768	73.38	58.39
Probable 3-hydroxyisobutyrate dehydrogenase-like 1, mitochondrial	Q9SZE1	chain_38674	4.1713	80.0	82.09
Endochitinase EP3	Q9M2U5	chain_1106	4.1686	81.97	66.95
Beta-fructofuranosidase, insoluble isoenzyme CWINV3	Q67XZ3	chain_3626	4.1639	88.48	68.02
Glycine-rich protein DOT1	Q9SIH2	chain_449	4.16	30.13	52.21
Shaggy-related protein kinase alpha	P43288	NODE_68698	4.158	100	95.45
Probable alpha,alpha-trehalose-phosphate synthase [UDP-forming] 10	O80738	chain_7367	4.1514	94.32	69.33
Probable NEDD8-conjugating enzyme Ubc12-like	Q9ZU75	NODE_32273	4.1435	93.15	62.69
E3 ubiquitin-protein ligase PUB23	Q84TG3	NODE_29962	4.1435	93.4	65.31
3-methyl-2-oxobutanoate hydroxymethyltransferase 1, mitochondrial	O82357	chain_6152	4.1416	81.23	82.73
Emb—CAB72159.1	Q9FF51	NODE_26622	4.1368	85.92	68.33
Glycosyl hydrolase family protein with chitinase insertion domain-containing protein	F4JTY4	NODE_52059	4.1366	75.31	53.33
HXXXD-type acyl-transferase family protein	Q9FFE4	chain_9967	4.133	92.27	69.92
Protein SWEETIE	F4HRS2	chain_28893	4.1329	55.1	76.92
P-loop NTPase domain-containing protein LPA1 homolog 1	Q9FJH9	chain_13919	4.1322	69.12	95.65
Serine/arginine-rich splicing factor SR45	Q9SEE9	chain_2419	4.1284	68.27	57.64
Aldose 1-epimerase	Q9STT3	chain_938	4.1234	88.74	69.23
Transcription factor bHLH30	Q9S7Y1	NODE_44567	4.1048	43.18	64.86
AP2-like ethylene-responsive transcription factor At2g41710	Q8GWK2	chain_2860	4.1005	87.03	93.13
Endochitinase At2g43590	O24658	chain_9090	4.0916	87.23	65.76
Monothiol glutaredoxin-S1	Q9SA68	chain_68979	4.0795	52.25	71.74
Topless-related protein 3	Q84JM4	chain_18643	4.0753	92.0	86.36
Heat shock 70 kDa protein 8	Q9SKY8	NODE_2268	4.0746	81.17	82.33
Heat stress transcription factor A-1d	Q9LQM7	NODE_11362	4.0712	32.2	61.33

continued on the next page

Contigs promoted at 34°C (*continued*)

Name	UniProt ID	Contig	$\log_2$ (fold-Change)	Target coverage (%)	Alignment identity (%)
Receptor serine/threonine kinase	F4I5D4	chain_20261	4.0645	24.7	55.0
U-box domain-containing protein 45	Q9C7G1	NODE_4435	4.0595	28.68	67.12
40S ribosomal protein S28-1	Q9SR73	chain_53229	4.0581	93.85	82.46
Leucine-rich repeat transmembrane protein kinase	A0A1P8AQ09	chain_12310	4.0554	68.25	62.99
tRNase Z TRZ3, mitochondrial	Q8VYS2	NODE_51759	4.0457	64.2	62.75
Potassium transporter 1	O22397	NODE_8233	4.0427	36.59	52.81
Iron-sulfur cluster assembly protein 2	Q9MAB6	NODE_37394	4.0287	82.61	75.68
Short-chain dehydrogenase/reductase 2b	Q9ZUH5	NODE_38694	4.0273	46.39	68.18
Serine carboxypeptidase-like 18	Q9C7Z9	chain_3228	4.0137	45.01	58.6
3-oxo-Delta(4,5)-steroid 5-beta-reductase	Q9STX2	chain_4492	4.0124	68.0	75.25
AT1G15380 protein	Q9XI31	NODE_6209	4.0065	53.01	67.74
Osmotin-like protein OSM34	P50700	NODE_27209	3.9953	66.35	73.21
Glutathione S-transferase U8	Q9SR36	chain_11000	3.993	97.83	64.81
Beta carbonic anhydrase 2, chloroplastic	P42737	NODE_15653	3.9921	61.6	69.74
1-aminocyclopropane-1-carboxylate oxidase 4	Q06588	chain_67618	3.9873	58.33	79.03
Leucine-rich repeat receptor-like serine/threonine-protein kinase BAM2	Q9M2Z1	NODE_14944	3.986	71.64	55.32
Basic helix-loop-helix (BHLH) DNA-binding superfamily protein	F4KAJ4	chain_1688	3.9795	86.56	74.05
At2g15220/F15A23.4	Q9SKL6	chain_483	3.964	91.18	69.83
Probable amidase At4g34880	A0A1P8B760	chain_7245	3.9636	78.84	74.32
AT1G01800 protein	Q94K30	chain_3168	3.9635	94.38	66.02
Eukaryotic initiation factor 4A-2	P41377	chain_30129	3.9586	92.0	88.24
Transcription factor MYB102	Q9LDR8	NODE_4315	3.9509	61.07	82.85
AT4g02340 protein	O81299	chain_14210	3.9494	60.71	70.3
Phospho-2-dehydro-3-deoxyheptonate aldolase	Q9SK84	chain_69344	3.9423	13.73	95.0
At4g39830	O65670	NODE_17109	3.9362	63.83	75.86
Probable protein phosphatase 2C 78	Q9FIF5	NODE_2144	3.9348	75.41	80.28

continued on the next page

Contigs promoted at 34°C (*continued*)

Name	UniProt ID	Contig	$\log_2$ (fold-Change)	Target coverage (%)	Alignment identity (%)
Core-2/I-branching beta-1,6-N-acetylglucosaminyltransferase family protein	Q9M9C4	chain_8772	3.9286	97.61	70.63
Subtilisin-like serine endopeptidase family protein	A0A1P8B448	NODE_23177	3.926	76.12	62.37
Aconitate hydratase 3, mitochondrial	Q9SIB9	chain_11363	3.9258	99.65	89.25
Cytochrome P450 71A21	Q9STL2	NODE_4866	3.9167	89.2	64.03
Heavy metal transport/detoxification superfamily protein	F4KAE6	chain_10302	3.9097	94.74	72.73
Dehydrin Rab18	P30185	NODE_13367	3.9077	24.0	75.86
Probable protein phosphatase 2C 75	Q9FLI3	chain_6765	3.9027	59.72	74.01
Formate dehydrogenase, chloroplastic/mitochondrial	Q9S7E4	chain_4484	3.8925	52.69	90.41
E4 SUMO-protein ligase PIAL2	F4JYG0	NODE_3580	3.8918	94.01	63.12
Beta carbonic anhydrase 4	Q94CE4	NODE_29172	3.8892	56.41	85.71
L-type lectin-domain containing receptor kinase V.9	Q9SZD5	chain_16827	3.8866	98.11	62.75
Probable nucleoredoxin 1	O80763	chain_39423	3.884	68.84	65.96
Beta carbonic anhydrase 2, chloroplastic	P42737	chain_29587	3.8799	82.14	61.9
Late embryogenesis abundant (LEA) hydroxyproline-rich glycoprotein family	Q9M287	chain_52664	3.8797	38.71	76.27
Glycine-rich protein family	Q9FJS3	chain_60248	3.8763	68.47	54.84
Probable acyl-activating enzyme 2	Q9SEY5	NODE_19591	3.866	66.84	60.0
Respiratory burst oxidase homolog protein D	Q9FIJ0	chain_10069	3.8653	91.79	75.9
Eukaryotic aspartyl protease family protein	Q0WQ50	NODE_2957	3.8555	89.38	69.98
2-oxoglutarate (2OG) and Fe(II)-dependent oxygenase superfamily protein	Q9LIF4	chain_8414	3.8512	69.61	70.77
15.7 kDa heat shock protein, peroxisomal	Q9FHQ3	NODE_14214	3.849	78.34	78.38
At5g07330	Q9LY27	chain_9359	3.8446	64.33	64.52
Serine/threonine-protein kinase PCRK2	Q9LZF8	chain_29824	3.8443	26.56	73.47
Aquaporin TIP1-3	O82598	NODE_7587	3.8431	84.62	85.06
Glycine-rich protein 23	F4ITU2	chain_11451	3.8391	71.13	52.35
Cysteine protease XCP2	Q9LM66	chain_13079	3.834	68.87	76.39

continued on the next page

Contigs promoted at 34°C (*continued*)

Name	UniProt ID	Contig	$\log_2$ (fold-Change)	Target coverage (%)	Alignment identity (%)
Pathogenesis-related protein 5	P28493	chain_38294	3.8285	60.42	58.93
Protein REVEILLE 6	Q8H0W3	chain_741	3.8275	38.83	73.61
Chalcone synthase	P13114	chain_10722	3.8264	80.93	87.8
Calreticulin-3	O04153	NODE_44830	3.8261	82.22	71.43
NEP1-interacting protein 1	Q8GT75	NODE_12857	3.8212	34.74	66.15
Protein FD	Q84JK2	NODE_28342	3.8145	50.82	83.2
UDP-glycosyltransferase 87A2	O64733	chain_69541	3.8128	83.33	55.56
Trans-cinnamate 4-monooxygenase	P92994	NODE_21291	3.8106	94.22	85.8
Senescence-associated protein 13	Q9ZW18	chain_6255	3.81	88.02	77.61
Pyruvate kinase	Q9M057	chain_23872	3.8089	59.57	77.78
Berberine bridge enzyme-like 8	Q9SA85	NODE_1294	3.8047	84.44	71.72
4-coumarate–CoA ligase-like 7	Q9M0X9	chain_11377	3.804	95.45	75.81
ACT domain-containing protein ACR2	F4JWR0	NODE_10224	3.796	59.63	76.88
Leucoanthocyanidin dioxygenase	Q96323	chain_445	3.7948	91.84	86.57
O-fucosyltransferase 19	Q9SH89	NODE_46576	3.7946	34.43	85.0
Peptidyl-prolyl cis-trans isomerase CYP20-3, chloroplastic	P34791	chain_14080	3.7938	96.3	72.55
Berberine bridge enzyme-like 26	Q9FKU8	NODE_3674	3.7916	83.75	67.4
Eukaryotic aspartyl protease family protein	Q9C8C9	chain_8135	3.7906	80.98	70.76
ATP-citrate synthase alpha chain protein 1	Q9SGY2	chain_46371	3.7853	16.54	100.0
AT1G01800 protein	Q94K30	chain_7805	3.7787	93.17	66.01
Mediator of RNA polymerase II transcription subunit	Q9ZU37	NODE_24477	3.7784	46.1	56.25
Magnesium transporter MRS2-3	Q9LJN2	chain_7515	3.7744	90.91	86.38
Zinc finger protein ZAT11	Q9SLD4	NODE_17741	3.7721	43.16	74.58
Probable phospholipid-transporting ATPase 11	Q9SAF5	chain_12963	3.7697	100.0	75.34
Transcription factor MYB108	Q9LDE1	NODE_13201	3.766	54.65	84.93
Pyruvate dehydrogenase E1 component subunit beta-1, mitochondrial	Q38799	NODE_41158	3.7599	88.46	84.44

continued on the next page

Contigs promoted at 34°C (*continued*)

Name	UniProt ID	Contig	$\log_2$ (fold-Change)	Target coverage (%)	Alignment identity (%)
Carbon catabolite repressor protein 4 homolog 3	Q9LS39	chain_8826	3.7465	97.47	63.16
Histone H4	P59259	NODE_19132	3.7457	98.11	98.06
Acidic endochitinase	P19172	chain_54481	3.7439	44.05	77.78
F9L1.42 protein	Q9XI22	chain_32767	3.7433	56.41	100.0
17.6 kDa class I heat shock protein 1	Q9XIE3	NODE_75062	3.7427	60.0	78.95
Probable inactive poly [ADP-ribose] polymerase SRO2	Q9ZUD9	chain_19589	3.7372	57.94	64.55
At5g23590	Q8L7M3	chain_46588	3.737	75.48	67.48
Mechanosensitive ion channel protein 9	Q84M97	chain_14048	3.7307	83.52	71.64
At1g14180/F7A19_27	Q9XI69	chain_13558	3.7252	73.68	74.07
Plant basic secretory protein (BSP) family protein	Q9ZUJ8	chain_12589	3.7183	60.0	75.79
Clavaminate synthase-like protein At3g21360	Q9LIG0	chain_8251	3.7125	99.66	75.47
UDP-glycosyltransferase 91A1	Q940V3	NODE_34530	3.712	69.64	76.32
Histone H2B.3	Q9SI96	NODE_17744	3.7033	84.85	91.85
Ferritin-4, chloroplastic	Q9S756	chain_7320	3.6948	38.78	57.33
Trans-cinnamate 4-monooxygenase	P92994	NODE_63193	3.6888	40.82	100.0
Ribonuclease 3	P42815	NODE_11303	3.6874	80.31	65.13
Glucosidase-like protein	Q9SNC1	NODE_5351	3.6848	97.31	73.18
DNAJ heat shock N-terminal domain-containing protein	Q9LPU3	chain_2606	3.6843	92.21	90.0
NDR1/HIN1-like protein 6	Q8LD98	NODE_10914	3.6819	78.4	63.92
Potassium transporter 5	Q9M7K4	NODE_8849	3.6768	20.63	56.25
Protein EMSY-LIKE 4	Q08A72	chain_4471	3.6766	71.08	71.2
NDR1/HIN1-like protein 10	Q9SJ52	chain_15536	3.6756	64.54	68.6
Heat shock 70 kDa protein 5	Q9S9N1	chain_19109	3.6733	82.14	86.67
Putative calcium-transporting ATPase 13, plasma membrane-type	Q9LIK7	NODE_71150	3.6666	91.43	71.43
Probable aquaporin PIP1-5	Q8LAA6	chain_15857	3.6608	80.12	87.55

continued on the next page

Contigs promoted at 34°C (*continued*)

Name	UniProt ID	Contig	$\log_2$ (fold-Change)	Target coverage (%)	Alignment identity (%)
Quinoprotein amine dehydrogenase, beta chain-like RIC1-like guanyl-nucleotide exchange factor	F4JEA6	chain_12882	3.6553	78.06	75.42
Endonuclease 2	Q9C9G4	chain_27849	3.6505	70.99	79.8
Probable acyl-activating enzyme 18, peroxisomal	Q84P17	chain_13350	3.6474	96.5	79.23
Proteasome subunit beta type-6	Q8LD27	chain_16409	3.6468	90.24	92.76
Lipoxygenase 2, chloroplastic	P38418	chain_11237	3.644	92.97	73.73
Alpha/beta-Hydrolases superfamily protein	Q9M9W5	NODE_25955	3.6412	59.63	68.25
Cytochrome b5 isoform A	Q9FDW8	NODE_19599	3.6409	42.96	66.67
Pentatricopeptide repeat-containing protein At4g35130, chloroplastic	O49619	NODE_55437	3.6408	83.02	65.12
Late embryogenis abundant protein 41	Q39084	NODE_17470	3.6353	30.97	79.41
Tetratricopeptide repeat (TPR)-like superfamily protein	Q9SD65	NODE_3203	3.634	50.52	67.49
Cinnamyl alcohol dehydrogenase 8	Q02972	NODE_6562	3.6308	96.54	76.13
Heparanase-like protein 2	Q8L608	chain_2657	3.6303	93.78	69.83
Vitellogenin-like protein	Q9FH24	chain_45880	3.6134	30.49	75.0
Beta carbonic anhydrase 2, chloroplastic	P42737	chain_19348	3.61	72.0	62.86
Adenosylhomocysteinase 2	Q9LK36	NODE_23148	3.6029	68.35	70.97
Cysteine-rich receptor-like protein kinase 25	Q9M0X5	NODE_30998	3.6021	47.19	60.98
Ankyrin-repeat containing protein	F4JXP4	chain_9601	3.5966	51.84	64.65
Mediator of RNA polymerase II transcription subunit 37a	Q9LKR3	chain_10938	3.5951	96.64	88.25
Quinone oxidoreductase-like protein	Q9LFK5	chain_12794	3.5928	86.36	71.43
Aquaporin TIP1-2	Q41963	NODE_27789	3.5829	100	67.5
UDP-glycosyltransferase 74C1	Q9SKC1	NODE_24690	3.5792	94.7	61.86
Respiratory burst oxidase homolog protein D	Q9FIJ0	NODE_503	3.576	88.45	77.31
Mechanosensitive ion channel protein 4	Q9LPG3	NODE_36773	3.5723	85.92	66.67
UDP-glycosyltransferase 91A1	Q940V3	chain_1496	3.5718	93.75	65.09
AT4g33900	Q9FPH4	NODE_934	3.5673	64.31	72.63

continued on the next page

Contigs promoted at 34°C (*continued*)

Name	UniProt ID	Contig	$\log_2$ (fold-Change)	Target coverage (%)	Alignment identity (%)
Wall-associated receptor kinase-like 14	Q8RY67	NODE_1637	3.5673	63.32	78.52
Putative UPF0481 protein At3g02645	P0C897	NODE_8086	3.5659	68.55	66.67
Ammonium transporter 2	Q9M6N7	NODE_5308	3.5601	93.77	80.65
Phosphoglycerate mutase-like protein 1	Q9FGF0	NODE_25455	3.5592	87.5	64.95
Arabinogalactan protein 22	Q9FK16	chain_7678	3.5568	38.78	69.44
Heat shock 70 kDa protein 9, mitochondrial	Q8GUM2	NODE_4826	3.5557	86.63	76.35
18.1 kDa class I heat shock protein	P19037	NODE_466	3.5548	91.62	79.87
Transcription factor GTE7	Q7Y214	NODE_2718	3.5543	41.98	68.33
Alpha/beta-Hydrolases superfamily protein	F4HXL0	chain_50156	3.5481	93.95	75.0
F16P17.10 protein	Q9LQ11	chain_37253	3.5456	68.27	52.86
Elongation factor 1-alpha 2	Q8W4H7	chain_2577	3.5444	100.0	94.12
Flavanone 3-hydroxylase	F4J3A5	NODE_44563	3.5444	60.53	81.82
Hexokinase-1	Q42525	chain_2573	3.5418	90.63	78.67
Glyceraldehyde-3-phosphate dehydrogenase GAPA2, chloroplastic	Q9LPW0	chain_1133	3.541	100.0	90.77
1-aminocyclopropane-1-carboxylate oxidase 4	Q06588	chain_108	3.5401	95.42	76.36
1-aminocyclopropane-1-carboxylate oxidase 4	Q06588	chain_19090	3.5285	95.42	76.36
17.6 kDa class I heat shock protein 2	Q9ZW31	chain_10136	3.5205	97.56	82.12
At1g53860	Q6DBE3	NODE_44580	3.5051	60.0	82.61
At4g39830	O65670	chain_3501	3.4992	98.15	74.37
Probable alpha,alpha-trehalose-phosphate synthase [UDP-forming] 11	Q9ZV48	NODE_16483	3.4933	64.41	58.59
AT3g59470/T16L24_20	Q93Z68	chain_9071	3.4924	86.16	77.94
Protein DETOXIFICATION 10	Q8VYL8	chain_13208	3.4901	81.25	68.75
UDP-glycosyltransferase 85A2	Q9ZWJ3	chain_13939	3.4812	99.25	73.77
AT4g33900	Q9FPH4	chain_12133	3.4773	26.19	69.77
Probable receptor-like protein kinase At1g11050	O04086	chain_30083	3.4702	83.08	67.92

continued on the next page

Contigs promoted at 34°C (*continued*)

Name	UniProt ID	Contig	$\log_2$ (fold-Change)	Target coverage (%)	Alignment identity (%)
Probable glucan endo-1,3-beta-glucosidase BG3	F4J270	chain_3756	3.4659	96.48	67.27
Elongation factor 1-alpha 2	Q8W4H7	NODE_25821	3.4527	71.15	88.89
Disease resistance family protein / LRR family protein	O64757	NODE_69736	3.4473	50.7	65.71
At2g15220/F15A23.4	Q9SKL6	chain_1489	3.4462	77.16	66.97
Probable aquaporin PIP2-8	Q9ZVX8	NODE_73106	3.4338	100	77.78
Phosphorylase superfamily protein	F4JM51	chain_11937	3.43	84.0	67.31
Myb family transcription factor PHL4	Q8GXC2	NODE_7408	3.426	64.29	72.58
Mechanosensitive ion channel protein 9	Q84M97	NODE_24258	3.4248	65.47	54.44
Late embryogenesis abundant protein 3	Q9SA57	NODE_24161	3.4187	90.32	59.55
Membrane lipoprotein lipid attachment site-like protein, putative (DUF1223)	Q9FL72	chain_7657	3.4178	91.54	67.76
1-aminocyclopropane-1-carboxylate oxidase 4	Q06588	chain_4700	3.4164	44.74	79.1
Beta-D-glucan exohydrolase-like protein	Q8W112	chain_5388	3.4141	98.73	80.42
NADH dehydrogenase [ubiquinone] iron-sulfur protein 3	Q95748	NODE_17746	3.4139	77.57	97.56
Extradiol ring-cleavage dioxygenase	Q949R4	chain_3345	3.4133	67.89	65.75
AT5g02020/T7H20_70	Q9LZM9	chain_8138	3.4081	61.39	90.0
Probable xyloglucan endotransglucosylase/hydrolase protein 23	Q38910	chain_13034	3.4055	97.36	81.56
AT4g32140/F10N7_50	O49378	NODE_16509	3.4021	98.63	79.72
NDR1/HIN1-like protein 3	Q9FNH6	chain_51086	3.3906	67.53	67.42
At1g80480	Q9M8L6	chain_14440	3.3874	93.95	82.44
Lipoxygenase 2, chloroplastic	P38418	chain_4580	3.3809	98.88	72.46
Inositol-3-phosphate synthase isozyme 1	P42801	chain_13625	3.3779	85.03	88.61
Probable alkaline/neutral invertase A, chloroplastic	Q84JL5	chain_12356	3.3765	99.65	80.63
Serine carboxypeptidase-like 18	A0A1P8ASK8	chain_4288	3.3653	45.74	65.81
Eukaryotic aspartyl protease family protein	Q9ZVS4	NODE_34415	3.3524	52.58	64.0
Zinc finger protein ZAT5	Q681X4	chain_43372	3.3479	21.12	81.82

continued on the next page

Contigs promoted at 34°C (*continued*)

Name	UniProt ID	Contig	$\log_2$ (fold-Change)	Target coverage (%)	Alignment identity (%)
Probable glucan endo-1,3-beta-glucosidase BG3	F4J270	chain_50168	3.3459	96.84	68.95
UDP-glycosyltransferase 74F2	O22822	chain_61294	3.3446	84.09	69.7
1-aminocyclopropane-1-carboxylate oxidase 4	Q06588	chain_35810	3.3433	48.28	82.93
Plasma membrane intrinsic protein 2	A0A1P8AYA1	NODE_33473	3.3427	82.98	78.95
40S ribosomal protein S10-1	Q9SW09	chain_48238	3.3403	76.0	88.89
Benzoate–CoA ligase, peroxisomal	Q9SS01	NODE_1474	3.3359	90.74	72.1
Chalcone synthase	P13114	NODE_9093	3.3278	66.28	80.36
Glutathione S-transferase U19	Q9ZRW8	chain_43727	3.3276	90.38	69.57
AT5g02020/T7H20_70	Q9LZM9	chain_9451	3.3269	61.39	90.0
Transcription repressor MYB4	Q9SZP1	NODE_49750	3.3246	85.71	86.96
Probable mediator of RNA polymerase II transcription subunit 37c	Q9LHA8	chain_1256	3.3084	98.63	93.91
Squalene epoxidase 1	Q9SM02	chain_12188	3.3057	85.32	82.33
O-fucosyltransferase 28	Q9M393	NODE_25095	3.3047	75.0	62.5
Probable galactinol–sucrose galactosyltransferase 2	Q94A08	NODE_68748	3.3	58.33	90.0
Adenine nucleotide alpha hydrolases-like superfamily protein	Q9SIJ8	chain_2685	3.2995	77.72	75.35
Vacuolar-processing enzyme gamma-isozyme	Q39119	chain_41113	3.2964	98.15	85.29
Probable aldo-keto reductase 1	Q9C5B9	chain_67120	3.2887	56.0	88.89
UDP-glycosyltransferase 72B1	Q9M156	NODE_21671	3.285	96.58	68.75
Probable acyl-activating enzyme 2	Q9SEY5	NODE_17227	3.2817	96.71	62.04
At1g67330	Q9FYG0	NODE_26195	3.2774	100.0	70.0
Late embryogenis abundant protein 41	Q39084	chain_1592	3.2774	50.0	77.78
Pathogenesis-related thaumatin superfamily protein	Q9SZP4	NODE_12043	3.2759	49.78	59.26
Probable galactinol–sucrose galactosyltransferase 2	Q94A08	NODE_50963	3.2702	47.92	81.82
ABC transporter C family member 3	Q9LK64	chain_30027	3.2669	99.51	80.44
17.6 kDa class I heat shock protein 2	Q9ZW31	chain_11076	3.2666	97.8	82.89
Chaperone protein dnaJ 20, chloroplastic	Q9SDN0	chain_53704	3.2627	36.51	85.29

continued on the next page

Contigs promoted at 34°C (*continued*)

Name	UniProt ID	Contig	$\log_2$ (fold-Change)	Target coverage (%)	Alignment identity (%)
1-aminocyclopropane-1-carboxylate oxidase 2	Q41931	chain_4937	3.2523	76.98	88.54
Basic endochitinase B	P19171	chain_25388	3.2484	78.89	76.62
Photosystem I reaction center subunit VI-1, chloroplastic	Q9SUI7	chain_67054	3.2396	31.43	90.48
AP2-like ethylene-responsive transcription factor At2g41710	Q8GWK2	NODE_7503	3.228	41.18	70.73
Chlorophyll a-b binding protein CP26, chloroplastic	Q9XF89	chain_8125	3.2237	34.92	90.2
At5g43870	Q9LKC2	chain_12501	3.2195	77.24	85.71
ORMDL family protein	Q9C5I0	chain_8955	3.2148	100	85.71
Cytochrome P450 76C3	O64638	NODE_10760	3.2055	44.57	60.0
Cytochrome P450 71B22	Q9LTM1	NODE_24520	3.2009	60.0	63.64
CBL-interacting serine/threonine-protein kinase 21	Q94CG0	chain_3602	3.199	32.73	82.86
CMP-sialic acid transporter 4	F4JN00	chain_1535	3.195	91.79	90.16
Protein DETOXIFICATION 47, chloroplastic	Q945F0	NODE_9597	3.1856	84.21	65.22
1-aminocyclopropane-1-carboxylate oxidase 2	Q41931	NODE_33266	3.1784	100.0	84.06
Nudix hydrolase 15, mitochondrial	Q8GYB1	chain_10895	3.1671	80.06	79.83
Putative respiratory burst oxidase homolog protein G	Q9SW17	NODE_23942	3.1653	100	77.89
Vacuolar-processing enzyme gamma-isozyme	Q39119	chain_14173	3.1652	98.15	85.29
At3g52530	Q9SVD4	NODE_32924	3.162	60.64	53.57
Methionine aminopeptidase 2A	Q9FV49	NODE_21629	3.1588	90.54	75.76
Glucosidase-like protein	Q9SNC1	chain_16182	3.158	91.35	58.51
Defensin-like protein 1	Q9ZUL7	NODE_14206	3.1578	47.86	70.91
S-adenosylmethionine decarboxylase proenzyme 1	Q96286	chain_70314	3.1515	33.33	75.86
Uncharacterized protein	Q1G3G9	NODE_45676	3.1508	36.05	73.33
Alpha-humulene/(-)-(E)-beta-caryophyllene synthase	Q84UU4	chain_12563	3.1502	31.95	61.39
Acidic endochitinase	P19172	NODE_32959	3.1476	92.5	77.78
Similar to part of disease resistance protein	Q9SSN5	chain_22443	3.1464	63.11	54.69

continued on the next page

Contigs promoted at 34°C (*continued*)

Name	UniProt ID	Contig	$\log_2$ (fold-Change)	Target coverage (%)	Alignment identity (%)
Beta glucosidase 41	A0A1P8BA32	chain_15123	3.1456	81.74	75.27
At1g68440/T2E12_1	Q9M9C9	chain_10931	3.1425	29.08	65.0
Uncharacterized protein At1g66480	Q6NLC8	NODE_8204	3.1378	47.41	72.73
Probable indole-3-acetic acid-amido synthetase GH3.1	O82333	NODE_20061	3.1372	77.11	85.04
G-type lectin S-receptor-like serine/threonine-protein kinase At1g11330	Q9SXB8	NODE_4813	3.1345	79.61	65.26
O-fucosyltransferase 19	Q9SH89	NODE_18657	3.1341	96.15	81.82
Probable WRKY transcription factor 48	Q9FGZ4	chain_14226	3.1309	52.66	78.41
Leucoanthocyanidin dioxygenase	Q96323	chain_22609	3.1282	20.0	94.44
Probable WRKY transcription factor 75	Q9FYA2	chain_6508	3.127	53.22	77.78
Alpha-galactosidase 1	Q9FT97	chain_14302	3.1228	99.23	82.81
Ubiquinol oxidase 3, mitochondrial	Q8LEE7	NODE_3393	3.1199	95.95	76.6
Leucoanthocyanidin dioxygenase	Q96323	NODE_12953	3.1169	81.51	78.81
AWPM-19-like family protein	O23029	chain_14405	3.1153	78.13	72.97
Vesicle-associated membrane protein 721	A0A1P8AS81	chain_7204	3.1151	98.25	88.29
Probable alpha,alpha-trehalose-phosphate synthase [UDP-forming] 8	Q0WUI9	NODE_944	3.1136	96.08	69.95
Caffeoylshikimate esterase	Q9C942	chain_47771	3.1134	79.13	82.1
SDA1 family protein	Q9M082	chain_6569	3.1131	59.73	71.43
Leucine-rich repeat receptor-like protein kinase PEPR1	Q9SSL9	NODE_23005	3.1125	81.44	73.61
40S ribosomal protein S3a-1	Q9CAV0	NODE_8590	3.111	60.07	71.26
At2g31940/F20M17.2	Q8RU85	chain_6634	3.1062	75.56	68.42
At3g50390	A2RVN9	NODE_16138	3.106	43.94	82.14
Probable LRR receptor-like serine/threonine-protein kinase At4g29180	C0LGR6	chain_22260	3.1027	57.89	80.95
NADH–cytochrome b5 reductase 1	Q9ZNT1	chain_2990	3.1022	87.59	80.49
Auxin response factor 5	P93024	NODE_13907	3.0971	89.67	85.71

continued on the next page

Contigs promoted at 34°C (*continued*)

Name	UniProt ID	Contig	$\log_2$ (fold-Change)	Target coverage (%)	Alignment identity (%)
Acid phosphatase-like protein	Q9M0F4	chain_460	3.0956	10.0	66.67
p-loop containing nucleoside triphosphate hydrolases superfamily protein	Q0WPP7	chain_12769	3.0953	48.96	70.65
Phosphorylase superfamily protein	F4JM51	chain_2891	3.0915	98.91	67.05
60S ribosomal protein L32-1	P49211	NODE_17099	3.0898	91.18	64.23
Alpha-amylase 1	Q8VZ56	NODE_22502	3.0782	95.56	69.05
2-nitropropane dioxygenase-like protein	Q9FMG0	chain_6773	3.0751	92.35	82.15
High mobility group B protein 3	P93047	NODE_21729	3.0738	76.8	70.59
Actin-related protein 4	Q84M92	chain_6405	3.0728	97.55	77.06
Wall-associated receptor kinase-like 22	Q8RY17	NODE_11596	3.0696	53.42	65.79
At2g05510	Q9SL16	chain_7721	3.0665	71.56	60.43
At2g15220/F15A23.4	Q9SKL6	NODE_12050	3.0586	78.62	66.97
AT4g32480/F8B4_180	Q9SUU4	chain_9909	3.0567	63.7	76.47
Peroxidase 52	Q9FLC0	NODE_15208	3.0538	100	73.45
Probable WRKY transcription factor 75	Q9FYA2	chain_3379	3.0478	51.3	88.78
Mitogen-activated protein kinase 19	Q9LUC3	chain_3343	3.0468	93.31	91.2
PHD finger protein ALFIN-LIKE 2	Q9SRM4	chain_5118	3.0452	99.02	83.94
Naringenin,2-oxoglutarate 3-dioxygenase	Q9S818	chain_63882	3.0443	36.11	92.0
Probable prolyl 4-hydroxylase 9	Q8VZJ7	chain_13633	3.0368	81.06	79.25
tRNA (guanine(37)-N1)-methyltransferase 2	Q6NQ64	chain_9807	3.033	92.93	82.46
Uncharacterized mitochondrial protein AtMg00030	P93276	chain_1446	3.0228	39.87	98.39
Elongation factor 1-alpha 2	Q8W4H7	chain_8239	3.0209	99.46	92.9
Cyclin-T1-5	Q9FKE6	chain_13935	3.0194	86.02	88.05
GDSL esterase/lipase At5g55050	Q9FIA1	chain_11269	3.0174	71.22	66.83
Probable nucleoredoxin 1	O80763	chain_9898	3.0169	90.48	69.23
Amino-terminal region of chorein	Q84R14	chain_2446	3.0116	52.77	68.15

continued on the next page

Contigs promoted at 34°C (*continued*)

Name	UniProt ID	Contig	$\log_2$ (fold-Change)	Target coverage (%)	Alignment identity (%)
Glutamate dehydrogenase 1	Q43314	chain_5269	3.0078	97.74	79.92
At1g05870	Q9MA39	chain_7122	3.0075	73.86	83.48
Purple acid phosphatase 10	Q9SIV9	chain_11034	2.9986	94.72	76.26
Methionine aminopeptidase 1B, chloroplastic	Q9FV52	chain_9781	2.992	59.09	86.84
Acidic endochitinase	P19172	chain_21484	2.9914	37.5	86.96
Putative DEAD-box ATP-dependent RNA helicase 29	O49289	chain_14115	2.9901	92.31	84.03
HXXXD-type acyl-transferase family protein	Q9MAP9	chain_234	2.9834	70.54	75.0
Uncharacterized protein At2g33490	O22799	chain_6526	2.9807	66.42	72.73
Photosystem II reaction center W protein, chloroplastic	Q39194	chain_51916	2.9796	86.08	82.09
MJK13.9 protein	Q9LDU3	chain_11538	2.9773	94.39	71.23
Cytochrome P450 714A1	Q93Z79	chain_8798	2.9766	41.59	67.35
Lung seven transmembrane receptor family protein	Q8GYD0	chain_11492	2.9762	91.57	61.33
Probable WRKY transcription factor 45	Q9S763	NODE_26473	2.9711	90.0	75.51
LETM1-like protein	F4IBH5	chain_27197	2.9701	95.62	77.11
Ethylene-responsive transcription factor ERF042	Q52QU1	NODE_26779	2.9679	22.69	84.44
Nucleic acid-binding, OB-fold-like protein	Q9FKI2	NODE_2455	2.9635	87.5	91.2
Protease Do-like 9	Q9FL12	NODE_11336	2.9634	90.32	87.27
Calcium-binding protein KIC	Q9ZPX9	NODE_17936	2.9594	90.91	71.79
E3 ubiquitin-protein ligase UPL5	Q9SU29	chain_33573	2.9509	57.89	80.95
ATP synthase subunit 9, mitochondrial	Q304C3	chain_8141	2.9468	98.82	90.36
Cellulase (Glycosyl hydrolase family 5) protein	Q9LTM8	NODE_3102	2.9313	96.36	70.04
Protein MOTHER of FT and TFL1	Q9XFK7	chain_25320	2.9312	77.39	65.88
Peptide methionine sulfoxide reductase A1	Q9FKF7	chain_587	2.9264	79.17	73.61
Plasma membrane intrinsic protein 2	A0A1P8AYA1	chain_66314	2.9261	96.74	86.36
Cysteine synthase 1	P47998	chain_8883	2.9256	95.52	83.07
Glutamine-dependent asparagine synthase 1	F4JBH6	chain_4557	2.9243	97.25	87.85

continued on the next page

Contigs promoted at 34°C (*continued*)

Name	UniProt ID	Contig	$\log_2$ (fold-Change)	Target coverage (%)	Alignment identity (%)
Protein ETHYLENE INSENSITIVE 3	O24606	chain_1943	2.9231	72.38	86.13
LysM domain receptor-like kinase 4	O64825	NODE_4013	2.9217	53.65	66.32
60S acidic ribosomal protein P0-3	P57691	chain_11727	2.9212	76.17	65.14
Disease resistance protein (TIR-NBS-LRR class) family	F4K5U7	NODE_41844	2.9166	57.14	64.71
3-ketoacyl-CoA thiolase 5, peroxisomal	Q570C8	chain_3825	2.9159	97.8	81.69
NDR1/HIN1-like protein 10	Q9SJ52	NODE_18535	2.9124	24.8	66.67
Monothiol glutaredoxin-S2	Q8L8Z8	NODE_13757	2.9106	84.17	61.0
Cupredoxin superfamily protein	A0A1P8B4N7	chain_15292	2.9106	57.89	65.12
Exocyst complex component EXO70H1	Q8VY27	NODE_2834	2.9092	95.11	64.79
Polygalacturonase inhibitor 2	Q9M5J8	NODE_8103	2.9067	100	69.23
Probable cysteine protease RD21C	Q9LT78	chain_13235	2.9048	33.55	60.78
Protein MARD1	Q8LGS1	chain_17195	2.9045	28.21	67.86
Beta-glucosidase 12	Q9FH03	NODE_2827	2.901	84.43	65.59
Phenylalanine ammonia-lyase 4	Q9SS45	NODE_977	2.9009	93.51	87.8
Glycine-rich protein family	A8MQZ5	chain_5537	2.9001	41.73	61.18
Peptidyl serine alpha-galactosyltransferase	Q8VYF9	NODE_8428	2.895	84.67	76.26
Calcium-binding EF-hand family protein	Q9T0I9	NODE_25368	2.8945	87.27	65.96
Chalcone–flavonone isomerase 1	P41088	chain_3860	2.892	83.33	71.19
F21J9.24	Q9FYK6	NODE_11664	2.8903	82.63	79.38
AT1G01800 protein	Q94K30	chain_69885	2.8843	69.37	72.73
Peroxisomal adenine nucleotide carrier 1	Q9MA90	NODE_9592	2.8715	86.21	80.0
Alpha-amylase 1	Q8VZ56	NODE_27875	2.8713	81.15	72.53
Farnesyl pyrophosphate synthase 1, mitochondrial	Q09152	chain_38249	2.8703	89.43	75.23
Probable anion transporter 3, chloroplastic	Q7XJR2	NODE_1756	2.8672	83.78	81.08
Protein arginine methyltransferase NDUFAF7	Q94C90	chain_13648	2.8667	38.55	100.0
Probable receptor-like protein kinase At5g39020	Q9FID6	chain_11959	2.8596	91.03	72.95

continued on the next page

Contigs promoted at 34°C (*continued*)

Name	UniProt ID	Contig	$\log_2$ (fold-Change)	Target coverage (%)	Alignment identity (%)
At3g27250	Q9LK28	NODE_7816	2.8544	66.67	64.44
Mechanosensitive ion channel protein 10	Q9LYG9	chain_9005	2.8538	89.36	67.03
F2K11.24	Q9SH24	NODE_9596	2.8495	96.83	67.94
Monothiol glutaredoxin-S2	Q8L8Z8	NODE_15681	2.8492	88.79	59.8
Laccase-7	Q9SR40	NODE_20595	2.8476	75.35	50.94
Classical arabinogalactan protein 5	Q8LCE4	NODE_30889	2.8425	31.09	63.89
Putative NADP-dependent oxidoreductase	Q9M9M7	NODE_28143	2.8414	97.71	76.38
Protein DETOXIFICATION 3	Q9SIA4	NODE_12680	2.8401	80.17	69.05
Pyrophosphate–fructose 6-phosphate 1-phosphotransferase subunit alpha 1	Q9SYP2	NODE_8460	2.8376	70.0	96.36
UDP-glycosyltransferase 76B1	Q9C768	NODE_9169	2.8344	66.27	63.59
Histone H3-like 3	Q9LR02	chain_11811	2.8306	92.59	97.96
Jasmonate-zim-domain protein 1	Q3ED96	NODE_5944	2.8174	13.55	69.7
Ethylene-responsive transcription factor 1B	Q8LDC8	NODE_14964	2.8169	57.33	78.13
Probable galactinol–sucrose galactosyltransferase 2	Q94A08	chain_2304	2.8158	94.53	69.23
Thioredoxin H3	Q42403	chain_34705	2.8129	61.54	67.27
Formate dehydrogenase, chloroplastic/mitochondrial	Q9S7E4	chain_35744	2.8125	52.69	90.41
Calreticulin-3	O04153	chain_1232	2.809	33.9	74.36
Amino acid-ligase	Q9SFU5	NODE_36491	2.7968	33.33	66.67
Late embryogenesis abundant protein 46	Q9FG31	chain_1548	2.7943	74.26	68.5
17.6 kDa class I heat shock protein 1	Q9XIE3	NODE_16037	2.7917	95.24	77.06
Probable mediator of RNA polymerase II transcription subunit 37c	Q9LHA8	chain_92	2.7848	86.46	87.8
F24O1.6	Q9MAV4	chain_2099	2.7777	59.22	59.43
At2g16940	Q9ZVW9	chain_47198	2.7743	61.36	72.64
Elongation factor 1-alpha 2	Q8W4H7	NODE_46858	2.7705	100.0	91.67
Peroxisomal adenine nucleotide carrier 1	Q9MA90	NODE_21643	2.7699	87.5	77.6
Dihydroflavonol 4-reductase	P51102	NODE_53917	2.7684	76.32	82.14

continued on the next page

Contigs promoted at 34°C (*continued*)

Name	UniProt ID	Contig	$\log_2$ (fold-Change)	Target coverage (%)	Alignment identity (%)
Mitochondrial substrate carrier family protein	Q9LY28	NODE_25723	2.7675	93.14	81.94
Calreticulin-3	O04153	NODE_37835	2.7617	72.13	71.43
Vacuolar sorting protein 3	F4I312	chain_5097	2.7609	72.39	76.17
Uncharacterized protein	F4HQT8	NODE_48984	2.7601	60.61	58.97
Protein TIC 20-IV, chloroplastic	Q9ZQZ9	NODE_19074	2.7582	71.13	59.0
Probable amino acid permease 7	Q9FF99	NODE_48252	2.7551	90.48	69.33
60S ribosomal protein L34-3	Q9LJW6	chain_2604	2.7515	84.67	93.04
Leucine-rich repeat protein kinase family protein	Q9SNE4	chain_16572	2.7415	55.84	59.52
Phospho-2-dehydro-3-deoxyheptonate aldolase	Q9SK84	chain_1326	2.7405	96.1	90.0
UDP-Glycosyltransferase superfamily protein	A0A1P8AXZ2	NODE_7224	2.7372	78.09	65.56
Uncharacterized protein At5g17350	Q9LF49	chain_23743	2.7353	15.18	63.64
AT3g18280/MIE15_7	Q9LJQ3	chain_5522	2.7348	29.83	64.15
1-aminocyclopropane-1-carboxylate oxidase 4	Q06588	chain_63932	2.7343	44.74	79.1
Early nodulin-like protein 2	Q9T076	chain_4069	2.7313	64.2	57.28
RHOMBOID-like protein 8	F4I8K2	NODE_35103	2.7287	38.89	90.0
Chlorophyll a-b binding protein CP29.2, chloroplastic	Q9XF88	chain_9410	2.7223	66.39	88.75
Probable nucleoredoxin 1	O80763	chain_37521	2.7202	96.88	73.33
Trans-cinnamate 4-monooxygenase	P92994	chain_707	2.7178	94.82	86.48
UDP-glycosyltransferase 84A3	O23401	chain_780	2.7159	83.67	68.59
17.6 kDa class I heat shock protein 2	Q9ZW31	NODE_9977	2.7144	98.18	81.76
Calcium-binding EF-hand family protein	Q9T0I9	NODE_33056	2.7082	53.57	82.76
Heavy metal transport/detoxification superfamily protein	Q9C684	chain_12755	2.7068	34.56	60.87
G-type lectin S-receptor-like serine/threonine-protein kinase At1g34300	Q9XID3	NODE_4183	2.7065	86.67	62.6
Serine carboxypeptidase-like 46	Q8VY01	NODE_30708	2.7056	50.0	75.47
Aspartate/glutamate/uridylate kinase family protein	Q9SR68	NODE_1376	2.7028	97.0	72.31

continued on the next page

Contigs promoted at 34°C (*continued*)

Name	UniProt ID	Contig	$\log_2$ (fold-Change)	Target coverage (%)	Alignment identity (%)
Flavin-containing monooxygenase FMO GS-OX-like 8	Q9FLK4	chain_63135	2.7024	27.94	88.89
Aspartokinase 3, chloroplastic	Q9S702	NODE_2060	2.7012	99.37	82.22
tRNA dimethylallyltransferase 9	Q9C5J6	NODE_22924	2.6918	98.16	72.41
2-C-methyl-D-erythritol 2,4-cyclodiphosphate synthase, chloroplastic	Q9CAK8	chain_62544	2.6915	82.23	91.3
Diacylglycerol kinase 5	Q9C5E5	chain_13032	2.6884	86.75	81.53
ATP synthase subunit alpha, mitochondrial	P92549	chain_13603	2.687	20.0	79.17
E3 ubiquitin-protein ligase RZFP34	Q9FFB6	NODE_35	2.6815	79.57	67.58
NADP-dependent glyceraldehyde-3-phosphate dehydrogenase	Q1WIQ6	chain_21407	2.6785	58.7	80.77
TLD-domain containing nucleolar protein	Q9FKA3	chain_7877	2.6781	68.06	74.23
Putative DUF21 domain-containing protein At3g13070, chloroplastic	Q9LK65	chain_48666	2.676	63.41	92.0
Zinc finger A20 and AN1 domain-containing stress-associated protein 2	Q8H0X0	chain_33915	2.6749	44.23	86.36
B12D protein	Q9LJ47	NODE_26599	2.6638	62.12	61.64
Peptide upstream protein	Q9LFH7	NODE_10432	2.6589	76.76	73.45
tRNA dimethylallyltransferase 9	Q9C5J6	NODE_24373	2.656	80.61	68.12
Aluminum-activated malate transporter 5	Q93Z29	NODE_4927	2.6478	85.96	80.08
Ras-related protein RABD2c	Q9SEH3	NODE_13518	2.647	81.54	87.34
Translocation protein-like protein	Q9LIH2	chain_1226	2.6461	56.57	75.0
G-type lectin S-receptor-like serine/threonine-protein kinase At1g11300	Q9SXB4	NODE_28947	2.6354	29.03	62.86
Serine/threonine-protein kinase AtPK1/AtPK6	P42818	chain_52344	2.6318	54.04	70.73
AT5g16650/MTG13_10	Q9FMD2	chain_51233	2.6309	59.15	85.42
At3g50390	A2RVN9	NODE_17103	2.6272	43.94	82.14
Bifunctional TENA-E protein	Q9ASY9	chain_6708	2.6263	72.33	70.18
Receptor-like protein 43	Q9LJW7	chain_16383	2.6235	57.66	49.21
Basic endochitinase B	P19171	chain_2971	2.6212	87.83	72.4

continued on the next page

Contigs promoted at 34°C (*continued*)

Name	UniProt ID	Contig	$\log_2$ (fold-Change)	Target coverage (%)	Alignment identity (%)
Cysteine-rich receptor-like protein kinase 29	Q8S9L6	chain_7790	2.6143	84.65	71.59
Cytokinin dehydrogenase 5	Q67YU0	NODE_2093	2.6096	93.84	78.44
Lung seven transmembrane receptor family protein	Q8GYD0	NODE_13340	2.603	75.45	81.82
Defensin-like protein 5	Q9C947	NODE_15467	2.6009	56.0	52.73
ATPase 5, plasma membrane-type	Q9SJB3	NODE_12853	2.5985	81.48	86.15
At1g07040	Q9LMJ7	chain_1511	2.5975	54.24	67.74
Beta carbonic anhydrase 2, chloroplastic	P42737	chain_9539	2.5974	90.14	58.73
Adenylosuccinate synthetase, chloroplastic	Q96529	NODE_8840	2.5951	93.02	87.18
At2g39210/T16B24.15	O80960	chain_1516	2.5919	92.26	78.64
AT1G01800 protein	Q94K30	chain_22640	2.5875	96.39	65.58
Glycine-rich protein family	F4K4U0	NODE_47563	2.5808	63.64	70.37
30S ribosomal protein	Q94K97	NODE_5690	2.58	86.45	79.28
Probable membrane-associated kinase regulator 1	Q3E936	NODE_10834	2.5775	34.72	93.15
Inosine-uridine preferring nucleoside hydrolase family protein	F4JZJ2	NODE_14369	2.5766	93.38	62.14
Nuclease	Q84J48	NODE_1893	2.5756	65.31	70.92
Peroxidase 54	Q9FG34	NODE_10281	2.5654	76.62	58.12
Amino acid transporter AVT6A	Q9LI61	chain_6888	2.5637	86.07	71.01
Probable serine/threonine-protein kinase PBL19	Q9LTC0	chain_7737	2.5601	81.35	72.88
Adenylyl-sulfate kinase 3	Q9SRW7	chain_5315	2.5471	84.78	72.41
Glutamine-fructose-6-phosphate transaminase 2	Q9LIP9	chain_13378	2.5457	92.71	84.88
Rop guanine nucleotide exchange factor 9	Q9SVQ3	chain_37907	2.5437	90.91	71.43
Receptor-like protein kinase 7	F4I2N7	NODE_1728	2.5422	93.57	69.7
2-oxoglutarate (2OG) and Fe(II)-dependent oxygenase superfamily protein	Q9SB32	NODE_24051	2.5237	68.42	70.13
Serine carboxypeptidase-like 50	Q9M9Q6	chain_13177	2.5173	57.78	58.44
Serine–glyoxylate aminotransferase	Q56YA5	chain_16494	2.5157	98.31	86.85

continued on the next page

Contigs promoted at 34°C (*continued*)

Name	UniProt ID	Contig	$\log_2$ (fold-Change)	Target coverage (%)	Alignment identity (%)
UDP-glycosyltransferase 73B4	Q7Y232	chain_11256	2.5142	62.71	76.42
Serine carboxypeptidase-like 50	Q9M9Q6	chain_44340	2.5127	75.82	58.82
Tricyclene synthase, chloroplastic	A4FVP2	NODE_6557	2.5125	45.88	53.25
Probable aquaporin PIP1-5	Q8LAA6	chain_70106	2.5075	96.08	91.67
At4g32960	O82638	chain_28198	2.5003	76.53	70.99
Protein DETOXIFICATION 33	Q9SX83	NODE_1788	2.4962	91.53	76.38
Photosystem I reaction center subunit III, chloroplastic	Q9SHE8	chain_5532	2.4956	52.82	91.89
AGC (cAMP-dependent, cGMP-dependent and protein kinase C) kinase family protein	O64573	chain_5240	2.4925	67.16	73.53
Alpha/beta-Hydrolases superfamily protein	Q9FMQ5	NODE_6876	2.4877	88.17	65.35
Probable calcium-binding protein CML46	Q93Z27	NODE_11763	2.4846	31.46	73.49
40S ribosomal protein S3a-1	Q9CAV0	NODE_7498	2.4779	90.97	85.0
1-aminocyclopropane-1-carboxylate oxidase 2	Q41931	NODE_31076	2.4718	100.0	80.0
Protein NETWORKED 1C	Q9ZQX8	NODE_13030	2.4709	100	58.11
Non-specific phospholipase C6	Q8H965	chain_12649	2.467	89.68	69.86
60S ribosomal protein L34-3	Q9LJW6	chain_59474	2.4651	84.67	93.04
Ethylene-responsive transcription factor ERF053	Q9SKT1	NODE_8419	2.465	34.23	87.04
Cotton fiber expressed protein 1-like protein	Q9FL70	NODE_13853	2.4641	32.38	72.73
RNA polymerase II C-terminal domain phosphatase-like 3	Q8LL04	chain_5809	2.4632	98.37	83.82
Probable serine/threonine-protein kinase PBL3	O49840	NODE_5435	2.4629	61.67	56.86
Cotton fiber (DUF761)	F4HTI9	NODE_16738	2.4621	40.82	61.02
Probable disease resistance protein At5g66910	Q9FKZ0	chain_13414	2.4619	64.54	58.93
Calreticulin 3	F4HW29	chain_7649	2.4604	90.05	78.19
17.6 kDa class I heat shock protein 2	Q9ZW31	chain_21773	2.4565	90.79	82.35
Monomeric G-protein	Q9LSZ4	NODE_13566	2.4542	100	82.83
At4g39830	O65670	chain_11213	2.4394	98.15	74.37

continued on the next page

Contigs promoted at 34°C (*continued*)

Name	UniProt ID	Contig	$\log_2$ (fold-Change)	Target coverage (%)	Alignment identity (%)
BAG family molecular chaperone regulator 5, mitochondrial	O65373	NODE_16660	2.4384	20.59	64.71
3-hydroxy-3-methylglutaryl-coenzyme A reductase 1	P14891	chain_3281	2.4355	86.57	83.44
Alpha-amylase 1	Q8VZ56	chain_4151	2.4335	100.0	68.0
UDP-glycosyltransferase 72B1	Q9M156	chain_15290	2.4277	79.67	63.64
Dolichol-phosphate mannosyltransferase subunit 1	Q9LM93	NODE_7401	2.4263	88.97	93.56
Glutaredoxin-C9	Q9SGP6	chain_18717	2.413	71.93	79.22
Cysteine-rich receptor-like protein kinase 38	Q9XEC8	chain_70153	2.4115	52.0	84.0
Leucine-rich repeat protein kinase family protein	Q9SN80	chain_41857	2.4095	48.21	73.08
Protein trichome birefringence-like 10	Q9LDG2	chain_11354	2.4083	94.74	68.18
Formate dehydrogenase, chloroplastic/mitochondrial	Q9S7E4	chain_5279	2.4047	52.5	90.41
Protein phosphatase 2C 56	P49597	NODE_7229	2.4031	97.56	77.78
Glucan endo-1,3-beta-glucosidase, acidic isoform	P33157	chain_11790	2.4027	83.94	64.36
AT3g17170/K14A17_29	Q948R9	chain_3623	2.3969	80.0	74.36
1-aminocyclopropane-1-carboxylate oxidase 2	Q41931	chain_47569	2.3964	53.73	80.0
Probable WRKY transcription factor 17	Q9SJA8	NODE_7226	2.3847	87.2	80.39
Forkhead-associated domain protein	Q84K72	NODE_7089	2.3842	63.53	67.07
ATP-dependent zinc metalloprotease FTSH 1, chloroplastic	Q39102	chain_11841	2.3798	90.63	84.88
Temperature-sensitive sn-2 acyl-lipid omega-3 desaturase (ferredoxin), chloroplastic	P48622	chain_4099	2.3754	68.97	86.46
F-box protein VBF	Q9C7K0	chain_11886	2.3738	90.91	59.49
Zinc finger protein ZAT10	Q96289	chain_3666	2.3714	68.12	75.71
Cytochrome P450, family 716, subfamily A, polypeptide 1	Q9LVY7	chain_923	2.3634	59.21	79.55
Ribosomal protein L1p/L10e family	Q8RWT4	NODE_4262	2.3624	87.89	70.93
Uncharacterized protein At5g25360	Q84JY4	chain_2669	2.3618	57.03	79.17
Protein kinase superfamily protein	A0A1P8B693	NODE_13807	2.3605	100.0	77.78
ATP synthase protein MI25	Q04613	NODE_22219	2.356	93.75	90.83

continued on the next page

Contigs promoted at 34°C (*continued*)

Name	UniProt ID	Contig	$\log_2$ (fold-Change)	Target coverage (%)	Alignment identity (%)
G-type lectin S-receptor-like serine/threonine-protein kinase At1g61490	O64770	chain_40643	2.3544	55.83	58.97
Glycerol-3-phosphate dehydrogenase [NAD(+)] At3g07690, cytosolic	Q9S785	chain_35450	2.3535	38.82	81.25
UDP-glycosyltransferase 74E2	Q9SYK9	chain_12921	2.3513	83.41	68.51
At2g47485	Q6DBG0	NODE_17737	2.3458	30.77	58.97
Transcription factor MYB62	Q9C9G7	NODE_8439	2.3453	40.29	78.9
2-oxoglutarate (2OG) and Fe(II)-dependent oxygenase superfamily protein	Q9FI36	NODE_4868	2.3423	88.26	81.3
PHD finger-like domain-containing protein 5B	Q0WMV8	NODE_13712	2.3388	95.69	99.09
Cytochrome P450 71A25	Q9STK8	NODE_5764	2.3366	85.33	57.87
Protein NRT1/ PTR FAMILY 5.3	Q9FNL8	chain_14193	2.3355	92.13	71.6
Glutaredoxin-C9	Q9SGP6	NODE_14632	2.3347	77.14	76.6
Mediator of RNA polymerase II transcription subunit 37a	Q9LKR3	chain_3837	2.3335	100.0	90.99
Ubiquitin carboxyl-terminal hydrolase 9	Q93Y01	NODE_4148	2.3323	80.72	85.5
Laccase-1	Q9LMS3	NODE_11014	2.3316	83.13	54.41
Bifunctional dihydrofolate reductase-thymidylate synthase 2	Q05763	NODE_902	2.3278	94.26	81.78
Ribose-phosphate pyrophosphokinase 4	Q680A5	NODE_3663	2.3262	85.79	78.26
E3 ubiquitin-protein ligase ATL41	Q9SLC3	chain_11203	2.3255	73.66	70.0
RGG repeats nuclear RNA binding protein A	O23523	chain_5272	2.3226	60.43	76.72
Uncharacterized protein	F4IMB2	NODE_50457	2.3219	44.62	96.43
50S ribosomal protein L12-3, chloroplastic	P36212	chain_37182	2.3206	34.48	94.74
Zinc finger CCCH domain-containing protein 5	Q9SY74	NODE_11652	2.3142	57.56	70.69
1-aminocyclopropane-1-carboxylate oxidase 2	Q41931	chain_21651	2.3134	76.98	88.54
F13B4.1 protein	Q9FZ74	chain_4979	2.3126	63.04	61.61
DnaJ protein ERDJ3B	Q9LZK5	chain_2323	2.3098	91.72	81.82
Dihydroflavonol 4-reductase	P51102	chain_6473	2.3097	88.7	65.35
Inosine-5'-monophosphate dehydrogenase 2	Q9SA34	NODE_2746	2.3095	72.83	88.72

continued on the next page

Contigs promoted at 34°C (*continued*)

Name	UniProt ID	Contig	$\log_2$ (fold-Change)	Target coverage (%)	Alignment identity (%)
Protein trichome birefringence-like 8	Q9CAX1	chain_13495	2.3071	82.05	68.42
Chalcone–flavonone isomerase 1	P41088	chain_5357	2.3071	43.24	80.65
E3 ubiquitin-protein ligase	Q9LHE6	NODE_4015	2.305	97.89	74.05
Seco-amyrin synthase	Q9SYN1	NODE_54630	2.303	100	59.72
60S ribosomal protein L23	P49690	chain_24624	2.3011	96.48	80.88
Probable glucan endo-1,3-beta-glucosidase At4g16260	Q8VZJ2	NODE_16038	2.3005	74.62	62.65
AT5g02020/T7H20_70	Q9LZM9	chain_10956	2.293	30.26	86.36
Cytochrome P450, family 716, subfamily A, polypeptide 1	Q9LVY7	chain_922	2.2905	61.36	67.14
At2g39210/T16B24.15	O80960	chain_6182	2.2898	79.13	68.89
Myb domain protein 16	A0A1P8BBP8	chain_4914	2.2871	14.29	75.0
Dihydroflavonol 4-reductase	P51102	chain_4681	2.2857	88.7	65.35
Peroxiredoxin-2B	Q9XEX2	chain_48189	2.2837	53.85	90.0
Homeobox-leucine zipper protein ATHB-7	P46897	chain_10836	2.2772	35.66	82.42
Probable pectinesterase/pectinesterase inhibitor 46	Q9FF78	chain_8105	2.2759	76.88	68.7
Probable WRKY transcription factor 53	Q9SUP6	NODE_823	2.2742	25.97	72.04
Alpha-galactosidase 3	Q8VXZ7	chain_11632	2.2723	84.75	78.82
Cytochrome P450 98A3	O22203	chain_14162	2.272	90.91	61.22
WRKY transcription factor 18	Q9C5T4	chain_5794	2.2717	50.17	76.39
Probable aldo-keto reductase 5	Q9ASZ9	chain_14180	2.27	71.69	84.62
Uncharacterized protein	F4I759	chain_5006	2.2693	18.41	63.89
RNA polymerase II C-terminal domain phosphatase-like 3	Q8LL04	chain_18030	2.2687	98.37	83.82
40S ribosomal protein S28-1	Q9SR73	chain_2278	2.2627	93.85	82.46
KRR1 family protein	F4J5D3	chain_9857	2.2611	65.38	76.32
Amine oxidase	Q8L866	chain_12740	2.259	79.1	94.23
1-aminocyclopropane-1-carboxylate oxidase 3	O65378	NODE_42732	2.2584	43.18	100.0
ATP-dependent Clp protease proteolytic subunit-related protein 1, chloroplastic	Q9XJ35	chain_6814	2.2548	90.7	84.29

continued on the next page

Contigs promoted at 34°C (*continued*)

Name	UniProt ID	Contig	$\log_2$ (fold-Change)	Target coverage (%)	Alignment identity (%)
Cytochrome P450 82G1	Q9LSF8	chain_8945	2.2529	52.31	66.67
Receptor-like serine/threonine-protein kinase SD1-6	Q9S972	chain_8723	2.2503	84.51	77.31
Probable pectinesterase/pectinesterase inhibitor 51	Q9LXD9	chain_9892	2.2483	92.03	72.22
4-coumarate–CoA ligase 3	Q9S777	NODE_1665	2.2443	94.11	79.01
Heavy metal-associated isoprenylated plant protein 39	O03982	chain_3164	2.2426	42.96	82.14
MACPF domain-containing protein At1g14780	Q8L612	NODE_8803	2.2394	50.9	58.56
Putative pentatricopeptide repeat-containing protein At5g36300	Q9FFZ2	NODE_39458	2.2389	56.25	79.25
DNA-directed RNA polymerases II and IV subunit 5A	O81098	chain_10865	2.2386	37.37	63.89
Pinoresinol reductase 2	Q9SVP6	NODE_8514	2.2374	81.95	68.26
Protein DETOXIFICATION 47, chloroplastic	Q945F0	NODE_17683	2.236	56.67	73.73
Protein disulfide-isomerase like 2-2	Q9MAU6	NODE_2855	2.2312	94.1	78.33
Glutathione S-transferase U21	F4IA73	NODE_14809	2.2311	96.77	67.55
Tetratricopeptide repeat (TPR)-like superfamily protein	F4KCR0	chain_7363	2.2293	100.0	68.93
ADP-ribosylation factor A1E	Q9M1P5	chain_914	2.2291	91.73	96.69
Delta-1-pyrroline-5-carboxylate synthase B	P54888	NODE_220	2.2286	98.35	82.11
Elongation factor 1-alpha 2	Q8W4H7	chain_1563	2.228	100.0	94.12
Cytochrome B5 isoform C	Q9ZNV4	chain_21579	2.2275	42.95	53.97
Mannose-6-phosphate isomerase 1	Q9M884	chain_33215	2.2214	70.38	74.2
4-coumarate–CoA ligase 3	Q9S777	chain_49255	2.2139	96.12	79.01
Aspartate aminotransferase, mitochondrial	P46643	chain_5616	2.2135	89.86	88.14
Ras-related protein RABC1	O23657	chain_46992	2.2114	70.37	88.89
Protein RETICULATA, chloroplastic	B9DFK5	chain_3892	2.2074	70.09	77.16
Putative cysteine-rich receptor-like protein kinase 39	Q9SYS7	NODE_10627	2.201	79.76	63.64
P-loop NTPase domain-containing protein LPA1 homolog 1	Q9FJH9	NODE_18491	2.1989	54.89	69.79
(+)-neomenthol dehydrogenase	Q9M2E2	chain_775	2.1961	65.67	58.14
At1g36070	Q0V7U5	chain_11997	2.1944	75.7	74.59

continued on the next page

Contigs promoted at 34°C (*continued*)

Name	UniProt ID	Contig	$\log_2$ (fold-Change)	Target coverage (%)	Alignment identity (%)
Endoplasmin homolog	Q9STX5	chain_1480	2.192	84.54	83.33
UDP-glycosyltransferase 76C3	Q9FI96	NODE_12883	2.1908	14.54	65.63
Uncharacterized protein At1g66480	Q6NLC8	NODE_11024	2.1902	54.37	64.08
1-aminocyclopropane-1-carboxylate oxidase 2	Q41931	chain_33848	2.19	89.9	78.21
Nuclear pore complex protein GP210	F4KHD8	chain_14029	2.1894	81.39	58.89
RING/FYVE/PHD zinc finger superfamily protein	Q9C6P8	chain_11542	2.1849	49.07	68.42
Kinase superfamily with octicosapeptide/Phox/Bem1p domain-containing protein	Q9LRY7	NODE_35344	2.1774	94.23	83.33
DnaJ protein ERDJ3A	Q9SR96	NODE_4252	2.1752	94.67	69.16
Alpha/beta-Hydrolases superfamily protein	Q0V802	chain_1095	2.1726	60.54	65.22
ATP synthase subunit a-1	P93298	NODE_40566	2.1722	100	94.87
Zinc finger A20 and AN1 domain-containing stress-associated protein 2	Q8H0X0	chain_1071	2.1674	60.75	71.88
Feruloyl CoA ortho-hydroxylase 2	Q9C899	chain_8569	2.1667	82.5	64.29
28 kDa heat/acid-stable phosphoprotein-like protein	Q9FNM0	chain_4494	2.1666	97.17	70.53
At3g15040	Q9LKA1	NODE_8650	2.1645	21.58	87.8
Mitochondrial uncoupling protein 5	Q9SJY5	NODE_23589	2.1626	60.94	89.47
At1g36070	Q0V7U5	chain_2637	2.1577	75.7	74.59
Protein DMR6-LIKE OXYGENASE 2	Q9ZSA7	NODE_6007	2.1553	84.19	77.3
1-aminocyclopropane-1-carboxylate synthase 6	Q9SAR0	chain_11803	2.1553	92.94	70.79
BAG family molecular chaperone regulator 7	Q9LVA0	NODE_3275	2.1541	40.14	71.01
UDP-glycosyltransferase 92A1	Q9LXV0	chain_5765	2.1516	32.6	53.45
At5g67620	Q9FJW1	NODE_17196	2.1515	83.46	78.85
Probable nucleoredoxin 1	O80763	NODE_18091	2.1511	84.25	70.75
Thiamine thiazole synthase, chloroplastic	Q38814	chain_9565	2.1505	98.78	81.25
COBRA-like protein 8	Q9LIB6	chain_13692	2.1497	63.41	68.82
L-type lectin-domain containing receptor kinase VIII.2	Q9LYX1	NODE_9053	2.1495	74.23	60.66

continued on the next page

Contigs promoted at 34°C (*continued*)

Name	UniProt ID	Contig	$\log_2$ (fold-Change)	Target coverage (%)	Alignment identity (%)
Protein EARLY FLOWERING 4	O04211	NODE_11880	2.1461	36.78	77.78
DNA repair RAD52-like protein 1, mitochondrial	Q9FVV7	chain_7919	2.1438	51.6	82.81
Alpha/beta-Hydrolases superfamily protein	Q9FVW6	chain_6884	2.1422	87.53	69.68
Probable E3 ubiquitin-protein ligase EDA40	F4JSV3	NODE_14263	2.1422	99.08	77.02
Pentatricopeptide repeat-containing protein At1g71490	Q9C9I6	NODE_15581	2.1408	90.3	66.89
Hydroxyproline O-arabinosyltransferase 3	Q9FY51	NODE_16186	2.1408	91.06	84.68
1-aminocyclopropane-1-carboxylate oxidase 2	Q41931	chain_5744	2.1397	76.98	88.54
Multiple organellar RNA editing factor 8, chloroplastic/mitochondrial	Q9LKA5	chain_4351	2.1394	69.35	87.59
Protein MOTHER of FT and TFL1	Q9XFK7	chain_14647	2.1379	77.39	65.88
Chaperone protein dnaJ 1, mitochondrial	Q38813	NODE_30141	2.1348	82.43	60.0
AT1G15380 protein	Q9XI31	chain_42691	2.1342	94.58	75.64
60S ribosomal protein L36a	O23290	chain_1194	2.1302	76.81	93.33
Heparanase-like protein 2	Q8L608	chain_2401	2.1285	93.78	69.83
CCAAT-binding factor	F4IDC2	chain_68637	2.1272	65.71	86.36
Glutamate synthase 1 [NADH], chloroplastic	Q9LV03	chain_4190	2.1253	99.58	87.63
COP9 signalosome complex subunit 3	Q8W575	chain_6051	2.121	97.01	70.28
Polyubiquitin 8	Q39256	chain_20441	2.1201	52.35	78.21
Tropinone reductase homolog At2g29370	Q9ZW20	chain_3203	2.1157	93.75	75.38
Transcription initiation factor IIB-2	Q9SS44	chain_6398	2.1083	93.75	97.73
Disease resistance protein	Q9FKB9	chain_14633	2.1073	86.13	60.91
Peroxisomal nicotinamide adenine dinucleotide carrier	O04200	NODE_7212	2.1057	77.21	84.05
40S ribosomal protein S11-2	O65569	chain_71053	2.1021	91.95	90.57
Potassium transporter 8	Q9M7J9	chain_7880	2.0902	90.0	80.85
Probable galactinol–sucrose galactosyltransferase 2	Q94A08	chain_3660	2.0862	87.5	80.46
1-aminocyclopropane-1-carboxylate oxidase 2	Q41931	chain_3917	2.0828	76.98	88.54
Heat shock 70 kDa protein 6, chloroplastic	Q9STW6	NODE_21929	2.0822	18.81	94.44

continued on the next page

Contigs promoted at 34°C (*continued*)

Name	UniProt ID	Contig	$\log_2$ (fold-Change)	Target coverage (%)	Alignment identity (%)
Calmodulin-binding protein 60 D	Q0WVV6	NODE_2958	2.0724	100.0	77.67
EIN3-binding F-box protein 1	Q9SKK0	chain_9277	2.0721	75.62	66.09
60S ribosomal protein L2, mitochondrial	P93311	NODE_14610	2.0707	91.82	87.86
Aquaporin TIP2-3	Q9FGL2	chain_16629	2.0689	93.37	81.98
UDP-glycosyltransferase 74E2	Q9SYK9	chain_3529	2.067	86.24	58.44
Alpha/beta-Hydrolases superfamily protein	F4HXL0	NODE_15784	2.0661	44.16	69.7
PAS domain-containing protein tyrosine kinase family protein	F4HVH9	chain_4312	2.0659	90.3	70.75
Afadin	F4JAG7	chain_11399	2.0655	23.18	75.76
Proteasome subunit beta type-1	P42742	chain_4287	2.0638	83.43	86.9
At5g10830	Q9LEV6	NODE_11185	2.0634	92.11	67.83
Spermine synthase	Q94BN2	chain_42638	2.0616	85.58	73.26
At4g39235	Q29Q00	chain_18515	2.0593	50.0	71.43
Glutathione hydrolase 1	Q8VYW6	NODE_1769	2.0568	89.47	67.74
Glutathione S-transferase U7	Q9ZW24	chain_11947	2.0553	100.0	66.02
Dihydrolipoamide acetyltransferase component of pyruvate dehydrogenase complex	F4J5T2	chain_8046	2.0523	44.58	68.12
Cyclic pyranopterin monophosphate synthase, mitochondrial	Q39056	NODE_22083	2.0482	96.53	84.06
Xylose isomerase	Q9FKK7	chain_2729	2.0382	82.05	86.71
Transmembrane protein, putative (DUF594)	Q9FHI3	NODE_21100	2.0381	82.11	75.0
Duplicated homeodomain-like superfamily protein	A0A1P8B126	NODE_11197	2.0377	45.56	64.29
Clavaminate synthase-like protein At3g21360	Q9LIG0	chain_10230	2.0301	89.55	74.3
Beta-D-glucan exohydrolase-like protein	Q8W112	chain_9288	2.0293	94.24	78.07
3-hydroxy-3-methylglutaryl-coenzyme A reductase 1	P14891	chain_35172	2.028	86.57	83.44
Alpha/beta hydrolase related protein	Q8L7D8	NODE_7078	2.0264	83.33	69.82
30S ribosomal protein S16-2, chloroplastic/mitochondrial	Q9LTS6	chain_4697	2.0242	84.09	87.27
Phenylalanine ammonia-lyase 2	P45724	chain_6921	2.0229	92.88	82.17

continued on the next page

Contigs promoted at 34°C (*continued*)

Name	UniProt ID	Contig	$\log_2$ (fold-Change)	Target coverage (%)	Alignment identity (%)
Alpha-L-fucosidase 3	Q9FXE5	chain_889	2.0211	78.19	66.14
Cysteine proteinase RD21A	P43297	NODE_2971	2.0132	91.56	74.33
F7A19.14 protein	Q9XI82	NODE_16956	2.0055	33.17	71.64
Cytochrome c oxidase subunit 1	P60620	NODE_31098	2.0049	89.9	96.59
U-box domain-containing protein 27	Q9FLF4	NODE_7867	2.0006	88.26	66.46
Probable mediator of RNA polymerase II transcription subunit 37c	Q9LHA8	chain_1407	2.0003	84.44	92.0
At2g43320/T1O24.6	O22847	NODE_5993	1.9978	88.72	78.49
Transmembrane protein, putative (DUF247)	Q9SN06	NODE_8731	1.9959	30.2	52.81
Guanosine deaminase	Q94BU8	chain_1799	1.9959	85.17	85.88
Hexosyltransferase (Fragment)	W8QP14	chain_1517	1.9954	83.72	79.71
Glucose-6-phosphate/phosphate translocator 2, chloroplastic	Q94B38	chain_6336	1.9952	71.94	89.27
FRIGIDA-like protein 4b	Q940H8	chain_11383	1.9927	38.15	93.85
Fatty-acid-binding protein 2	Q84RK2	NODE_8127	1.9884	87.5	76.47
Somatic embryogenesis receptor kinase 2	Q9XIC7	chain_58105	1.986	100.0	75.47
Glutathione S-transferase F12	Q9FE46	NODE_12419	1.9859	60.47	66.67
Probable inactive heme oxygenase 2, chloroplastic	O48722	chain_5290	1.9852	73.43	73.08
Calcium-dependent protein kinase 17	Q9FMP5	NODE_23668	1.9821	80.95	63.64
Pathogenesis-related thaumatin superfamily protein	Q8VYN5	NODE_4441	1.9815	76.39	68.04
Probable serine/threonine-protein kinase PBL4	Q5PP29	chain_12003	1.9794	90.67	67.16
Leucine-rich repeat protein kinase family protein	F4KGL1	chain_34791	1.9783	76.33	75.63
Pyridoxal 5'-phosphate synthase-like subunit PDX1.2	Q9ZNR6	chain_4204	1.9754	89.07	73.36
G-type lectin S-receptor-like serine/threonine-protein kinase SD2-2	Q39203	chain_16823	1.9737	55.71	61.34
At5g55900	Q9FG75	chain_8483	1.9736	92.98	72.78
Hydroxyproline O-arabinosyltransferase 3	Q9FY51	NODE_12708	1.9702	67.9	76.5
Phytosulfokine receptor 1	Q9ZVR7	chain_53617	1.9696	79.45	56.14
U3 small nucleolar RNA-associated protein 18 homolog	Q9FMU5	NODE_17110	1.9668	20.77	73.08

continued on the next page

Contigs promoted at 34°C (*continued*)

Name	UniProt ID	Contig	$\log_2$ (fold-Change)	Target coverage (%)	Alignment identity (%)
AT5g04480/T32M21_80	Q940Y7	chain_10796	1.9665	95.92	78.07
Amyrin synthase LUP2	Q8RWT0	chain_9167	1.965	92.2	63.27
Metallothionein-like protein 2B	Q38805	chain_31625	1.961	53.45	83.33
Chaperonin CPN60, mitochondrial	P29197	NODE_899	1.9602	100	89.32
Chaperone protein ClpB3, chloroplastic	Q9LF37	chain_13450	1.9587	99.03	86.14
Protein ATAF2	Q9C598	chain_46319	1.9543	56.78	83.93
4-hydroxyphenylpyruvate dioxygenase	P93836	chain_1093	1.9533	89.38	81.3
Alanine aminotransferase 1, mitochondrial	F4I7I0	NODE_1852	1.9512	100	83.62
Senescence-associated carboxylesterase 101	Q4F883	chain_13555	1.9498	16.87	87.5
Cytochrome P450 714A2	Q6NKZ8	chain_7924	1.9488	63.96	59.36
At5g20700	Q8GYX2	chain_65851	1.9465	55.7	67.44
ATP synthase subunit c, chloroplastic	P56760	chain_6071	1.9449	79.61	98.77
60S ribosomal protein L2, mitochondrial	P93311	NODE_18893	1.9448	94.48	83.58
Ethylene-responsive transcription factor 5	O80341	NODE_18719	1.9393	57.89	83.51
Protein NETWORKED 1D	F4HZB5	NODE_64	1.9375	51.59	57.17
Cytochrome b561 and DOMON domain-containing protein At5g35735	Q9FKH6	chain_2148	1.9354	80.9	66.67
Cytochrome P450 84A1	Q42600	chain_1588	1.9313	86.33	76.34
Eukaryotic translation initiation factor 4A1	A0A1I9LSZ7	chain_3637	1.9312	67.83	91.67
Putative leucine-rich repeat receptor-like serine/threonine-protein kinase At2g19230	O64556	chain_4814	1.9301	49.43	57.14
Calcium uniporter protein 3, mitochondrial	Q9LVR5	chain_3102	1.9297	61.93	70.37
At5g18540	Q6GKY0	chain_56052	1.9294	57.95	77.08
AT5g11580/F15N18_170	Q93Z10	NODE_2021	1.9283	84.05	81.0
DNA ligase	Q9SUE9	chain_10575	1.9274	43.54	69.78
Probable alkaline/neutral invertase A, chloroplastic	Q84JL5	chain_9124	1.9237	99.65	80.63
Glutathione S-transferase L2, chloroplastic	Q9M2W2	chain_50618	1.9208	87.18	65.57

continued on the next page

Contigs promoted at 34°C (*continued*)

Name	UniProt ID	Contig	$\log_2$ (fold-Change)	Target coverage (%)	Alignment identity (%)
Protein ELF4-LIKE 1	O80877	NODE_18347	1.9196	47.95	71.01
Kinesin-like protein KIN-UB	Q9LPC6	NODE_11933	1.9169	97.22	84.06
N-alpha-acetyltransferase MAK3	O80438	NODE_12263	1.9134	75.12	87.42
Serine/threonine-protein kinase SRK2D	Q39192	NODE_4829	1.9127	76.2	82.56
Membrane steroid-binding protein 1	Q9XFM6	chain_22531	1.9126	87.5	85.0
Chaperone protein ClpB1	P42730	NODE_12719	1.911	100	94.08
Chalcone synthase	P13114	chain_4709	1.9085	92.45	86.11
AT3g51000/F24M12_40	Q9SD45	NODE_5247	1.9019	95.38	72.19
Myosin-17	F4K5J1	chain_5875	1.8993	90.23	71.96
Calmodulin-5	Q682T9	chain_14545	1.8962	81.94	98.28
ATP-dependent zinc metalloprotease FTSH 1, chloroplastic	Q39102	chain_14862	1.8955	90.63	84.88
Thioredoxin reductase 2	Q39242	NODE_5256	1.8946	74.38	83.04
Golgin candidate 6	B0F9L4	chain_39642	1.8943	36.73	94.12
Histone H3-like 3	Q9LR02	chain_33651	1.8929	36.99	98.41
At3g19660	Q9LJN0	chain_4528	1.8916	73.91	66.0
At4g06536	Q1PEB2	NODE_11334	1.8894	31.91	66.29
Heavy metal-associated isoprenylated plant protein 39	O03982	NODE_28050	1.8844	86.96	72.37
Transcription regulator	F4HVV7	chain_5274	1.8839	97.52	80.17
Monooxygenase 3	Q9FLC2	chain_10155	1.8834	56.19	58.62
At1g58420	Q9C647	NODE_7648	1.8811	11.85	69.7
Protochlorophyllide reductase A, chloroplastic	Q42536	chain_962	1.8795	100.0	92.35
Oxalate–CoA ligase	Q9SMT7	NODE_12080	1.8697	94.31	82.45
Citrate synthase 3, peroxisomal	Q9SJH7	chain_61557	1.8674	90.34	90.26
Linoleate 9S-lipoxygenase 5	Q9LUW0	chain_64197	1.8645	91.59	75.18
40S ribosomal protein S3-2	Q9M339	chain_5591	1.8602	84.98	81.46
At1g13730	Q9LMX6	chain_12565	1.8557	35.71	61.22

continued on the next page

Contigs promoted at 34°C (*continued*)

Name	UniProt ID	Contig	$\log_2$ (fold-Change)	Target coverage (%)	Alignment identity (%)
Disease resistance protein (CC-NBS-LRR class) family	A0A1P8BE24	chain_13421	1.8524	58.19	61.97
Alpha/beta-Hydrolases superfamily protein	Q84JV3	NODE_6105	1.8519	92.0	81.98
Anthocyanidin reductase	Q9SEV0	chain_1281	1.8477	88.05	67.65
F-box protein SKIP8	Q93YV9	chain_12002	1.8398	40.25	95.24
COBRA-like protein 8	Q9LIB6	NODE_12022	1.8387	87.78	70.06
Nitrilase 3	P46010	NODE_5571	1.8382	89.47	71.62
Probable mediator of RNA polymerase II transcription subunit 37c	P22954	chain_734	1.8377	64.56	76.29
40S ribosomal protein S12-2	Q9SKZ3	chain_10945	1.8312	89.39	81.03
U-box domain-containing protein 29	Q9LSA6	chain_9165	1.8247	14.8	72.6
Calreticulin-3	O04153	chain_1914	1.8197	90.69	83.6
tRNA (guanine(37)-N1)-methyltransferase 2	Q6NQ64	chain_11711	1.8179	94.38	81.63
NADP-dependent malic enzyme 2	Q9LYG3	chain_5125	1.817	90.23	83.33
Auxilin-like protein 1	Q9FWS1	NODE_990	1.8162	37.96	70.0
Transcription factor jumonji (JmjC) domain-containing protein	Q8VYB9	NODE_2926	1.8159	70.67	73.42
Probable aquaporin PIP1-5	Q8LAA6	chain_5907	1.8157	97.94	86.48
40S ribosomal protein S25-4	Q9T029	chain_3088	1.8107	68.55	87.96
Glutaredoxin-C2	Q9FNE2	chain_51898	1.8099	78.05	82.54
Subtilisin-like protease SBT1.7	O65351	chain_29415	1.807	68.6	65.6
Transcription elongation factor 1 homolog	Q8LEF3	chain_46899	1.8032	79.57	83.56
Thioredoxin H3	Q42403	chain_53735	1.7998	61.54	67.27
Nematode resistance protein-like HSPRO2	O04203	NODE_3930	1.7976	96.02	66.76
Mediator of RNA polymerase II transcription subunit 37a	Q9LKR3	chain_4401	1.7956	98.87	91.09
Mitochondrial import inner membrane translocase subunit TIM23-3	Q9S837	NODE_9940	1.7944	92.43	61.74
Polypyrimidine tract-binding protein homolog 2	Q9FGL9	chain_11071	1.7897	89.14	82.12
F-box protein PP2-B11	Q949S5	chain_10062	1.7863	61.9	61.49
Pectinesterase inhibitor 10	Q9SI74	chain_7761	1.786	97.1	59.09

continued on the next page

Contigs promoted at 34°C (*continued*)

Name	UniProt ID	Contig	$\log_2$ (fold-Change)	Target coverage (%)	Alignment identity (%)
Transcription factor MYB94	Q9SN78	chain_6915	1.7857	86.86	69.81
Ribulose bisphosphate carboxylase small chain 3B, chloroplastic	P10798	chain_1125	1.7823	64.2	85.42
DEAD-box ATP-dependent RNA helicase 3, chloroplastic	Q8L7S8	chain_4688	1.7716	70.9	83.05
IAA-amino acid hydrolase ILR1	P54968	NODE_3921	1.7639	86.96	58.82
Maturase K	P56784	NODE_40950	1.7634	100	77.91
At5g19440	Q29Q34	chain_5451	1.7619	85.16	79.87
La-related protein 6B	O80567	chain_1509	1.7618	58.75	78.11
Protein VASCULAR ASSOCIATED DEATH 1, chloroplastic	F4HVW5	NODE_1010	1.7614	66.15	74.77
Beta carbonic anhydrase 1, chloroplastic	P27140	chain_845	1.7601	37.68	80.0
Sphingoid long-chain bases kinase 2, mitochondrial	O82359	chain_11808	1.7601	87.0	80.31
Anthocyanin-related membrane protein 1	Q948Q9	chain_1746	1.7573	84.05	76.8
Probable carboxylesterase 2	Q9SX78	chain_9819	1.7549	78.92	64.66
At4g11630	Q9T0D0	chain_11077	1.7541	46.38	86.61
Metalloendoproteinase 3-MMP	Q5XF51	NODE_3771	1.7466	85.94	72.02
Protein PLASTID MOVEMENT IMPAIRED 1-RELATED 1	F4K5K6	NODE_256	1.7408	78.16	75.0
Glycerophosphodiester phosphodiesterase GDPD6	Q9SD81	chain_5188	1.7388	83.21	79.14
DNA-binding bromodomain-containing protein	F4I9P9	NODE_7729	1.7366	30.4	67.26
Glutamyl-tRNA reductase 2, chloroplastic	P49294	chain_15337	1.7351	90.14	73.37
WUSCHEL-related homeobox 13	O81788	NODE_2574	1.7335	64.4	78.61
Late embryogenis abundant protein 2	Q9SRX6	NODE_18010	1.7317	31.86	71.43
Protein NRT1/ PTR FAMILY 4.5	Q8VYE4	chain_7247	1.7313	81.89	76.57
Glucose-1-phosphate adenylyltransferase large subunit 2, chloroplastic	P55230	chain_44404	1.7238	96.77	68.83
CBS domain-containing protein CBSX1, chloroplastic	O23193	NODE_3837	1.7221	58.6	81.93
AT5g58110/k21l19_90	Q9FGT3	NODE_7710	1.7204	89.52	69.71
Cryptic loci regulator	Q8LF87	NODE_21282	1.7176	24.0	68.29
Mediator of RNA polymerase II transcription subunit 37a	Q9LKR3	chain_1597	1.7157	94.19	91.82

continued on the next page

Contigs promoted at 34°C (*continued*)

Name	UniProt ID	Contig	$\log_2$ (fold-Change)	Target coverage (%)	Alignment identity (%)
NADP-dependent malic enzyme 3	Q9XGZ0	NODE_1275	1.7114	84.75	82.99
Probable serine/threonine-protein kinase PBL3	O49840	chain_14553	1.7095	80.8	62.79
Transcription factor jumonji (JmjC) domain-containing protein (Fragment)	C0SV12	chain_8118	1.7089	94.16	75.66
Serine/arginine-rich splicing factor SR45	Q9SEE9	NODE_8451	1.7026	39.32	57.78
D-lactate dehydrogenase (DUF668)	Q9C5B1	chain_11437	1.7	63.05	71.43
Hydroxymethylglutaryl-CoA synthase	P54873	chain_858	1.6924	97.25	83.62
Enolase 1, chloroplastic	Q9C9C4	NODE_2634	1.6905	99.05	93.27
Aha1 domain-containing protein	Q9LHL7	NODE_4107	1.6902	100.0	77.65
Ras-related protein RABH1d	Q9SID8	NODE_8448	1.6876	98.52	84.85
Endoglucanase 21	Q9STW8	NODE_1017	1.6851	92.71	67.52
Cyclic nucleotide-gated ion channel 1	O65717	NODE_3112	1.6843	98.39	91.21
Proline-rich extensin-like family protein	Q84JV0	chain_6292	1.6825	41.67	51.85
Tryptophan synthase beta chain	Q9FFW8	NODE_4771	1.6735	98.2	84.47
UDP-glycosyltransferase 74E2	Q9SYK9	chain_4237	1.6668	86.82	68.51
Ribosomal protein L9/RNase H1	Q9LVU5	NODE_17293	1.6656	92.27	68.42
At4g28830	Q84TF1	chain_9099	1.6639	85.48	79.02
Non-intrinsic ABC protein 9	F4KCB8	chain_5488	1.6608	92.63	87.71
Fructose-bisphosphate aldolase 2, chloroplastic	Q944G9	chain_2122	1.6598	69.62	66.67
Peroxidase 12	Q96520	chain_2715	1.6593	92.58	69.74
AT4g02340 protein	O81299	NODE_6051	1.6548	23.4	72.0
1,4-dihydroxy-2-naphthoyl-CoA thioesterase 1	Q9SX65	chain_10612	1.6505	88.48	71.74
Probable beta-1,3-galactosyltransferase 5	Q9LM60	NODE_8987	1.6497	95.11	75.0
Mechanosensitive ion channel protein 2, chloroplastic	Q56X46	NODE_6822	1.6497	10.29	81.48
Leucine-rich repeat family protein	Q8H1Q4	chain_11562	1.6491	82.89	73.94
ADP-ribosylation factor A1E	Q9M1P5	chain_8875	1.6448	82.27	97.39
ATP-citrate synthase alpha chain protein 1	Q9SGY2	chain_557	1.6378	93.6	85.82

continued on the next page

Contigs promoted at 34°C (*continued*)

Name	UniProt ID	Contig	log_2 (fold-Change)	Target coverage (%)	Alignment identity (%)
14-3-3-like protein GF14 iota	Q9C5W6	chain_16206	1.6377	90.2	75.84
Leucine-rich repeat transmembrane protein kinase	F4HRH4	chain_7413	1.6366	69.81	68.94
Golgi apparatus membrane protein-like protein ECHIDNA	Q8LEK2	chain_13819	1.6347	92.55	83.24
60S acidic ribosomal protein P1-3	Q8LEQ0	chain_5826	1.6346	55.67	71.43
Homeobox-DDT domain protein RLT3	F4JRF5	chain_11116	1.6333	82.38	74.86
ATP synthase subunit a, chloroplastic	P56758	chain_4460	1.6318	93.21	93.09
Spermine synthase	Q94BN2	NODE_32474	1.6316	98.25	80.91
bZIP transcription factor 28	Q9SG86	chain_1001	1.6256	57.94	90.74
DBH-like monooxygenase	O65233	chain_7101	1.6238	26.62	83.33
At5g20700	Q8GYX2	chain_6130	1.6197	13.24	84.62
UDP-glycosyltransferase 91C1	Q9LTA3	NODE_4211	1.6123	78.47	53.57
Peroxidase 12	Q96520	chain_704	1.6107	87.27	68.42
Chalcone–flavonone isomerase 1	P41088	chain_503	1.607	43.24	80.65
Probable alpha-mannosidase At5g13980	Q8LPJ3	chain_10916	1.6048	98.34	67.83
RING-H2 finger protein ATL2	Q8L9T5	chain_25711	1.6015	48.34	75.0
Golgi SNAP receptor complex member 1-1	Q9LMP7	NODE_8306	1.5994	93.28	85.0
ZCF37	F4I499	NODE_19372	1.5991	30.0	60.53
NAD(P)H dehydrogenase B2	F4JGL5	NODE_924	1.5921	99.46	80.45
Mitochondrial uncoupling protein 4	Q9SB52	NODE_982	1.5919	86.85	80.74
Senescence/dehydration-associated protein-like protein	Q9LVE6	chain_7241	1.5886	94.37	67.21
ATP synthase F1 complex assembly factor	O22958	chain_9941	1.5864	70.77	75.5
Sphingosine kinase 1	Q8L7L1	NODE_3058	1.5852	98.23	75.45
Ribosome biogenesis protein BRX1 homolog 1	Q9LE16	NODE_2967	1.5831	55.75	83.15
Trans-cinnamate 4-monooxygenase	P92994	chain_7012	1.5797	79.72	87.18
Probable aquaporin PIP1-5	Q8LAA6	chain_56915	1.5772	83.1	91.38
Heat shock protein 90-6, mitochondrial	F4JFN3	NODE_439	1.5755	85.68	82.83

continued on the next page

Contigs promoted at 34°C (*continued*)

Name	UniProt ID	Contig	$\log_2$ (fold-Change)	Target coverage (%)	Alignment identity (%)
HAD superfamily, subfamily IIIB acid phosphatase	Q9ZVI2	chain_7305	1.5745	82.95	70.64
Cullin-3A	Q9ZVH4	chain_8292	1.5624	99.32	79.81
Hexokinase-1	Q42525	chain_14843	1.5608	90.63	78.67
Multiprotein-bridging factor 1c	Q9LV58	chain_42125	1.5537	37.42	77.19
DUF1677 family protein (DUF1677)	Q9SK23	NODE_11772	1.5523	55.95	61.73
Probable glycosyltransferase At5g03795	Q9FFN2	NODE_25427	1.5481	72.38	65.33
Myeloid leukemia factor	F4IG50	chain_6231	1.5481	40.38	80.65
RHOMBOID-like protein 4	F4JBM4	chain_7053	1.5364	78.55	68.86
Tropinone reductase homolog At2g29260, chloroplastic	Q9ZW12	NODE_9273	1.5357	98.35	71.3
Polyadenylate-binding protein 3	Q9LX90	NODE_9583	1.52	83.1	78.98
AT5g11000/T30N20_270	Q9LEU1	NODE_12912	1.5187	31.42	80.33
Protein TIFY 3B	Q9C5K8	chain_1667	1.5187	25.83	76.67
Ribulose bisphosphate carboxylase small chain 3B, chloroplastic	P10798	chain_56222	1.5167	61.9	85.42
DnaJ protein P58IPK homolog	Q9LYW9	NODE_1876	1.5154	93.36	77.99
At5g54760	Q9FFV1	chain_1361	1.5081	49.78	90.27
Adenylyl-sulfate kinase 3	Q9SRW7	chain_16173	1.5077	84.78	72.41
Probable protein phosphatase 2C 49	Q3EAF9	NODE_6863	1.5062	98.54	71.26
4-hydroxyphenylpyruvate dioxygenase	P93836	chain_14718	1.4987	91.53	89.53
At1g27330	Q84K46	NODE_19259	1.4968	70.41	85.29
ARP protein	Q9FX95	chain_6002	1.4963	94.58	81.97
AT3g23600/MDB19_9	Q9LUG8	chain_4088	1.493	67.12	65.38
Probable GABA transporter 2	Q8L4X4	NODE_2742	1.4903	88.6	80.0
Heat stress transcription factor B-2b	Q9T0D3	NODE_9373	1.4877	58.79	70.83
At3g16510	Q9LK74	NODE_7227	1.4873	36.42	67.59
ARM repeat superfamily protein	F4IAF6	chain_42866	1.4818	96.79	72.14

continued on the next page

Contigs promoted at 34°C (*continued*)

Name	UniProt ID	Contig	$\log_2$ (fold-Change)	Target coverage (%)	Alignment identity (%)
5-methyltetrahydropteroyltriglutamate–homocysteine methyltransferase 2	Q9SRV5	chain_3539	1.4787	98.8	87.09
Aldose 1-epimerase family protein	Q9LXG7	NODE_4894	1.4779	82.83	66.42
Trihelix transcription factor ASIL2	Q9LJG8	NODE_9958	1.4738	44.44	64.04
Cytochrome c oxidase subunit 1	P60620	NODE_19480	1.4668	100	98.95
Potassium transporter 8	Q9M7J9	chain_14161	1.4653	91.43	77.78
Elongation factor G, putative (DUF668)	Q8L5Y3	NODE_1229	1.4597	72.45	65.26
Probable 2-oxoacid dependent dioxygenase	Q9SKK4	NODE_49341	1.4551	42.17	64.71
Protein PLASTID REDOX INSENSITIVE 2, chloroplastic	Q9XIK0	NODE_10421	1.455	59.68	75.45
Zinc finger A20 and AN1 domain-containing stress-associated protein 5	Q9LHJ8	chain_849	1.4493	55.73	89.9
Probable mediator of RNA polymerase II transcription subunit 37c	Q9LHA8	chain_10637	1.4479	91.67	75.15
Probable cysteine protease RD19D	Q8VYS0	chain_12134	1.4478	90.2	73.26
Calcium-binding EF-hand family protein	Q9T0I9	chain_44395	1.4461	85.82	73.05
Peroxisomal acyl-coenzyme A oxidase 1	O65202	chain_5896	1.4397	98.13	82.03
Regulator of chromosome condensation (RCC1) family with FYVE zinc finger domain-containing protein	A0A1P8BCA2	chain_2746	1.4301	94.43	76.99
Uncharacterized protein AT4g30230	Q9SUM1	NODE_6507	1.4262	11.88	73.33
AT5g53570/MNC6_11	Q94BY9	chain_6447	1.4219	60.76	70.42
Chorismate mutase 2	Q9S7H4	NODE_7928	1.4139	98.95	75.9
Formate dehydrogenase, chloroplastic/mitochondrial	Q9S7E4	chain_10896	1.4062	49.83	90.41
Probable sucrose-phosphate synthase 4	F4JLK2	chain_9145	1.4027	72.91	85.06
CSC1-like protein RXW8	F4IBD7	chain_8850	1.3935	80.23	75.89
Stromal cell-derived factor 2-like protein	Q93ZE8	chain_2915	1.3883	91.53	79.44
ABC transporter G family member 29	Q94A18	chain_6410	1.3719	70.54	73.68
Protein HHL1, chloroplastic	Q8LDL0	chain_5986	1.3719	96.21	85.19
O-Glycosyl hydrolases family 17 protein	Q8GYS2	chain_9256	1.3674	87.27	80.52

continued on the next page

Contigs promoted at 34°C (*continued*)

Name	UniProt ID	Contig	$\log_2$ (fold-Change)	Target coverage (%)	Alignment identity (%)
Nudix hydrolase 15, mitochondrial	Q8GYB1	chain_4435	1.3657	80.06	79.83
AT4G30480 protein	Q9M0B2	chain_4507	1.3588	58.63	75.42
Probable serine/threonine-protein kinase PBL7	Q0WRY5	chain_9704	1.3397	75.3	84.39
E3 ubiquitin protein ligase DRIP2	Q94AY3	NODE_3492	1.3344	78.85	73.77
Protein DJ-1 homolog B	Q9MAH3	chain_4995	1.3151	90.86	72.96
Cytochrome P450, family 716, subfamily A, polypeptide 1	Q9LVY7	chain_2125	1.3035	75.1	71.04
Protein LYK5	O22808	NODE_1616	1.2696	89.44	72.8
5-methyltetrahydropteroyltriglutamate–homocysteine methyltransferase 2	Q9SRV5	chain_1175	1.2638	98.46	86.27
Gb—AAD20086.1	Q9FND0	chain_7334	1.2632	72.0	77.42
Protein SUPPRESSOR OF MAX2 1	Q9FHH2	NODE_1064	1.2491	29.69	72.58
At1g63660	Q9CAD1	NODE_2694	1.2456	100.0	82.17
At1g10410/F14N23_31	Q8LPT2	NODE_1050	1.2397	37.31	70.35
Synaptotagmin-1	Q9SKR2	chain_4777	1.2211	92.36	74.19
PHD finger family protein / SWIB complex BAF60b domain-containing protein / GYF domain-containing protein	A0A1P8B2P9	chain_2645	1.2151	43.86	67.74
Derlin-1	Q8VZU9	NODE_6291	1.2063	74.46	80.12
Eukaryotic aspartyl protease family protein	Q9M8R6	chain_5067	1.2018	96.63	67.76
3-hydroxyisobutyryl-CoA hydrolase-like protein 5	Q9SHJ8	chain_2509	1.1808	95.54	70.14
